# Interactome Analyses of Mature γ-Secretase Complexes Reveal Distinct Molecular Environments of Presenilin (PS) Paralogs and Preferential Binding of Signal Peptide Peptidase to PS2*[Fn FN2]

**DOI:** 10.1074/jbc.M112.441840

**Published:** 2013-04-15

**Authors:** Amy Hye Won Jeon, Christopher Böhm, Fusheng Chen, Hairu Huo, Xueying Ruan, Carl He Ren, Keith Ho, Seema Qamar, Paul M. Mathews, Paul E. Fraser, Howard T. J. Mount, Peter St George-Hyslop, Gerold Schmitt-Ulms

**Affiliations:** From the ‡ Departments of Laboratory Medicine and Pathobiology, Neurology, Physiology, Medical Biophysics, and Medicine (Neurology), Tanz Centre for Research in Neurodegenerative Diseases, University of Toronto, Ontario M5S3H2, Canada,; the § Cambridge Institute for Medical Research, University of Cambridge, Cambridge CB2 0XY, United Kingdom, and; the ¶ New York University School of Medicine, Orangeburg, New York 10962

**Keywords:** Alzheimers Disease, Mass Spectrometry (MS), Presenilin, Proteomics, Secretases, Transgenic, Interactome

## Abstract

γ-Secretase plays a pivotal role in the production of neurotoxic amyloid β-peptides (Aβ) in Alzheimer disease (AD) and consists of a heterotetrameric core complex that includes the aspartyl intramembrane protease presenilin (PS). The human genome codes for two presenilin paralogs. To understand the causes for distinct phenotypes of PS paralog-deficient mice and elucidate whether PS mutations associated with early-onset AD affect the molecular environment of mature γ-secretase complexes, quantitative interactome comparisons were undertaken. Brains of mice engineered to express wild-type or mutant PS1, or HEK293 cells stably expressing PS paralogs with N-terminal tandem-affinity purification tags served as biological source materials. The analyses revealed novel interactions of the γ-secretase core complex with a molecular machinery that targets and fuses synaptic vesicles to cellular membranes and with the H^+^-transporting lysosomal ATPase macrocomplex but uncovered no differences in the interactomes of wild-type and mutant PS1. The catenin/cadherin network was almost exclusively found associated with PS1. Another intramembrane protease, signal peptide peptidase, predominantly co-purified with PS2-containing γ-secretase complexes and was observed to influence Aβ production.

## Teaser

**Background:** The human genome codes for two presenilin (PS) paralogs, PS1 and PS2.

**Results:** PS paralogs are embedded in overlapping but distinct molecular environments, with signal peptide peptidase (SPP) primarily binding to PS2.

**Conclusion:** Study paves the way for understanding functional divergence of PS paralogs.

**Significance:** Example of an interaction between a Type I- and Type II-directed intramembrane protease.

## Introduction

A defining pathological hallmark of Alzheimer disease (AD)^5^ is the deposition of extracellular plaques, largely consisting of the 38 to 43 amino acid amyloid β-peptide (Aβ). Aβ is generated by consecutive cleavages of the amyloid precursor protein (APP) by two proteolytic activities, β-secretase and γ-secretase. γ-Secretases are membrane-embedded multiprotein complexes consisting of at least four different proteins (presenilin, nicastrin, Pen-2, and Aph-1) proposed to be present in single copies in the mature complex (1, 2). Presenilins (PS) are ∼50-kDa membrane proteins that are thought to adapt a 9-transmembrane topology and harbor the aspartate-based catalytic APP cleavage activity of the γ-secretase core complex (3–5). The proteolytic activity of presenilins cleaves Type-I transmembrane proteins at sites within their respective membrane-spanning domains (1, 6, 7). Although initially considered unique in their ability to carry out regulated intramembrane proteolysis, it has become apparent that presenilins together with the signal peptide peptidases (SPPs) are members of an ancient family of aspartyl intramembrane proteases. Other intramembrane proteases include site-2 proteases and the rhomboids (8, 9). Assuming a 1:1:1:1 stoichiometry of its four core constituents, the γ-secretase core complex is expected to have a mass of less than 200 kDa. The observed size of active γ-secretase has, dependent on the methodology employed, been estimated to range from 200 kDa to more than 1 MDa. It is currently unclear what accounts for this discrepancy. However, more than a dozen proteins have been proposed to interact and, at least temporarily, associate with this protein complex. In previous work we have, for example, shown that TMP21 is a protein that binds to the γ-secretase complex and modulates its APP cleavage activity (10). Others have reported interactions with CD147 (10–12), the cadherin/catenin adhesion system (13–17), gSAP (18), and a subset of tetraspanin proteins, including CD81 and Upk1b (19).

Most investigations that explored the composition of the γ-secretase complex published to date have not taken into account the fact that the human genome codes for two *PSEN* genes, *PSEN1* on chromosome 14 and *PSEN2* on chromosome 1 (20–22). Little is known about how these alternative gene products contribute to the assembly of distinct subpopulations of γ-secretase complexes. Previous evidence suggested that PS1 and PS2 paralogs, which exhibit 67% amino acid sequence identity, carry out distinct but overlapping functions (23). In support of this notion, the two PS paralogs (i) display different expression profiles, with PS1 expression highest in testis and lung, and PS2 expression highest in heart, pancreas, and brain (24); (ii) generate distinct knock-out phenotypes, with PS1 knock-out mice characterized by late embryonic lethality, disturbed somitogenesis, cranial hemorrhage, and PS2 knock-out mice being viable and fertile but exhibiting mild pulmonary fibrosis and hemorrhage with age (25, 26); (iii) display differences in APP processing and γ-secretase activity (27, 28); and (iv) may influence distinct signaling pathways, with PDGF signaling, for example, being influenced only by PS2 (29). The question arises whether differences in protein-protein interactions that distinct γ-secretase complexes engage in can explain differences in their biology and serve as starting points for refining therapeutic approaches, which may selectively target their APP cleavage activity.

We report on a quantitative comparative analysis of wild-type and L286V mutant PS1-containing γ-secretase complexes purified from mice engineered to express near physiological levels of these bait proteins (30). We further report on the gentle purification of active PS-containing γ-secretase complexes from HEK293 parental cells that express PS1 or PS2 variants equipped with an N-terminal tandem affinity purification (TAP) tag in the context of endogenous nicastrin, Aph-1, and Pen-2. Interactome data tables confirmed a number of previously reported PS interactors, shed doubt on others, and revealed predominant co-enrichment of the catenin/cadherin molecular machinery with PS1-containing complexes. Surprisingly, SPP was primarily associated with PS2-containing complexes (8, 9, 31). Subsequent biochemical validation experiments confirmed a bias of SPP for co-purifying with the PS2 paralog and established an influence of SPP levels on the cellular release of Aβ.

## EXPERIMENTAL PROCEDURES

### 

#### 

##### Lentiviral Expression System

The TAP tag cassette was amplified from pRV_NTAP (32) through PCR with the forward primer, TTTTGGATCCGACCATGGGCACCCCCGCAGTCAC, and backward primer, TTTTTGAATTCCCGGCTCGCGCTGCCC. Human PS1 was amplified with TTTTTCTGCAGACAGAGTTACCTGCAC and TTTTTCTCGAGCTAGATAAAATTGA from pCMV_PS1. Human PS2 was amplified with primer pair TTTTTCTCGAGTCAGATGTAGAGCTGATGG and TTTTTGAATTCTGCTCACATTCATGGCCTCTGAC. TAP tag, PS1, or PS2 PCR products were digested with the restriction enzymes BamHI/EcoRI and PstI/XhoI (New England Biolabs, Ipswich, MA), respectively, and inserted into the pcDNA4 eukaryotic expression vector pre-digested with the same restriction enzymes. Subsequently, TAP-PS cassettes assembled in this manner were amplified by PCR, the resulting products were digested with NdeI/BamHI and transferred into the pre-cleaved cloning cassette of the lentiviral pWPI.Neo.MCS+ vector. Lentiviral particles were generated by transfecting HEK293T cells with the CalPhos transfection reagent kit (Clontech, Mountain View, CA) and harvesting the cell medium after 2 days of incubation. Subsequently, lentivirus particles were enriched by ultracentrifugation (Beckman SW32ti) at 120,000 × *g* for 2 h at 4 °C, and HEK293F cells were transduced overnight with lentivirus particles. After an additional 24 h of incubation, a neomycin-based selection of successfully transduced cells was initiated by the addition of antibiotic selection marker G418 (Invitrogen) to the cell medium. Following clonal selection by the dilution method, individual clones were analyzed by Western blot analyses to confirm PS1 or PS2 expression. For an integrant clone to be selected for downstream interactome analyses it had to express the TAP-tagged PS paralog at near physiological levels and demonstrate a degree of PS endoproteolysis comparable with endogenous wild-type PS.

##### Antibodies

Mouse monoclonal anti-PS1 IgG1 antibody (NT1) directed against human residues 41–49 (amino acids RRSLGHPEP) does not cross-react with mouse PS1 and was provided by PMM. Affinity-purified polyclonal rabbit anti-PS1-NTF (A4) antibody was provided by PEF. Commercially obtained were rabbit polyclonal anti-SPP (Abcam, Cambridge, MA), anti-Nct (Sigma), anti-Aph-1 (O2C2; Affinity Bioreagents), and anti-Pen-2 (Anaspec, Fremont, CA) antibodies.

##### Purification from Transgenic Mice Expressing Human Wild-type or Mutant PS1 Variants

Transgenic mice expressing wild-type or L286V mutant human PS1 from a prion promoter has been described (30). 10 brains each of age-matched (12 week old) transgenic mice expressing wild-type PS1 or PS1-L286V were rapidly dissected. Brains were cut into 1-mm^3^ pieces with a razor blade and pieces further ground to dust with a pestle and mortar. Cells were lysed in 25 mm HEPES, pH 7.4, 150 mm NaCl, 2 mm EDTA, protease inhibitor mixture (Roche Applied Science), insoluble membranes were harvested by centrifugation and, subsequently dissolved with 1% (w/v) CHAPSO (Anatrace, Maumee, OH) and 0.05% (w/v) DDM (Anatrace). The sample was incubated on ice for 1 h and insoluble material was removed by centrifugation at 12,000 × *g* for 10 min. The supernatant was diluted in the CHAPSO/DDM buffer used for lysis to a total protein concentration of 0.5 mg/ml (BCA Protein Assay, Pierce/Thermo Scientific). Solubilized membrane proteins were loaded overnight at 4 °C onto wheat germ agglutinin (WGA) lectin resin (Vector Laboratories, Burlingame, CA) using a 1 to 15 (v/v) ratio of resin to eluate. The WGA resin was collected in a disposable column and washed with CHAPSO/DDM lysis buffer. Complexes were eluted by the addition of 4 volume eq (relative to the WGA resin wet volume) of 0.5 m *N*-acetyl-d-glucosamine (Sigma) in CHAPSO/DDM lysis buffer and the eluate split in two. One part of the eluate was incubated with NT-1 antibody resin the other part with cognate peptide-saturated NT-1 antibody resin (which served as a negative control) for 2 h at 4 °C. The resin was sedimented by gravity, washed twice with CHAPSO/DDM lysis buffer, and once with lysis buffer in which CHAPSO/DDM had been replaced with 0.5% (w/v) DDM. Finally, purified complexes were eluted from the antibody resin by pH drop elution with 0.2% trifluoroacetic acid and 20% acetonitrile, pH 1.9.

##### Purification Procedure from HEK293F Cells

HEK293F cells stably expressing TAP-PS1 or TAP-PS2 were adapted for growth in serum-free suspension cultures. Cells were harvested and lysed in 25 mm HEPES, pH 7.4, 150 mm NaCl, 2 mm EDTA, protease inhibitor mixture (Roche Applied Science) with 0.25% (w/v) CHAPSO (Anatrace, Maumee, OH) and 0.05% (w/v) DDM (Anatrace). The sample was incubated on ice for 1 h and insoluble material was removed by centrifugation at 12,000 × *g* for 10 min. The supernatant was diluted in the CHAPSO/DDM buffer used for lysis to a total protein concentration of 0.5 mg/ml (BCA Protein Assay, Pierce/Thermo Scientific, Rockford, IL). A 1:2000 volume eq of pre-washed IgG-resin was added to the diluted supernatant. Following overnight incubation with gentle rotation at 4 °C, the resin was collected in a disposable column (Bio-Rad) and washed with a 10-fold volume (relative to the IgG-resin wet volume) of CHAPSO/DDM lysis buffer. Subsequently, a suspension of 1:1 resin to CHAPSO/DDM lysis buffer was incubated for 2 h at 4 °C with 1 units/ml of tobacco etch virus (TEV) protease (Invitrogen) and 1 mm DTT (Invitrogen TEV kit). The TEV cleavage step was repeated once and the ensuing two TEV eluates were combined and loaded overnight at 4 °C onto WGA lectin resin (Vector Laboratories, Burlingame, CA) using a 1 to 15 (v/v) ratio of resin to eluate. The WGA resin was collected in a disposable column and washed with CHAPSO/DDM lysis buffer. Complexes were eluted by the addition of 4 volume eq (relative to the WGA resin wet volume) of 0.5 m *N*-acetyl-d-glucosamine (Sigma) in CHAPSO/DDM lysis buffer and the eluate split in two. One part of the eluate was incubated with streptavidin resin (Pierce) the other part with biotin-saturated streptavidin resin (which served as a negative control) for 2 h at 4 °C. The resin was sedimented by gravity, washed twice with CHAPSO/DDM lysis buffer, and once with lysis buffer in which CHAPSO/DDM had been replaced with 0.5% (w/v) DDM. Finally, purified complexes were eluted from the streptavidin resin with 2 mm biotin in the aforementioned DDM buffer.

##### Western Blot Analyses and Antibodies

Samples were prepared in SDS sample buffer, separated on 4–12% precast BisTris gels (Invitrogen), and transferred to nitrocellulose membranes. Proteins were detected by enhanced chemiluminescence (ECL) following incubations with primary antibodies and peroxidase-conjugated secondary antibodies.

##### Protein Reduction, Alkylation, and Trypsinization

Eluates from multistep purifications were concentrated to a volume of 5 μl in a speed vacuum concentrator (Thermo Scientific, Waltham, MA). Samples were subsequently denatured in the presence of 9 m urea for 10 min at room temperature, followed by reduction with 5 mm tris-(2-carboxyethyl)-phosphine for 30 min at 60 °C and alkylation with 9 mm 4-vinylpyridine for 1 h at room temperature in the dark. Next, samples were diluted 5-fold to ensure that the concentration of urea did not exceed 2 m for the subsequent trypsinization. The latter was initiated by the addition of 1% (w/w) of side chain-modified, l-1-tosylamido-2-phenylethyl chloromethyl ketone-treated porcine trypsin and allowed to proceed at 37 °C for 6 h.

##### Isotopic Tags for Relative and Absolute Quantitation (iTRAQ) Labeling of Peptides

Individual iTRAQ labeling reagents (Applied Biosystems, Foster City, CA) were reconstituted in ethanol according to the manufacturer's recommendation and added to peptide mixtures derived from the tryptic digestion of eluates and incubated at room temperature in the dark for 3 h with occasional mixing. Equal labeling with iTRAQ reagents was verified by documenting equal intensities of 114:115:116:117 iTRAQ signature ion mass peaks within collision-induced dissociation (CID) spectra assigned to a small number of peptides observed for trypsin that had undergone autolysis. Significant deviations from this ratio would have indicated problems with the purity of the iTRAQ reagents, the labeling reaction, or the recovery of individual samples prior to the sample mixing step.

##### Liquid Chromatography and Mass Spectrometry

Strong cation exchange chromatography was used to achieve peptide fractionation of the complex digest mixture. Sample preparation procedures, separation conditions, and mass spectrometry analyses were conducted as described (33).

##### Database Searches

Searches were performed using designated MS/MS data interpretation algorithms within ProteinPilot^TM^ (version 3; AB Sciex) and Mascot (version 2.2; MatrixScience). Modifications considered were oxidation of methionine, phosphorylations of serine, threonine, and tyrosine, N-terminal pyro-Glu, and alkylation with 4-vinylpyridine. Searches further considered up to one missed cleavage and charge states ranging from +2 to +4. A total of four biological replicates of brain PS1 interactome studies (from wild-type and mutant hPS1 transgenic mice) and TAP PS paralog experiments followed by downstream mass spectrometry analyses were carried out. Because repeat experiments were not conducted under exactly identical conditions but used different stringency of washing steps (see results for details), we chose to select representative data tables obtained for presenting these data and provide for each protein identified information on the number of times (of four near-identical replicates) a confident identification was made. Please note that the majority of proteins were identified on the basis of Mascot scores and ProteinPilot confidence assignments, which easily exceeded thresholds conventionally applied for confident identifications. The mass tolerance range between expected and observed masses used for database searches was ±150 ppm for MS peaks, and ±0.15 Da for MS/MS fragment ions when Mascot was used as the search engine. These relatively large thresholds were used to capture more of the low intense peaks that frequently display broader distribution and thus are assigned with lower mass accuracy. Threshold levels were optimized based on LC-MS/MS datasets of tryptic digests of standard proteins. All samples were searched against the mouse International Protein Index database, SwissProt, or the Ensemble database (releases: June 2011) and “decoy” databases in which all entries had been inverted. iTRAQ ratios were determined with quantitation algorithms embedded in software packages Mascot and ProteinPilot. Both software packages also contain a feature that was used to correct raw iTRAQ ratios for impurity levels of individual iTRAQ reagent lots determined by the manufacturer. Because the underlying variation in peptide enrichments cannot be assumed to have a Gaussian distribution, we employed the nonparametric Mann-Whitney *U* test (PASW Statistics version 18, IBM) to determine statistically significant differences between iTRAQ 115:114 and 117:116 enrichments for each identified interactor.

##### shRNA Knockdown Experiments

The SPP shRNA vector was acquired from Open Biosystems (Thermo Fisher Scientific/Open Biosystems, Huntsville, AL). HEK293 cells stably expressing Swedish APP were transfected with this vector and after 48 h incubation, the selection of cells expressing the shRNA in a stable manner was initiated by adding 1 μg/ml of puromycin (Invitrogen).

##### Aβ40/42 ELISA and Cell-free Assays

Determination of γ-secretase activity by a cell-free assay was performed as previously described (10). Briefly, samples were incubated with recombinant FLAG-tagged APP-C100. Aβ peptide generated by γ-secretase-dependent proteolysis was measured by ELISA according to the manufacturer's instructions (BIOSOURCE International/Invitrogen).

*SPP* cDNA (*Drosophila melanogaster*) was subcloned into pMAL4X vector to introduce an maltose-binding protein tag at the N terminus of the protein and a His_6_ tag at the C terminus. Transformed *Escherichia coli* C43 cells were harvested, resuspended in Buffer A containing, 20 mm HEPES, 150 mm NaCl, pH 7.5, with protease inhibitor mixture (Roche) followed by cell disruption in Constant Systems Cell Disruptor at 30 kpsi. Cell debris was removed by centrifugation at 10,000 × *g* and total cell membranes were harvested by centrifugation at 100,000 × *g* for 1 h at 4 °C. Membranes were homogenized in 20 mm Buffer A to a final protein concentration of 5.0 mg/ml. Buffer A containing 1% DM was used to solubilize the membrane for 3 h at 4 °C followed by centrifugation at 100,000 × *g* for 1 h to remove any unsolubilized material. Solubilized SPP in the supernatant was captured on amylose affinity resin (New England Biolabs) and eluted in 5 column volumes with Buffer A containing 0.2% DM and 10 mm maltose, concentrated, and loaded on a Superose 6 10/300 GL column. Fractions containing the protein were pooled and concentrated to 3.0 mg/ml for the activity assays.

##### Co-immunoprecipitation

For immunoprecipitation of SPP complexes, cell pellets were homogenized in Lysis buffer (25 mm HEPES, pH 7.4, 150 mm NaCl, 2 mm EDTA, 1% CHAPSO, protease inhibitor mixture). Lysates were diluted to a final concentration of 0.5% CHAPSO with a Lysis buffer formulation that lacked the chaotropic detergent. After a pre-clearing step with Protein G-Sepharose Fast Flow (GE Healthcare) for 1 h at 4 °C, lysates were subjected to immunoprecipitation with the SPP antibody. Co-immunoprecipitated proteins were captured by overnight incubation at 4 °C with Protein G-Sepharose Fast Flow. Next, the capture resin was washed 3 times with Lysis buffer adjusted to 0.5% CHAPSO and once with PBS. Captured proteins were eluted in the presence of reducing SDS-PAGE sample buffer and subjected to Western blot analyses.

## RESULTS

### 

#### 

##### Strategy for Quantitative Interactome Mapping of Wild-type and Mutant PS1 in Mice

**FIGURE 1.**
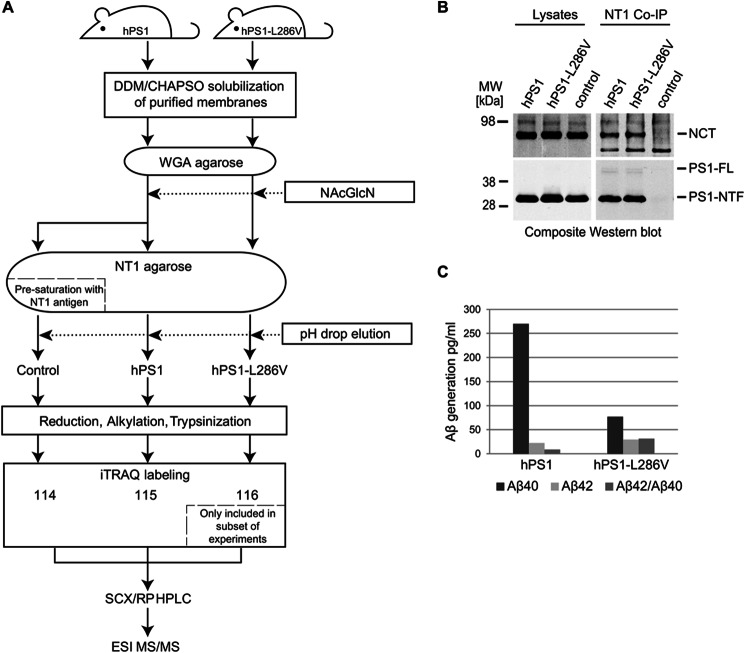
**Strategy for purification of PS1-containing γ-secretase complexes from transgenic mice expressing near physiological levels of wild-type or mutant PS1.** *A,* the presence of *N*-glycans on mature nicastrin is exploited for the capture of mature γ-secretase complexes on WGA-agarose. Next, bait protein complexes are eluted in the presence of an excess of *N*-acetylglucosamine (NAcGlcN) and loaded onto a pre-generated NT1-immunoaffinity matrix. *B,* pilot experiment that documented successful immunoaffinity capture of γ-secretase complexes with anti-PS1 monoclonal NT1 antibody that selectively recognizes human PS1. *C,* relative activity of purified complexes in a standard ELISA that monitors the production of Aβ peptides and can distinguish between Aβ40 and Aβ42 variants.

Brains of transgenic mice, which expressed human wild-type PS1 or mutant PS1-L286V, known to cause inherited early onset AD in humans, were used as biological source material. The analysis was to be based on an immunoprecipitation strategy followed by mass spectrometry of co-purifying bait protein interactors. Because the method targeted unmodified bait proteins, the latter objective had to rely on intrinsic features of the known γ-secretase core components for purification. Thus, lectin affinity chromatography based on WGA resin was employed to capture maturely glycosylated nicastrin and thereby enrich fully assembled γ-secretase complexes (34–37). A second orthogonal capture step was based on the PS1 bait protein and made use of a high-affinity monoclonal antibody (NT1) that selectively recognizes a short epitope within the N-terminal domain of human PS1 (residues 41–49, amino acid sequence “RRSLGHPEP”) not present in mouse PS1. To generate a negative control for the downstream interactome analysis, half of the WGA eluate sample was side by side incubated with an identical NT1 immunoaffinity matrix that had been pre-saturated with the synthetic peptide antigen the NT1 antibody had been raised against (Fig. 1*A*). Following tryptic digestion, peptides in negative control samples and specific samples were conjugated to distinct isotopic tags for relative and absolute quantitation (iTRAQ) using the following assignment of labels to samples: iTRAQ114, negative control; iTRAQ115, PS1; iTRAQ116, PS1 mutant. This approach allowed us to combine and analyze multiple samples concomitantly, capitalizing on the fact that the relative contribution of a given sample to the downstream identification of individual peptides can be determined on the basis of signature mass ions unique for each iTRAQ conjugate (38). To deal with the relative high complexity, the iTRAQ-labeled sample mixture was fractionated by two-dimensional liquid chromatography and introduced by electrospray ionization (ESI) into a quadrupole time-of-flight tandem mass spectrometer.

Small-scale pilot experiments established that the levels of expression of wild-type PS1 and its mutant counterpart in the respective transgenic mouse models was comparable and that the epitope recognized by the NT1 antibody is indeed accessible in mature γ-secretase complexes under physiological conditions (Fig. 1*B*). A comparative analysis of γ-secretase activities in solubilized membrane fractions established that the complexes containing human wild-type or mutant PS1 were active. As expected, further characterization of the respective activities with an ELISA, which can distinguish between Aβ40 and Aβ42 cleavage products, revealed that γ-secretase complexes containing human wild-type PS1 had a higher overall activity but led to a lower Aβ42:Aβ40 ratio than the respective complexes based on mutant PS1 (Fig. 1*C*).

##### Wild-type PS1 Binds to Members of the Catenin/Cadherin Family, the Synaptic Fusion Machinery, and the Lysosomal ATPase Complex

**FIGURE 2.**
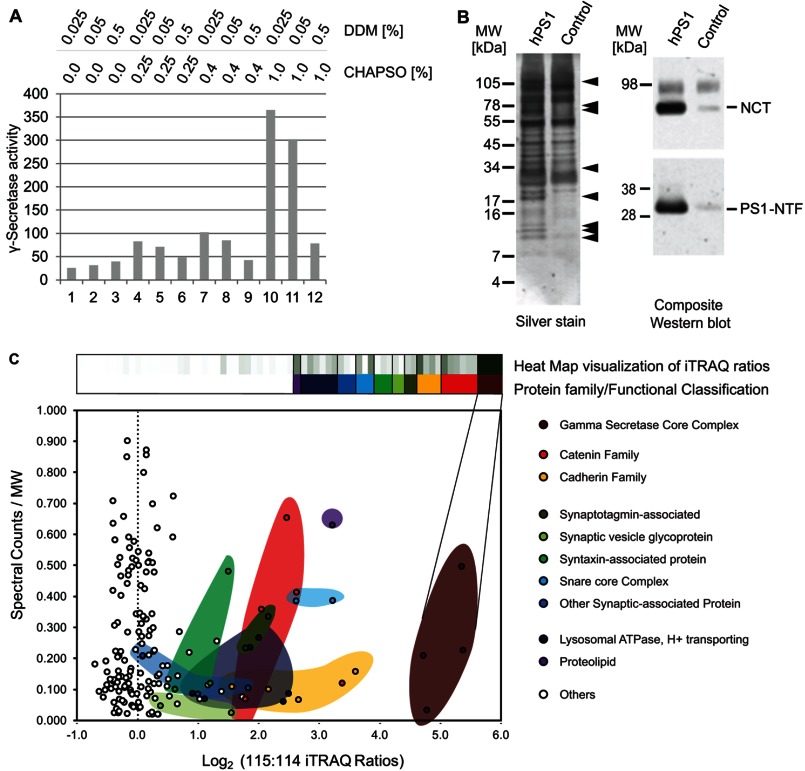
*A,* activity-based optimization of the detergent mixture for purification of active γ-secretase complexes from mouse brain. Brains from transgenic mice expressing wild-type human PS1 were homogenized in the absence of detergent, the fraction was enriched for cellular membranes obtained by centrifugation and proteins extracted by the addition of defined combinations of DDM or CHAPSO. *B,* analysis of eluate fractions obtained after two-step purification (see Fig. 1*A*) by denaturing SDS-PAGE followed by silver staining or immunoblotting. *C*, chart depicting bait-specific enrichment of proteins within interactome data set (based on iTRAQ signature mass ion intensity ratios) against spectral counts underlying identification of individual proteins (normalized by molecular weight). Note that each signal in the graph represents a protein. Color shading is used to indicate members of protein families or to group proteins based on their known functional association. See Table 1 for details of candidate interactors identified and the assignment of proteins to heat map values indicated *above* the graph.

Various detergents and detergent mixtures were tested to formulate a lysis buffer composition most compatible with obtaining stable and active γ-secretase complexes. Mixtures of 1% CHAPSO and 0.025–0.05% DDM emerged as the detergent conditions that best solubilized γ-secretase from mouse brain membranes (Fig. 2*A*).

A first series of large-scale interactome analyses focused on uncovering the molecular environment of wild-type PS1 alone and employed relatively mild conditions for pre-elution wash steps of the affinity matrix (referred to as “low stringency” in the tables). 1% Aliquots of the final eluates were subjected to denaturing SDS-PAGE followed by silver stain analysis or Western blotting with antibodies, which detect nicastrin or the N terminus of PS1 (Fig. 2*B*). The analysis documented that the two-step purification method had led to a strong enrichment of PS1 and nicastrin, indicative of the presence of mature γ-secretase complexes, and established that only trace amounts of these two proteins could be seen in the nonspecific eluate fraction. Differences in silver-stained protein profiles indicated that multiple protein bands were specifically co-enriched together with PS1. However, this analysis also confirmed the anticipation that a considerable number of proteins would bind to the affinity matrix nonspecifically, emphasizing the need to incorporate isobaric peptide labels into the downstream sample work-up scheme.

Computational searches of the International Protein Index database with masses extracted from CID spectra were used to generate an initial non-curated interactome dataset containing specific and nonspecific interactors. Searches against a “decoy database” in which all sequence entries were inverted did not give rise to any protein identification that passed significance thresholds that had been applied. When biological replicates of the study were undertaken, and the proteins were sorted according to their known classifications and functional associations, ∼80 proteins were repeatedly seen in the wild-type PS1 sample (Fig. 2*C*, Table 1, supplemental Table S1). In subsequent biological replicates a higher number of washing steps (referred to as “high stringency” condition) was applied to the affinity matrix upon capture of bait protein complexes to reduce the number of weak or nonspecific interactors (Table 1).

**TABLE 1:**
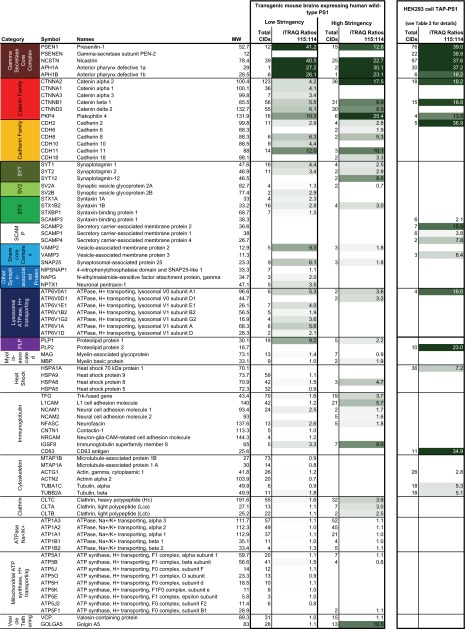
**Summary of data tables comparing the molecular environment of PS1 in transgenic mouse brains or HEK293 cells**

On the basis of iTRAQ 115:114 ratios it was evident that members of the cadherin/catenin network of calcium-dependent cell adhesion molecules co-purified robustly with wild-type PS1 (Table 1). In particular, catenin α2, catenin β1, and plakophilin 4, as well as the cadherins 2 and 11 were repeatedly and strongly enriched in the PS1-specific sample. It further was evident that proteins that contribute to a molecular machinery that targets and fuses synaptic vesicles to cellular membranes were co-purifying with PS1. In addition, proteolipid protein 1 and multiple subunits belonging to V0 and V1 macro-subcomplexes of the H^+^-transporting lysosomal ATPase co-enriched together with PS1. Interestingly, the H^+^-transporting mitochondrial ATPase, known to acquire a similar molecular architecture to its lysosomal counterpart was robustly detected but, apparently, was captured nonspecifically by the affinity matrix as it did not selectively co-enrich with PS1. Finally, cell adhesion molecules harboring fibronectin-type 3 or IgG-like subdomains, heat shock proteins and constituents of clathrin triskelia were modestly but inconsistently enriched in wild-type PS1 eluates.

Other proteins mostly gave rise to iTRAQ 115:114 signature mass ion ratios near ∼1, which exposed them as nonspecific interactors to the affinity matrix. Note that some of these nonspecific interactors, including members of the Na/K-ATPase, the aforementioned mitochondrial H^+^-transporting ATPase, or valosin-containing protein, were observed with high spectral counts.

##### Identical Molecular Environment of Wild-type PS1 or Mutant PS1-L286V

A next series of experiments aimed to uncover whether the molecular environments are different for mature γ-secretase complexes comprising wild-type or mutant PS1. Differences in the characteristics with which wild-type or mutant PS1-containing γ-secretase complexes cleave an APP-related substrate (Fig. 1*C*) may manifest as differences in protein-protein interactions or can be based on subtle differences in the molecular architecture of the catalytic cleavage center. To our knowledge these alternative scenarios had not been experimentally addressed by quantitative interactome analyses. Here, side by side interactome analyses of wild-type and PS1-L286V complexes repeatedly failed to uncover differences in the proteins that co-enriched with wild-type or mutant PS1 bait proteins (supplemental Table S2). More specifically, when on the basis of iTRAQ signature mass peak intensities the levels of enrichment were compared for proteins that co-purified with wild-type (iTRAQ 115:114 ratio) or mutant PS1 (116:114 ratio), no significant difference in enrichment levels (iTRAQ 116:115 ratio) was observed for any candidate interactor.

Taken together, the mouse brain-based PS1 interactome analyses described uncovered many robust and shared interactors of mature γ-secretase complexes harboring wild-type or mutant PS1 but did not reveal whether these interactions occur in a single cell-type or represent the cumulative molecular environment PS1 encounters across multiple cell types in the brain. Also, experiments up to this point did not address whether differences exist in the interactomes of PS1 and PS2.

##### Biochemical Analyses of PS1 or PS2 Complexes

**FIGURE 3.**
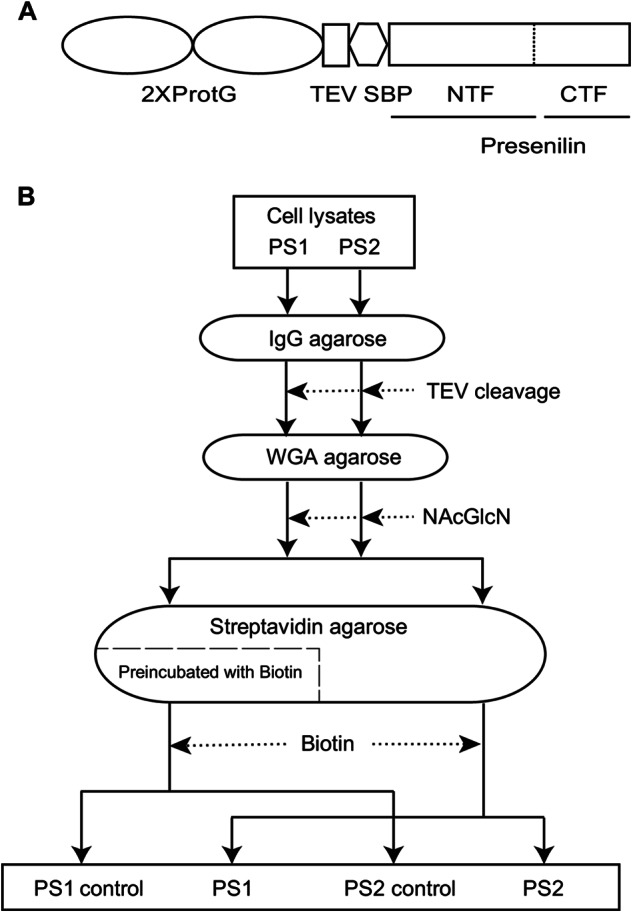
*A,* schematic representation of the Protein G-TEV-SBP TAP tag that consists of two IgG binding domains of *S. aureus* Protein G (*ProtG*) and a SBP separated by a TEV protease cleavage site. *B*, flow chart depicting key steps of purification strategy. The TAP-tagged PS1 or PS2 bait proteins were captured on IgG resin by means of their tandem ProtG moieties. Following the release of bait protein-containing complexes by TEV cleavage, the presence of *N*-glycans on mature nicastrin is exploited for capture of mature γ-secretase complexes on wheat germ agglutinin-agarose. Next, bait protein complexes are eluted in the presence of an excess of *N*-acetylglucosamine (NAcGlcN), followed by recapture on streptavidin resins by means of the SBP. Finally, purified complexes were obtained following their competitive displacement from the streptavidin resin in the presence of an excess of biotin.

To compare the molecular environments of PS1 and PS2 and the activity of γ-secretase complexes harboring these paralogs, we made use of human embryonic kidney cells (HEK293), a cell model frequently employed for γ-secretase studies. Although excellent antibodies for the detection or immunocapture of PS1 or PS2 are available, the benefit of working with endogenous proteins might in this application be outweighed by the risk to inadvertently introduce sample to sample variance through the use of non-identical antibody capture reagents with idiosyncratic cross-reactivities. To avoid this confounder a TAP tag comprised of tandem IgG binding domains derived from *Staphylococcus aureus* Protein G and a streptavidin-binding peptide (SBP) separated by a TEV protease cleavage site were attached to the N terminus of PS1 or PS2 bait proteins (Fig. 3*A*). Previous work by others documented this particular TAP tag to afford gentle purification of transiently interacting partners under near native conditions and to give rise to enhanced yields for the purification of mammalian protein complexes (32). An N-terminal attachment site was selected for tagging PS1 and PS2 on the basis of prior data, which demonstrated partial loss of γ-secretase activity in the presence of modifications to the C termini of presenilins (39). To achieve near physiological conditions of bait protein expression, lentiviral integrant clones were selected that process full-length presenilins into N- and C-terminal fragments at a level comparable with endogenous PS1 or PS2 (40).

**FIGURE 4.**
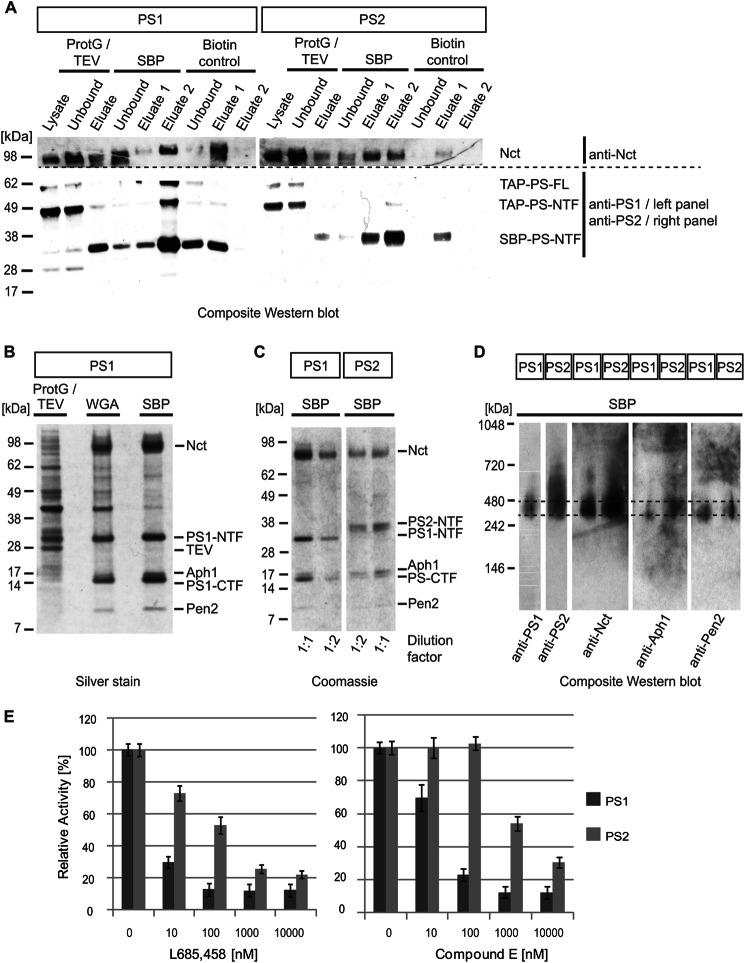
**Purification of TAP-tagged γ-secretase complexes.** *A*, Western blot analyses of informative fractions collected during key steps of a multistep purification procedure. The successful incorporation of the heterologously expressed TAP-PS proteins into mature γ-secretase complexes can be estimated by the relative intensities of signals attributed to the TAP-PS-FL precursors that migrated with an apparent mass of 58–62 kDa and the faster migrating TAP-PS-NTF cleavage products seen at 46–50 kDa. *B,* denaturing SDS-PAGE analysis followed by silver staining of three representative eluate fractions collected during the multistep purification of PS1-containing γ-secretase complexes. *C*, denaturing SDS-PAGE analysis of final streptavidin-agarose eluates followed by Coomassie staining. To facilitate assessment of the relative amounts of γ-secretase core components present two different volumes of each eluate fraction were subjected to analysis. Note the expected differences in migration of the N-terminal fragments (*NTF*) of PS2 and PS1 and the absence of unprocessed full-length TAP-PS signals in these fractions. *D,* nondenaturing Blue Native polyacrylamide gel analysis documenting migration of purified PS1- and PS2-containing γ-secretase complexes in single bands of similar apparent *M*_r_. *E,* PS1-containing γ-secretase complexes exhibit stronger responsiveness to established γ-secretase inhibitors in an *in vitro* ELISA that monitors the release of Aβ40 from a recombinant APP-C100 substrate.

Following TAP-based isolation of PS paralog-specific γ-secretase complexes the success of the purification was evaluated by immunoblot analyses of key fractions (Fig. 4*A*). In gel trypsinization and tandem mass spectrometry analyses of silver-stained bands (not shown) confirmed both PS1- and PS2-containing γ-secretase core complexes to be primarily comprised of nicastrin, presenilin, Aph-1, and Pen-2 (Fig. 4*B*). The side by side comparison of denatured complex components further revealed that the relative abundance and migration of proteins that constitute the γ-secretase core is shared between PS1- and PS2-containing γ-secretase complexes. The only γ-secretase core subunits that migrated with different apparent *M*_r_ were the PS paralogs themselves, an observation that had been anticipated based on differences in the primary structures of PS1 and PS2 (Fig. 4*C*). To compare molecular weights of native PS1- and PS2-containing γ-secretase complexes and assess their integrity and heterogeneity Blue Native gel analyses followed by Western blotting were conducted (Fig. 4*D*). These analyses revealed that both PS1- and PS2-containing γ-secretase complexes migrated at a single molecular mass of ∼350 kDa in the presence of mixed micelles composed of CHAPSO and DDM. Consistent with the notion that both γ-secretase preparations largely contained intact mature complexes, Blue Native gel analyses did not reveal the presence of molecular species that migrate with lower apparent *M*_r_. Furthermore, a standard *in vitro* γ-secretase activity assay based on a recombinant APP-C100-FLAG substrate documented that PS1- and PS2-containing γ-secretase complexes were active but also were responsive to inhibition by small-molecule pharmacological inhibitors (Fig. 4*E*). More specifically, when adjusted for total amounts of presenilins present, purified γ-secretase complexes containing PS1 exhibited ∼30% higher absolute cleavage activity toward the APP-C100-FLAG substrate than the respective complexes containing PS2. The cleavage activity of both types of complexes toward the APP-C100-FLAG substrate could be inhibited in a concentration-dependent manner in the presence of both L685,458, a transition state analog inhibitor (41), or Compound E, a non-transition state analog inhibitor (42). However, when activity charts were normalized and plotted based on relative activity levels of naive purified complexes, PS2-containing complexes required higher inhibitor concentrations for achieving comparable levels of inhibition.

##### PS1- or PS2-containing γ-Secretase Interactomes

To generate a comprehensive comparative interactome data set of PS1- and PS2-containing γ-secretase complexes, streptavidin eluate fractions were in subsequent experiments not subjected to gel-based analyses but directly trypsinized in solution. Negative control samples were in these studies generated by passing 50% of WGA eluates over a streptavidin resin, which had been pre-saturated with biotin (Fig. 3*B*). As for the mouse brain interactome studies of PS1, peptides in negative control samples and specific samples were conjugated to distinct isotopic tags (iTRAQ114, PS1 negative control; iTRAQ115, PS1; iTRAQ, PS2 negative control; iTRAQ117, PS2) and processed in an identical manner to the samples described above. In total, this approach led to the identification of 39 proteins that repeatedly passed significance thresholds in biological replicates based on separate purifications (Table 2).

**TABLE 2:**
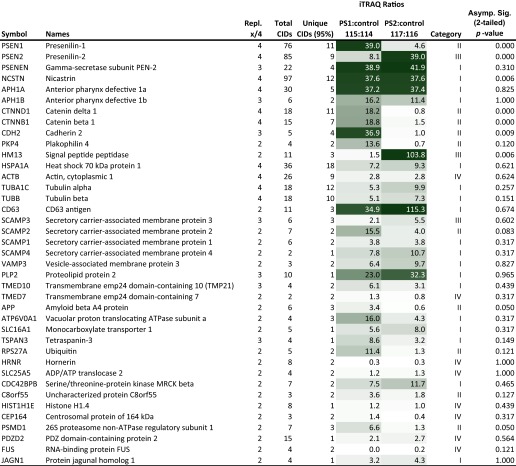
**Summary data table depicting quantitative comparison of TAP-PS1 versus TAP-PS2 interactomes in HEK293 cells**

Based on distinct iTRAQ signature mass peak intensity patterns, identified proteins could be grouped into four different candidate categories (supplemental Fig. S1). (i) PS1/PS2-specific binder category: proteins assigned to this category displayed iTRAQ signature mass peak distributions consistent with the interpretation that they were found in both PS1- and PS2-specific samples but not in the nonspecific control samples. The known γ-secretase core constituents nicastrin, Pen-2, Aph-1A, and Aph-1B were assigned to this category (supplemental Fig. S2), as well as Tmp21, CD63, and a few other proteins. (ii) PS1-specific binder category: proteins in this group are characterized by an iTRAQ signature mass peak pattern in which only the intensity of the iTRAQ 115 signature peak is elevated. PS1 itself (supplemental Fig. 3*A*), β-catenin, δ-catenin, cadherin-2, plakophhilin-4, and a few other proteins were observed to co-enrich in this manner. (iii) PS2-specific binder category: analogous to the previous category except that CID spectra that underlie the identification of proteins in this group only show a relative intensity elevation of the iTRAQ 117 signature peak. Only PS2 itself (supplemental Fig. S3*B*) and SPP could be assigned to this category (supplemental Fig. S4). (iv) Nonspecific binder category: a small subset of proteins appeared to have been nonspecifically captured by the TAP procedure or was introduced during sample handling, intentionally or inadvertently. Proteins in this group were recognizable by an iTRAQ signature mass peak pattern displaying similar intensities for all iTRAQ signature masses. As expected from the silver and Coomassie staining analyses of SBP eluate fractions (Fig. 4, *B* and *C*) very few proteins were in this manner interpreted to be nonspecifically carried through the TAP purification procedure. As such, this category was primarily based on CID spectra that could be assigned to TEV protease, autolysis of trypsin, streptavidin, or keratins (not shown).

The presence of all known constituents of the γ-secretase core complex in the interactome data set, including the small protein Pen-2, which was detected on the basis of 3 CID spectra, served as a positive control in this analysis. Additional proteins identified as candidate PS1- and PS2-interactors that had previously been shown to co-purify with γ-secretase complexes were members of the catenin/cadherin cell adhesion system, TMP21, monocarboxylate transporter 1 (MCT1), and proteolipid protein 2. Altogether, 18 proteins co-purified with both PS paralogs, 10 proteins selectively co-enriched with PS1, and only two proteins showed preferential association with PS2.

##### Association of SPP with PS2

**FIGURE 5.**
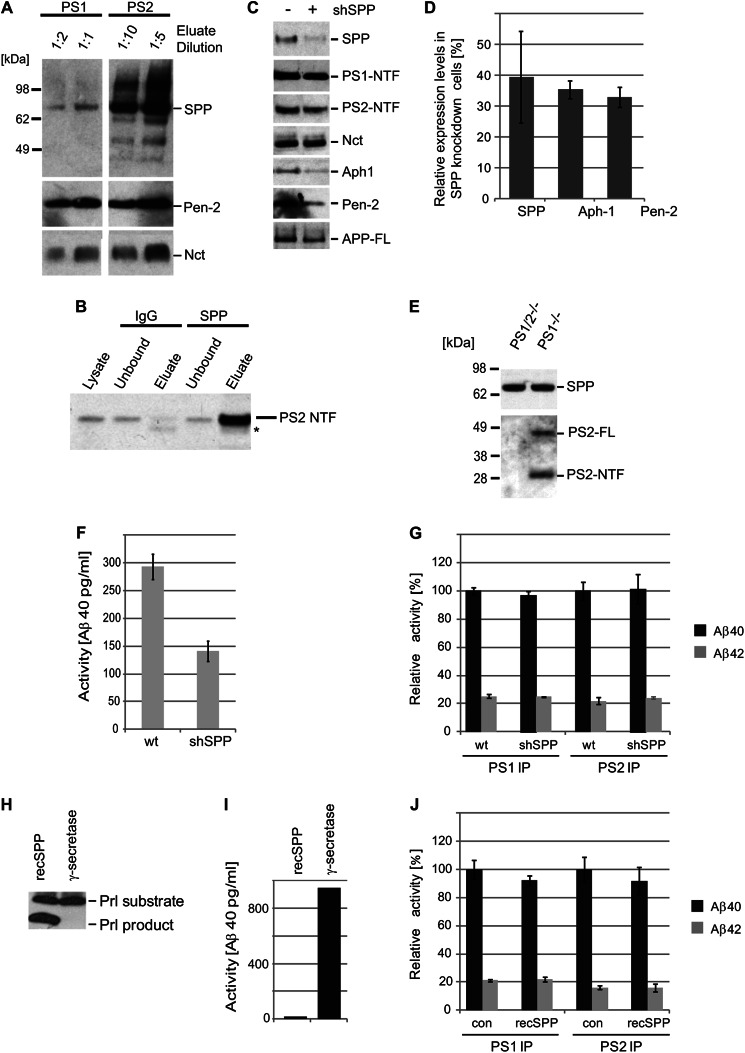
**SPP affects Aβ release by modulating Aph-1 and Pen-2 levels.** *A*, validation of preferential co-enrichment of SPP together with PS2-containing γ-secretase complexes. SBP eluate fractions from multistep purifications of PS1- and PS2-containing γ-secretase complexes were subjected to Western blot analyses. Two different dilutions of each eluate fraction were analyzed side by side to identify a loading quantity that would give rise to near identical signals derived from the presence of Pen-2 and Nct in the samples. *B*, reciprocal co-immunoprecipitation analysis of endogenous SPP in wild-type HEK293 confirms co-enrichment of PS2. Signals labeled with an *asterisk* most likely represent a band derived from the light chain of the polyclonal antibody employed for the immunoprecipitation of SPP. *C,* multipanel Western blot data documenting shRNA-mediated knockdown of SPP in HEK293 cells stably expressing the Swedish variant of APP. *D,* densitometric quantitation of Western blot signals detected in biological replicates (*n* = 3) of SPP knockdown experiment shown in *panel D. E*, expression levels of SPP are not affected by the presence or absence of PS2. Mouse embryonic fibroblasts derived from double PS1/PS2 or single PS1 knock-out embryos show consistent expression of SPP. Equal amounts of cellular extracts were subjected to Western blot analyses and probed with antibodies directed against SPP or the N-terminal fragment of PS2. *F*, significant reduction of γ-secretase activity in HEK293 cells that had been subjected to shRNA-mediated SPP knockdown. Measurements of γ-secretase activity were based on APP-C100 substrate assay that determines the quantities of secreted Aβ40 by ELISA. *G,* naive HEK293 cells or HEK293 cells stably expressing an SPP shRNA (*shSPP*) were lysed and endogenous PS1- or PS2-containing γ-secretase complexes were co-immunoprecipitated with paralog-specific antibodies. Precipitates were incubated at 37 °C with reaction buffer and exogenously generated APP-C100 γ-secretase substrate. A β levels produced in three independent experiments were measured by ELISA and expressed as mean ± S.D. *H, in vitro* prolactin substrate cleavage analysis documenting that recombinant SPP is active toward this substrate. A preparation of TAP purified PS1-containing γ-secretase complexes served as a negative control in this experiment. *I,* recombinant SPP does not cleave the APP-C100 substrate *in vitro*. TAP purified PS1-containing γ-secretase complexes served as a positive control. *J,* recombinant SPP was added to immunopurified PS1- or PS2-containing γ-secretase complexes and the *in vitro* APP-C100 substrate cleavage activity was measured by ELISA.

The consistent distribution of iTRAQ signature mass peaks observed in SPP-specific CID spectra (supplemental Table S3 and Fig. S4) suggested that this candidate interactor was co-purifying predominantly with PS2 (*p* value: 0.006), *i.e.*, whereas the ratio of the 117:116 iTRAQ signature mass peaks indicating the relative enrichment of SPP in PS2-specific *versus* negative control samples averaged a value of 103.8, the corresponding ratio of 115:114 iTRAQ signature mass peaks for PS1 averaged a value of 1.5 (Table 2). To validate the preferential co-enrichment of SPP with PS2-containing complexes, we next analyzed purified PS1- and PS2-containing γ-secretase complexes by Western blotting using an SPP-directed antibody for its detection. When levels of PS1- and PS2-containing mature γ-secretase complexes were normalized based on band intensities of their shared Pen-2 and nicastrin subunits, the amount of SPP that was co-enriched with PS2 complexes strongly exceeded SPP levels detected in PS1 complex samples (Fig. 5*A*), consistent with the preferential enrichment of SPP with PS2 that iTRAQ mass peak patterns within the CID spectra assigned to SPP had indicated. To assess whether co-purification of SPP merely represented an artifact of the heterologous expression of the bait proteins in lentivirally transduced HEK293 integrant clones, reciprocal co-immunoprecipitation experiments were next conducted with naive HEK293 cells that express exclusively endogenous PS paralogs. Western blot analyses of SPP-specific and negative control co-immunoprecipitations based on a nonspecific immunoglobulin established that SPP-capture leads to the specific co-enrichment of endoproteolytically processed N-terminal fragments of PS2 (Fig. 5*B*).

##### SPP Affects Aβ Release by Modulating Aph-1 and Pen-2 Levels

Having established the authenticity of the interaction between PS2 and SPP a subsequent series of experiments examined whether SPP and presenilins influence levels of expression or post-translational modifications of each other. To this end, SPP levels were suppressed by shRNA-based knockdown in HEK293 cells stably expressing the Alzheimer disease Swedish variant of APP. Next, equal levels of total protein from cell extracts were analyzed by Western blotting and it was determined by densitometry that levels of SPP expression were silenced in shRNA-treated cells to less than 40% signal intensities observed in naive cells (Fig. 5*C*). When the same fractions were probed with antibodies specific for γ-secretase core constituents no significant changes in PS1, PS2, or nicastrin expression levels were observed. However, the SPP knockdown caused a considerable decrease in the expression levels of Aph-1 and Pen-2 that approximately matched the level of signal reduction seen for SPP itself (Fig. 5, *C* and *D*). A reciprocal analysis of SPP levels in cell extracts derived from mouse embryonic fibroblast cell clones that express only PS2 or were entirely devoid of presenilins (PS1/PS2 double knock-out cells) similarly exhibited no difference in SPP protein expression levels, corroborating the view that SPP and presenilins do not influence the expression of each other (Fig. 5*E*).

We next examined whether SPP is involved in the production and/or release of cellular Aβ. The experiment was based on a sandwich ELISA and relative γ-secretase activities in cellular extracts were compared *in vitro* by monitoring Aβ40 release following intramembrane endoproteolysis of the APP-C100 substrate. A comparison of HEK293 cells that had been stably transfected with SPP-specific or mock shRNAs revealed a significant reduction in secreted Aβ40 peptide levels from cellular extracts that exhibited diminished SPP levels (Fig. 5*F*).

Finally, we assessed if the reduction in secreted Aβ levels in SPP knockdown cells is linked to the lower Aph-1 or Pen-2 levels we observed or were based on a more direct effect of SPP on γ-secretase activity. To this end, the APP-C100 cleavage activities of immunoprecipitated PS2-containing γ-secretase complexes harvested from naive or SPP knock-out HEK293 cells were measured side by side (Fig. 5*G*). Alternatively, recombinant SPP, which could cleave a prolactin-derived peptide (Fig. 5*H*) but did not process the APP-C100 peptide by itself (Fig. 5*I*), was added directly to immunoprecipitated PS2-containing γ-secretase preparations to determine whether it can modulate γ-secretase cleavage of APP-C100 (Fig. 5*J*). Both approaches relied on the detection of Aβ40/42 levels by sandwich ELISA and revealed no direct effect of SPP levels on the ability of PS2-containing γ-secretase complexes to generate Aβ40/42 levels *in vitro*. Taken together, these experiments support a model whereby SPP, although preferentially interacting with PS2-containing γ-secretase complexes, may influence Aβ production not through its association with PS2 but by modulating cellular levels of Aph-1 and Pen-2.

## DISCUSSION

This study was designed to shed light on the molecular environment of distinct γ-secretase complexes. Through a series of comparative and quantitative interactome analyses it (i) confirmed the heterotetrameric composition of mature γ-secretase core complexes; (ii) confirmed several previously proposed γ-secretase interactors (*e.g.* catenins and proteolipid protein); (iii) uncovered novel interactions of mature γ-secretase with a molecular machinery that targets and fuses synaptic vesicles to cellular membranes and the H^+^-transporting lysosomal ATPase macrocomplex; (iv) provided examples of proteins that can engage in robust interactions with γ-secretase complexes in individual cell types but may escape detection when whole brain interactome studies are conducted (*e.g.* CD63, Tmp21, SCAMPs, heat shock protein 70); (v) revealed mature γ-secretase complexes containing wild-type or mutant PS1 to be indistinguishable in their protein composition; (vi) firmly established a predominant association of the catenin-cadherin network with PS1-containing γ-secretase complexes; and (vi) uncovered a preferential enrichment of SPP with PS2-containing γ-secretase complexes.

### 

#### 

##### Specific Versus Nonspecific Interactors

The scale of the interactome analyses presented in this study was comparable with two previous reports (19, 43). To our knowledge, it was the first to identify by mass spectrometry all known constituents of the γ-secretase core complex, including Pen-2 and Aph-1b. The strong coverage of known γ-secretase core constituents may have been facilitated by the inclusion of the WGA lectin affinity capture step in the sample work-up procedures (Figs. 1 and 3) that restricted the interactome analyses to fully assembled γ-secretase complexes. This interpretation is supported by the conspicuous absence in interactome tables we presented of a group of previously reported candidate interactors that includes calnexin, protein-disulfide isomerase, calreticulin, and BIP, proteins that, on the basis of their predominant localization in ER and Golgi compartments, may primarily engage in contacts with immature PS during its passage through the secretory pathway.

A previous large-scale γ-secretase interactome study could draw qualitative conclusions about PS1 and PS2 interactomes being similar, but the consecutive analysis of PS1 and PS2 interactomes undertaken in that work precluded a confident assessment of differences in the interactomes of the PS paralogs (19). The concomitant analyses of control and bait-specific affinity capture eluates afforded by the iTRAQ labels were instrumental to overcome run to run variance and enable relative comparisons. These comparisons helped to flag several proteins (*e.g.* Na/K-ATPase subunits and valosin-containing protein) that had previously been proposed to represent *bona fide* candidate interactors (19) as probably belonging to the nonspecific binder category.

Multiple components of the lysosomal V-ATPase complex (but not its mitochondrial cousin) co-purified selectively with PS complexes in this work, corroborating data from a recent report that had tied PS1 to lysosomal targeting of the V-ATPase V0a1 subunit (44). The authors demonstrated that in the absence of immature full-length PS1 the V0a1 subunit failed to be *N*-glycosylated, a step required for its efficient ER to lysosome delivery. Data presented in this work suggest that an interaction with lysosomal V-ATPase may not be limited to PS1 but may extend to PS2. The interaction may in certain paradigms also not be restricted to immature full-length PS because the sample work-up scheme employed in this work restricted capture to fully mature γ-secretase complexes containing *N*-glycosylated nicastrin and endoproteolytically cleaved PS.

##### Distinct Molecular Environments of PS Paralogs

Data presented in this work confirmed the anticipation that a majority of presenilin candidate interactors associate with both PS1- and PS2-containing γ-secretase complexes but also revealed a small number of proteins that co-purified in a PS paralog-specific manner. In the latter category belong members of the catenin/cadherin family (catenin δ-1, catenin β-1, plakophilin-4, and cadherin-2) that exhibited a strong bias for co-enrichment with PS1. This conclusion was unequivocally supported by the presence of more than three dozen CID spectra, which could be assigned to peptides derived from these proteins and displayed strong signals for the PS1-pecific iTRAQ115 signature mass peak and only weak signals for the PS2-specific iTRAQ117 signature mass peak (supplemental Table S3). Cadherins are transmembrane proteins involved in a calcium-dependent cell adhesion biology and the transduction of signals into the cell, a biology that is at least partly mediated by their interaction with catenins (15). These findings extend previous data that revealed a direct interaction between PS1 and a subset of catenins (p120 δ-catenin) and an enzyme-substrate relationship of γ-secretase toward a subset of cadherins (for example, N- and E-cadherins) (14–16, 45). It has been proposed that the large cytoplasmic loop within the C-terminal endoproteolytic fragment of PS1 is required for the direct association of PS1 with δ-catenin (15). Our observation that the catenin/cadherin network does not interact with PS2 is not only consistent with these earlier data but could have been predicted considering that this paralog exhibits very little sequence homology with PS1 in the large cytoplasmic loop domain.

Previous reports attributed APP intramembrane cleavages *in vivo* foremost to γ-secretase complexes containing PS1 (46, 47). This conclusion was drawn from studies on PS1 knock-out cells that exhibited near complete abolishment of APP-directed γ-secretase activity and PS2 knock-out cells that resulted in only minor reductions in γ-secretase activity (27). Although PS1 is known to be expressed at higher levels than PS2 during development (48) the aforementioned observations did not appear to merely reflect differences in the levels of expression (49). These cellular data stood in contrast to biochemical comparisons of purified γ-secretase complexes that reported similar turnover rates of PS1- or PS2-containing preparations for the APP substrate (50). Data in this work were consistent with data from the latter study but also documented that differences in APP substrate processing emerged when PS1- and PS2-containing γ-secretase complexes were compared with regard to their response to treatments with two well characterized γ-secretase inhibitors and inhibitor dose-response curves were recorded.

##### “Proteasome-of-the-membrane” Concept

Surprisingly, SPP predominantly co-purified with PS2 in this work. The physiological role of SPP has been linked to the processing of signal peptides in the context of ER quality control activities (51). SPP and presenilins exhibit almost no sequence homology but share active site signature motifs “G*X*GD” and “YD” found in juxtaposed transmembrane domains, with aspartate residues within this motif contributing to the respective catalytic centers of these intramembrane proteases. Both proteins are further equipped with a “PAL” sequence motif presumed to play a role in stabilizing the active site (52). Relative to presenilins, SPP appears to be inserted into cellular membranes with an inverted membrane topology (31), and whereas presenilins process substrate proteins that acquire a Type 1 transmembrane topology, SPP has been shown to cleave, in accordance with the orientation of its catalytic center, substrate proteins with a Type II transmembrane topology.

##### What Might Be the Physiological Significance of the SPP-PS2 Interaction?

When SPP levels were experimentally reduced in this study in the HEK293 cell model by the use of SPP-specific shRNAs, cellular extracts exhibited lower γ-secretase activity in a conventional assay that measures the release of Aβ peptides. It is currently unclear whether this observation is the result of SPP affecting the maturation of γ-secretase, access of the active complex to its APP substrate, or of SPP influencing the γ-secretase-mediated intramembrane proteolysis step more directly. The reduction in Aph1 and Pen2 protein levels we observed is, however, suggestive of SPP mediating this effect on Aβ release, at least in part, by influencing steady-state levels of mature γ-secretase.

It is likely that the broader significance of the interaction between PS2 and SPP will emerge once a complex biology surrounding the intramembrane cleavage of their transmembrane substrates is better understood. Although for a subset of presenilin substrates a physiological role of γ-secretase cleavage products could be identified (for example, for the notch intracellular domain), for other substrates this has not been straightforward. The broad spectrum of γ-secretase substrates has invoked the analogy of γ-secretase as a “secretosome” or “proteasome of the membrane.” According to this model γ-secretase may patrol cellular membranes to rid them of the cumulative burden posed by Type-I transmembrane stubs left behind from ongoing cellular sheddase activities (53–55). The surprising association of PS2 and SPP revealed in this work adds fuel to this concept. Just as the proteasome is equipped with multiple proteolytic activities to be able to deal with a diverse range of substrates, the cell may have devised an analogous machine for the removal of transmembrane stubs by pairing intramembrane proteases that exhibit proteolytic activity against transmembrane domains embedded in the lipid bilayer by a range of topologies.

## Supplementary Material

Supplemental Data

## Footnotes

5 The abbreviations used are:

AD: Alzheimer disease
Aβ: amyloid β-peptide
APP: amyloid precursor protein
CHAPSO: 3-[(3-cholamidopropyl)dimethylammonio]-1-propanesulfonate
CID: collision-induced dissociation
DDM: *n*-dodecyl β-d-maltoside
iTRAQ: isotopic tags for relative and absolute quantitation
MS/MS: tandem mass spectrometry
PS: presenilins
SPP: signal peptide peptidase
TAP: tandem affinity purification
WGA: wheat germ agglutinin
TEV: tobacco etch virus
BisTris: 2-[bis(2-hydroxyethyl)amino]-2-(hydroxymethyl)propane-1,3-diol
SBP: streptavidin-binding peptide
SPP: signal peptide peptidase
ER: endoplasmic reticulum.

## References

[1] Sato T. Diehl T. S. Narayanan S. Funamoto S. Ihara Y. De Strooper B. Steiner H. Haass C. Wolfe M. S. (2007) Active γ-secretase complexes contain only one of each component. J. Biol. Chem. 282, 33985–33993 17911105 10.1074/jbc.M705248200

[2] Lazarov V. K. Fraering P. C. Ye W. Wolfe M. S. Selkoe D. J. Li H. (2006) Electron microscopic structure of purified, active γ-secretase reveals an aqueous intramembrane chamber and two pores. Proc. Natl. Acad. Sci. U.S.A. 103, 6889–6894 16636269 10.1073/pnas.0602321103 PMC1458989

[3] Laudon H. Hansson E. M. Melén K. Bergman A. Farmery M. R. Winblad B. Lendahl U. von Heijne G. Näslund J. (2005) A nine-transmembrane domain topology for presenilin 1. J. Biol. Chem. 280, 35352–35360 16046406 10.1074/jbc.M507217200

[4] Spasic D. Tolia A. Dillen K. Baert V. De Strooper B. Vrijens S. Annaert W. (2006) Presenilin-1 maintains a nine-transmembrane topology throughout the secretory pathway. J. Biol. Chem. 281, 26569–26577 16846981 10.1074/jbc.M600592200

[5] Wolfe M. S. Xia W. Ostaszewski B. L. Diehl T. S. Kimberly W. T. Selkoe D. J. (1999) Two transmembrane aspartates in presenilin-1 required for presenilin endoproteolysis and γ-secretase activity. Nature 398, 513–517 10206644 10.1038/19077

[6] Edbauer D. Winkler E. Regula J. T. Pesold B. Steiner H. Haass C. (2003) Reconstitution of γ-secretase activity. Nat. Cell Biol. 5, 486–488 12679784 10.1038/ncb960

[7] Wolfe M. S. (2007) When loss is gain. Reduced presenilin proteolytic function leads to increased Abeta42/Abeta40. Talking Point on the role of presenilin mutations in Alzheimer disease. EMBO Rep. 8, 136–140 17268504 10.1038/sj.embor.7400896 PMC1796780

[8] Wolfe M. S. De Los Angeles J. Miller D. D. Xia W. Selkoe D. J. (1999) Are presenilins intramembrane-cleaving proteases? Implications for the molecular mechanism of Alzheimer's disease. Biochemistry 38, 11223–11230 10471271 10.1021/bi991080q

[9] Spasic D. Annaert W. (2008) Building γ-secretase. The bits and pieces. J. Cell Sci. 121, 413–420 18256384 10.1242/jcs.015255

[10] Chen F. Hasegawa H. Schmitt-Ulms G. Kawarai T. Bohm C. Katayama T. Gu Y. Sanjo N. Glista M. Rogaeva E. Wakutani Y. Pardossi-Piquard R. Ruan X. Tandon A. Checler F. Marambaud P. Hansen K. Westaway D. St George-Hyslop P. Fraser P. (2006) TMP21 is a presenilin complex component that modulates γ-secretase but not ϵ-secretase activity. Nature 440, 1208–1212 16641999 10.1038/nature04667

[11] Vetrivel K. S. Zhang X. Meckler X. Cheng H. Lee S. Gong P. Lopes K. O. Chen Y. Iwata N. Yin K. J. Lee J. M. Parent A. T. Saido T. C. Li Y. M. Sisodia S. S. Thinakaran G. (2008) Evidence that CD147 modulation of β-amyloid (Aβ) levels is mediated by extracellular degradation of secreted Aβ. J. Biol. Chem. 283, 19489–19498 18456655 10.1074/jbc.M801037200 PMC2443668

[12] Zhou S. Zhou H. Walian P. J. Jap B. K. (2005) CD147 is a regulatory subunit of the γ-secretase complex in Alzheimer's disease amyloid β-peptide production. Proc. Natl. Acad. Sci. U.S.A. 102, 7499–7504 15890777 10.1073/pnas.0502768102 PMC1103709

[13] Stahl B. Diehlmann A. Südhof T. C. (1999) Direct interaction of Alzheimer's disease-related presenilin 1 with armadillo protein p0071. J. Biol. Chem. 274, 9141–9148 10092585 10.1074/jbc.274.14.9141

[14] Serban G. Kouchi Z. Baki L. Georgakopoulos A. Litterst C. M. Shioi J. Robakis N. K. (2005) Cadherins mediate both the association between PS1 and β-catenin and the effects of PS1 on β-catenin stability. J. Biol. Chem. 280, 36007–36012 16126725 10.1074/jbc.M507503200 PMC4005066

[15] Kouchi Z. Barthet G. Serban G. Georgakopoulos A. Shioi J. Robakis N. K. (2009) p120 catenin recruits cadherins to γ-secretase and inhibits production of Abeta peptide. J. Biol. Chem. 284, 1954–1961 19008223 10.1074/jbc.M806250200 PMC2629097

[16] Georgakopoulos A. Marambaud P. Efthimiopoulos S. Shioi J. Cui W. Li H. C. Schütte M. Gordon R. Holstein G. R. Martinelli G. Mehta P. Friedrich V. L. Jr. Robakis N. K. (1999) Presenilin-1 forms complexes with the cadherin/catenin cell-cell adhesion system and is recruited to intercellular and synaptic contacts. Mol. Cell 4, 893–902 10635315 10.1016/s1097-2765(00)80219-1

[17] Baki L. Marambaud P. Efthimiopoulos S. Georgakopoulos A. Wen P. Cui W. Shioi J. Koo E. Ozawa M. Friedrich V. L. Jr. Robakis N. K. (2001) Presenilin-1 binds cytoplasmic epithelial cadherin, inhibits cadherin/p120 association, and regulates stability and function of the cadherin/catenin adhesion complex. Proc. Natl. Acad. Sci. U.S.A. 98, 2381–2386 11226248 10.1073/pnas.041603398 PMC30147

[18] He G. Luo W. Li P. Remmers C. Netzer W. J. Hendrick J. Bettayeb K. Flajolet M. Gorelick F. Wennogle L. P. Greengard P. (2010) γ-Secretase activating protein is a therapeutic target for Alzheimer's disease. Nature 467, 95–98 20811458 10.1038/nature09325 PMC2936959

[19] Wakabayashi T. Craessaerts K. Bammens L. Bentahir M. Borgions F. Herdewijn P. Staes A. Timmerman E. Vandekerckhove J. Rubinstein E. Boucheix C. Gevaert K. De Strooper B. (2009) Analysis of the γ-secretase interactome and validation of its association with tetraspanin-enriched microdomains. Nat. Cell Biol. 11, 1340–1346 19838174 10.1038/ncb1978

[20] Rogaev E. I. Sherrington R. Rogaeva E. A. Levesque G. Ikeda M. Liang Y. Chi H. Lin C. Holman K. Tsuda T. (1995) Familial Alzheimer's disease in kindreds with missense mutations in a gene on chromosome 1 related to the Alzheimer's disease type 3 gene. Nature 376, 775–778 7651536 10.1038/376775a0

[21] Sherrington R. Rogaev E. I. Liang Y. Rogaeva E. A. Levesque G. Ikeda M. Chi H. Lin C. Li G. Holman K. Tsuda T. Mar L. Foncin J. F. Bruni A. C. Montesi M.P. Sorbi S. Rainero I. Pinessi L. Nee L. Chumakov I. Pollen D. Brookes A. Sanseau P. Polinsky R. J. Wasco W. Da Silva H. A. Haines J. L. Perkicak-Vance M. A. Tanzi R. E. Roses A. D. Fraser P. E. Rommens J. M. St George-Hyslop P. H. (1995) Cloning of a gene bearing missense mutations in early-onset familial Alzheimer's disease. Nature 375, 754–760 7596406 10.1038/375754a0

[22] Levy-Lahad E. Wijsman E. M. Nemens E. Anderson L. Goddard K. A. Weber J. L. Bird T. D. Schellenberg G. D. (1995) A familial Alzheimer's disease locus on chromosome 1. Science 269, 970–973 7638621 10.1126/science.7638621

[23] Mastrangelo P. Mathews P. M. Chishti M. A. Schmidt S. D. Gu Y. Yang J. Mazzella M. J. Coomaraswamy J. Horne P. Strome B. Pelly H. Levesque G. Ebeling C. Jiang Y. Nixon R. A. Rozmahel R. Fraser P. E. St George-Hyslop P. Carlson G. A. Westaway D. (2005) Dissociated phenotypes in presenilin transgenic mice define functionally distinct γ-secretases. Proc. Natl. Acad. Sci. U.S.A. 102, 8972–8977 15951428 10.1073/pnas.0500940102 PMC1149500

[24] Hutton M. Hardy J. (1997) The presenilins and Alzheimer's disease. Hum. Mol. Genet. 6, 1639–1646 9300655 10.1093/hmg/6.10.1639

[25] Donoviel D. B. Hadjantonakis A. K. Ikeda M. Zheng H. Hyslop P. S. Bernstein A. (1999) Mice lacking both presenilin genes exhibit early embryonic patterning defects. Genes Dev. 13, 2801–2810 10557208 10.1101/gad.13.21.2801 PMC317124

[26] Herreman A. Hartmann D. Annaert W. Saftig P. Craessaerts K. Serneels L. Umans L. Schrijvers V. Checler F. Vanderstichele H. Baekelandt V. Dressel R. Cupers P. Huylebroeck D. Zwijsen A. Van Leuven F. De Strooper B. (1999) Presenilin 2 deficiency causes a mild pulmonary phenotype and no changes in amyloid precursor protein processing but enhances the embryonic lethal phenotype of presenilin 1 deficiency. Proc. Natl. Acad. Sci. U.S.A. 96, 11872–11877 10518543 10.1073/pnas.96.21.11872 PMC18379

[27] Martoglio B. Golde T. E. (2003) Intramembrane-cleaving aspartic proteases and disease. Presenilins, signal peptide peptidase and their homologs. Hum. Mol. Genet. 12, R201–206 12966028 10.1093/hmg/ddg303

[28] Frånberg J. Svensson A. I. Winblad B. Karlström H. Frykman S. (2011) Minor contribution of presenilin 2 for γ-secretase activity in mouse embryonic fibroblasts and adult mouse brain. Biochem. Biophys. Res. Commun. 404, 564–568 21146496 10.1016/j.bbrc.2010.12.029

[29] Kang D. E. Yoon I. S. Repetto E. Busse T. Yermian N. Ie L. Koo E. H. (2005) Presenilins mediate phosphatidylinositol 3-kinase/AKT and ERK activation via select signaling receptors. Selectivity of PS2 in platelet-derived growth factor signaling. J. Biol. Chem. 280, 31537–31547 16014629 10.1074/jbc.M500833200

[30] Janus C. D'Amelio S. Amitay O. Chishti M. A. Strome R. Fraser P. Carlson G. A. Roder J. C. St George-Hyslop P. Westaway D. (2000) Spatial learning in transgenic mice expressing human presenilin 1 (PS1) transgenes. Neurobiol. Aging 21, 541–549 10924767 10.1016/s0197-4580(00)00107-x

[31] Weihofen A. Binns K. Lemberg M. K. Ashman K. Martoglio B. (2002) Identification of signal peptide peptidase, a presenilin-type aspartic protease. Science 296, 2215–2218 12077416 10.1126/science.1070925

[32] Bürckstümmer T. Bennett K. L. Preradovic A. Schütze G. Hantschel O. Superti-Furga G. Bauch A. (2006) An efficient tandem affinity purification procedure for interaction proteomics in mammalian cells. Nat. Methods 3, 1013–1019 17060908 10.1038/nmeth968

[33] Watts J. C. Huo H. Bai Y. Ehsani S. Jeon A. H. Won A. H. Shi T. Daude N. Lau A. Young R. Xu L. Carlson G. A. Williams D. Westaway D. Schmitt-Ulms G. (2009) Interactome analyses identify ties of PrP and its mammalian paralogs to oligomannosidic *N*-glycans and endoplasmic reticulum-derived chaperones. PLoS Pathog. 5, e1000608 19798432 10.1371/journal.ppat.1000608 PMC2749441

[34] Arawaka S. Hasegawa H. Tandon A. Janus C. Chen F. Yu G. Kikuchi K. Koyama S. Kato T. Fraser P. E. St George-Hyslop P. (2002) The levels of mature glycosylated nicastrin are regulated and correlate with γ-secretase processing of amyloid β-precursor protein. J. Neurochem. 83, 1065–1071 12437577 10.1046/j.1471-4159.2002.01207.x

[35] Kimberly W. T. LaVoie M. J. Ostaszewski B. L. Ye W. Wolfe M. S. Selkoe D. J. (2002) Complex *N*-linked glycosylated nicastrin associates with active γ-secretase and undergoes tight cellular regulation. J. Biol. Chem. 277, 35113–35117 12130643 10.1074/jbc.M204446200

[36] Yang D. S. Tandon A. Chen F. Yu G. Yu H. Arawaka S. Hasegawa H. Duthie M. Schmidt S. D. Ramabhadran T. V. Nixon R. A. Mathews P. M. Gandy S. E. Mount H. T. St George-Hyslop P. Fraser P. E. (2002) Mature glycosylation and trafficking of nicastrin modulate its binding to presenilins. J. Biol. Chem. 277, 28135–28142 12032140 10.1074/jbc.M110871200

[37] Tomita T. Katayama R. Takikawa R. Iwatsubo T. (2002) Complex *N*-glycosylated form of nicastrin is stabilized and selectively bound to presenilin fragments. FEBS Lett. 520, 117–121 12044882 10.1016/s0014-5793(02)02802-8

[38] Zieske L. R. (2006) A perspective on the use of iTRAQ reagent technology for protein complex and profiling studies. J. Exp. Bot. 57, 1501–1508 16574745 10.1093/jxb/erj168

[39] Bergman A. Hansson E. M. Pursglove S. E. Farmery M. R. Lannfelt L. Lendahl U. Lundkvist J. Näslund J. (2004) Pen-2 is sequestered in the endoplasmic reticulum and subjected to ubiquitylation and proteasome-mediated degradation in the absence of presenilin. J. Biol. Chem. 279, 16744–16753 14724271 10.1074/jbc.M313999200

[40] Trono D. (2000) Lentiviral vectors. Turning a deadly foe into a therapeutic agent. Gene Ther. 7, 20–23 10680011 10.1038/sj.gt.3301105

[41] Shearman M. S. Beher D. Clarke E. E. Lewis H. D. Harrison T. Hunt P. Nadin A. Smith A. L. Stevenson G. Castro J. L. (2000) L-685,458, an aspartyl protease transition state mimic, is a potent inhibitor of amyloid β-protein precursor γ-secretase activity. Biochemistry 39, 8698–8704 10913280 10.1021/bi0005456

[42] Seiffert D. Bradley J. D. Rominger C. M. Rominger D. H. Yang F. Meredith J. E. Jr. Wang Q. Roach A. H. Thompson L. A. Spitz S. M. Higaki J. N. Prakash S. R. Combs A. P. Copeland R. A. Arneric S. P. Hartig P. R. Robertson D. W. Cordell B. Stern A. M. Olson R. E. Zaczek R. (2000) Presenilin-1 and -2 are molecular targets for gamma-secretase inhibitors. J. Biol. Chem. 275, 34086–34091 10915801 10.1074/jbc.M005430200

[43] Winkler E. Hobson S. Fukumori A. Dümpelfeld B. Luebbers T. Baumann K. Haass C. Hopf C. Steiner H. (2009) Purification, pharmacological modulation, and biochemical characterization of interactors of endogenous human γ-secretase. Biochemistry 48, 1183–1197 19159235 10.1021/bi801204g

[44] Lee J. H. Yu W. H. Kumar A. Lee S. Mohan P. S. Peterhoff C. M. Wolfe D. M. Martinez-Vicente M. Massey A. C. Sovak G. Uchiyama Y. Westaway D. Cuervo A. M. Nixon R. A. (2010) Lysosomal proteolysis and autophagy require presenilin 1 and are disrupted by Alzheimer-related PS1 mutations. Cell 141, 1146–1158 20541250 10.1016/j.cell.2010.05.008 PMC3647462

[45] Parisiadou L. Fassa A. Fotinopoulou A. Bethani I. Efthimiopoulos S. (2004) Presenilin 1 and cadherins. Stabilization of cell-cell adhesion and proteolysis-dependent regulation of transcription. Neurodegener. Dis. 1, 184–191 16908988 10.1159/000080984

[46] Ghidoni R. Paterlini A. Benussi L. Binetti G. (2007) Presenilin 2 is secreted in mouse primary neurons. A release enhanced by apoptosis. Mech. Ageing. Dev. 128, 350–353 17292944 10.1016/j.mad.2007.01.003

[47] Lai M. T. Chen E. Crouthamel M. C. DiMuzio-Mower J. Xu M. Huang Q. Price E. Register R. B. Shi X. P. Donoviel D. B. Bernstein A. Hazuda D. Gardell S. J. Li Y. M. (2003) Presenilin-1 and presenilin-2 exhibit distinct yet overlapping γ-secretase activities. J. Biol. Chem. 278, 22475–22481 12684521 10.1074/jbc.M300974200

[48] Lee M. K. Slunt H. H. Martin L. J. Thinakaran G. Kim G. Gandy S. E. Seeger M. Koo E. Price D. L. Sisodia S. S. (1996) Expression of presenilin 1 and 2 (PS1 and PS2) in human and murine tissues. J. Neurosci. 16, 7513–7525 8922407 10.1523/JNEUROSCI.16-23-07513.1996 PMC6579112

[49] Huynh D. P. Vinters H. V. Ho D. H. Ho V. V. Pulst S. M. (1997) Neuronal expression and intracellular localization of presenilins in normal and Alzheimer disease brains. J. Neuropathol. Exp. Neurol. 56, 1009–1017 9291942 10.1097/00005072-199709000-00006

[50] Shirotani K. Tomioka M. Kremmer E. Haass C. Steiner H. (2007) Pathological activity of familial Alzheimer's disease-associated mutant presenilin can be executed by six different γ-secretase complexes. Neurobiol. Dis. 27, 102–107 17560791 10.1016/j.nbd.2007.04.011

[51] Golde T. E. Wolfe M. S. Greenbaum D. C. (2009) Signal peptide peptidases. A family of intramembrane-cleaving proteases that cleave type 2 transmembrane proteins. Semin. Cell Dev. Biol. 20, 225–230 19429495 10.1016/j.semcdb.2009.02.003 PMC6701170

[52] Wang J. Beher D. Nyborg A. C. Shearman M. S. Golde T. E. Goate A. (2006) C-terminal PAL motif of presenilin and presenilin homologues required for normal active site conformation. J. Neurochem. 96, 218–227 16305624 10.1111/j.1471-4159.2005.03548.x

[53] Schenk D. (2000) Alzheimer's disease. A partner for presenilin. Nature 407, 34–35 10993060 10.1038/35024194

[54] Yu C. Kim S. H. Ikeuchi T. Xu H. Gasparini L. Wang R. Sisodia S. S. (2001) Characterization of a presenilin-mediated amyloid precursor protein carboxyl-terminal fragment γ. Evidence for distinct mechanisms involved in γ-secretase processing of the APP and Notch1 transmembrane domains. J. Biol. Chem. 276, 43756–43760 11583985 10.1074/jbc.C100410200

[55] Kopan R. Ilagan M. X. (2004) γ-Secretase. Proteasome of the membrane? Nat. Rev. Mol. Cell Biol. 5, 499–504 15173829 10.1038/nrm1406

