# Supplementary material for: Interactome Analyses of Mature γ-Secretase Complexes Reveal Distinct Molecular Environments of Presenilin (PS) Paralogs and Preferential Binding of Signal Peptide Peptidase to PS2
Source: J Biol Chem. 2013 Apr 15;288(21):15352–66. doi: 10.1074/jbc.M112.441840 (PMC3663554; doi:10.1074/jbc.M112.441840)
Supplement: Supplemental Data [file supp_288_21_15352__index.html]

Interactome analyses of mature γ-secretase complexes reveals distinct molecular environments of presenilin (PS) paralogs and preferential binding of signal peptide peptidase to PS2 — Interactome Analyses of Mature γ-Secretase Complexes Reveal Distinct Molecular Environments of Presenilin (PS) Paralogs and Preferential Binding of Signal Peptide Peptidase to PS2 — Interactomes of Distinct γ-Secretase Complexes — Supplemental Data 

# Interactome Analyses of Mature γ-Secretase Complexes Reveal Distinct Molecular Environments of Presenilin (PS) Paralogs and Preferential Binding of Signal Peptide Peptidase to PS2

## Supplemental Data

**Files in this Data Supplement:**

- Supplemental Figures (.pdf, 234 KB) - Experimental setup for quantitative PS1 and PS2 interactome comparison and representative mass spectrometry data
- Supplemental Table 1 (.pdf, 442 KB) - Quantitative interactome analysis of human wild-type PS1 in transgenic mice
- Supplemental Table 2 (.pdf, 50 KB) - Quantitative comparative interactome analysis of human wild-type versus mutant PS1
- Supplemental Table 3 (.pdf, 85 KB) - Quantitative comparison of TAP-PS1 versus TAP-PS2 interactomes in HEK293 cells
