# Supplementary material for: Interactome Analyses of Mature γ-Secretase Complexes Reveal Distinct Molecular Environments of Presenilin (PS) Paralogs and Preferential Binding of Signal Peptide Peptidase to PS2
Source: J Biol Chem. 2013 Apr 15;288(21):15352–66. doi: 10.1074/jbc.M112.441840 (PMC3663554; doi:10.1074/jbc.M112.441840)

SUPPLEMENTAL FIGURES

Supplemental Figure 1. Sample processing of TAP-purified complexes and experimental setup for quantitative PS1 and PS2 interactome comparisons. (A) Flow-chart depicting steps in preparation of quantitative mass spectrometry analyses, including assignments of iTRAQ conjugation reagents to samples compared. Once labeled samples were combined and subjected to offline strong cation exchange (SCX) and online reverse-phase (RP) liquid chromatography coupled to electrospray (ESI) tandem mass spectrometry (MS/MS). (B) Cartoon depicting iTRAQ signature mass peak intensity patterns (observed in the low mass region of collision-induced dissociation spectra) according to which proteins can be grouped into four categories: (i) PS1/PS2-specific binder category; (ii) PS1-specific binder category; (iii) PS2-specific binder category; (iv) Non-specific binder category.

Supplemental Figure 2. Representative mass spectra demonstrating enrichment of Aph-1A and nicastrin with both PS1- and PS2-containing γ-secretase complexes but not with pre-saturated control matrices. (A) CID spectrum assigned to tryptic peptide 'ADEGLASLSEDGR' of Aph-1A with insert depicting iTRAQ signature mass peak region at higher resolution. (B) CID spectrum assigned to 'SGAGVPAVILR' tryptic peptide derived from nicastrin. Insets: high resolution graphs depicting isotopic envelopes of precursor ions and iTRAQ signature mass peak regions within CID spectra.

Supplemental Figure 3. Representative mass spectra demonstrating selective enrichment of PS1 or PS2 in individual γ-secretase complexes. (A) CID spectrum assigned to tryptic PS1 peptide with the sequence 'QVVEQDEEEDEELTLK'. (B) CID spectrum assigned to tryptic peptide 'QGEPEDGENTAQWR' derived from PS2. Insets: high resolution graphs depicting isotopic envelopes of precursor ions and iTRAQ signature mass peak regions within CID spectra. Note that the distribution of iTRAQ signature ion mass peaks not only documents a selective enrichment of PS paralogs as per the design of the experiment but also argues against the existence of γ-secretase complexes with mixed PS1 and PS2 content.

Supplemental Figure 4. Selective enrichment of SPP with PS2-containing γ-secretase complexes. CID spectrum assigned to tryptic peptide 'NASDMPETITSR' derived from SPP documenting selective enrichment of this protein in PS2-specific eluate fractions labelled with iTRAQ117reagent. Insets: high resolution graphs depicting isotopic envelopes of precursor ions and iTRAQ signature mass peak regions within CID spectra.

Suppl. Figure 1

A

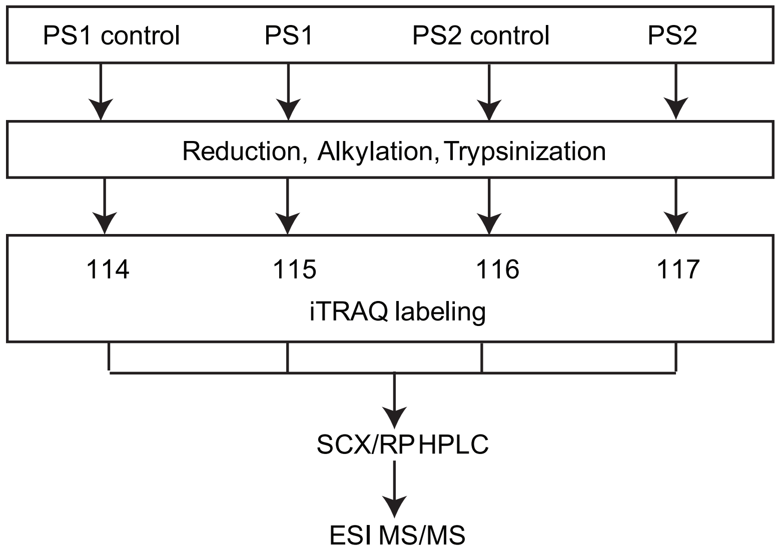

B

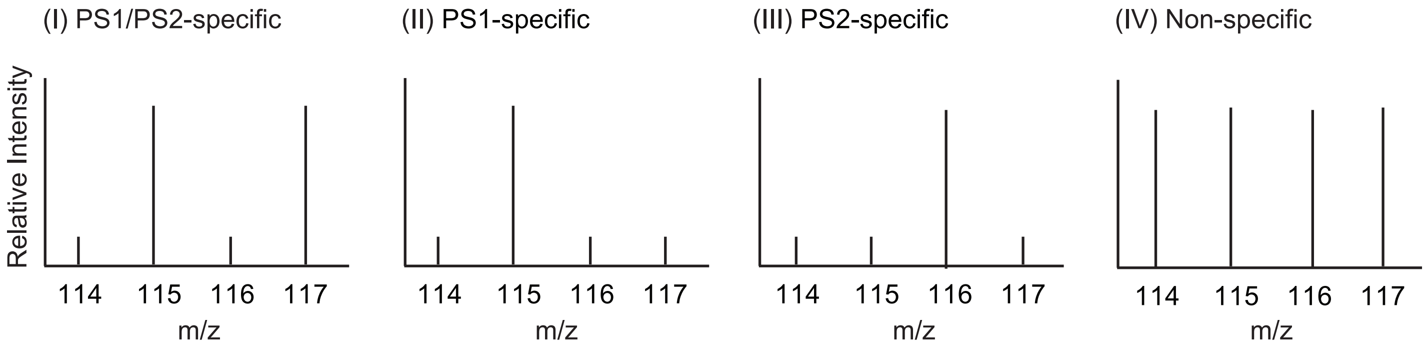

Suppl. Figure 2

A Aph1A (ENSP00000358105), MS/MS of m/z 732.35

ADEGLASLSEDGR (aa 97-109) + iTRAQ114 (N-term)

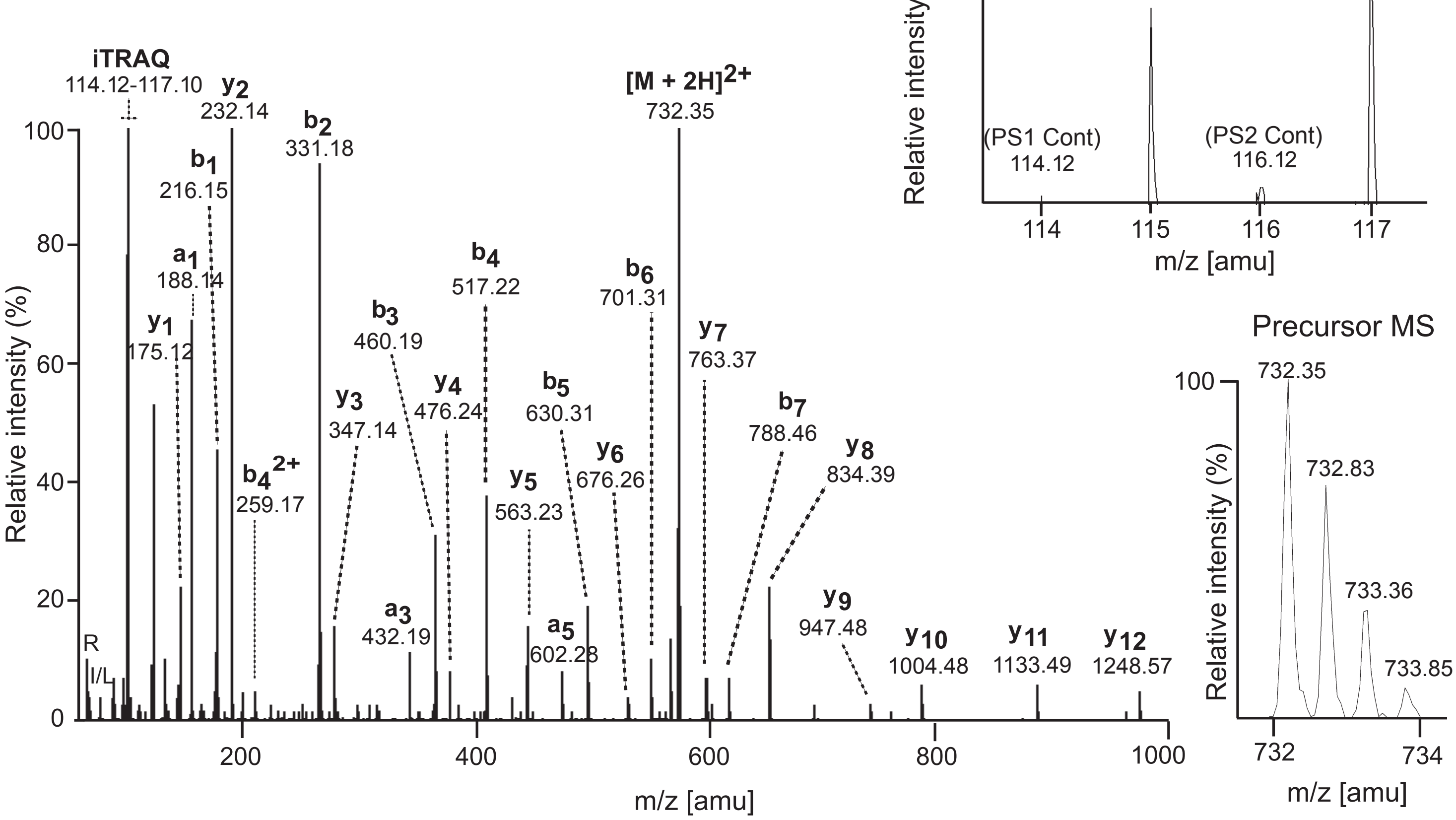

B Nicastrin (ENSP00000357042), MS/MS of m/z 592.39

SGAGVPAVILR (aa 404-414) + iTRAQ114 (N-term)

MS/MS Quantitation Region

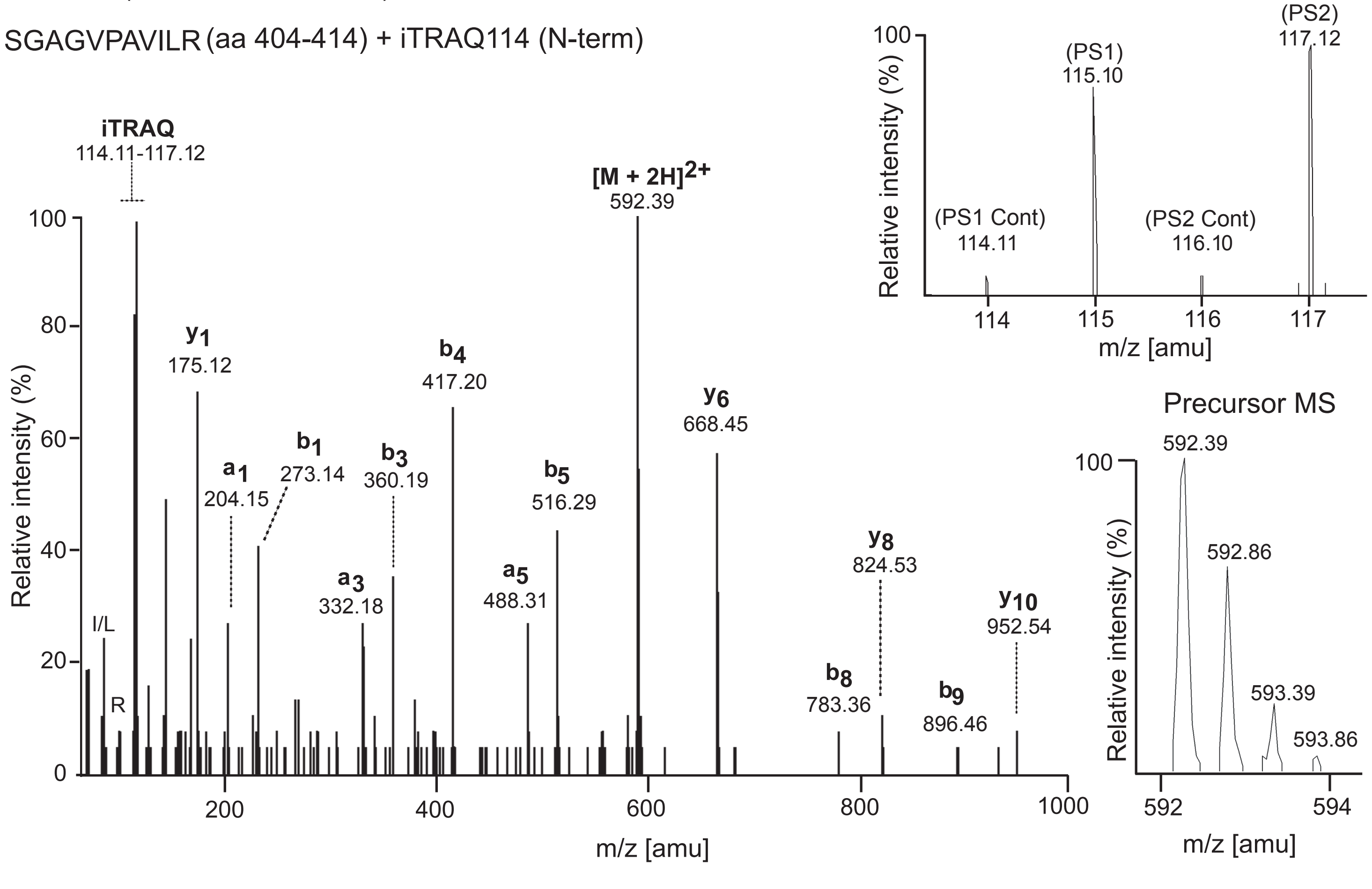

Suppl. Figure 3

A Presenilin 1(ENSP00000326366), MS/MS of m/z 741.00

QVVEQDEEEDEELTLK (aa 61-76) + iTRAQ114 (N-term)

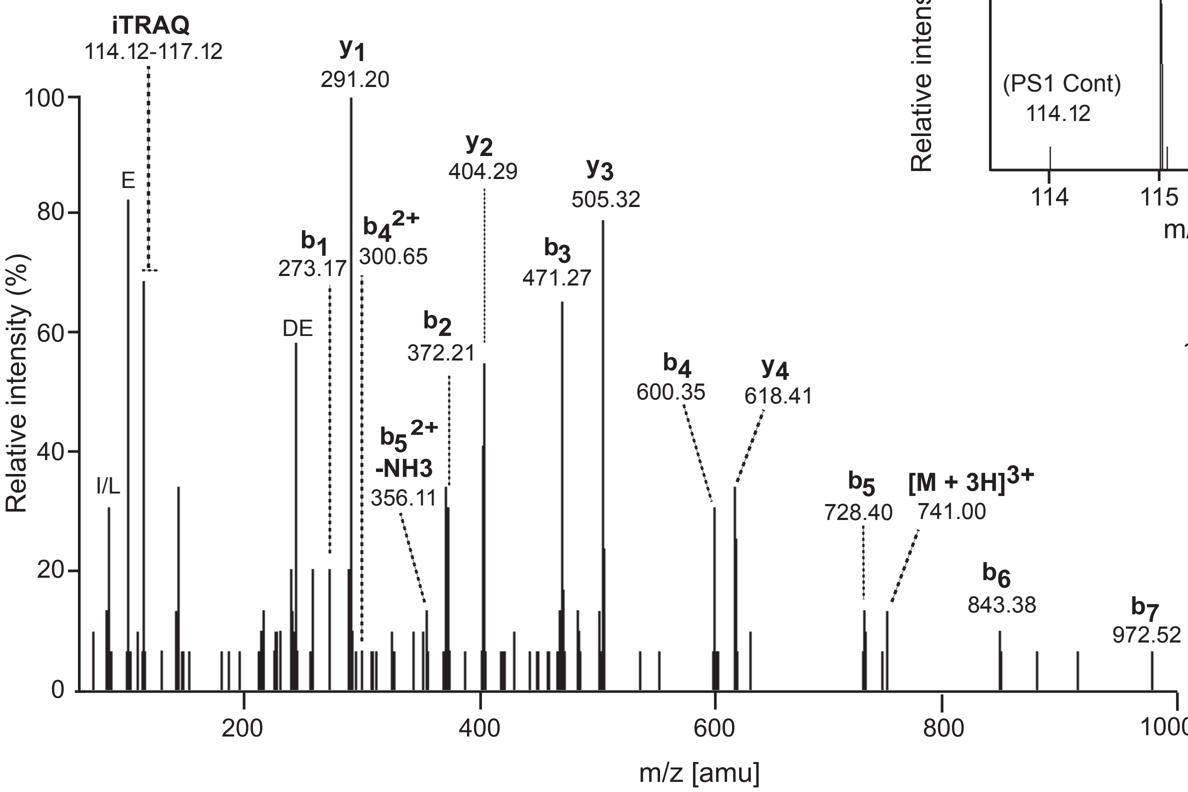

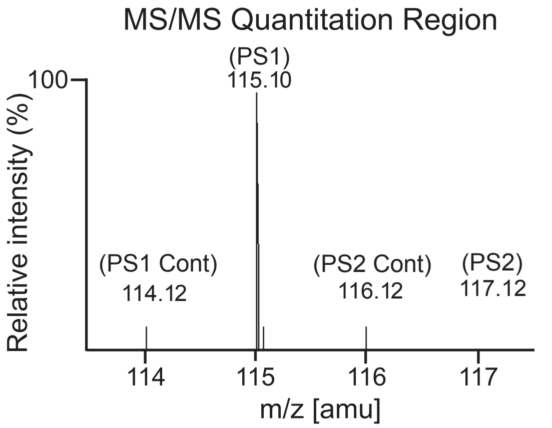

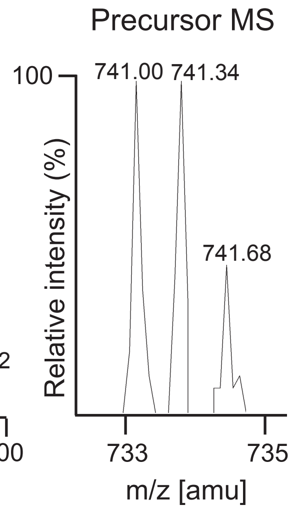

B Presenilin 2 (ENSP00000355747), MS/MS of m/z 544.57

QGPEDGENTAQWR (aa 36-48) + iTRAQ 114(N-term)

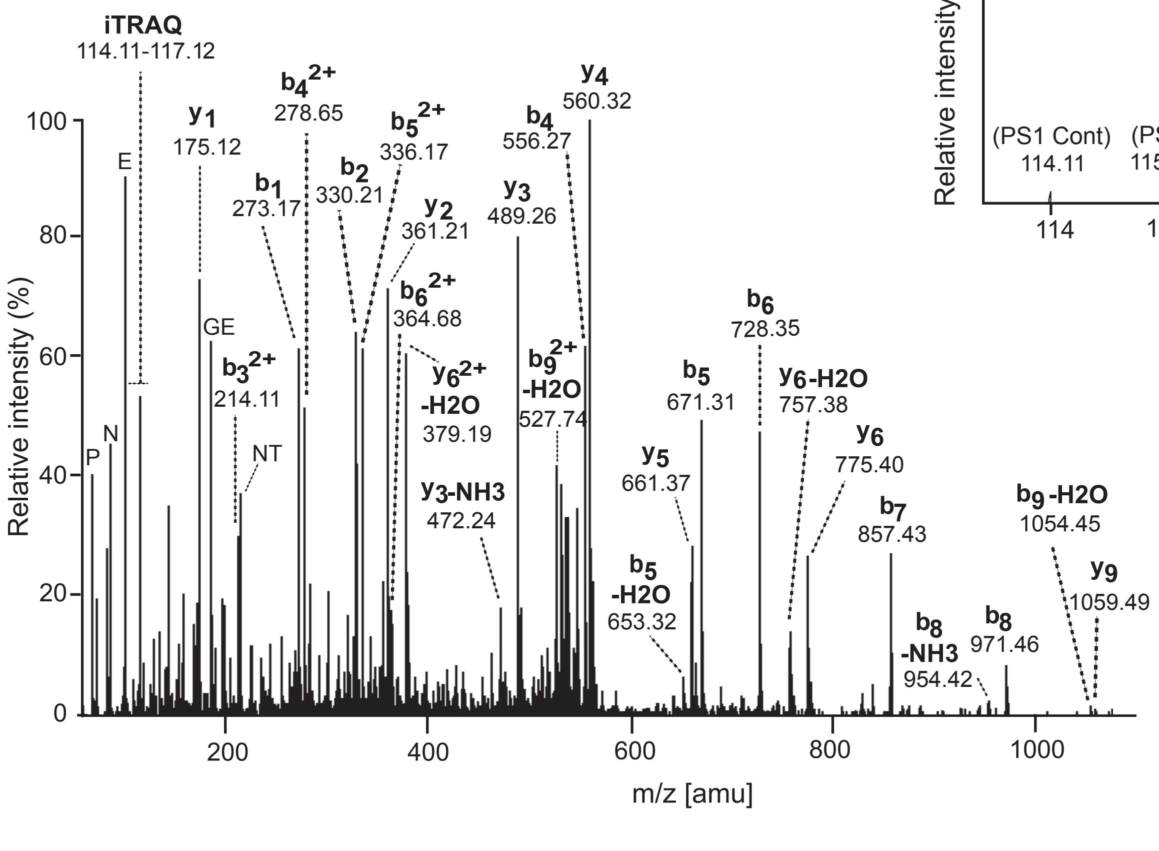

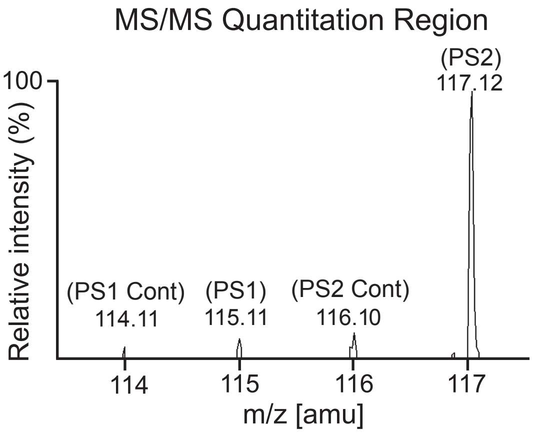

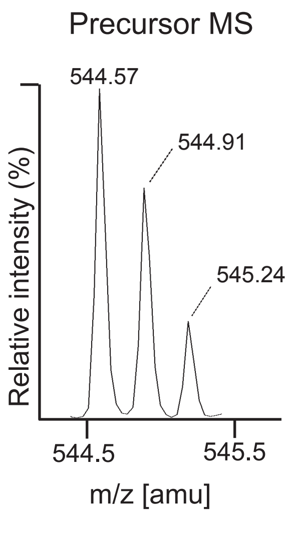

Suppl. Figure 4

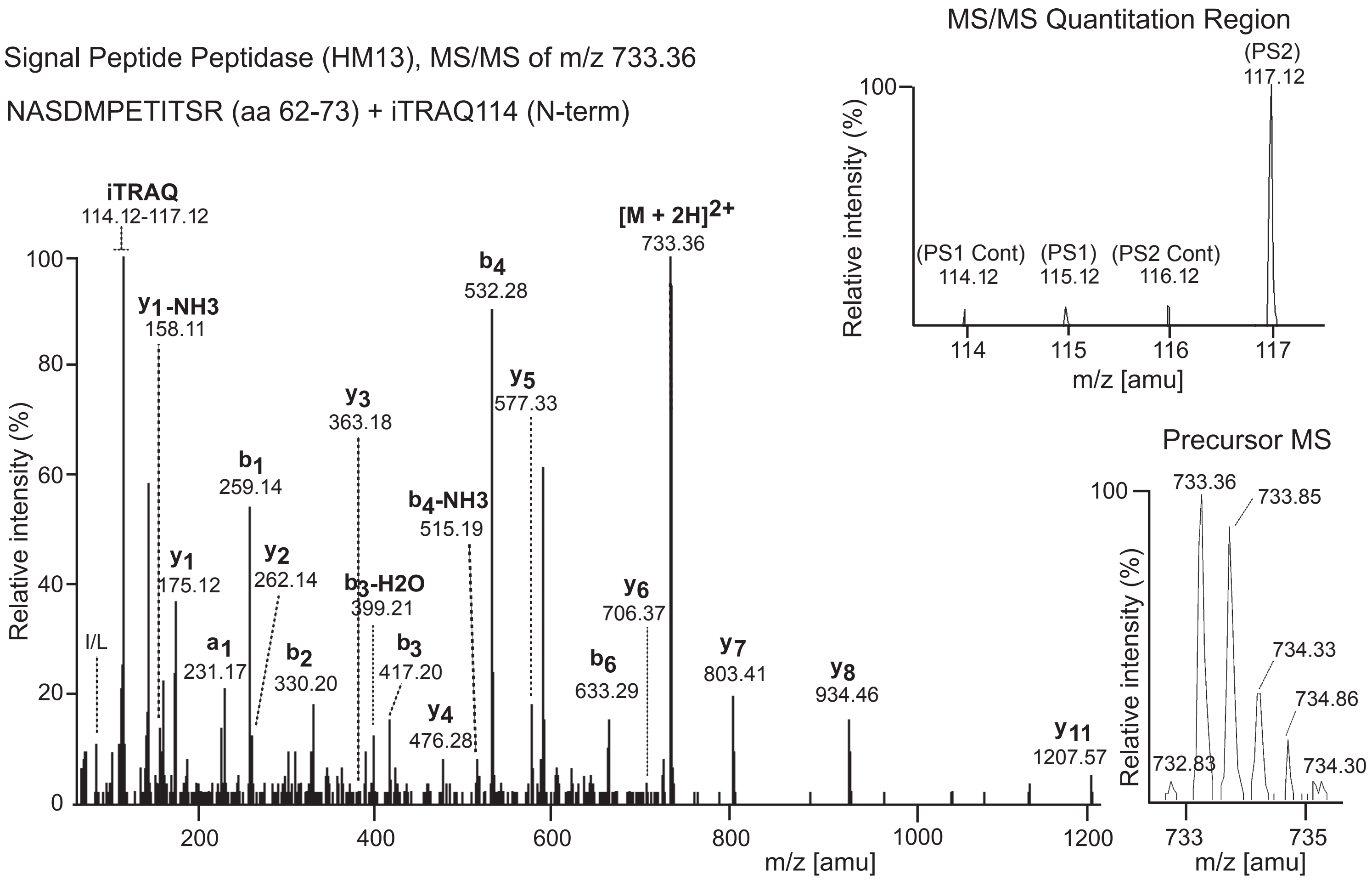

Supplement: Supplemental Data [file supp_M112.441840_jbc.M112.441840-1.pdf]
