# Supplementary material for: Interactome Analyses of Mature γ-Secretase Complexes Reveal Distinct Molecular Environments of Presenilin (PS) Paralogs and Preferential Binding of Signal Peptide Peptidase to PS2
Source: J Biol Chem. 2013 Apr 15;288(21):15352–66. doi: 10.1074/jbc.M112.441840 (PMC3663554; doi:10.1074/jbc.M112.441840)
Supplement: Supplemental Data [file supp_M112.441840_jbc.M112.441840-2.pdf]

Suppl. Table 1. Quantitative interactome analysis of human wild-type PS1 in transgenic mice.

Accessions	Symbol	Names	Conf.	Sequence	Modifications	Cleavages	dMass	Prec MW	Prec m/z	Theor MW	Theor m/z	z	iTRAQ Area 114	iTRAQ Area 115	Normalized 114	Normalized 115	iTRAQ Ratio 115:114
IPI:IPI00117124.1	PSEN1	Presenilin-1	99	DGQLIYTPFTEDTETVGQR	iTRAQ4plex@N-term		0.0	2313.14	772.05	2313.12	772.05	3	1.7	58.7	2.8	97.2	34.5
			99	MLVETAQER	iTRAQ4plex@N-term		0.0	1219.65	610.83	1219.64	610.82	2	124.1	5561.2	2.2	97.8	44.8
			99	MLVETAQER	iTRAQ4plex@N-term		0.0	1219.65	610.83	1219.64	610.82	2	127.5	11187.9	1.1	98.9	87.7
			99	MLVETAQER	iTRAQ4plex@N-term; Dimethyl(R)@9		0.0	1247.64	624.83	1247.67	624.84	2	96.1	2074.0	4.4	95.6	21.6
			99	QVVEQDEEEDEELTLK	iTRAQ4plex@N-term; iTRAQ4plex(K)@16		0.0	2220.10	741.04	2220.09	741.04	3	27.2	255.7	9.6	90.4	9.4
			99	QVVEQDEEEDEELTLK	iTRAQ4plex@N-term; Lys->Gln@16		0.0	2075.93	692.98	2075.95	692.99	3	6.2	130.8	4.5	95.5	21.1
			98	MLVETAQER	iTRAQ4plex@N-term		0.0	1219.64	610.83	1219.64	610.82	2	54.2	2420.6	2.2	97.8	44.7
			93	MLVETAQER	iTRAQ4plex@N-term; Dimethyl(R)@9		0.0	1247.63	624.82	1247.67	624.84	2	117.7	6484.2	1.8	98.2	55.1
			75	MLVETAQER	iTRAQ4plex@N-term		3.0	1222.62	612.32	1219.64	610.82	2	46.2	2388.4	1.9	98.1	51.7
			49	AAVQELSGSILTSEDPEER	iTRAQ4plex@N-term; Ser->Gly@13		0.0	2144.02	715.68	2144.07	715.70	3					
			11	AAVQELSGSILTSEDPEER	iTRAQ4plex@N-term; Ser->Gly@13		-0.1	2144.01	715.68	2144.07	715.70	3					
			10	MLVETAQER	iTRAQ4plex@N-term; Oxidation(M)@1		0.0	1235.62	618.82	1235.63	618.82	2					
																Average	41.2
																Stdev	23.3
IPI:IPI00118674.15	NCSTN	Nicastrin	99	SGAGVPEVVLR	iTRAQ4plex@N-term		0.9	1227.66	614.84	1226.71	614.36	2	776.9	14123.2	5.2	94.8	18.2
			99	SGAGVPEVVLR	iTRAQ4plex@N-term		0.0	1226.71	614.36	1226.71	614.36	2	419.6	19238.3	2.1	97.9	45.9
			99	LAQSQALPPSSLQR	iTRAQ4plex@N-term		-5.1	1633.83	817.92	1638.92	820.47	2	386.0	1303.3	22.9	77.1	3.4
			99	ALANVATVLAR	iTRAQ4plex@N-term		0.0	1241.72	621.87	1241.76	621.89	2	170.3	5548.2	3.0	97.0	32.6
			99	ALANVATVLAR	iTRAQ4plex@N-term		0.0	1241.75	621.88	1241.76	621.89	2	113.5	1848.6	5.8	94.2	16.3
			99	LAQSQALPPSSLQR	iTRAQ4plex@N-term		0.0	1638.93	547.32	1638.92	547.31	3	108.1	195.9	35.6	64.4	1.8
			99	SGAGVPEVVLRR	iTRAQ4plex@N-term	missed R-R@11	0.0	1382.78	461.93	1382.81	461.94	3	70.5	573.7	10.9	89.1	8.1
			99	APDVTTLSR	iTRAQ4plex@N-term		0.0	1102.59	552.30	1102.61	552.31	2	55.6	10531.6	0.5	99.5	189.3
			99	LAQSQALPPSSLQR	iTRAQ4plex@N-term		0.0	1638.88	820.45	1638.92	820.47	2	14.3	509.9	2.7	97.3	35.7
			99	LENIDSFVELGQVALR	iTRAQ4plex@N-term		0.0	1946.08	649.70	1946.06	649.69	3	1.7	13.0	11.5	88.5	7.7
			99	LENIDSFVELGQVALR	iTRAQ4plex@N-term		0.0	1946.03	649.68	1946.06	649.69	3	1.7	16.3	9.4	90.6	9.6
			99	LENIDSFVELGQVALR	iTRAQ4plex@N-term		2.0	1948.07	650.36	1946.06	649.69	3	1.7	17.4	8.9	91.1	10.2
			99	SGAGVPEVVLR	iTRAQ4plex@N-term; Dimethyl(R)@11		0.0	1254.74	628.38	1254.74	628.38	2	124.7	1093.7	10.2	89.8	8.8
			99	SGAGVPEVVLR	iTRAQ4plex@N-term; Dimethyl(R)@11		0.0	1254.73	628.37	1254.74	628.38	2	123.3	1832.3	6.3	93.7	14.9
			99	SGAGVPEVVLR	iTRAQ4plex@N-term; Dimethyl(R)@11		0.0	1254.74	628.38	1254.74	628.38	2	93.0	1008.7	8.4	91.6	10.8
			99	ALANVATVLAR	iTRAQ4plex@N-term; Dimethyl(R)@11		0.0	1269.80	635.91	1269.79	635.90	2	30.2	1675.2	1.8	98.2	55.5
			99	NQVEDLLATLEK	iTRAQ4plex@N-term; iTRAQ4plex(K)@12		0.0	1659.94	830.98	1659.93	830.97	2	34.3	1263.3	2.6	97.4	36.8
			99	NQVEDLLATLEK	iTRAQ4plex@N-term; iTRAQ4plex(K)@12		0.0	1659.92	554.32	1659.93	554.32	3	1.7	111.7	1.5	98.5	65.7
			99	WVLTDGPNPPYMVLLEGK	iTRAQ4plex@N-term; iTRAQ4plex(K)@18		0.0	2316.29	773.10	2316.24	773.09	3	1.7	14.1	10.7	89.3	8.3
			99	WVLTDGPNPPYMVLLEGK	iTRAQ4plex@N-term; iTRAQ4plex(K)@18		0.0	2316.24	773.09	2316.24	773.09	3	1.7	35.7	4.5	95.5	21.0
			99	LLYGFLVK	iTRAQ4plex@N-term; iTRAQ4plex(K)@8		0.0	1239.76	620.89	1239.78	620.90	2	28.6	3653.9	0.8	99.2	127.9
			99	LLYGFLVK	iTRAQ4plex@N-term; iTRAQ4plex(K)@8		0.0	1239.79	620.90	1239.78	620.90	2	21.3	3068.6	0.7	99.3	144.4
			99	LLYGFLVK	iTRAQ4plex@N-term; iTRAQ4plex(K)@8		0.0	1239.75	620.88	1239.78	620.90	2	23.3	3146.5	0.7	99.3	135.1
			99	LLYGFLVK	iTRAQ4plex@N-term; iTRAQ4plex(K)@8		0.0	1239.79	620.90	1239.78	620.90	2	29.2	691.5	4.1	95.9	23.6
			99	NQVEDLLATLEK	iTRAQ4plex@N-term; Lys->Arg@12		0.0	1543.83	772.92	1543.83	772.92	2	22.5	235.4	8.7	91.3	10.5
			99	NQVEDLLATLEK	iTRAQ4plex@N-term; Lys->Gln@12		0.1	1515.84	758.93	1515.79	758.90	2	1.7	95.7	1.7	98.3	56.3
			99	MVYDMENGKFPVR	iTRAQ4plex@N-term; Lys->Gln@9	missed K-F@9	0.0	1728.85	577.29	1728.81	577.28	3	16.5	399.7	4.0	96.0	24.2
			98	LENIDSFVELGQVALR	iTRAQ4plex@N-term; Dimethyl(R)@16		0.0	1974.09	659.04	1974.09	659.04	3	1.7	5.4	23.8	76.2	3.2
			98	NQVEDLLATLEK	iTRAQ4plex@N-term; Thr->Glu@9; iTRAQ4plex(K)@12		0.1	1687.99	845.00	1687.92	844.97	2	1.7	200.4	0.8	99.2	117.9
			97	SYLDDRPLQH	iTRAQ4plex@N-term	cleaved H-Y@C-term	0.0	1386.71	463.24	1386.70	463.24	3	145.1	435.5	25.0	75.0	3.0
			97	ALSPAFELSQWSSTEYSTWAESR	iTRAQ4plex@N-term		0.0	2776.34	926.45	2776.31	926.44	3	6.8	13.9	33.0	67.0	2.0
			97	MVYDMENGK	iTRAQ4plex@N-term; Lys->Gln@9		0.0	1229.54	615.78	1229.52	615.77	2	51.7	437.8	10.6	89.4	8.5
			94	VVVAATR	iTRAQ4plex@N-term		0.0	858.54	430.28	858.54	430.28	2	122.9	16220.3	0.8	99.2	132.0
			94	LLYGFLVK	iTRAQ4plex@N-term; Lys->Gln@8		0.0	1095.68	548.85	1095.65	548.83	2	19.2	536.4	3.5	96.5	27.9
			84	LLYGFLVK	iTRAQ4plex@N-term; Lys->Gln@8		0.0	1095.65	548.83	1095.65	548.83	2	38.6	346.5	10.0	90.0	9.0
			78	NQVEDLLATLEK	iTRAQ4plex@N-term; Lys->Arg@12		0.1	1543.90	515.64	1543.83	515.62	3	1.7	17.4	8.9	91.1	10.2
			54	LLYGFLVK	iTRAQ4plex@N-term; Lys->Arg@8		0.0	1123.73	562.87	1123.69	562.85	2	7.7	445.5	1.7	98.3	58.2
			54	LLYGFLVK	iTRAQ4plex@N-term; Methyl(L)@6; Val->Leu@7; iTRAQ4plex(K)@8		0.0	1267.79	634.90	1267.81	634.91	2	6.6	305.7	2.1	97.9	46.1
			43	NQVEDLLATLEK	iTRAQ4plex@N-term; iTRAQ4plex(T)@9; Lys->Arg@12		0.1	1688.01	563.68	1687.93	563.65	3					
																Average	40.5
																Stdev	48.3
IPI:IPI00266590.4	APH1A	Anterior pharynx defective 1a	99	VMVYSALR	iTRAQ4plex@N-term		0.0	1081.60	541.81	1081.61	541.81	2	107.2	2920.2	3.5	96.5	27.2
																Average	27.2
																Stdev	0.0
IPI:IPI00229029.1	APH1B	Anterior pharynx defective 1b	99	SINPEETAPSMR	iTRAQ4plex@N-term		-7.0	1467.73	734.87	1474.72	738.37	2	369.1	1816.5	16.9	83.1	4.9
			99	SINPEETAPSMR	iTRAQ4plex@N-term		0.0	1474.74	738.38	1474.72	738.37	2	162.1	1772.3	8.4	91.6	10.9
			99	SINPEETAPSMR	iTRAQ4plex@N-term		0.0	1474.74	738.38	1474.72	738.37	2	19.3	873.5	2.2	97.8	45.3
			97	ASEGLK	iTRAQ4plex@N-term; iTRAQ4plex(K)@6		0.0	891.56	446.79	891.53	446.77	2	1.7	65.0	2.5	97.5	38.2
			76	ASEGLK	iTRAQ4plex@N-term; iTRAQ4plex(K)@6		1.0	892.56	447.29	891.53	446.77	2	5.0	163.2	3.0	97.0	32.5
			70	ASEGLK	iTRAQ4plex@N-term; iTRAQ4plex(K)@6		-2.0	889.56	445.79	891.53	446.77	2	12.5	309.4	3.9	96.1	24.8
																Average	26.1
																Stdev	15.7
IPI:IPI00230751.2	CTNNA2	Catenin alpha 2	99	AEVQNLGGELIVSGLDSATSLIQAAK	iTRAQ4plex@N-term; iTRAQ4plex(K)@26		0.0	2871.60	958.21	2871.58	958.20	3	1.7	6.5	20.7	79.3	3.8
			99	LLSETVMPR	iTRAQ4plex@N-term		0.0	1188.64	595.33	1188.67	595.34	2	1370.5	3852.5	26.2	73.8	2.8
			99	ALLSAVTR	iTRAQ4plex@N-term		0.0	973.58	487.80	973.60	487.81	2	1062.4	3523.0	23.2	76.8	3.3
			99	NATNEQDLANR	iTRAQ4plex@N-term		0.1	1388.73	695.37	1388.68	695.35	2	998.9	6356.5	13.6	86.4	6.4
			99	IILDPMTFSEAR	iTRAQ4plex@N-term		0.0	1535.82	768.92	1535.81	768.91	2	476.4	1283.4	27.1	72.9	2.7
			99	QDLLAYLQR	iTRAQ4plex@N-term		0.0	1262.71	632.36	1262.71	632.36	2	400.7	1451.7	21.6	78.4	3.6
			99	NATMLYTASQAFLR	iTRAQ4plex@N-term		0.0	1729.89	865.95	1729.89	865.95	2	376.0	825.5	31.3	68.7	2.2
			99	QDLLAYLQR	iTRAQ4plex@N-term		0.0	1262.71	632.36	1262.71	632.36	2	338.6	1347.5	20.1	79.9	4.0
			99	IILDPMTFSEAR	iTRAQ4plex@N-term		0.0	1535.81	768.91	1535.81	768.91	2	304.8	870.6	25.9	74.1	2.9
			99	LLILADMADVMR	iTRAQ4plex@N-term		0.0	1503.84	752.93	1503.83	752.92	2	287.0	1357.8	17.4	82.6	4.7
			99	QDLLAYLQR	iTRAQ4plex@N-term		0.0	1262.71	632.36	1262.71	632.36	2	258.6	832.9	23.7	76.3	3.2
			99	EELVAAVEDVR	iTRAQ4plex@N-term		0.0	1372.70	687.36	1372.73	687.37	2	170.5	1141.0	13.0	87.0	6.7
			99	IILDPMTFSEAR	iTRAQ4plex@N-term		0.0	1535.82	768.91	1535.81	768.91	2	83.2	225.6	27.0	73.0	2.7
			99	IILDPMTFSEAR	iTRAQ4plex@N-term		0.0	1535.82	768.92	1535.81	768.91	2	73.9	231.3	24.2	75.8	3.1
			99	LLILADMADVMR	iTRAQ4plex@N-term		0.0	1503.87	752.94	1503.83	752.92	2	52.6	212.5	19.8	80.2	4.0
			99	QALQDLLSEYMNNTGR	iTRAQ4plex@N-term		0.0	1996.00	666.34	1995.98	666.33	3	25.6	49.9	33.9	66.1	1.9
			99	NATMLYTASQAFLR	iTRAQ4plex@N-term		0.0	1729.88	577.63	1729.89	577.64	3	20.2	48.9	29.2	70.8	2.4
			99	LLILADMADVMR	iTRAQ4plex@N-term		1.1	1504.89	753.45	1503.83	752.92	2	17.2	80.6	17.6	82.4	4.7
			99	QALQDLLSEYMNNTGR	iTRAQ4plex@N-term		1.0	1996.98	666.67	1995.98	666.33	3	13.1	41.8	23.9	76.1	3.2
			99	QALQDLLSEYMNNTGR	iTRAQ4plex@N-term		0.0	1995.99	666.34	1995.98	666.33	3	11.6	34.3	25.2	74.8	3.0
			99	NATMLYTASQAFLR	iTRAQ4plex@N-term		0.0	1729.88	577.63	1729.89	577.64	3	10.4	17.0	37.9	62.1	1.6
			99	TSVQTEDDQLIAGQSAR	iTRAQ4plex@N-term		0.0	1961.98	655.00	1961.98	655.00	3	10.0	27.9	26.3	73.7	2.8
			99	LLILADMADVMR	iTRAQ4plex@N-term		0.0	1503.84	752.93	1503.83	752.92	2	7.6	93.1	7.5	92.5	12.3
			99	DEMAAAR	iTRAQ4plex@N-term		0.1	906.49	454.25	906.44	454.22	2	6.5	23.6	21.5	78.5	3.7
			99	QALQDLLSEYMNNTGR	iTRAQ4plex@N-term		0.0	1996.02	666.35	1995.98	666.33	3	4.8	19.4	19.8	80.2	4.1
			99	IILDPMTFSEAR	iTRAQ4plex@N-term; Ala->Val@11		0.0	1563.85	782.93	1563.85	782.93	2	120.5	857.7	12.3	87.7	7.1
			99	IILDPMTFSEAR	iTRAQ4plex@N-term; Ala->Val@11		0.0	1563.88	782.95	1563.85	782.93	2	45.1	369.0	10.9	89.1	8.2
			99	IILDPMTFSEAR	iTRAQ4plex@N-term; Ala->Val@11		0.0	1563.83	782.92	1563.85	782.93	2	43.2	220.6	16.4	83.6	5.1
			99	LLEPLVTQVTTLVNTSNK	iTRAQ4plex@N-term; Deamidated(Q)@8; Methyl(T)@11; iTRAQ4plex(K)@18		0.0	2272.31	758.44	2272.31	758.45	3	8.3	22.5	27.0	73.0	2.7
			99	SRTSVQTEDDQLIAGQSAR	iTRAQ4plex@N-term; Deamidated(R)@2	missed R-T@2	0.0	2206.10	736.37	2206.09	736.37	3	55.5	82.1	40.3	59.7	1.5
			99	LLEPLVTQVTTLVNTSNK	iTRAQ4plex@N-term; Dehydrated(T)@10; iTRAQ4plex(K)@18		0.0	2239.34	747.45	2239.30	747.44	3	1.7	5.4	23.8	76.2	3.2
			99	IILDPMTFSEAR	iTRAQ4plex@N-term; Dimethyl(R)@12		0.0	1563.86	782.94	1563.85	782.93	2	134.2	732.8	15.5	84.5	5.5
			99	NATMLYTASQAFLR	iTRAQ4plex@N-term; Dimethyl(R)@14		0.0	1757.91	879.96	1757.93	879.97	2	47.8	342.9	12.2	87.8	7.2
			99	NATMLYTASQAFLR	iTRAQ4plex@N-term; Dimethyl(R)@14		0.0	1757.91	586.98	1757.93	586.98	3	1.7	45.7	3.6	96.4	26.9
			99	NATMLYTASQAFLR	iTRAQ4plex@N-term; Dimethyl(R)@14		0.0	1757.91	586.98	1757.93	586.98	3	1.7	16.3	9.4	90.6	9.6
			99	QALQDLLSEYMNNTGR	iTRAQ4plex@N-term; Dimethyl(R)@16		0.0	2024.03	675.68	2024.01	675.68	3	1.7	6.5	20.7	79.3	3.8
			99	QDLLAYLQR	iTRAQ4plex@N-term; Dimethyl(R)@9		0.0	1290.74	646.38	1290.74	646.38	2	13.6	228.1	5.6	94.4	16.7
			99	NTSDVINAAK	iTRAQ4plex@N-term; iTRAQ4plex(K)@10		0.0	1319.76	660.89	1319.73	660.87	2	911.8	3485.6	20.7	79.3	3.8
			99	IVEEALEAVK	iTRAQ4plex@N-term; iTRAQ4plex(K)@10		0.0	1387.81	694.91	1387.82	694.92	2	121.9	418.0	22.6	77.4	3.4
			99	WDDSGNDIIVLAK	iTRAQ4plex@N-term; iTRAQ4plex(K)@13		-0.1	1732.87	867.44	1732.92	867.47	2	183.3	490.4	27.2	72.8	2.7
			99	WDDSGNDIIVLAK	iTRAQ4plex@N-term; iTRAQ4plex(K)@13		0.0	1732.89	578.64	1732.92	578.65	3	99.9	320.1	23.8	76.2	3.2
			99	VYGTAAVNSPVVSWK	iTRAQ4plex@N-term; iTRAQ4plex(K)@15		0.0	1865.04	622.69	1865.03	622.68	3	67.4	158.7	29.8	70.2	2.4
			99	QALQDLLSEYMNNTGRK	iTRAQ4plex@N-term; iTRAQ4plex(K)@17	missed R-K@16	0.0	2268.14	757.06	2268.18	757.07	3	52.2	73.9	41.4	58.6	1.4
			99	AHVLAASVEQATQNFLEK	iTRAQ4plex@N-term; iTRAQ4plex(K)@18		0.0	2243.24	748.75	2243.22	748.75	3	20.3	95.6	17.5	82.5	4.7
			99	LLEPLVTQVTTLVNTSNK	iTRAQ4plex@N-term; iTRAQ4plex(K)@18		0.1	2257.38	753.47	2257.31	753.45	3	1.7	12.0	12.4	87.6	7.0
			99	LLEPLVTQVTTLVNTSNKGPSG	iTRAQ4plex@N-term; iTRAQ4plex(K)@18; Ser->Ala@21	cleaved G-K@C-term; missed K-G@18	0.0	2539.40	847.47	2539.45	847.49	3	26.7	19.6	57.7	42.3	0.7
			99	LLEPLVTQVTTLVNTSNKGPSG	iTRAQ4plex@N-term; iTRAQ4plex(K)@18; Ser->Ala@21	cleaved G-K@C-term; missed K-G@18	0.0	2539.42	847.48	2539.45	847.49	3	12.4	10.4	54.5	45.5	0.8
			99	AEVQNLGGELIVSGLDSATSLIQAAK	iTRAQ4plex@N-term; iTRAQ4plex(K)@26		-0.9	2870.66	718.67	2871.58	718.90	4	7.1	5.2	58.0	42.0	0.7
			99	ESQDLKEELVAAVEDVR	iTRAQ4plex@N-term; iTRAQ4plex(K)@6		0.0	2217.19	740.07	2217.17	740.07	3	15.6	73.1	17.6	82.4	4.7
			99	NTSDVINAAK	iTRAQ4plex@N-term; Lys->Arg@10		0.0	1203.67	602.84	1203.63	602.82	2	155.4	555.0	21.9	78.1	3.6
			99	IVEEALEAVK	iTRAQ4plex@N-term; Lys->Arg@10		0.0	1271.74	636.88	1271.72	636.87	2	18.3	150.0	10.9	89.1	8.2
			99	AIMAQLPQEEK	iTRAQ4plex@N-term; Lys->Arg@11		0.0	1428.78	715.40	1428.75	715.38	2	217.4	759.7	22.3	77.7	3.5
			99	AIMAQLPQEEK	iTRAQ4plex@N-term; Lys->Arg@11		0.0	1428.77	715.39	1428.75	715.38	2	144.1	461.5	23.8	76.2	3.2
			99	WDDSGNDIIVLAK	iTRAQ4plex@N-term; Lys->Arg@13		0.1	1616.88	809.45	1616.83	809.42	2	32.8	155.0	17.4	82.6	4.7
			99	AHVLAASVEQATQNFLEK	iTRAQ4plex@N-term; Lys->Arg@18		0.1	2127.18	710.07	2127.12	710.05	3	6.6	18.2	26.8	73.2	2.7
			99	GHTGIGELAAALNEFDNK	iTRAQ4plex@N-term; Lys->Arg@18		0.0	2028.03	677.02	2028.01	677.01	3	4.7	20.5	18.8	81.2	4.3
			99	LLEPLVTQVTTLVNTSNK	iTRAQ4plex@N-term; Lys->Arg@18		0.1	2141.28	714.77	2141.22	714.75	3	1.7	10.9	13.5	86.5	6.4
			99	QVQEAIAGISSAAQATSPTDEAK	iTRAQ4plex@N-term; Lys->Arg@23		0.0	2444.19	815.74	2444.23	815.75	3	11.8	27.8	29.8	70.2	2.4
			99	IVEEALEAVK	iTRAQ4plex@N-term; Lys->Gln@10		0.0	1243.70	622.86	1243.68	622.85	2	87.6	257.7	25.4	74.6	2.9
			99	IVEEALEAVK	iTRAQ4plex@N-term; Lys->Gln@10		0.0	1243.69	622.85	1243.68	622.85	2	56.9	194.0	22.7	77.3	3.4
			99	VAQDNMDVFKDQWEK	iTRAQ4plex@N-term; Lys->Gln@10; iTRAQ4plex(K)@15		0.0	2140.02	714.35	2140.01	714.35	3	125.3	275.9	31.2	68.8	2.2
			99	NLMNAVVLTVK	iTRAQ4plex@N-term; Lys->Gln@11		0.0	1344.80	673.40	1344.76	673.39	2	491.4	573.2	46.2	53.8	1.2
			99	EELVAAVEDVRK	iTRAQ4plex@N-term; Lys->Gln@12	missed R-K@11	0.0	1500.81	501.28	1500.79	501.27	3	72.8	160.7	31.2	68.8	2.2
			99	WDDSGNDIIVLAK	iTRAQ4plex@N-term; Lys->Gln@13		0.0	1588.83	795.42	1588.79	795.40	2	265.7	439.7	37.7	62.3	1.7
			99	WDDSGNDIIVLAK	iTRAQ4plex@N-term; Lys->Gln@13		0.1	1588.84	795.43	1588.79	795.40	2	187.9	365.6	33.9	66.1	1.9
			99	VYGTAAVNSPVVSWK	iTRAQ4plex@N-term; Lys->Gln@15		0.0	1720.89	574.64	1720.89	574.64	3	40.1	54.9	42.2	57.8	1.4
			99	AHVLAASVEQATQNFLEK	iTRAQ4plex@N-term; Lys->Gln@18		0.0	2099.12	700.71	2099.08	700.70	3	62.3	97.5	39.0	61.0	1.6
			99	GHTGIGELAAALNEFDNK	iTRAQ4plex@N-term; Lys->Gln@18		0.0	1999.98	667.67	1999.97	667.66	3	18.5	94.6	16.4	83.6	5.1
			99	GHTGIGELAAALNEFDNK	iTRAQ4plex@N-term; Lys->Gln@18		0.1	2000.04	667.69	1999.97	667.66	3	6.3	27.9	18.5	81.5	4.4
			99	VIHIINAEMENYEAGVYTEK	iTRAQ4plex@N-term; Lys->Gln@20		0.0	2466.19	823.07	2466.19	823.07	3	25.3	58.8	30.1	69.9	2.3
			99	QVQEAIAGISSAAQATSPTDEAK	iTRAQ4plex@N-term; Lys->Gln@23		0.0	2416.19	806.40	2416.18	806.40	3	1.7	12.0	12.4	87.6	7.0
			99	VAQDNMDVFK	iTRAQ4plex@N-term; No iTRAQ4plex(K)@10		0.0	1309.63	655.82	1309.65	655.83	2	146.0	309.6	32.1	67.9	2.1
			99	AIMAQLPQEEK	iTRAQ4plex@N-term; No iTRAQ4plex(K)@11		0.0	1400.76	701.39	1400.75	701.38	2	354.0	728.6	32.7	67.3	2.1
			99	AIMAQLPQEEK	iTRAQ4plex@N-term; No iTRAQ4plex(K)@11		0.0	1400.76	701.39	1400.75	701.38	2	318.9	587.0	35.2	64.8	1.8
			99	VIHIINAEMENYEAGVYTEK	iTRAQ4plex@N-term; No iTRAQ4plex(K)@20		0.0	2466.18	823.07	2466.22	823.08	3	7.3	50.8	12.6	87.4	7.0
			99	ESQDLKEELVAAVEDVRK	iTRAQ4plex@N-term; No iTRAQ4plex(K)@6; iTRAQ4plex(K)@18	missed R-K@17	0.0	2345.24	587.32	2345.27	587.32	4	8.7	11.6	42.9	57.1	1.3
			99	IILDPMTFSEAR	iTRAQ4plex@N-term; Oxidation(M)@6		0.0	1551.77	776.89	1551.81	776.91	2	86.3	240.6	26.4	73.6	2.8
			99	QALQDLLSEYMNNTGR	iTRAQ4plex@N-term; Oxidation(Y)@10		0.0	2011.98	671.67	2011.98	671.67	3	4.7	21.5	17.9	82.1	4.6
			99	QVQEAIAGISSAAQATSPTDEAK	iTRAQ4plex@N-term; Phospho(S)@17; iTRAQ4plex(K)@23		-0.1	2640.23	881.08	2640.29	881.10	3	13.0	46.2	21.9	78.1	3.6
			99	CVIALQEGDVDTLDR	iTRAQ4plex@N-term; Pyridylethyl(C)@1		0.0	1894.93	632.65	1894.96	632.66	3	423.7	873.7	32.7	67.3	2.1
			99	MAATQIDSLCPQVINAALTLAAR	iTRAQ4plex@N-term; Pyridylethyl(C)@10	cleaved R-P@C-term	0.0	2619.44	874.15	2619.40	874.14	3	1.7	16.3	9.4	90.6	9.6
			99	MAATQIDSLCPQVINAALTLAARPQSK	iTRAQ4plex@N-term; Pyridylethyl(C)@10; Lys->Gln@27		0.1	3059.69	765.93	3059.60	765.91	4	5.0	13.9	26.4	73.6	2.8
			99	IASSEFADDPCSSVK	iTRAQ4plex@N-term; Pyridylethyl(C)@11; Lys->Arg@15		0.0	1831.87	611.63	1831.85	611.63	3	33.7	129.7	20.6	79.4	3.9
			99	LESIISGAALMADSSCTR	iTRAQ4plex@N-term; Pyridylethyl(C)@16		0.0	2073.04	692.02	2073.04	692.02	3	17.2	30.8	35.8	64.2	1.8
			99	LESIISGAALMADSSCTR	iTRAQ4plex@N-term; Pyridylethyl(C)@16; Thr->Glu@17		0.0	2101.02	701.35	2101.03	701.35	3	1.7	18.5	8.4	91.6	10.9
			99	LVEVANLACSISNNEEGVK	iTRAQ4plex@N-term; Pyridylethyl(C)@9; iTRAQ4plex(K)@19		0.0	2381.26	596.32	2381.25	596.32	4	28.9	109.5	20.9	79.1	3.8
			99	LLEPLVTQVTTLVNTSNK	iTRAQ4plex@N-term; Val->Trp@9; iTRAQ4plex(K)@18		0.1	2344.44	782.49	2344.32	782.45	3	1.7	26.1	6.1	93.9	15.3
			98	ALLSAVTR	iTRAQ4plex@N-term		1.0	974.59	488.30	973.60	487.81	2	1495.3	5895.5	20.2	79.8	3.9
			98	IAEAGSR	iTRAQ4plex@N-term		1.0	847.51	424.76	846.47	424.24	2	66.8	124.1	35.0	65.0	1.9
			98	QDLLAYLQR	iTRAQ4plex@N-term; Gln->Arg@8		0.0	1290.72	646.37	1290.75	646.38	2	41.1	327.7	11.1	88.9	8.0
			98	AIMAQLPQEEK	iTRAQ4plex@N-term; Lys->Gln@11		0.0	1400.75	701.38	1400.71	701.36	2	198.3	481.4	29.2	70.8	2.4
			96	LVYDGVR	iTRAQ4plex@N-term		0.0	964.56	483.29	964.55	483.28	2	2886.2	8741.0	24.8	75.2	3.0
			96	HISPVQALSEFK	iTRAQ4plex@N-term; Lys->Gln@12		0.0	1498.78	500.60	1498.79	500.60	3	68.1	139.1	32.9	67.1	2.0
			95	LLSETVMPR	iTRAQ4plex@N-term; Dimethyl(R)@9		0.0	1216.70	609.36	1216.70	609.36	2	107.2	771.7	12.2	87.8	7.2
			95	LLEPLVTQVTTLVNTSNKGPSGK	iTRAQ4plex@N-term; iTRAQ4plex(K)@18; Lys->Gln@23	missed K-G@18	0.1	2683.56	671.90	2683.50	671.88	4	7.0	8.4	45.4	54.6	1.2
			95	ESQDLKEELVAAVEDVRK	iTRAQ4plex@N-term; iTRAQ4plex(K)@6; Lys->Gln@18	missed R-K@17	0.0	2345.25	587.32	2345.23	587.32	4	13.5	81.9	14.1	85.9	6.1
			94	DEMAAAR	iTRAQ4plex@N-term		-1.9	904.49	453.25	906.44	454.22	2	19.8	58.8	25.2	74.8	3.0
			94	IVEEALEAVK	iTRAQ4plex@N-term; iTRAQ4plex(K)@10		0.0	1387.80	463.61	1387.82	463.61	3	16.1	61.1	20.8	79.2	3.8
			92	EYAQVFR	iTRAQ4plex@N-term		0.0	1055.57	528.79	1055.55	528.78	2	471.3	1273.3	27.0	73.0	2.7
			91	LNYVAAR	iTRAQ4plex@N-term		0.0	949.53	475.77	949.55	475.78	2	4302.8	13784.8	23.8	76.2	3.2
			91	NLMNAVVLTVK	iTRAQ4plex@N-term; iTRAQ4plex(K)@11		0.0	1488.87	497.30	1488.89	497.31	3	45.9	46.0	49.9	50.1	1.0
			91	GEQIAK	iTRAQ4plex@N-term; iTRAQ4plex(K)@6		2.1	934.62	468.32	932.55	467.28	2	15.4	80.6	16.0	84.0	5.3
			91	ASYVASTK	iTRAQ4plex@N-term; iTRAQ4plex(K)@8		0.0	1113.65	557.83	1113.63	557.82	2	1056.1	2593.3	28.9	71.1	2.5
			91	LLILADMADVMR	iTRAQ4plex@N-term; Oxidation(M)@11		0.1	1519.88	760.94	1519.82	760.92	2	15.9	66.5	19.3	80.7	4.2
			89	LLEPLVTQVTTLVNTSNK	iTRAQ4plex@N-term; iTRAQ4plex(S)@16; Lys->Arg@18		0.1	2285.39	762.80	2285.32	762.78	3	1.7	13.0	11.5	88.5	7.7
			88	IILDPMTFSEAR	iTRAQ4plex@N-term		-7.0	1528.84	765.43	1535.81	768.91	2	99.6	228.2	30.4	69.6	2.3
			85	AIMAQLPQEEK	iTRAQ4plex@N-term; Methyl(E)@9; Methyl(E)@10; iTRAQ4plex(K)@11		0.0	1572.88	525.30	1572.88	525.30	3	50.0	184.5	21.3	78.7	3.7
			81	QALQDLLSEYMNNTGR	iTRAQ4plex@N-term; Oxidation(M)@11; Dimethyl(R)@16		0.0	2040.03	681.02	2040.01	681.01	3	5.1	9.6	34.9	65.1	1.9
			78	GEQIAK	iTRAQ4plex@N-term; iTRAQ4plex(K)@6		0.0	932.58	467.30	932.55	467.28	2	135.3	353.6	27.7	72.3	2.6
			74	IAEAGSR	iTRAQ4plex@N-term		1.0	847.50	424.76	846.47	424.24	2	142.4	159.1	47.2	52.8	1.1
			62	LLEPLVTQVTTLVNTSNK	iTRAQ4plex@N-term; iTRAQ4plex(K)@18		0.0	2257.30	565.33	2257.31	565.34	4	6.9	11.7	37.1	62.9	1.7
			60	NTSDVINAAK	iTRAQ4plex@N-term; Lys->Gln@10		0.1	1175.65	588.83	1175.59	588.80	2	723.5	2231.8	24.5	75.5	3.1
			53	QALQDLLSEYMNNTGR	iTRAQ4plex@N-term; Dehydrated(E)@9; Tyr->Trp@10		0.0	2001.02	668.01	2000.99	668.00	3	6.4	24.7	20.6	79.4	3.9
			47	IAEAGSR	iTRAQ4plex@N-term		-2.0	844.50	423.26	846.47	424.24	2					
			43	QQELK	iTRAQ4plex@N-term; iTRAQ4plex(K)@5		0.0	932.59	467.30	932.55	467.28	2					
			38	AEVQNLGGELIVSGLDSATSLIQAAK	iTRAQ4plex@N-term; iTRAQ4plex(K)@26		0.1	2871.66	718.92	2871.58	718.90	4					
			33	VLEATK	iTRAQ4plex@N-term; iTRAQ4plex(K)@6		0.0	947.61	474.81	947.59	474.80	2					
			30	QDLLAYLQR	iTRAQ4plex@N-term; Deamidated(Q)@1; Deamidated(Q)@8		0.0	1264.70	633.36	1264.68	633.35	2					
			29	TLTVER	iTRAQ4plex@N-term		0.0	861.50	431.76	861.50	431.76	2					
			23	ESQDLK	iTRAQ4plex@N-term; iTRAQ4plex(K)@6		0.0	1006.59	504.30	1006.55	504.28	2					
			16	QALQDLLSEYMNNTGR	iTRAQ4plex@N-term; Deamidated(N)@12; Dimethyl(R)@16		0.1	2025.05	676.02	2025.00	676.01	3					
			14	LNYVAAR	iTRAQ4plex@N-term; Ala->Val@6		0.0	977.56	489.79	977.58	489.80	2					
			14	LLEPLVTQVTTLVNTSNK	iTRAQ4plex@N-term; Ser->Asp@16; iTRAQ4plex(K)@18		0.1	2285.37	762.80	2285.31	762.78	3					
																Average	4.2
																Stdev	3.5
IPI:IPI00112963.1	CTNNA1	Catenin alpha 1	99	ALLSAVTR	iTRAQ4plex@N-term		0.0	973.58	487.80	973.60	487.81	2	1062.4	3523.0	23.2	76.8	3.3
			99	QDLLAYLQR	iTRAQ4plex@N-term		0.0	1262.71	632.36	1262.71	632.36	2	400.7	1451.7	21.6	78.4	3.6
			99	QDLLAYLQR	iTRAQ4plex@N-term		0.0	1262.71	632.36	1262.71	632.36	2	338.6	1347.5	20.1	79.9	4.0
			99	QDLLAYLQR	iTRAQ4plex@N-term		0.0	1262.71	632.36	1262.71	632.36	2	258.6	832.9	23.7	76.3	3.2
			99	TSVQTEDDQLIAGQSAR	iTRAQ4plex@N-term		0.0	1961.98	655.00	1961.98	655.00	3	10.0	27.9	26.3	73.7	2.8
			99	QALQDLLSEYMGNAGR	iTRAQ4plex@N-term		0.0	1908.91	637.31	1908.95	637.32	3	1.7	8.7	16.3	83.7	5.1
			99	QDLLAYLQR	iTRAQ4plex@N-term; Dimethyl(R)@9		0.0	1290.74	646.38	1290.74	646.38	2	13.6	228.1	5.6	94.4	16.7
			99	WDDSGNDIIVLAK	iTRAQ4plex@N-term; iTRAQ4plex(K)@13		-0.1	1732.87	867.44	1732.92	867.47	2	183.3	490.4	27.2	72.8	2.7
			99	WDDSGNDIIVLAK	iTRAQ4plex@N-term; iTRAQ4plex(K)@13		0.0	1732.89	578.64	1732.92	578.65	3	99.9	320.1	23.8	76.2	3.2
			99	LLEPLVTQVTTLVNTSNK	iTRAQ4plex@N-term; iTRAQ4plex(K)@18		0.1	2257.37	753.46	2257.31	753.45	3	1.7	8.7	16.3	83.7	5.1
			99	LLEPLVTQVTTLVNTSNK	iTRAQ4plex@N-term; iTRAQ4plex(K)@18		0.1	2257.38	753.47	2257.31	753.45	3	1.7	12.0	12.4	87.6	7.0
			99	WDDSGNDIIVLAK	iTRAQ4plex@N-term; Lys->Arg@13		0.1	1616.88	809.45	1616.83	809.42	2	32.8	155.0	17.4	82.6	4.7
			99	WDDSGNDIIVLAK	iTRAQ4plex@N-term; Lys->Gln@13		0.0	1588.83	795.42	1588.79	795.40	2	265.7	439.7	37.7	62.3	1.7
			99	WDDSGNDIIVLAK	iTRAQ4plex@N-term; Lys->Gln@13		0.1	1588.84	795.43	1588.79	795.40	2	187.9	365.6	33.9	66.1	1.9
			99	LESIISGAALMADSSCTR	iTRAQ4plex@N-term; Pyridylethyl(C)@16		0.0	2073.04	692.02	2073.04	692.02	3	17.2	30.8	35.8	64.2	1.8
			99	LESIISGAALMADSSCTR	iTRAQ4plex@N-term; Pyridylethyl(C)@16; Thr->Glu@17		0.0	2101.02	701.35	2101.03	701.35	3	1.7	18.5	8.4	91.6	10.9
			98	ALLSAVTR	iTRAQ4plex@N-term		1.0	974.59	488.30	973.60	487.81	2	1495.3	5895.5	20.2	79.8	3.9
			98	IAEAGSR	iTRAQ4plex@N-term		1.0	847.51	424.76	846.47	424.24	2	66.8	124.1	35.0	65.0	1.9
			98	QDLLAYLQR	iTRAQ4plex@N-term; Gln->Arg@8		0.0	1290.72	646.37	1290.75	646.38	2	41.1	327.7	11.1	88.9	8.0
			92	EYAQVFR	iTRAQ4plex@N-term		0.0	1055.57	528.79	1055.55	528.78	2	471.3	1273.3	27.0	73.0	2.7
			91	ASYVASTK	iTRAQ4plex@N-term; iTRAQ4plex(K)@8		0.0	1113.65	557.83	1113.63	557.82	2	1056.1	2593.3	28.9	71.1	2.5
			89	LLEPLVTQVTTLVNTSNK	iTRAQ4plex@N-term; iTRAQ4plex(S)@17; Lys->Arg@18		0.1	2285.39	762.80	2285.32	762.78	3	1.7	13.0	11.5	88.5	7.7
			88	LLEPLVTQVTTLVNTSNKGPS	iTRAQ4plex@N-term; iTRAQ4plex(K)@18; Ser->Gln@21	cleaved S-N@C-term; missed K-G@18	0.0	2539.42	847.48	2539.45	847.49	3	12.4	10.4	54.5	45.5	0.8
			74	IAEAGSR	iTRAQ4plex@N-term		1.0	847.50	424.76	846.47	424.24	2	142.4	159.1	47.2	52.8	1.1
			70	AIMAQLPQEQK	iTRAQ4plex@N-term; Deamidated(Q)@10; Lys->Arg@11		0.0	1428.78	715.40	1428.75	715.38	2	217.4	759.7	22.3	77.7	3.5
			70	AIMAQLPQEQK	iTRAQ4plex@N-term; Deamidated(Q)@10; Lys->Arg@11		0.0	1428.77	715.39	1428.75	715.38	2	144.1	461.5	23.8	76.2	3.2
			70	AIMAQLPQEQK	iTRAQ4plex@N-term; Deamidated(Q)@10; No iTRAQ4plex(K)@11		0.0	1400.76	701.39	1400.75	701.38	2	354.0	728.6	32.7	67.3	2.1
			70	AIMAQLPQEQK	iTRAQ4plex@N-term; Deamidated(Q)@10; No iTRAQ4plex(K)@11		0.0	1400.76	701.39	1400.75	701.38	2	318.9	587.0	35.2	64.8	1.8
			69	AIMAQLPQEQK	iTRAQ4plex@N-term; Deamidated(Q)@10; Lys->Gln@11		0.0	1400.75	701.38	1400.71	701.36	2	198.3	481.4	29.2	70.8	2.4
			62	DQMAAAR	iTRAQ4plex@N-term; Deamidated(Q)@2		0.1	906.49	454.25	906.44	454.22	2	6.5	23.6	21.5	78.5	3.7
			47	IAEAGSR	iTRAQ4plex@N-term		-2.0	844.50	423.26	846.47	424.24	2					
			43	QQELK	iTRAQ4plex@N-term; iTRAQ4plex(K)@5		0.0	932.59	467.30	932.55	467.28	2					
			33	VLEATK	iTRAQ4plex@N-term; iTRAQ4plex(K)@6		0.0	947.61	474.81	947.59	474.80	2					
			30	QDLLAYLQR	iTRAQ4plex@N-term; Deamidated(Q)@1; Deamidated(Q)@8		0.0	1264.70	633.36	1264.68	633.35	2					
			14	LLEPLVTQVTTLVNTSNK	iTRAQ4plex@N-term; Ser->Asp@17; iTRAQ4plex(K)@18		0.1	2285.37	762.80	2285.31	762.78	3					
			10	LLEPLVTQVTTLVNTSNK	iTRAQ4plex@N-term; Deamidated(Q)@8; Methyl(T)@11; iTRAQ4plex(K)@18		0.0	2272.31	758.44	2272.31	758.45	3					
																Average	4.1
																Stdev	3.3
IPI:IPI00223882.5	CTNNA3	Catenin alpha 3	99	DQDADNLDR	iTRAQ4plex@N-term		0.0	1204.58	603.30	1204.54	603.28	2	29.8	135.6	18.0	82.0	4.5
			99	QALQDLLTEYMSNTGK	iTRAQ4plex@N-term; iTRAQ4plex(K)@16		-1.0	2098.05	700.36	2099.08	700.70	3	6.6	18.2	26.8	73.2	2.7
			99	ELESLDYLAFK	iTRAQ4plex@N-term; Lys->Gln@11		0.0	1470.78	736.40	1470.74	736.38	2	20.6	36.1	36.4	63.6	1.8
			99	QALQDLLTEYMSNTGK	iTRAQ4plex@N-term; Lys->Gln@16		0.1	1955.02	652.68	1954.94	652.65	3	1.7	9.8	14.8	85.2	5.8
			98	ELESLDYLAFK	iTRAQ4plex@N-term; iTRAQ4plex(K)@11		0.0	1614.86	539.29	1614.87	539.30	3	8.4	19.2	30.5	69.5	2.3
			42	DEIAGAR	iTRAQ4plex@N-term		2.1	876.55	439.28	874.46	438.24	2					
			41	DEIAGAR	iTRAQ4plex@N-term		0.1	874.52	438.27	874.46	438.24	2					
																Average	3.4
																Stdev	1.7
IPI:IPI00753025.1	CTNNB1	Catenin beta 1	99	AAMFPETLDEGMQIPSTQFDAAHPTNVQR	iTRAQ4plex@N-term		-0.1	3345.52	837.39	3345.59	837.40	4	37.4	78.7	32.2	67.8	2.1
			99	SPQMVSAIVR	iTRAQ4plex@N-term		-0.1	1230.62	616.32	1230.69	616.35	2	5590.8	17341.9	24.4	75.6	3.1
			99	TMQNTNDVETAR	iTRAQ4plex@N-term		0.0	1522.70	762.36	1522.72	762.37	2	2145.8	7570.5	22.1	77.9	3.5
			99	SPQMVSAIVR	iTRAQ4plex@N-term		-0.1	1230.63	616.32	1230.69	616.35	2	1887.0	6791.9	21.7	78.3	3.6
			99	TMQNTNDVETAR	iTRAQ4plex@N-term		0.0	1522.73	762.37	1522.72	762.37	2	1653.2	6079.0	21.4	78.6	3.7
			99	TMQNTNDVETAR	iTRAQ4plex@N-term		0.0	1522.73	762.37	1522.72	762.37	2	1215.1	4551.0	21.1	78.9	3.7
			99	HQEAEMAQNAVR	iTRAQ4plex@N-term		0.0	1526.78	509.93	1526.74	509.92	3	521.3	1707.5	23.4	76.6	3.3
			99	HAVVNLINYQDDAELATR	iTRAQ4plex@N-term		0.0	2185.12	729.38	2185.12	729.38	3	334.2	469.4	41.6	58.4	1.4
			99	NEGVATYAAAVLFR	iTRAQ4plex@N-term		0.0	1624.87	813.44	1624.87	813.44	2	77.5	232.3	25.0	75.0	3.0
			99	LSVELTSSLFR	iTRAQ4plex@N-term		0.0	1394.81	698.41	1394.79	698.40	2	55.8	175.5	24.1	75.9	3.1
			99	HAVVNLINYQDDAELATR	iTRAQ4plex@N-term		0.0	2185.14	729.39	2185.12	729.38	3	53.8	80.3	40.1	59.9	1.5
			99	AAMFPETLDEGMQIPSTQFDAAHPTNVQR	iTRAQ4plex@N-term		-0.9	3344.68	837.18	3345.59	837.40	4	24.0	44.7	34.9	65.1	1.9
			99	AAMFPETLDEGMQIPSTQFDAAHPTNVQR	iTRAQ4plex@N-term		0.0	3345.59	837.40	3345.59	837.40	4	22.2	43.5	33.8	66.2	2.0
			99	EAAEAIEAEGATAPLTELLHSR	iTRAQ4plex@N-term		0.0	2422.27	808.43	2422.25	808.42	3	17.1	31.8	35.0	65.0	1.9
			99	EAAEAIEAEGATAPLTELLHSR	iTRAQ4plex@N-term		0.0	2422.29	808.44	2422.25	808.42	3	15.3	32.0	32.4	67.6	2.1
			99	AAMFPETLDEGMQIPSTQFDAAHPTNVQR	iTRAQ4plex@N-term		-0.1	3345.50	837.38	3345.59	837.40	4	8.3	23.4	26.1	73.9	2.8
			99	LIILASGGPQALVNIMR	iTRAQ4plex@N-term		0.0	1909.15	637.39	1909.13	637.38	3	5.1	11.8	30.1	69.9	2.3
			99	LIILASGGPQALVNIMR	iTRAQ4plex@N-term		0.0	1909.16	637.39	1909.13	637.38	3	1.7	13.0	11.5	88.5	7.7
			99	NEGVATYAAAVLFR	iTRAQ4plex@N-term		0.0	1624.89	542.64	1624.87	542.63	3	1.7	71.6	2.3	97.7	42.1
			99	AAMFPETLDEGMQIPSTQFDAAHPTNVQR	iTRAQ4plex@N-term; Deamidated(Q)@18; Gln->Arg@28		0.0	3374.63	844.67	3374.61	844.66	4	4.7	22.6	17.1	82.9	4.9
			99	TMQNTNDVETAR	iTRAQ4plex@N-term; Dimethyl(R)@12		0.0	1550.74	776.37	1550.75	776.38	2	165.9	1966.3	7.8	92.2	11.8
			99	NEGVATYAAAVLFR	iTRAQ4plex@N-term; Dimethyl(R)@14		0.0	1652.94	551.99	1652.90	551.97	3	1.7	11.8	12.6	87.4	7.0
			99	AAMFPETLDEGMQIPSTQFDAAHPTNVQR	iTRAQ4plex@N-term; Glu->Gln@10; Gln->Arg@28		-0.1	3372.59	844.15	3372.65	844.17	4	1.7	37.0	4.4	95.6	21.7
			99	LLNDEDQVVVNK	iTRAQ4plex@N-term; iTRAQ4plex(K)@12		0.0	1672.90	558.64	1672.92	558.65	3	157.2	303.9	34.1	65.9	1.9
			99	NLSDAATK	iTRAQ4plex@N-term; iTRAQ4plex(K)@8		0.0	1106.66	554.34	1106.62	554.32	2	804.1	2679.7	23.1	76.9	3.3
			99	NLSDAATK	iTRAQ4plex@N-term; iTRAQ4plex(K)@8		0.0	1106.65	554.33	1106.62	554.32	2	495.1	2722.2	15.4	84.6	5.5
			99	EGLLAIFK	iTRAQ4plex@N-term; iTRAQ4plex(K)@8		0.0	1177.73	589.87	1177.73	589.87	2	57.2	286.0	16.7	83.3	5.0
			99	LAEPSQMLK	iTRAQ4plex@N-term; iTRAQ4plex(K)@9		0.0	1303.75	652.88	1303.74	652.88	2	1902.2	6275.6	23.3	76.7	3.3
			99	SGGIPALVK	iTRAQ4plex@N-term; iTRAQ4plex(K)@9		2.0	1130.71	566.36	1128.71	565.36	2	423.7	1623.2	20.7	79.3	3.8
			99	AAVMVHQLSK	iTRAQ4plex@N-term; Lys->Gln@10		0.0	1226.68	409.90	1226.66	409.89	3	197.9	392.3	33.5	66.5	2.0
			99	LHYGLPVVVK	iTRAQ4plex@N-term; Lys->Gln@10		0.0	1267.76	423.60	1267.74	423.59	3	139.7	133.5	51.1	48.9	1.0
			99	LLNDEDQVVVNK	iTRAQ4plex@N-term; Lys->Gln@12		0.0	1528.79	765.40	1528.79	765.40	2	376.0	2177.7	14.7	85.3	5.8
			99	LLNDEDQVVVNK	iTRAQ4plex@N-term; Lys->Gln@12		0.0	1528.83	765.42	1528.79	765.40	2	198.3	483.2	29.1	70.9	2.4
			99	AAMFPETLDEGMQIPSTQFDAAHPTNVQR	iTRAQ4plex@N-term; Oxidation(M)@12; Deamidated(Q)@13		0.0	3362.60	841.66	3362.57	841.65	4	28.0	35.9	43.8	56.2	1.3
			99	HQEAEMAQNAVR	iTRAQ4plex@N-term; Oxidation(M)@6		0.1	1542.79	515.27	1542.73	515.25	3	1.7	58.7	2.8	97.2	34.5
			98	EGLLAIFK	iTRAQ4plex@N-term; iTRAQ4plex(K)@8		0.0	1177.72	589.87	1177.73	589.87	2	107.0	577.0	15.6	84.4	5.4
			98	LAEPSQMLK	iTRAQ4plex@N-term; Lys->Gln@9		0.0	1159.61	580.81	1159.60	580.81	2	1960.9	5073.2	27.9	72.1	2.6
			96	SPQMVSAIVR	iTRAQ4plex@N-term; Dimethyl(R)@10		0.0	1258.72	630.37	1258.72	630.37	2	194.1	1495.8	11.5	88.5	7.7
			95	AIPELTK	iTRAQ4plex@N-term; iTRAQ4plex(K)@7		0.0	1058.63	530.32	1058.66	530.34	2	1555.7	5245.0	22.9	77.1	3.4
			95	EGLLAIFK	iTRAQ4plex@N-term; Lys->Gln@8		0.0	1033.61	517.81	1033.59	517.80	2	89.9	343.4	20.7	79.3	3.8
			93	NLSDAATK	iTRAQ4plex@N-term; Lys->Gln@8		0.1	962.54	482.28	962.48	482.25	2	16.3	54.5	23.0	77.0	3.3
			86	LVQLLVR	iTRAQ4plex@N-term		0.0	983.64	492.83	983.66	492.84	2	353.2	1150.1	23.5	76.5	3.3
			83	NLSDAATK	iTRAQ4plex@N-term; Lys->Gln@8		0.1	962.57	482.29	962.48	482.25	2	14.0	67.7	17.1	82.9	4.8
			82	LVQLLVR	iTRAQ4plex@N-term		0.0	983.65	492.83	983.66	492.84	2	361.9	1109.5	24.6	75.4	3.1

			76	LAEPSQMLK	iTRAQ4plex@N-term; Lys->Arg@9		0.0	1187.60	594.81	1187.65	594.83	2	118.2	469.8	20.1	79.9	4.0
			66	NEGVATYAAAVLFR	iTRAQ4plex@N-term; Ala->Val@10		0.0	1652.91	827.46	1652.90	827.46	2	10.5	64.5	14.0	86.0	6.2
			65	EQGAIPR	iTRAQ4plex@N-term		0.0	913.54	457.78	913.51	457.76	2	743.8	3227.2	18.7	81.3	4.3
			65	EGLLAIFK	iTRAQ4plex@N-term; iTRAQ4plex(K)@8		-3.1	1174.58	588.30	1177.73	589.87	2	173.6	756.3	18.7	81.3	4.4
			50	EGLLAIFK	iTRAQ4plex@N-term; Lys->Gln@8		0.1	1033.65	517.83	1033.59	517.80	2	21.9	51.3	29.9	70.1	2.3
			47	NLSDAATK	iTRAQ4plex@N-term; Lys->Gln@8		0.1	962.55	482.28	962.48	482.25	2					
			36	NLSDAATK	iTRAQ4plex@N-term; Lys->Gln@8		0.1	962.53	482.27	962.48	482.25	2					
			35	EQGAIPR	iTRAQ4plex@N-term		0.0	913.53	457.77	913.51	457.76	2					
			28	TMQNTNDVETAR	iTRAQ4plex@N-term; Oxidation(M)@2		0.1	1538.77	770.39	1538.71	770.36	2					
			23	AAMFPETLDEGMQIPSTQFDAAHPTNVQR	iTRAQ4plex@N-term; Dimethyl(R)@29		0.0	3373.61	844.41	3373.62	844.41	4					
			22	MVALLNK	iTRAQ4plex@N-term; iTRAQ4plex(K)@7		0.0	1075.67	538.84	1075.67	538.84	2					
			15	AIPELTK	iTRAQ4plex@N-term; Lys->Gln@7		0.0	914.56	458.29	914.52	458.27	2					
																Average	5.5
																Stdev	7.6
IPI:IPI00136135.2	CTNND2	Catenin delta 2	99	AASQVLNSMWQYR	iTRAQ4plex@N-term		0.0	1696.86	849.44	1696.85	849.43	2	182.2	1073.2	14.5	85.5	5.9
			99	SGPALPPEGTLAR	iTRAQ4plex@N-term		0.0	1408.78	705.40	1408.78	705.40	2	1977.2	5826.4	25.3	74.7	2.9
			99	TSTAPSSPGVDSVPLQR	iTRAQ4plex@N-term		0.0	1841.93	614.98	1841.96	614.99	3	478.1	486.2	49.6	50.4	1.0
			99	DGWSQYHFVASSSTIER	iTRAQ4plex@N-term		-0.1	2112.94	705.32	2113.00	705.34	3	259.7	453.8	36.4	63.6	1.7
			99	EQELQFER	iTRAQ4plex@N-term		0.0	1221.63	611.82	1221.61	611.81	2	144.8	444.0	24.6	75.4	3.1
			99	ASYAAGPASNYADPYR	iTRAQ4plex@N-term		0.0	1816.87	909.44	1816.85	909.43	2	113.5	1049.8	9.8	90.2	9.3
			99	DGWSQYHFVASSSTIER	iTRAQ4plex@N-term		0.0	2113.01	705.34	2113.00	705.34	3	102.5	348.9	22.7	77.3	3.4
			99	DYETYQPFPNSTR	iTRAQ4plex@N-term		0.0	1760.83	881.42	1760.81	881.41	2	94.0	481.7	16.3	83.7	5.1
			99	NVSSAGEEAR	iTRAQ4plex@N-term		0.0	1162.58	582.30	1162.57	582.29	2	92.1	632.7	12.7	87.3	6.9
			99	IQLHSSQVLR	iTRAQ4plex@N-term		0.0	1323.77	442.27	1323.77	442.27	3	69.6	148.0	32.0	68.0	2.1
			99	ASYAAGPASNYADPYR	iTRAQ4plex@N-term		0.0	1816.87	606.63	1816.85	606.62	3	64.5	236.7	21.4	78.6	3.7
			99	TSTAPSSPGVDSVPLQR	iTRAQ4plex@N-term		1.0	1842.99	615.34	1841.96	614.99	3	62.6	140.4	30.8	69.2	2.2
			99	NVSSAGEEAR	iTRAQ4plex@N-term		0.0	1162.59	582.30	1162.57	582.29	2	48.6	371.8	11.6	88.4	7.6
			99	NVSSAGEEAR	iTRAQ4plex@N-term		0.0	1162.62	582.32	1162.57	582.29	2	43.8	203.4	17.7	82.3	4.6
			99	SLSQSQGDPLPPAHTGTFR	iTRAQ4plex@N-term		0.0	2139.11	714.04	2139.08	714.03	3	37.7	169.1	18.2	81.8	4.5
			99	NVSSAGEEAR	iTRAQ4plex@N-term		1.1	1163.62	582.82	1162.57	582.29	2	22.7	129.2	15.0	85.0	5.7
			99	AASQVLNSMWQYR	iTRAQ4plex@N-term		0.0	1696.84	566.62	1696.85	566.62	3	22.1	96.6	18.6	81.4	4.4
			99	SASAPASPR	iTRAQ4plex@N-term		2.1	988.59	495.30	986.53	494.27	2	20.0	154.0	11.5	88.5	7.7
			99	NVSSAGEEAR	iTRAQ4plex@N-term		0.0	1162.61	582.31	1162.57	582.29	2	15.4	179.2	7.9	92.1	11.6
			99	DYETYQPFPNSTR	iTRAQ4plex@N-term		0.0	1760.82	587.95	1760.81	587.94	3	11.2	43.0	20.7	79.3	3.8
			99	NVSSAGEEAR	iTRAQ4plex@N-term		0.0	1162.62	582.32	1162.57	582.29	2	7.7	89.8	7.9	92.1	11.7
			99	DTMTAQNTGVSTLYR	iTRAQ4plex@N-term		0.0	1800.87	601.30	1800.88	601.30	3	6.5	23.6	21.5	78.5	3.7
			99	GLPILVELLR	iTRAQ4plex@N-term		0.0	1265.84	633.93	1265.82	633.92	2	1.7	13.0	11.5	88.5	7.7
			99	GWAEDVAGMAYALR	iTRAQ4plex@N-term		0.0	1652.81	551.94	1652.81	551.94	3	1.7	7.6	18.3	81.7	4.5
			99	QGGIQLLVDLLDHR	iTRAQ4plex@N-term		0.0	1719.98	574.34	1719.98	574.33	3	1.7	8.7	16.3	83.7	5.1
			99	NVSSAGEEAR	iTRAQ4plex@N-term; Ala->Val@9		0.0	1190.60	596.31	1190.60	596.31	2	455.5	9554.4	4.6	95.4	21.0
			99	AASQVLNSMWQYR	iTRAQ4plex@N-term; Dimethyl(R)@13		0.0	1724.87	575.96	1724.88	575.97	3	1.7	15.2	10.0	90.0	9.0
			99	DGWSQYHFVASSSTIER	iTRAQ4plex@N-term; Dimethyl(R)@17		0.0	2140.99	714.67	2141.03	714.68	3	38.8	90.6	30.0	70.0	2.3
			99	TSTAPSSPGVDSVPLQR	iTRAQ4plex@N-term; Gln->Arg@16		0.0	1870.00	624.34	1870.00	624.34	3	34.5	56.1	38.1	61.9	1.6
			99	TSTAPSSPGVDSVPLQR	iTRAQ4plex@N-term; Gln->Arg@16		0.0	1869.98	624.33	1870.00	624.34	3	11.4	39.6	22.3	77.7	3.5
			99	SYSTSSPINIVVSSAGLSPIR	iTRAQ4plex@N-term; HexNAc(N)@9		0.1	2481.36	828.13	2481.31	828.11	3	1.7	30.4	5.3	94.7	17.9
			99	NSYGAPAEDIK	iTRAQ4plex@N-term; Lys->Gln@11		0.0	1307.64	654.83	1307.61	654.81	2	110.9	618.5	15.2	84.8	5.6
			99	ELVTGVLWNLSSCDALK	iTRAQ4plex@N-term; Pyridylethyl(C)@13; iTRAQ4plex(K)@17		0.1	2240.28	747.77	2240.21	747.74	3	10.5	62.6	14.4	85.6	5.9
			99	ELVTGVLWNLSSCDALK	iTRAQ4plex@N-term; Pyridylethyl(C)@13; Lys->Arg@17		0.0	2124.12	709.05	2124.12	709.05	3	7.6	43.1	14.9	85.1	5.7
			99	ELVTGVLWNLSSCDALK	iTRAQ4plex@N-term; Pyridylethyl(C)@13; Lys->Gln@17		0.1	2096.13	699.72	2096.07	699.70	3	11.1	46.3	19.4	80.6	4.2
			99	ELVTGVLWNLSSCDALK	iTRAQ4plex@N-term; Pyridylethyl(C)@13; Lys->Gln@17		0.0	2096.11	699.71	2096.07	699.70	3	9.6	37.6	20.3	79.7	3.9
			99	ELVTGVLWNLSSCDALK	iTRAQ4plex@N-term; Pyridylethyl(C)@13; Lys->Gln@17		0.0	2096.11	699.71	2096.07	699.70	3	9.2	49.6	15.6	84.4	5.4
			99	SQDQWDGVGPLPDCAEPPK	iTRAQ4plex@N-term; Pyridylethyl(C)@14; Lys->Gln@19		0.0	2287.03	763.35	2287.03	763.35	3	47.6	148.6	24.3	75.7	3.1
			99	DIEDELTTGLELVDSCIR	iTRAQ4plex@N-term; Pyridylethyl(C)@16		0.0	2269.14	757.39	2269.13	757.38	3	6.4	25.8	19.8	80.2	4.0
			99	DIEDELTTGLELVDSCIR	iTRAQ4plex@N-term; Pyridylethyl(C)@16		0.1	2269.19	757.40	2269.13	757.38	3	1.7	15.2	10.0	90.0	9.0
			98	TPSISPVR	iTRAQ4plex@N-term		0.0	999.59	500.80	999.58	500.80	2	327.6	1998.2	14.1	85.9	6.1
			97	NMENAK	iTRAQ4plex@N-term; iTRAQ4plex(K)@6		0.0	993.56	497.79	993.52	497.77	2	18.5	245.3	7.0	93.0	13.3
			96	DYETYQPFPNSTR	iTRAQ4plex@N-term		0.0	1760.77	587.93	1760.81	587.94	3	64.9	126.9	33.8	66.2	2.0
			94	NVSSAGEEAR	iTRAQ4plex@N-term		4.0	1166.61	584.31	1162.57	582.29	2	32.0	226.7	12.4	87.6	7.1
			93	DAGGIEK	iTRAQ4plex@N-term; iTRAQ4plex(K)@7		0.0	976.55	489.28	976.54	489.28	2	528.8	5451.8	8.8	91.2	10.3
			90	AASQVLNSMWQYR	iTRAQ4plex@N-term		4.0	1700.86	567.96	1696.85	566.62	3	33.3	89.5	27.1	72.9	2.7
			70	SASAPASPR	iTRAQ4plex@N-term		0.1	986.58	494.30	986.53	494.27	2	8.2	26.8	23.3	76.7	3.3
			66	GLPILVELLR	iTRAQ4plex@N-term		0.0	1265.86	633.94	1265.82	633.92	2	1.7	21.7	7.3	92.7	12.8
			61	NMENAK	iTRAQ4plex@N-term; iTRAQ4plex(K)@6		0.0	993.52	497.77	993.52	497.77	2	61.3	675.2	8.3	91.7	11.0
			48	DYETYQPFPNSTR	iTRAQ4plex@N-term; Thr->Glu@12		0.0	1788.83	597.28	1788.81	597.28	3					
			46	ELEAER	iTRAQ4plex@N-term		0.1	889.51	445.76	889.46	445.74	2					
			25	NLVYGK	iTRAQ4plex@N-term; iTRAQ4plex(K)@6		0.0	980.58	491.30	980.59	491.30	2					
			20	ELEAER	iTRAQ4plex@N-term		0.1	889.52	445.77	889.46	445.74	2					
			15	ELEAER	iTRAQ4plex@N-term		0.1	889.52	445.77	889.46	445.74	2					
			12	DPELPEVIQMLQH	iTRAQ4plex@N-term	cleaved H-Q@C-term	0.0	1691.91	564.98	1691.87	564.96	3					
																Average	6.1
																Stdev	4.2
IPI:IPI00757327.1	PKP4	Plakophilin 4	99	AAAQVLNTLWQYR	iTRAQ4plex@N-term		0.0	1676.89	839.45	1676.91	839.46	2	16.9	139.2	10.8	89.2	8.3
			99	AAAQVLNTLWQYR	iTRAQ4plex@N-term		0.0	1676.92	559.98	1676.91	559.98	3	1.7	22.7	7.0	93.0	13.3
			99	GLPILVELLR	iTRAQ4plex@N-term		0.0	1265.84	633.93	1265.82	633.92	2	1.7	13.0	11.5	88.5	7.7
			99	TPQEDQWDGVGPIPGLSK	iTRAQ4plex@N-term; iTRAQ4plex(K)@18		0.0	2211.15	738.06	2211.14	738.05	3	25.5	52.0	32.9	67.1	2.0
			99	LLGLNELDDLLGK	iTRAQ4plex@N-term; Lys->Gln@13		0.0	1555.90	778.96	1555.86	778.94	2	1.7	46.7	3.5	96.5	27.5
			99	LLGLNELDDLLGK	iTRAQ4plex@N-term; Lys->Gln@13		0.1	1555.92	778.97	1555.86	778.94	2	1.7	44.6	3.7	96.3	26.2
			99	TPQEDQWDGVGPIPGLSK	iTRAQ4plex@N-term; Lys->Gln@18		0.0	2067.01	690.01	2067.00	690.01	3	27.6	44.6	38.3	61.7	1.6
			98	TPQEDQWDGVGPIPGLSK	iTRAQ4plex@N-term; Lys->Gln@18		0.0	2067.03	690.02	2067.00	690.01	3	18.8	37.3	33.5	66.5	2.0
			97	NMENAK	iTRAQ4plex@N-term; iTRAQ4plex(K)@6		0.0	993.56	497.79	993.52	497.77	2	18.5	245.3	7.0	93.0	13.3
			90	NLSSAGEEAR	iTRAQ4plex@N-term; Ala->Val@9		0.0	1204.63	603.32	1204.62	603.32	2	91.2	508.9	15.2	84.8	5.6
			66	GLPILVELLR	iTRAQ4plex@N-term		0.0	1265.86	633.94	1265.82	633.92	2	1.7	21.7	7.3	92.7	12.8
			63	EQELQFQR	iTRAQ4plex@N-term; Deamidated(Q)@7		0.0	1221.63	611.82	1221.61	611.81	2	144.8	444.0	24.6	75.4	3.1
			61	NMENAK	iTRAQ4plex@N-term; iTRAQ4plex(K)@6		0.0	993.52	497.77	993.52	497.77	2	61.3	675.2	8.3	91.7	11.0
			24	AAAQVLNTLWQYR	iTRAQ4plex@N-term; Dimethyl(R)@13		0.0	1704.94	853.48	1704.94	853.48	2					
			12	DLVNR	iTRAQ4plex@N-term		0.0	759.47	380.74	759.44	380.73	2					
			12	NLVFGK	iTRAQ4plex@N-term; Oxidation(F)@4; iTRAQ4plex(K)@6		0.0	980.58	491.30	980.59	491.30	2					
																Average	10.3
																Stdev	8.5
IPI:IPI00749860.1	CDH2	Cadherin 2	99	DWVIPPINLPENSR	iTRAQ4plex@N-term		0.0	1792.97	598.66	1792.96	598.66	3	24.6	27.3	47.4	52.6	1.1
			99	QLLIDPEDDVR	iTRAQ4plex@N-term		0.0	1455.77	728.89	1455.77	728.89	2	297.1	1283.6	18.8	81.2	4.3
			99	QLLIDPEDDVR	iTRAQ4plex@N-term		0.0	1455.75	728.88	1455.77	728.89	2	214.6	787.2	21.4	78.6	3.7
			99	IDPVNGQITTIAVLDR	iTRAQ4plex@N-term		0.0	1868.02	623.68	1868.05	623.69	3	55.5	134.2	29.2	70.8	2.4
			99	QEEGLHAGTMLTTLTAQDPDR	iTRAQ4plex@N-term		0.0	2427.15	810.06	2427.18	810.07	3	40.3	98.1	29.1	70.9	2.4
			99	IDPVNGQITTIAVLDR	iTRAQ4plex@N-term		-1.0	1867.03	623.35	1868.05	623.69	3	30.1	76.9	28.2	71.8	2.6
			99	FAILTDPNSNDGLVTVVKPIDFETNR	iTRAQ4plex@N-term; iTRAQ4plex(K)@18		0.0	3162.70	791.68	3162.68	791.68	4	9.4	44.2	17.5	82.5	4.7
			99	FLEAGIYEVPIIITDSGNPPK	iTRAQ4plex@N-term; iTRAQ4plex(K)@21		-1.0	2559.44	854.15	2560.40	854.48	3	1.7	6.5	20.7	79.3	3.8
			99	SAAPHPGDIGDFINEGLK	iTRAQ4plex@N-term; Lys->Gln@18		0.0	1980.95	661.32	1980.97	661.33	3	125.7	266.2	32.1	67.9	2.1
			94	YMQQNIR	iTRAQ4plex@N-term		0.0	1095.56	548.79	1095.56	548.79	2	2189.0	5588.6	28.1	71.9	2.6
			54	ESPNVK	iTRAQ4plex@N-term; iTRAQ4plex(K)@6		0.0	960.58	481.30	960.55	481.28	2	35.2	87.6	28.7	71.3	2.5
																Average	2.9
																Stdev	1.1
IPI:IPI00857932.1	CDH8	Cadherin 8	99	FAQSLYHFSVPEDVVLGTAIGR	iTRAQ4plex@N-term		0.0	2549.35	850.79	2549.34	850.79	3	13.8	23.4	37.1	62.9	1.7
			99	ITLATPLDR	iTRAQ4plex@N-term		0.0	1142.65	572.33	1142.68	572.35	2	173.3	1813.7	8.7	91.3	10.5
			99	DPDITSSPIR	iTRAQ4plex@N-term		0.0	1243.67	622.84	1243.65	622.83	2	133.9	992.5	11.9	88.1	7.4
			99	DPDITSSPIR	iTRAQ4plex@N-term		0.0	1243.66	622.84	1243.65	622.83	2	121.5	980.2	11.0	89.0	8.1
			99	VEAANIHIDPR	iTRAQ4plex@N-term		0.0	1377.72	460.25	1377.75	460.26	3	66.0	246.5	21.1	78.9	3.7
			10	QFNINADDGK	iTRAQ4plex@N-term; No iTRAQ4plex(K)@10		0.0	1264.65	633.33	1264.62	633.32	2					
																Average	6.3
																Stdev	3.5
IPI:IPI00670856.3	CDH10	Cadherin 10	99	ATDADTGK	iTRAQ4plex@N-term; iTRAQ4plex(K)@8		0.1	1065.64	533.83	1065.55	533.78	2	9.4	43.1	17.9	82.1	4.6
			99	DIIPETLFIPR	iTRAQ4plex@N-term		0.0	1456.82	729.42	1456.84	729.43	2	92.8	215.4	30.1	69.9	2.3
			99	DIIPETLFIPR	iTRAQ4plex@N-term		0.0	1456.83	486.62	1456.84	486.62	3	6.9	12.8	34.9	65.1	1.9
			99	DIIPETLFIPR	iTRAQ4plex@N-term		-1.0	1455.87	486.30	1456.84	486.62	3	1.7	6.5	20.7	79.3	3.8
			99	ISIEDVDEPPVFSR	iTRAQ4plex@N-term		0.0	1745.92	582.98	1745.90	582.97	3	1.7	25.0	6.4	93.6	14.7
			99	IIDGDGTDMFDIITEK	iTRAQ4plex@N-term; iTRAQ4plex(K)@16		0.0	2070.06	691.03	2070.04	691.02	3	5.0	12.8	28.1	71.9	2.6
			99	IIDGDGTDMFDIITEK	iTRAQ4plex@N-term; iTRAQ4plex(K)@16		-1.0	2069.05	690.69	2070.04	691.02	3	4.8	18.3	20.9	79.1	3.8
			86	EQYQVVIQAK	iTRAQ4plex@N-term; iTRAQ4plex(K)@10		0.0	1492.84	498.62	1492.85	498.62	3	107.7	208.2	34.1	65.9	1.9
			39	DIIPETLFIPR	iTRAQ4plex@N-term; Dimethyl(R)@11		0.0	1484.91	495.98	1484.87	495.96	3					
																Average	4.4
																Stdev	4.3
IPI:IPI00138190.1	CDH11	Cadherin 11	99	AQYTLMAQAVDR	iTRAQ4plex@N-term		0.0	1509.75	504.26	1509.77	504.26	3	6.5	21.5	23.3	76.7	3.3
			99	DNTAGVYAR	iTRAQ4plex@N-term		0.0	1109.60	555.81	1109.56	555.79	2	192.5	1241.7	13.4	86.6	6.5
			99	FVIEEYTGPDPVLVGR	iTRAQ4plex@N-term	cleaved F-F@N-term	0.0	1934.03	645.69	1934.03	645.68	3	1.7	9.8	14.8	85.2	5.8
			99	ISVEDADEPPMFLAPSYIHEVQENAAAGTVVGI	iTRAQ4plex@N-term		0.1	3655.89	914.98	3655.80	914.96	4	1.7	6.5	20.7	79.3	3.8
			99	ISVEDADEPPMFLAPSYIHEVQENAAAGTVVGI	iTRAQ4plex@N-term		2.0	3657.83	915.46	3655.80	914.96	4	1.7	9.8	14.8	85.2	5.8
			99	FFTINPEDGFIK	iTRAQ4plex@N-term; iTRAQ4plex(K)@12		0.0	1714.88	572.63	1714.92	572.65	3	22.5	236.4	8.7	91.3	10.5
			99	DTNRPLEPPSEFIVK	iTRAQ4plex@N-term; iTRAQ4plex(K)@15		0.0	2029.13	677.38	2029.11	677.38	3	77.6	378.2	17.0	83.0	4.9
			99	FFTINPEDGFIK	iTRAQ4plex@N-term; No iTRAQ4plex(K)@12		0.0	1570.84	786.43	1570.82	786.41	2	212.7	488.0	30.4	69.6	2.3
			98	DPDAANSPIR	iTRAQ4plex@N-term		0.0	1198.62	600.32	1198.61	600.31	2	619.0	4087.2	13.2	86.8	6.6
			98	FFTINPEDGFIK	iTRAQ4plex@N-term; Lys->Arg@12		0.0	1598.84	800.43	1598.82	800.42	2	1.4	112.6	1.2	98.8	82.8
			93	FFTINPEDGFIK	iTRAQ4plex@N-term; Lys->Gln@12		0.1	1570.85	786.43	1570.78	786.40	2	131.6	303.8	30.2	69.8	2.3
			51	FFTINPEDGFIK	iTRAQ4plex@N-term; Lys->Arg@12		0.1	1598.89	800.45	1598.82	800.42	2	8.1	79.9	9.2	90.8	9.9
			49	DPDAANSPIR	iTRAQ4plex@N-term		0.0	1198.65	600.33	1198.61	600.31	2					
			18	VPVAIR	iTRAQ4plex@N-term		0.0	797.51	399.76	797.52	399.77	2					
																Average	12.0
																Stdev	22.4
IPI:IPI00133903.1	HSPA9	Heat shock protein 9	99	AMQDAEVSK	iTRAQ4plex@N-term; No iTRAQ4plex(K)@9		0.1	1121.61	561.81	1121.55	561.78	2	856.6	494.2	63.4	36.6	0.6
			99	VLENAEGAR	iTRAQ4plex@N-term		0.0	1101.58	551.80	1101.59	551.80	2	18823.0	13141.0	58.9	41.1	0.7
			99	TTPSVVAFTADGER	iTRAQ4plex@N-term		0.0	1593.77	797.89	1593.81	797.91	2	2732.9	1377.4	66.5	33.5	0.5
			99	VQQTVQDLFGR	iTRAQ4plex@N-term		0.0	1433.79	717.90	1433.77	717.89	2	2333.3	1296.0	64.3	35.7	0.6
			99	DAGQISGLNVLR	iTRAQ4plex@N-term		0.0	1385.74	693.88	1385.77	693.89	2	2200.1	1186.9	65.0	35.0	0.5
			99	DAGQISGLNVLR	iTRAQ4plex@N-term		0.0	1385.74	693.88	1385.77	693.89	2	1470.4	774.8	65.5	34.5	0.5
			99	DAGQISGLNVLR	iTRAQ4plex@N-term		0.0	1385.74	693.88	1385.77	693.89	2	1074.6	539.1	66.6	33.4	0.5
			99	VQQTVQDLFGR	iTRAQ4plex@N-term		0.0	1433.78	717.90	1433.77	717.89	2	861.6	557.0	60.7	39.3	0.6
			99	DAGQISGLNVLR	iTRAQ4plex@N-term		2.0	1387.78	694.90	1385.77	693.89	2	703.3	384.0	64.7	35.3	0.5
			99	SDIGEVILVGGMTR	iTRAQ4plex@N-term		0.0	1589.85	795.93	1589.86	795.94	2	388.4	235.7	62.2	37.8	0.6
			99	TTPSVVAFTADGER	iTRAQ4plex@N-term		0.0	1593.77	532.26	1593.81	532.28	3	153.3	109.9	58.2	41.8	0.7
			99	NAVITVPAYFNDSQR	iTRAQ4plex@N-term		0.0	1837.95	613.66	1837.94	613.66	3	105.8	59.0	64.2	35.8	0.6
			99	NAVITVPAYFNDSQR	iTRAQ4plex@N-term		0.0	1837.90	613.64	1837.94	613.66	3	60.8	39.9	60.4	39.6	0.7
			99	STNGDTFLGGEDFDQALLR	iTRAQ4plex@N-term		0.0	2199.07	734.03	2199.06	734.03	3	34.5	108.4	24.1	75.9	3.1
			99	STNGDTFLGGEDFDQALLR	iTRAQ4plex@N-term		0.0	2199.10	734.04	2199.06	734.03	3	13.7	25.5	34.9	65.1	1.9
			99	SDIGEVILVGGMTR	iTRAQ4plex@N-term		0.0	1589.87	530.96	1589.86	530.96	3	10.6	11.5	47.8	52.2	1.1
			99	STNGDTFLGGEDFDQALLR	iTRAQ4plex@N-term; Dimethyl(R)@19		0.0	2227.06	743.36	2227.09	743.37	3	12.2	18.0	40.3	59.7	1.5
			99	ETAENYLGHTAK	iTRAQ4plex@N-term; iTRAQ4plex(K)@12		0.0	1620.83	541.28	1620.84	541.29	3	334.1	370.8	47.4	52.6	1.1
			99	QAASSLQQASLK	iTRAQ4plex@N-term; iTRAQ4plex(K)@12		2.0	1520.89	507.97	1518.86	507.29	3	297.3	274.6	52.0	48.0	0.9
			99	AQFEGIVTDLIK	iTRAQ4plex@N-term; iTRAQ4plex(K)@12		0.0	1620.96	541.33	1620.93	541.32	3	21.4	14.4	59.8	40.2	0.7
			99	QAVTNPNNTFYATK	iTRAQ4plex@N-term; iTRAQ4plex(K)@14		0.0	1855.97	619.66	1855.97	619.66	3	577.5	571.9	50.2	49.8	1.0
			99	LYSPSQIGAFVLMK	iTRAQ4plex@N-term; iTRAQ4plex(K)@14		0.0	1841.07	614.70	1841.04	614.69	3	75.0	50.4	59.8	40.2	0.7
			99	VINEPTAAALAYGLDK	iTRAQ4plex@N-term; iTRAQ4plex(K)@16		0.0	1933.09	645.37	1933.08	645.37	3	94.8	108.1	46.7	53.3	1.1
			99	VINEPTAAALAYGLDK	iTRAQ4plex@N-term; iTRAQ4plex(K)@16		0.0	1933.08	645.37	1933.08	645.37	3	92.1	84.3	52.2	47.8	0.9
			99	VEAVNMAEGIIHDTETK	iTRAQ4plex@N-term; iTRAQ4plex(K)@17		0.0	2144.05	715.69	2144.10	715.71	3	262.2	184.0	58.8	41.2	0.7
			99	ETGVDLTK	iTRAQ4plex@N-term; iTRAQ4plex(K)@8		0.0	1149.66	575.84	1149.65	575.83	2	2119.6	1783.8	54.3	45.7	0.8
			99	AQFEGIVTDLIK	iTRAQ4plex@N-term; Lys->Arg@12		0.0	1504.86	753.44	1504.84	753.43	2	35.6	76.8	31.7	68.3	2.2
			99	AQFEGIVTDLIK	iTRAQ4plex@N-term; Lys->Arg@12		0.1	1504.90	753.46	1504.84	753.43	2	28.4	74.8	27.5	72.5	2.6
			99	AQFEGIVTDLIK	iTRAQ4plex@N-term; Lys->Arg@12		0.1	1504.90	753.46	1504.84	753.43	2	25.1	63.1	28.5	71.5	2.5
			99	LYSPSQIGAFVLMK	iTRAQ4plex@N-term; Lys->Arg@14		0.0	1724.98	576.00	1724.94	575.99	3	1.7	5.4	23.8	76.2	3.2
			99	VEAVNMAEGIIHDTETK	iTRAQ4plex@N-term; Lys->Arg@17		0.0	2028.03	677.02	2028.01	677.01	3	24.2	38.2	38.8	61.2	1.6
			99	VEAVNMAEGIIHDTETK	iTRAQ4plex@N-term; Lys->Arg@17		0.0	2028.04	677.02	2028.01	677.01	3	17.0	36.3	31.9	68.1	2.1
			99	QAASSLQQASLK	iTRAQ4plex@N-term; Lys->Gln@12		0.0	1374.75	688.38	1374.72	688.37	2	848.1	376.6	69.3	30.7	0.4
			99	AQFEGIVTDLIK	iTRAQ4plex@N-term; Lys->Gln@12		0.1	1476.85	739.43	1476.79	739.40	2	286.1	183.1	61.0	39.0	0.6
			99	AQFEGIVTDLIK	iTRAQ4plex@N-term; Lys->Gln@12		0.1	1476.87	739.44	1476.79	739.40	2	110.4	83.6	56.9	43.1	0.8
			99	QAVTNPNNTFYATK	iTRAQ4plex@N-term; Lys->Gln@14		0.1	1711.89	856.95	1711.83	856.92	2	1213.2	552.5	68.7	31.3	0.5
			99	QAVTNPNNTFYATK	iTRAQ4plex@N-term; Lys->Gln@14		0.0	1711.84	856.93	1711.83	856.92	2	580.5	286.0	67.0	33.0	0.5
			99	LYSPSQIGAFVLMK	iTRAQ4plex@N-term; Lys->Gln@14		0.0	1696.93	566.65	1696.90	566.64	3	12.5	9.3	57.3	42.7	0.7
			99	VINEPTAAALAYGLDK	iTRAQ4plex@N-term; Lys->Gln@16		0.0	1788.97	597.33	1788.94	597.32	3	50.8	62.9	44.7	55.3	1.2
			99	VINEPTAAALAYGLDK	iTRAQ4plex@N-term; Lys->Gln@16		0.0	1788.97	597.33	1788.94	597.32	3	13.8	23.4	37.1	62.9	1.7
			99	VEAVNMAEGIIHDTETK	iTRAQ4plex@N-term; Lys->Gln@17		0.0	2000.01	667.68	1999.96	667.66	3	97.0	48.3	66.8	33.2	0.5
			99	VEAVNMAEGIIHDTETK	iTRAQ4plex@N-term; Lys->Gln@17		0.0	1999.98	667.67	1999.96	667.66	3	69.7	45.0	60.8	39.2	0.6
			99	SQVFSTAADGQTQVEIK	iTRAQ4plex@N-term; Lys->Gln@17		0.0	1952.01	651.68	1951.96	651.66	3	26.5	26.1	50.3	49.7	1.0
			99	QAASSLQQASLK	iTRAQ4plex@N-term; No iTRAQ4plex(K)@12		0.0	1374.76	688.39	1374.76	688.39	2	494.5	239.3	67.4	32.6	0.5
			98	NAVITVPAYFNDSQR	iTRAQ4plex@N-term; Ser->Asn@13; Deamidated(Q)@14		0.0	1865.95	622.99	1865.94	622.99	3	11.8	27.7	29.9	70.1	2.3
			92	QAASSLQQASLK	iTRAQ4plex@N-term; iTRAQ4plex(K)@12		-6.0	1512.83	505.28	1518.86	507.29	3	908.6	1073.4	45.8	54.2	1.2
			91	DNMALQR	iTRAQ4plex@N-term		0.0	990.54	496.28	990.50	496.26	2	4361.8	3469.5	55.7	44.3	0.8
			83	YAEEDR	iTRAQ4plex@N-term		0.0	925.46	463.74	925.43	463.72	2	30.5	16.2	65.3	34.7	0.5
			81	NAVITVPAYFNDSQR	iTRAQ4plex@N-term; Val->Ser@6		-0.1	1825.84	609.62	1825.91	609.64	3	37.1	36.6	50.3	49.7	1.0
			44	EMAGDNK	iTRAQ4plex@N-term; iTRAQ4plex(K)@7		0.1	1051.58	526.79	1051.52	526.77	2					
			42	LYSPSQIGAFVLMK	iTRAQ4plex@N-term; Lys->Gln@14		0.0	1696.93	849.47	1696.90	849.46	2					
			40	YAEEDR	iTRAQ4plex@N-term		0.1	925.48	463.75	925.43	463.72	2					
			36	GVFEVK	iTRAQ4plex@N-term; iTRAQ4plex(K)@6		0.0	965.57	483.79	965.58	483.80	2					
			34	ASEAIK	iTRAQ4plex@N-term; iTRAQ4plex(K)@6	cleaved Y-A@N-term	0.1	905.59	453.80	905.54	453.78	2					
			31	NTTIPTK	iTRAQ4plex@N-term; iTRAQ4plex(K)@7		0.0	1061.67	531.84	1061.63	531.82	2					
			30	LLGQFTLIGIPPAPR	iTRAQ4plex@N-term; Ala->Val@13		0.1	1764.13	589.05	1764.08	589.03	3					
			21	DSETGENIR	iTRAQ4plex@N-term		0.0	1163.60	582.81	1163.55	582.78	2					
			15	TTPSVVAFTADGER	iTRAQ4plex@N-term; Glu->Arg@13; Deamidated(R)@14		0.0	1621.83	541.62	1621.85	541.63	3					
			12	STNGDTFLGGEDFDQALLR	iTRAQ4plex@N-term; Glu->Gln@11; Dimethyl(R)@19		0.0	2226.10	743.04	2226.10	743.04	3					
																Average	1.1
																Stdev	0.7
IPI:IPI00480560.1	HSPA8	Heat shock protein 8	99	DAGTIAGLNVLR	iTRAQ4plex@N-term		0.0	1342.75	672.38	1342.77	672.39	2	1236.8	806.8	60.5	39.5	0.7
			99	VEIIANDQGNR	iTRAQ4plex@N-term		0.0	1371.72	686.87	1371.72	686.87	2	4216.0	3455.6	55.0	45.0	0.8
			99	TTPSYVAFTDTER	iTRAQ4plex@N-term		-0.1	1630.74	816.38	1630.80	816.41	2	3046.3	2365.4	56.3	43.7	0.8
			99	VEIIANDQGNR	iTRAQ4plex@N-term		0.0	1371.73	686.87	1371.72	686.87	2	2125.9	1712.2	55.4	44.6	0.8
			99	TTPSYVAFTDTER	iTRAQ4plex@N-term		0.0	1630.76	816.39	1630.80	816.41	2	1482.7	1187.9	55.5	44.5	0.8
			99	VEIIANDQGNR	iTRAQ4plex@N-term		0.0	1371.71	686.86	1371.72	686.87	2	559.3	418.8	57.2	42.8	0.7
			99	STAGDTHLGGEDFDNR	iTRAQ4plex@N-term		1.0	1835.84	612.95	1834.82	612.61	3	457.2	458.1	50.0	50.0	1.0
			99	FEELNADLFR	iTRAQ4plex@N-term		0.0	1396.71	699.36	1396.71	699.36	2	410.0	397.3	50.8	49.2	1.0
			99	FEELNADLFR	iTRAQ4plex@N-term		0.0	1396.71	699.36	1396.71	699.36	2	352.5	315.9	52.7	47.3	0.9
			99	SQIHDIVLVGGSTR	iTRAQ4plex@N-term		0.0	1624.88	542.64	1624.90	542.64	3	233.5	269.3	46.4	53.6	1.2
			99	TVTNAVVTVPAYFNDSQR	iTRAQ4plex@N-term		0.0	2125.04	709.36	2125.09	709.37	3	29.3	49.8	37.0	63.0	1.7
			99	TVTNAVVTVPAYFNDSQR	iTRAQ4plex@N-term		0.0	2125.12	709.38	2125.09	709.37	3	28.1	32.4	46.4	53.6	1.2
			99	NSLESYAFNMK	iTRAQ4plex@N-term; iTRAQ4plex(K)@11		0.0	1590.77	531.27	1590.80	531.27	3	231.9	213.5	52.1	47.9	0.9
			99	SFYPEEVSSMVLTK	iTRAQ4plex@N-term; iTRAQ4plex(K)@14		0.0	1904.01	635.68	1903.98	635.67	3	179.3	150.5	54.4	45.6	0.8
			99	SFYPEEVSSMVLTK	iTRAQ4plex@N-term; iTRAQ4plex(K)@14		0.0	1903.96	635.66	1903.98	635.67	3	49.2	54.6	47.4	52.6	1.1
			99	NQVAMNPTNTVFDAK	iTRAQ4plex@N-term; iTRAQ4plex(K)@15		0.0	1936.97	646.66	1936.99	646.67	3	222.5	171.2	56.5	43.5	0.8
			99	SINPDEAVAYGAAVQAAILSGDI	iTRAQ4plex@N-term; iTRAQ4plex(K)@23		0.0	2547.36	850.13	2547.34	850.12	3	17.6	18.9	48.3	51.7	1.1
			99	SINPDEAVAYGAAVQAAILSGDI	iTRAQ4plex@N-term; iTRAQ4plex(K)@23		-1.0	2546.32	849.78	2547.34	850.12	3	12.3	13.7	47.4	52.6	1.1
			99	SINPDEAVAYGAAVQAAILSGDI	iTRAQ4plex@N-term; iTRAQ4plex(K)@23		0.0	2547.35	850.12	2547.34	850.12	3	7.0	8.4	45.4	54.6	1.2
			99	GTLDPVEK	iTRAQ4plex@N-term; iTRAQ4plex(K)@8		0.0	1145.66	573.84	1145.65	573.83	2	2505.8	2083.0	54.6	45.4	0.8
			99	LLQDFFNGK	iTRAQ4plex@N-term; iTRAQ4plex(K)@9		0.0	1368.78	685.40	1368.76	685.39	2	249.1	192.2	56.5	43.5	0.8
			99	MVNHFIAEFK	iTRAQ4plex@N-term; Lys->Gln@10		0.0	1378.70	460.57	1378.68	460.57	3	110.0	93.4	54.1	45.9	0.8
			99	NSLESYAFNMK	iTRAQ4plex@N-term; Lys->Gln@11		0.0	1446.68	724.35	1446.66	724.34	2	993.5	502.2	66.4	33.6	0.5
			99	NSLESYAFNMK	iTRAQ4plex@N-term; Lys->Gln@11		0.0	1446.66	724.34	1446.66	724.34	2	887.3	506.3	63.7	36.3	0.6
			99	RFDDAVVQSDMK	iTRAQ4plex@N-term; Lys->Gln@12	missed R-F@1	0.0	1553.75	518.93	1553.73	518.92	3	77.5	81.3	48.8	51.2	1.0
			99	NQVAMNPTNTVFDAK	iTRAQ4plex@N-term; Lys->Gln@15		0.0	1792.90	897.46	1792.85	897.43	2	412.4	282.5	59.3	40.7	0.7
			99	NQVAMNPTNTVFDAK	iTRAQ4plex@N-term; Lys->Gln@15		0.0	1792.89	598.64	1792.85	598.63	3	126.0	106.1	54.3	45.7	0.8
			99	EIAEAYLGK	iTRAQ4plex@N-term; Lys->Gln@9		0.1	1136.64	569.33	1136.58	569.30	2	348.3	333.3	51.1	48.9	1.0
			98	VEIIANDQGNR	iTRAQ4plex@N-term		0.0	1371.73	458.25	1371.72	458.25	3	19.3	22.1	46.7	53.3	1.1
			96	LLQDFFNGK	iTRAQ4plex@N-term; Lys->Gln@9		0.0	1224.66	613.34	1224.63	613.32	2	358.7	298.4	54.6	45.4	0.8
			95	ATVEDEK	iTRAQ4plex@N-term; iTRAQ4plex(K)@7		0.0	1078.61	540.31	1078.57	540.29	2	792.2	1005.3	44.1	55.9	1.3
			94	LLQDFFNGK	iTRAQ4plex@N-term; Lys->Gln@9		0.0	1224.67	613.34	1224.63	613.32	2	265.7	189.4	58.4	41.6	0.7
			93	LLQDFFNGK	iTRAQ4plex@N-term; Lys->Gln@9		0.0	1224.66	613.33	1224.63	613.32	2	235.8	156.1	60.2	39.8	0.7
			89	ITITNDK	iTRAQ4plex@N-term; iTRAQ4plex(K)@7		0.0	1091.63	546.82	1091.64	546.83	2	1272.7	1727.2	42.4	57.6	1.4
			82	GTLDPVEK	iTRAQ4plex@N-term; Lys->Gln@8		0.0	1001.56	501.79	1001.52	501.76	2	1424.1	887.7	61.6	38.4	0.6
			81	SFYPEEVSSMVLTK	iTRAQ4plex@N-term; Lys->Gln@14		0.1	1759.91	587.64	1759.85	587.62	3	14.1	15.8	47.2	52.8	1.1
			79	MVQEAEK	iTRAQ4plex@N-term; iTRAQ4plex(K)@7		0.1	1121.66	561.84	1121.60	561.81	2	3184.5	3083.8	50.8	49.2	1.0
			35	SFYPEEVSSMVLTK	iTRAQ4plex@N-term; iTRAQ4plex(T)@13; Lys->Arg@14		0.0	1932.00	645.01	1931.99	645.00	3					
			31	NTTIPTK	iTRAQ4plex@N-term; iTRAQ4plex(K)@7		0.0	1061.67	531.84	1061.63	531.82	2					
			15	ATVEDEK	iTRAQ4plex@N-term; Lys->Gln@7		0.1	934.49	468.25	934.44	468.23	2					
			14	TVTNAVVTVPAYFNDSQR	iTRAQ4plex@N-term; Gln->Arg@17		0.0	2153.12	718.71	2153.14	718.72	3					
			12	FNDSQRQATKDAGTIAGLNVLR	iTRAQ4plex@N-term; Gln->Arg@7; iTRAQ4plex(K)@10	cleaved Y-F@N-term; missed R-Q@6	0.0	2690.53	673.64	2690.48	673.63	4					
																Average	1.5
																Stdev	1.0
IPI:IPI00319992.1	HSPA5	Heat shock protein 5	99	DNHLLGTFDLTGIPPAPR	iTRAQ4plex@N-term		0.0	2077.12	693.38	2077.11	693.38	3	12.2	16.9	41.9	58.1	1.4
			99	ITITNDQNR	iTRAQ4plex@N-term		1.0	1218.67	610.34	1217.65	609.83	2	4956.4	3775.6	56.8	43.2	0.8
			99	VEIIANDQGNR	iTRAQ4plex@N-term		0.0	1371.72	686.87	1371.72	686.87	2	4216.0	3455.6	55.0	45.0	0.8
			99	VEIIANDQGNR	iTRAQ4plex@N-term		0.0	1371.73	686.87	1371.72	686.87	2	2125.9	1712.2	55.4	44.6	0.8
			99	DAGTIAGLNVMR	iTRAQ4plex@N-term		0.0	1360.71	681.36	1360.73	681.37	2	2063.2	1627.7	55.9	44.1	0.8
			99	ITITNDQNR	iTRAQ4plex@N-term		0.0	1217.68	609.85	1217.65	609.83	2	1763.5	1265.5	58.2	41.8	0.7
			99	ITPSYVAFTPEGER	iTRAQ4plex@N-term		0.0	1709.88	855.95	1709.87	855.94	2	1579.7	936.5	62.8	37.2	0.6
			99	VEIIANDQGNR	iTRAQ4plex@N-term		0.0	1371.71	686.86	1371.72	686.87	2	559.3	418.8	57.2	42.8	0.7
			99	ITPSYVAFTPEGER	iTRAQ4plex@N-term		0.0	1709.88	570.97	1709.87	570.97	3	142.9	95.3	60.0	40.0	0.7
			99	DNHLLGTFDLTGIPPAPR	iTRAQ4plex@N-term		0.0	2077.12	693.38	2077.11	693.38	3	12.2	16.9	41.9	58.1	1.4
			99	ELEEIVQPIISK	iTRAQ4plex@N-term; iTRAQ4plex(K)@12		0.0	1684.96	562.66	1684.99	562.67	3	33.6	32.4	50.9	49.1	1.0
			99	TFAPEEISAMVLTK	iTRAQ4plex@N-term; iTRAQ4plex(K)@14		0.0	1824.00	609.01	1823.99	609.01	3	58.9	40.1	59.5	40.5	0.7
			99	TFAPEEISAMVLTK	iTRAQ4plex@N-term; iTRAQ4plex(K)@14		0.0	1824.00	609.01	1823.99	609.01	3	31.9	27.0	54.2	45.8	0.8
			99	NQLTSNPENTVFDAK	iTRAQ4plex@N-term; iTRAQ4plex(K)@15		0.0	1964.97	656.00	1965.00	656.01	3	86.3	92.0	48.4	51.6	1.1
			99	IINEPTAAAIAYGLDKR	iTRAQ4plex@N-term; iTRAQ4plex(K)@16	missed K-R@16	0.0	2103.21	702.08	2103.19	702.07	3	329.9	185.8	64.0	36.0	0.6
			99	NELESYAYSLK	iTRAQ4plex@N-term; Lys->Gln@11		0.1	1459.75	730.88	1459.70	730.85	2	246.0	129.5	65.5	34.5	0.5
			99	TWNDPSVQQDIK	iTRAQ4plex@N-term; Lys->Gln@12		0.0	1573.79	787.90	1573.75	787.88	2	890.7	460.6	65.9	34.1	0.5
			99	TWNDPSVQQDIK	iTRAQ4plex@N-term; Lys->Gln@12		0.0	1573.75	787.88	1573.75	787.88	2	802.0	385.9	67.5	32.5	0.5
			99	IEWLESHQDADIEDFK	iTRAQ4plex@N-term; Lys->Gln@16		0.0	2118.00	707.01	2117.97	707.00	3	74.6	62.2	54.5	45.5	0.8
			99	SQIFSTASDNQPTVTIK	iTRAQ4plex@N-term; Lys->Gln@17		0.0	1980.02	661.02	1979.99	661.00	3	25.1	13.2	65.6	34.4	0.5
			99	IINEPTAAAIAYGLDKR	iTRAQ4plex@N-term; Lys->Val@16; Deamidated(R)@17	missed K-R@16	0.0	1931.09	644.70	1931.05	644.69	3	73.8	83.0	47.1	52.9	1.1
			99	IEWLESHQDADIEDFK	iTRAQ4plex@N-term; No iTRAQ4plex(K)@16		0.0	2117.99	707.01	2118.00	707.01	3	98.1	70.0	58.4	41.6	0.7
			99	LYGSGGPPPTGEEDTSEKDEL	iTRAQ4plex@N-term; No iTRAQ4plex(K)@18		0.0	2321.09	774.70	2321.07	774.70	3	83.9	56.3	59.9	40.1	0.7
			98	LTPEEIER	iTRAQ4plex@N-term		0.0	1129.59	565.80	1129.61	565.81	2	6837.7	4471.0	60.5	39.5	0.7
			98	VEIIANDQGNR	iTRAQ4plex@N-term		0.0	1371.73	458.25	1371.72	458.25	3	19.3	22.1	46.7	53.3	1.1
			98	DAGTIAGLNVMR	iTRAQ4plex@N-term; Dimethyl(R)@12		0.0	1388.73	463.92	1388.76	463.93	3	11.7	31.0	27.3	72.7	2.7
			98	NELESYAYSLK	iTRAQ4plex@N-term; iTRAQ4plex(K)@11		0.0	1603.83	535.62	1603.83	535.62	3	95.1	100.4	48.7	51.3	1.1
			98	SQIFSTASDNQPTVTIK	iTRAQ4plex@N-term; Lys->Gln@17		0.1	1980.05	661.02	1979.99	661.00	3	130.4	87.1	60.0	40.0	0.7
			96	MVNDAEK	iTRAQ4plex@N-term; iTRAQ4plex(K)@7		0.0	1093.61	547.81	1093.57	547.79	2	1364.7	1061.3	56.3	43.7	0.8
			82	SQIFSTASDNQPTVTIK	iTRAQ4plex@N-term; iTRAQ4plex(K)@17		4.9	2129.07	710.70	2124.13	709.05	3	304.6	427.6	41.6	58.4	1.4
			20	MVNDAEK	iTRAQ4plex@N-term; Lys->Gln@7		0.1	949.50	475.76	949.43	475.72	2					
			19	VTAEDK	iTRAQ4plex@N-term; iTRAQ4plex(K)@6		0.1	949.59	475.80	949.53	475.77	2					
																Average	0.9
																Stdev	0.4
IPI:IPI00130920.1	MTAP1B	Microtubule-associated protein 1B	99	AAEAGVTEEQYGYLGTSAK	iTRAQ4plex@N-term; Lys->Gln@19		0.0	2087.98	697.00	2087.98	697.00	3	16.0	13.5	54.2	45.8	0.8
			99	VSAEGEAR	iTRAQ4plex@N-term		0.0	961.52	481.77	961.50	481.75	2	352.7	162.2	68.5	31.5	0.5
			99	AQWGSNMQVTLIPTHDSEVMR	iTRAQ4plex@N-term		0.0	2543.22	848.75	2543.24	848.75	3	224.7	161.6	58.2	41.8	0.7
			99	VSAEGEAR	iTRAQ4plex@N-term		1.0	962.54	482.28	961.50	481.75	2	131.3	61.0	68.3	31.7	0.5
			99	SSYYVVSGNDPAAEEPSR	iTRAQ4plex@N-term		0.0	2070.96	691.33	2070.96	691.33	3	68.8	69.2	49.9	50.1	1.0
			99	TSDVETMSSQSALALDER	iTRAQ4plex@N-term		-6.0	2077.03	693.35	2082.99	695.34	3	55.7	29.0	65.7	34.3	0.5
			99	TSDVETMSSQSALALDER	iTRAQ4plex@N-term		0.0	2082.95	695.32	2082.99	695.34	3	51.6	41.3	55.5	44.5	0.8
			99	ETAAAHQASSSPPIDAATAEPYGFR	iTRAQ4plex@N-term		0.0	2688.32	897.11	2688.29	897.10	3	31.9	29.2	52.2	47.8	0.9
			99	GDSALFAVNGFNMLINGGSER	iTRAQ4plex@N-term		0.1	2312.22	771.75	2312.13	771.72	3	21.0	27.4	43.3	56.7	1.3
			99	VDSILLTHIGDDNLPGINSMLQR	iTRAQ4plex@N-term		0.0	2664.39	889.14	2664.40	889.14	3	21.0	27.4	43.3	56.7	1.3
			99	VDSILLTHIGDDNLPGINSMLQR	iTRAQ4plex@N-term		-0.9	2663.49	888.84	2664.40	889.14	3	14.1	14.7	49.0	51.0	1.0

			99	TATTGPGTTK	iTRAQ4plex@N-term; iTRAQ4plex(K)@10		1.1	1222.77	612.39	1221.68	611.85	2	121.5	80.9	60.0	40.0	0.7
			99	SVGNTIEPVILFQK	iTRAQ4plex@N-term; iTRAQ4plex(K)@14		0.0	1832.06	611.70	1832.07	611.70	3	37.4	29.0	56.3	43.7	0.8
			99	SVGNTIEPVILFQK	iTRAQ4plex@N-term; iTRAQ4plex(K)@14		0.0	1832.03	611.68	1832.07	611.70	3	31.9	28.1	53.2	46.8	0.9
			99	ESSPLYSPGFSDSTSAAK	iTRAQ4plex@N-term; iTRAQ4plex(K)@18		0.1	2118.09	707.04	2118.04	707.02	3	79.4	81.3	49.4	50.6	1.0
			99	VLFPGNSTQYNILEGLEK	iTRAQ4plex@N-term; iTRAQ4plex(K)@18		0.0	2309.24	770.75	2309.25	770.76	3	46.0	43.9	51.2	48.8	1.0
			99	AAEAGVTEEQYGYLGTSAK	iTRAQ4plex@N-term; iTRAQ4plex(K)@19		0.0	2232.08	745.03	2232.12	745.05	3	117.1	199.7	37.0	63.0	1.7
			99	ASLSPMDEPVPDSESPVEK	iTRAQ4plex@N-term; iTRAQ4plex(K)@19		-0.1	2301.06	768.03	2301.13	768.05	3	90.2	86.2	51.1	48.9	1.0
			99	SPPLLGSESPYEDFLSADSK	iTRAQ4plex@N-term; iTRAQ4plex(K)@20		0.0	2426.19	809.74	2426.21	809.74	3	85.9	52.0	62.3	37.7	0.6
			99	SPPLLGSESPYEDFLSADSK	iTRAQ4plex@N-term; iTRAQ4plex(K)@20		0.0	2426.19	809.74	2426.21	809.74	3	44.5	34.2	56.6	43.4	0.8
			99	TLEVVSPSQSVTGSAGHTPYYQSPTDEK	iTRAQ4plex@N-term; iTRAQ4plex(K)@28		0.1	3252.66	814.17	3252.60	814.16	4	73.2	51.3	58.8	41.2	0.7
			99	TTEAAATAVGTAATTAAVVAAAGIAASGPV	iTRAQ4plex@N-term; iTRAQ4plex(K)@31		0.0	2957.64	740.42	2957.63	740.41	4	82.0	209.1	28.2	71.8	2.5
			99	TTEAAATAVGTAATTAAVVAAAGIAASGPV	iTRAQ4plex@N-term; iTRAQ4plex(K)@31		-4.8	2952.78	739.20	2957.63	740.41	4	76.3	114.2	40.1	59.9	1.5
			99	NVDVEFFK	iTRAQ4plex@N-term; iTRAQ4plex(K)@8		0.0	1284.70	643.36	1284.70	643.36	2	4527.1	5505.0	45.1	54.9	1.2
			99	EETPEVTK	iTRAQ4plex@N-term; iTRAQ4plex(K)@8		0.0	1219.67	610.84	1219.65	610.83	2	1788.4	1434.6	55.5	44.5	0.8
			99	NAANASASK	iTRAQ4plex@N-term; iTRAQ4plex(K)@9		0.1	1120.68	561.35	1120.61	561.31	2	123.4	77.9	61.3	38.7	0.6
			99	AVLDALLEGK	iTRAQ4plex@N-term; Lys->Arg@10		0.0	1199.72	600.87	1199.70	600.86	2	33.3	89.8	27.1	72.9	2.7
			99	VLFPGNSTQYNILEGLEK	iTRAQ4plex@N-term; Lys->Arg@18		0.0	2193.16	732.06	2193.16	732.06	3	10.6	11.5	47.8	52.2	1.1
			99	VLFPGNSTQYNILEGLEK	iTRAQ4plex@N-term; Lys->Arg@18		0.1	2193.25	732.09	2193.16	732.06	3	7.1	6.3	53.1	46.9	0.9
			99	LEMYVLNPVK	iTRAQ4plex@N-term; Lys->Gln@10		0.0	1348.73	675.37	1348.72	675.37	2	675.2	450.0	60.0	40.0	0.7
			99	LEMYVLNPVK	iTRAQ4plex@N-term; Lys->Gln@10		0.0	1348.71	675.36	1348.72	675.37	2	487.4	331.3	59.5	40.5	0.7
			99	AVLDALLEGK	iTRAQ4plex@N-term; Lys->Gln@10		0.1	1171.72	586.87	1171.66	586.84	2	375.5	240.5	61.0	39.0	0.6
			99	AVLDALLEGK	iTRAQ4plex@N-term; Lys->Gln@10		0.0	1171.69	586.85	1171.66	586.84	2	286.3	226.5	55.8	44.2	0.8
			99	MSISEGTVSDK	iTRAQ4plex@N-term; Lys->Gln@11		0.0	1296.62	649.32	1296.60	649.31	2	1411.5	534.2	72.5	27.5	0.4
			99	SVNFSLTPNEIK	iTRAQ4plex@N-term; Lys->Gln@12		0.0	1491.77	746.89	1491.77	746.89	2	1093.7	366.1	74.9	25.1	0.3
			99	SVNFSLTPNEIK	iTRAQ4plex@N-term; Lys->Gln@12		0.1	1491.84	746.93	1491.77	746.89	2	309.0	106.4	74.4	25.6	0.3
			99	EMQYFMQQWTGTNK	iTRAQ4plex@N-term; Lys->Gln@14		0.1	1934.91	645.98	1934.84	645.95	3	39.3	26.8	59.5	40.5	0.7
			99	SVGNTIEPVILFQK	iTRAQ4plex@N-term; Lys->Gln@14		0.1	1688.01	563.68	1687.93	563.65	3	25.0	16.4	60.3	39.7	0.7
			99	EMQYFMQQWTGTNK	iTRAQ4plex@N-term; Lys->Gln@14		0.0	1934.85	645.96	1934.84	645.95	3	22.8	27.4	45.4	54.6	1.2
			99	ESPVSDLTSTGLYQDK	iTRAQ4plex@N-term; Lys->Gln@16		0.1	1882.94	628.66	1882.89	628.64	3	10.6	9.4	53.2	46.8	0.9
			99	VLFPGNSTQYNILEGLEK	iTRAQ4plex@N-term; Lys->Gln@18		0.0	2165.14	722.72	2165.11	722.71	3	46.6	26.5	63.8	36.2	0.6
			99	VLFPGNSTQYNILEGLEK	iTRAQ4plex@N-term; Lys->Gln@18		0.1	2165.19	722.74	2165.11	722.71	3	21.2	19.8	51.7	48.3	0.9
			99	ASLSPMDEPVPDSESPVEK	iTRAQ4plex@N-term; Lys->Gln@19		0.1	2157.05	720.02	2156.99	720.00	3	25.1	13.1	65.8	34.2	0.5
			99	TLEVVSPSQSVTGSAGHTPYYQSPTDEK	iTRAQ4plex@N-term; Lys->Gln@28		0.1	3108.52	778.14	3108.46	778.12	4	48.3	29.6	62.0	38.0	0.6
			99	TLEVVSPSQSVTGSAGHTPYYQSPTDEK	iTRAQ4plex@N-term; Lys->Gln@28		0.0	3108.45	778.12	3108.46	778.12	4	30.3	22.7	57.1	42.9	0.8
			99	TTEAAATAVGTAATTAAVVAAAGIAASGPVKELEAE	iTRAQ4plex@N-term; Lys->Gln@31		0.1	3540.90	886.23	3540.84	886.22	4	17.6	18.9	48.3	51.7	1.1
			99	VLFPGNSTQYNILEGLEK	iTRAQ4plex@N-term; Methyl(L)@16; Methyl(E)@17; iTRAQ4plex(K)@18		0.0	2337.32	780.11	2337.28	780.10	3	6.9	11.7	37.1	62.9	1.7
			99	TATTGPGTTK	iTRAQ4plex@N-term; No iTRAQ4plex(K)@10		0.0	1077.62	539.82	1077.58	539.80	2	894.3	464.8	65.8	34.2	0.5
			99	MSISEGTVSDK	iTRAQ4plex@N-term; No iTRAQ4plex(K)@11		0.0	1296.65	649.33	1296.64	649.32	2	1583.8	675.2	70.1	29.9	0.4
			99	TESKPSVTEK	iTRAQ4plex@N-term; No iTRAQ4plex(K)@4; iTRAQ4plex(K)@10		0.0	1392.82	465.28	1392.77	465.26	3	37.4	29.0	56.3	43.7	0.8
			99	ASLSPMDEPVPDSESPVEK	iTRAQ4plex@N-term; Phospho(S)@15; iTRAQ4plex(K)@19		0.0	2381.14	794.72	2381.10	794.71	3	28.3	27.1	51.1	48.9	1.0
			99	SWDTNLIECNLDQELK	iTRAQ4plex@N-term; Pyridylethyl(C)@9; iTRAQ4plex(K)@16		0.0	2313.13	772.05	2313.16	772.06	3	36.7	47.5	43.6	56.4	1.3
			98	VSAEGEAR	iTRAQ4plex@N-term		0.1	961.55	481.78	961.50	481.75	2	64.9	28.0	69.9	30.1	0.4
			98	AVLDALLEGK	iTRAQ4plex@N-term; iTRAQ4plex(K)@10		0.0	1315.78	439.60	1315.80	439.61	3	19.0	29.7	39.1	60.9	1.6
			97	VLFPGNSTQYNILEGLEK	iTRAQ4plex@N-term; iTRAQ4plex(K)@18		2.0	2311.30	771.44	2309.25	770.76	3	40.8	35.4	53.6	46.4	0.9
			96	EMQYFMQQWTGTNK	iTRAQ4plex@N-term; iTRAQ4plex(K)@14		0.1	2079.03	694.02	2078.98	694.00	3	13.8	22.3	38.3	61.7	1.6
			95	TATTGPGTTK	iTRAQ4plex@N-term; iTRAQ4plex(K)@10		0.1	1221.76	611.89	1221.68	611.85	2	41.0	30.9	57.0	43.0	0.8
			95	SPPLLGSESPYEDFLSADSK	iTRAQ4plex@N-term; Lys->Gln@20		0.1	2282.17	761.73	2282.07	761.70	3	47.4	4.7	90.9	9.1	0.1
			86	AEVAEK	iTRAQ4plex@N-term; iTRAQ4plex(K)@6		0.0	933.58	467.80	933.54	467.78	2	38.7	42.0	48.0	52.0	1.1
			82	NVDVEFFK	iTRAQ4plex@N-term; Lys->Gln@8		0.0	1140.57	571.29	1140.56	571.29	2	1355.1	722.3	65.2	34.8	0.5
			71	TDATDGK	iTRAQ4plex@N-term; iTRAQ4plex(K)@7		0.1	994.57	498.29	994.52	498.27	2	8.5	18.1	31.9	68.1	2.1
			67	DLTTSSVEK	iTRAQ4plex@N-term; iTRAQ4plex(K)@9		0.0	1266.72	634.37	1266.69	634.35	2	105.5	68.6	60.6	39.4	0.7
			65	ETQPGEAYVIQK	iTRAQ4plex@N-term; Lys->Gln@12		0.0	1505.78	502.93	1505.75	502.92	3	7.1	5.2	58.0	42.0	0.7
			46	ELEAER	iTRAQ4plex@N-term		0.1	889.51	445.76	889.46	445.74	2					
			35	ETEVSK	iTRAQ4plex@N-term; iTRAQ4plex(K)@6		0.0	979.58	490.79	979.54	490.78	2					
			35	EETPEVTK	iTRAQ4plex@N-term; Lys->Gln@8		0.1	1075.58	538.80	1075.52	538.77	2					
			34	EEQSPVK	iTRAQ4plex@N-term; Lys->Gln@7		0.1	959.56	480.79	959.47	480.74	2					
			22	ETAAAHQASSSPPIDAATAEPYGFF	iTRAQ4plex@N-term		-0.1	2688.22	673.06	2688.29	673.08	4					
			20	ELEAER	iTRAQ4plex@N-term		0.1	889.52	445.77	889.46	445.74	2					
			15	ELEAER	iTRAQ4plex@N-term		0.1	889.52	445.77	889.46	445.74	2					
			14	TTEAAATAVGTAATTAAVVAAAGIAASGPV	iTRAQ4plex@N-term; iTRAQ4plex(K)@31		1.0	2958.67	740.68	2957.63	740.41	4					
			14	DVSDER	iTRAQ4plex@N-term		0.0	863.45	432.73	863.41	432.71	2					
			12	SQGSTSNSDWMK	iTRAQ4plex@N-term; Lys->Gln@12		0.1	1470.72	736.37	1470.62	736.32	2					
																Average	0.9
																Stdev	0.5
IPI:IPI00676243.4	MTAP1A	Microtubule-associated protein 1 A	99	ASYYVVSGNDPANGEPSR	iTRAQ4plex@N-term		0.0	2025.94	676.32	2025.95	676.32	3	69.1	63.2	52.2	47.8	0.9
			99	AVVFETGEAGAASGAGSLPGEVF	iTRAQ4plex@N-term		-0.1	2275.10	759.38	2275.16	759.39	3	53.7	33.6	61.5	38.5	0.6
			99	AQWGENLQVTLIPTHDTEVTR	iTRAQ4plex@N-term		-1.0	2550.29	851.10	2551.32	851.45	3	49.8	40.5	55.2	44.8	0.8
			99	AVVFETGEAGAASGAGSLPGEVF	iTRAQ4plex@N-term		0.0	2275.16	759.39	2275.16	759.39	3	19.5	17.7	52.4	47.6	0.9
			99	AVLDALLEGK	iTRAQ4plex@N-term; Lys->Gln@10		0.1	1171.72	586.87	1171.66	586.84	2	375.5	240.5	61.0	39.0	0.6
			99	AVLDALLEGK	iTRAQ4plex@N-term; Lys->Gln@10		0.0	1171.69	586.85	1171.66	586.84	2	286.3	226.5	55.8	44.2	0.8
			99	STPSQVTSEEK	iTRAQ4plex@N-term; Lys->Gln@11		0.0	1335.67	668.84	1335.63	668.82	2	276.7	190.1	59.3	40.7	0.7
			99	LSSQYAAVFGAPGHALHPGEPALGEVEEF	iTRAQ4plex@N-term; Oxidation(H)@17		-0.1	3148.52	788.14	3148.57	788.15	4	51.8	33.8	60.6	39.4	0.7
			98	AVLDALLEGK	iTRAQ4plex@N-term; iTRAQ4plex(K)@10		0.0	1315.78	439.60	1315.80	439.61	3	19.0	29.7	39.1	60.9	1.6
			94	AGSTALGSK	iTRAQ4plex@N-term; Lys->Gln@9		0.1	934.54	468.28	934.48	468.25	2	17.8	14.5	55.0	45.0	0.8
			92	EGAALDQDNR	iTRAQ4plex@N-term		0.0	1231.64	616.83	1231.59	616.80	2	58.8	44.5	56.9	43.1	0.8
			79	ELSSEPR	iTRAQ4plex@N-term		0.0	960.54	481.28	960.50	481.26	2	73.1	51.4	58.7	41.3	0.7
			40	NRAPDSGAEVER	iTRAQ4plex@N-term	missed R-A@2	0.1	1443.82	482.28	1443.72	482.25	3					
			34	NVTTSEK	iTRAQ4plex@N-term; iTRAQ4plex(K)@7		0.1	1065.65	533.83	1065.59	533.80	2					
																Average	0.8
																Stdev	0.3
IPI:IPI00652436.3	ACTG1	Actin, gamma, cytoplasmic 1	99	AVFPSIVGR	iTRAQ4plex@N-term	cleaved R-P@C-term	0.0	1088.64	545.33	1088.65	545.33	2	485.7	928.6	34.3	65.7	1.9
			99	GYSFTTTAER	iTRAQ4plex@N-term		0.0	1275.60	638.81	1275.62	638.82	2	998.1	1130.1	46.9	53.1	1.1
			99	GYSFTTTAER	iTRAQ4plex@N-term		0.0	1275.65	638.83	1275.62	638.82	2	962.4	1102.8	46.6	53.4	1.1
			99	GYSFTTTAER	iTRAQ4plex@N-term		-8.0	1267.60	634.81	1275.62	638.82	2	323.3	316.3	50.6	49.4	1.0
			99	TTGIVMDSGDGVTHTVPIYEGYALPHAILR	iTRAQ4plex@N-term		0.0	3326.73	832.69	3326.71	832.68	4	53.1	47.8	52.7	47.3	0.9
			99	TTGIVMDSGDGVTHTVPIYEGYALPHAILR	iTRAQ4plex@N-term		0.0	3326.75	832.69	3326.71	832.68	4	45.9	46.0	49.9	50.1	1.0
			99	DLYANTVLSGGTTMYPGIADR	iTRAQ4plex@N-term		0.0	2358.19	787.07	2358.16	787.06	3	40.8	36.3	52.9	47.1	0.9
			99	SYELPDGQVITIGNER	iTRAQ4plex@N-term		0.0	1933.96	645.66	1933.99	645.67	3	30.6	64.0	32.4	67.6	2.1
			99	SYELPDGQVITIGNER	iTRAQ4plex@N-term		0.0	1933.96	645.66	1933.99	645.67	3	28.0	34.6	44.7	55.3	1.2
			99	TTGIVMDSGDGVTHTVPIYEGYALPH	iTRAQ4plex@N-term	cleaved H-A@C-term	0.0	2873.39	958.81	2873.40	958.81	3	25.1	62.9	28.5	71.5	2.5
			99	SYELPDGQVITIGNER	iTRAQ4plex@N-term		0.0	1933.98	645.67	1933.99	645.67	3	22.6	32.8	40.8	59.2	1.5
			99	VAPEEHPVLLTEAPLNPK	iTRAQ4plex@N-term; iTRAQ4plex(K)@18		0.0	2241.26	561.32	2241.26	561.32	4	106.0	102.9	50.7	49.3	1.0
			99	DLTDYLMK	iTRAQ4plex@N-term; iTRAQ4plex(K)@8		0.0	1285.65	643.83	1285.68	643.85	2	460.9	656.5	41.2	58.8	1.4
			99	DLTDYLMK	iTRAQ4plex@N-term; iTRAQ4plex(K)@8		0.0	1285.67	643.84	1285.68	643.85	2	320.1	452.9	41.4	58.6	1.4
			99	EITALAPSTMK	iTRAQ4plex@N-term; Lys->Arg@11		0.0	1332.76	667.39	1332.72	667.37	2	309.2	450.7	40.7	59.3	1.5
			99	DSYVGDEAQSK	iTRAQ4plex@N-term; Lys->Arg@11		0.0	1369.66	685.84	1369.62	685.82	2	224.8	457.5	32.9	67.1	2.0
			99	EITALAPSTMK	iTRAQ4plex@N-term; Lys->Arg@11		0.0	1332.70	667.36	1332.72	667.37	2	139.0	252.3	35.5	64.5	1.8
			99	DSYVGDEAQSK	iTRAQ4plex@N-term; Lys->Gln@11		0.1	1341.63	671.82	1341.58	671.80	2	621.5	519.6	54.5	45.5	0.8
			99	DSYVGDEAQSK	iTRAQ4plex@N-term; Lys->Gln@11		0.1	1341.64	671.83	1341.58	671.80	2	283.5	253.7	52.8	47.2	0.9
			99	DSYVGDEAQSK	iTRAQ4plex@N-term; Lys->Gln@11		0.1	1341.64	671.83	1341.58	671.80	2	167.9	160.5	51.1	48.9	1.0
			99	YPIEHGIVTNWDDMEK	iTRAQ4plex@N-term; Methyl(H)@5; Lys->Gln@16		0.0	2103.95	702.33	2103.97	702.33	3	311.3	243.1	56.2	43.8	0.8
			99	EITALAPSTMK	iTRAQ4plex@N-term; No iTRAQ4plex(K)@11		0.0	1304.71	653.36	1304.71	653.36	2	1060.9	762.9	58.2	41.8	0.7
			99	DSYVGDEAQSK	iTRAQ4plex@N-term; Ser->Val@10; iTRAQ4plex(K)@11		0.0	1497.77	500.26	1497.76	500.26	3	280.1	96.7	74.3	25.7	0.3
			98	DLTDYLMK	iTRAQ4plex@N-term; iTRAQ4plex(K)@8		-7.0	1278.72	640.37	1285.68	643.85	2	139.0	152.9	47.6	52.4	1.1
			61	EITALAPSTMK	iTRAQ4plex@N-term; iTRAQ4plex(K)@11		0.0	1448.79	483.94	1448.82	483.95	3	303.6	356.2	46.0	54.0	1.2
			18	DLTDYLMK	iTRAQ4plex@N-term; Lys->Gln@8		0.0	1141.58	571.80	1141.54	571.78	2					
																Average	1.2
																Stdev	0.5
IPI:IPI00387557.3	ACTN2	Actinin alpha 2	99	AGTQIENIEEDFR	iTRAQ4plex@N-term		0.0	1664.81	833.41	1664.81	833.41	2	126.5	94.0	57.4	42.6	0.7
			99	DQSLQEELAR	iTRAQ4plex@N-term		0.0	1331.71	666.86	1331.68	666.85	2	1755.1	943.3	65.0	35.0	0.5
			99	DQSLQEELAR	iTRAQ4plex@N-term		0.0	1331.68	666.85	1331.68	666.85	2	1445.8	745.6	66.0	34.0	0.5
			99	VLAVNQENER	iTRAQ4plex@N-term		0.0	1314.69	658.35	1314.70	658.36	2	928.3	485.3	65.7	34.3	0.5
			99	MVSDIAGAWQR	iTRAQ4plex@N-term		0.0	1376.71	689.36	1376.70	689.36	2	870.8	455.6	65.7	34.3	0.5
			99	TINEVETQILTR	iTRAQ4plex@N-term		0.0	1559.88	780.94	1559.86	780.94	2	249.3	136.9	64.6	35.4	0.5
			99	LASELLEWIR	iTRAQ4plex@N-term		0.0	1372.81	687.41	1372.78	687.40	2	181.6	87.5	67.5	32.5	0.5
			99	LASELLEWIR	iTRAQ4plex@N-term		0.0	1372.81	687.41	1372.78	687.40	2	133.2	61.1	68.5	31.5	0.5
			99	VGWELLLTTIAR	iTRAQ4plex@N-term		0.0	1514.92	758.47	1514.89	758.45	2	67.7	49.4	57.8	42.2	0.7
			99	VGWELLLTTIAR	iTRAQ4plex@N-term		0.0	1514.88	758.45	1514.89	758.45	2	63.1	26.9	70.1	29.9	0.4
			99	TINEVETQILTR	iTRAQ4plex@N-term		0.0	1559.86	520.96	1559.86	520.96	3	17.8	13.4	57.0	43.0	0.8
			99	LLETIDQLHLEFAK	iTRAQ4plex@N-term; iTRAQ4plex(K)@14		0.0	1957.10	653.37	1957.11	653.38	3	29.7	39.1	43.2	56.8	1.3
			99	SSIQITGALEDQMNQLK	iTRAQ4plex@N-term; iTRAQ4plex(K)@17		0.0	2163.16	722.06	2163.14	722.06	3	49.6	45.9	51.9	48.1	0.9
			99	ILASDKPYILAEELR	iTRAQ4plex@N-term; iTRAQ4plex(K)@6		0.0	2018.12	673.72	2018.17	673.73	3	91.2	57.4	61.4	38.6	0.6
			99	ILASDKPYILAEELR	iTRAQ4plex@N-term; iTRAQ4plex(K)@6		-9.1	2009.05	670.69	2018.17	673.73	3	83.5	69.6	54.5	45.5	0.8
			99	QFAAQANAIGPWIQNK	iTRAQ4plex@N-term; Lys->Gln@16		0.0	1900.00	634.34	1899.97	634.33	3	43.3	66.4	39.5	60.5	1.5
			97	SSIQITGALEDQMNQLK	iTRAQ4plex@N-term; Lys->Gln@17		0.0	2019.05	674.02	2019.01	674.01	3	12.7	3.9	76.6	23.4	0.3
			24	ATLPEADGER	iTRAQ4plex@N-term		0.0	1201.63	601.82	1201.61	601.81	2					
			17	TMQAMQK	iTRAQ4plex@N-term; Lys->Gln@7		0.1	980.55	491.28	980.45	491.23	2					
			15	EALER	iTRAQ4plex@N-term		0.0	760.46	381.24	760.42	381.22	2					
																Average	0.7
																Stdev	0.3
IPI:IPI00403810.2	TUBA1C	Tubulin, alpha	99	EDAANNYAR	iTRAQ4plex@N-term		0.0	1166.58	584.30	1166.54	584.28	2	657.2	794.2	45.3	54.7	1.2
			99	QLFHPEQLITGKEDAANNYAR	iTRAQ4plex@N-term; Lys->Gln@12		0.1	2558.31	640.59	2558.26	640.57	4	104.7	39.3	72.7	27.3	0.4
			99	TIGGGDDSFNTFFSETGAGK	iTRAQ4plex@N-term; Lys->Gln@20		0.0	2150.96	717.99	2150.95	717.99	3	31.9	29.2	52.2	47.8	0.9
			99	TIGGGDDSFNTFFSETGAGK	iTRAQ4plex@N-term; Lys->Gln@20		0.0	2150.99	718.00	2150.95	717.99	3	26.5	25.1	51.4	48.6	0.9
			80	EDAANNYAR	iTRAQ4plex@N-term		0.0	1166.56	584.29	1166.54	584.28	2	24.8	23.0	51.9	48.1	0.9
			50	EIIDLVLDR	iTRAQ4plex@N-term		0.0	1228.71	615.36	1228.71	615.36	2	92.5	121.0	43.3	56.7	1.3
																Average	0.9
																Stdev	0.3
IPI:IPI00338039.1	TUBB2A	Tubulin, beta	99	AILVDLEPGTMDSVR	iTRAQ4plex@N-term		0.0	1758.95	587.33	1758.93	587.32	3	24.1	42.4	36.2	63.8	1.8
			99	LAVNMVPFPR	iTRAQ4plex@N-term		0.0	1286.73	644.37	1286.73	644.37	2	823.6	846.0	49.3	50.7	1.0
			99	ISEQFTAMFR	iTRAQ4plex@N-term		0.0	1372.66	687.34	1372.69	687.35	2	174.6	229.6	43.2	56.8	1.3
			99	GHYTEGAELVDSVLDVVR	iTRAQ4plex@N-term		0.0	2102.09	701.71	2102.08	701.70	3	36.8	44.2	45.4	54.6	1.2
			99	GHYTEGAELVDSVLDVVR	iTRAQ4plex@N-term		0.0	2102.07	701.70	2102.08	701.70	3	10.0	25.7	28.1	71.9	2.6
			99	NSSYFVEWIPNNVK	iTRAQ4plex@N-term; iTRAQ4plex(K)@14		0.0	1984.03	662.35	1984.03	662.35	3	22.7	30.6	42.5	57.5	1.4
			99	NSSYFVEWIPNNVK	iTRAQ4plex@N-term; iTRAQ4plex(K)@14		0.0	1983.99	662.34	1984.03	662.35	3	18.8	35.1	34.9	65.1	1.9
			99	ALTVPELTQQMFDSK	iTRAQ4plex@N-term; iTRAQ4plex(K)@15		0.0	1995.08	666.03	1995.06	666.03	3	6.9	11.7	37.1	62.9	1.7
			99	MSATFIGNSTAIQELFK	iTRAQ4plex@N-term; iTRAQ4plex(K)@17		0.0	2145.16	716.06	2145.14	716.05	3	1.7	8.7	16.3	83.7	5.1
			92	ALTVPELTQQMFDSK	iTRAQ4plex@N-term; Lys->Gln@15		0.0	1850.95	617.99	1850.92	617.98	3	8.8	10.5	45.4	54.6	1.2
			58	IMNTFSVMPSPK	iTRAQ4plex@N-term; iTRAQ4plex(K)@12		0.0	1638.88	547.30	1638.87	547.30	3	187.4	128.4	59.3	40.7	0.7
																Average	1.8
																Stdev	1.2
IPI:IPI00752412.1	ATP1A3	ATPase, Na+/K+ transporting, alpha 3	99	DFTSEQIDEILQNHTEIVFAR	iTRAQ4plex@N-term		-0.1	2648.26	883.76	2648.32	883.78	3	21.0	27.3	43.4	56.6	1.3
			99	NMVPQQALVIR	iTRAQ4plex@N-term		0.0	1411.76	706.89	1411.81	706.91	2	5773.1	5610.0	50.7	49.3	1.0
			99	VDNSSLTGESEPQTR	iTRAQ4plex@N-term		0.0	1762.80	882.41	1762.85	882.43	2	1908.1	2113.1	47.5	52.5	1.1
			99	LNIPVSQVNPR	iTRAQ4plex@N-term		0.0	1379.83	690.92	1379.80	690.91	2	1840.4	1813.2	50.4	49.6	1.0
			99	NMVPQQALVIR	iTRAQ4plex@N-term		0.0	1411.76	706.89	1411.81	706.91	2	1467.5	1502.7	49.4	50.6	1.0
			99	LNIPVSQVNPR	iTRAQ4plex@N-term		0.0	1379.78	690.90	1379.80	690.91	2	1044.0	874.0	54.4	45.6	0.8
			99	YQLSIHETEDPNDNR	iTRAQ4plex@N-term		0.0	1973.95	658.99	1973.92	658.98	3	695.7	1041.9	40.0	60.0	1.5
			99	VDNSSLTGESEPQTR	iTRAQ4plex@N-term		0.0	1762.84	588.62	1762.85	588.62	3	360.6	346.2	51.0	49.0	1.0
			99	YQLSIHETEDPNDNR	iTRAQ4plex@N-term		0.0	1973.95	658.99	1973.92	658.98	3	208.4	305.1	40.6	59.4	1.5
			99	LNIPVSQVNPR	iTRAQ4plex@N-term		0.0	1379.76	460.93	1379.80	460.94	3	143.7	123.4	53.8	46.2	0.9
			99	GVGIISEGNETVEDIAAR	iTRAQ4plex@N-term		1.9	1974.97	659.33	1973.02	658.68	3	74.7	59.9	55.5	44.5	0.8
			99	GVGIISEGNETVEDIAAR	iTRAQ4plex@N-term		0.0	1973.04	658.69	1973.02	658.68	3	54.8	53.1	50.8	49.2	1.0
			99	DFTSEQIDEILQNHTEIVFAR	iTRAQ4plex@N-term		0.0	2648.27	883.76	2648.32	883.78	3	48.4	77.1	38.6	61.4	1.6
			99	GVGIISEGNETVEDIAAR	iTRAQ4plex@N-term		4.0	1977.03	660.02	1973.02	658.68	3	48.0	37.0	56.5	43.5	0.8
			99	GVGIISEGNETVEDIAAR	iTRAQ4plex@N-term		0.0	1972.98	658.67	1973.02	658.68	3	38.9	36.3	51.8	48.2	0.9
			99	EAFQNAYLELGGLGER	iTRAQ4plex@N-term		0.0	1909.97	637.67	1909.97	637.66	3	22.9	25.2	47.6	52.4	1.1
			99	TVNDLEDSYGQQWTYEQR	iTRAQ4plex@N-term		0.0	2375.11	792.71	2375.08	792.70	3	13.9	21.2	39.5	60.5	1.5
			99	GVGIISEGNETVEDIAARLNIPVSQVNPR	iTRAQ4plex@N-term	missed R-L@18	-1.0	3189.71	798.44	3190.71	798.68	4	10.6	10.5	50.3	49.7	1.0
			99	NMVPQQALVIR	iTRAQ4plex@N-term; Deamidated(N)@1		0.0	1412.74	707.38	1412.79	707.40	2	186.8	195.5	48.9	51.1	1.0
			99	QGAIVAVTGDGVNDSPALK	iTRAQ4plex@N-term; Deamidated(Q)@1; Lys->Gln@19		0.1	1956.12	653.05	1955.99	653.00	3	52.8	57.4	47.9	52.1	1.1
			99	GVGIISEGNETVEDIAAR	iTRAQ4plex@N-term; Dimethyl(R)@18		0.1	2001.14	668.05	2001.05	668.02	3	20.6	36.1	36.4	63.6	1.8
			99	DFTSEQIDEILQNHTEIVFAR	iTRAQ4plex@N-term; Glu->Gln@16		-0.1	2647.20	662.81	2647.34	662.84	4	10.7	8.3	56.3	43.7	0.8
			99	GGQDNIPVLK	iTRAQ4plex@N-term; iTRAQ4plex(K)@10		0.0	1327.75	664.88	1327.77	664.89	2	1380.7	2124.8	39.4	60.6	1.5
			99	QGAIVAVTGDGVNDSPALK	iTRAQ4plex@N-term; iTRAQ4plex(K)@19		0.0	2099.16	700.73	2099.15	700.72	3	184.2	217.2	45.9	54.1	1.2
			99	AAVPDAVGK	iTRAQ4plex@N-term; iTRAQ4plex(K)@9		0.0	1114.69	558.35	1114.66	558.34	2	1607.5	2177.6	42.5	57.5	1.4
			99	VAEIPFNSTNK	iTRAQ4plex@N-term; Lys->Gln@11		0.0	1362.72	682.37	1362.69	682.35	2	1689.1	1548.6	52.2	47.8	0.9
			99	VAEIPFNSTNK	iTRAQ4plex@N-term; Lys->Gln@11		0.1	1362.75	682.38	1362.69	682.35	2	904.7	780.5	53.7	46.3	0.9
			99	VAEIPFNSTNK	iTRAQ4plex@N-term; Lys->Gln@11		0.0	1362.70	682.36	1362.69	682.35	2	805.5	691.9	53.8	46.2	0.9
			99	VIMVTGDHPITAK	iTRAQ4plex@N-term; Lys->Gln@13		0.1	1524.88	509.30	1524.81	509.28	3	554.6	545.5	50.4	49.6	1.0
			99	DVAGDASESALLK	iTRAQ4plex@N-term; Lys->Gln@13		0.0	1418.71	710.36	1418.70	710.36	2	400.6	353.0	53.2	46.8	0.9
			99	MTVAHMWFDNQIHEADTTEDQSGTSFDK	iTRAQ4plex@N-term; Lys->Gln@28		0.0	3384.48	847.13	3384.44	847.12	4	39.8	63.6	38.5	61.5	1.6
			99	EQPLDEEMK	iTRAQ4plex@N-term; Lys->Gln@9		0.1	1261.63	631.82	1261.56	631.79	2	398.1	873.3	31.3	68.7	2.2
			99	NLEAVETLGSTSTICSDK	iTRAQ4plex@N-term; Pyridylethyl(C)@15; iTRAQ4plex(K)@18		0.0	2260.17	754.40	2260.15	754.39	3	523.8	638.6	45.1	54.9	1.2
			99	NLEAVETLGSTSTICSDK	iTRAQ4plex@N-term; Pyridylethyl(C)@15; Lys->Gln@18		0.0	2116.05	706.36	2116.01	706.34	3	595.2	636.0	48.3	51.7	1.1
			99	NITFFSTNCVEGTAR	iTRAQ4plex@N-term; Pyridylethyl(C)@9		0.0	1907.93	636.98	1907.93	636.98	3	954.3	925.8	50.8	49.2	1.0
			96	EAFQNAYLELGGLGER	iTRAQ4plex@N-term		-7.0	1902.95	635.32	1909.97	637.66	3	81.7	67.3	54.8	45.2	0.8
			93	EQPLDEEMK	iTRAQ4plex@N-term; Lys->Arg@9		0.0	1289.63	645.82	1289.60	645.81	2	717.8	788.7	47.6	52.4	1.1
			90	AQEILAR	iTRAQ4plex@N-term		0.0	943.58	472.80	943.56	472.79	2	7210.1	7347.0	49.5	50.5	1.0
			79	EAFQNAYLELGGLGER	iTRAQ4plex@N-term		-7.0	1902.96	635.33	1909.97	637.66	3	31.7	33.5	48.6	51.4	1.1
			76	TGTLTQNR	iTRAQ4plex@N-term		0.1	1033.63	517.82	1033.56	517.79	2	428.7	536.3	44.4	55.6	1.3
			52	QGAIVAVTGDGVNDSPALKK	iTRAQ4plex@N-term; iTRAQ4plex(K)@19; Lys->Gln@20	missed K-K@19	0.1	2227.26	557.82	2227.21	557.81	4	668.7	527.9	55.9	44.1	0.8
			36	YLLVMK	iTRAQ4plex@N-term; iTRAQ4plex(K)@6		0.0	1053.63	527.82	1053.65	527.83	2					
			32	GVGIISEGNETVEDIAARLNIPVSQVNPR	iTRAQ4plex@N-term	missed R-L@18	-1.0	3189.73	798.44	3190.71	798.68	4					
			26	DFTSEQIDEILQNHTEIVFAR	iTRAQ4plex@N-term		-0.9	2647.40	662.86	2648.32	663.09	4					
			23	TGTLTQNR	iTRAQ4plex@N-term		0.0	1033.61	517.81	1033.56	517.79	2					
			20	TVNDLEDSYGQQWTYEQR	iTRAQ4plex@N-term; Thr->Ser@1; Oxidation(N)@3; Deamidated(Q)@12		0.0	2378.08	793.70	2378.04	793.69	3					
			13	LIFDNLK	iTRAQ4plex@N-term; iTRAQ4plex(K)@7		0.0	1149.68	575.85	1149.70	575.86	2					
																Average	1.1
																Stdev	0.3
IPI:IPI00762871.2	ATP1A2	ATPase, Na+/K+ transporting, alpha 2	99	AAVPDAVGK	iTRAQ4plex@N-term; iTRAQ4plex(K)@9		0.0	1114.69	558.35	1114.66	558.34	2	1607.5	2177.6	42.5	57.5	1.4
			99	SPTWTALSR	iTRAQ4plex@N-term		0.0	1161.60	581.81	1161.63	581.82	2	6520.4	5739.8	53.2	46.8	0.9
			99	NMVPQQALVIR	iTRAQ4plex@N-term		0.0	1411.76	706.89	1411.81	706.91	2	5773.1	5610.0	50.7	49.3	1.0
			99	VDNSSLTGESEPQTR	iTRAQ4plex@N-term		0.0	1762.80	882.41	1762.85	882.43	2	1908.1	2113.1	47.5	52.5	1.1
			99	LNIPVSQVNPR	iTRAQ4plex@N-term		0.0	1379.83	690.92	1379.80	690.91	2	1840.4	1813.2	50.4	49.6	1.0
			99	NMVPQQALVIR	iTRAQ4plex@N-term		0.0	1411.76	706.89	1411.81	706.91	2	1467.5	1502.7	49.4	50.6	1.0
			99	LNIPVSQVNPR	iTRAQ4plex@N-term		0.0	1379.78	690.90	1379.80	690.91	2	1044.0	874.0	54.4	45.6	0.8
			99	DMTSEQLDEILR	iTRAQ4plex@N-term		0.0	1592.75	797.38	1592.78	797.40	2	652.1	534.0	55.0	45.0	0.8
			99	SPEFTHENPLETR	iTRAQ4plex@N-term		0.0	1699.79	567.60	1699.83	567.62	3	463.4	387.6	54.5	45.5	0.8
			99	VDNSSLTGESEPQTR	iTRAQ4plex@N-term		0.0	1762.84	588.62	1762.85	588.62	3	360.6	346.2	51.0	49.0	1.0
			99	DHTEIVFAR	iTRAQ4plex@N-term		0.0	1230.65	411.23	1230.65	411.22	3	290.3	269.4	51.9	48.1	0.9
			99	LNIPVSQVNPR	iTRAQ4plex@N-term		0.0	1379.76	460.93	1379.80	460.94	3	143.7	123.4	53.8	46.2	0.9
			99	GVGIISEGNETVEDIAAR	iTRAQ4plex@N-term		1.9	1974.97	659.33	1973.02	658.68	3	74.7	59.9	55.5	44.5	0.8
			99	GVGIISEGNETVEDIAAR	iTRAQ4plex@N-term		0.0	1973.04	658.69	1973.02	658.68	3	54.8	53.1	50.8	49.2	1.0
			99	GVGIISEGNETVEDIAAR	iTRAQ4plex@N-term		4.0	1977.03	660.02	1973.02	658.68	3	48.0	37.0	56.5	43.5	0.8
			99	GVGIISEGNETVEDIAAR	iTRAQ4plex@N-term		0.0	1972.98	658.67	1973.02	658.68	3	38.9	36.3	51.8	48.2	0.9
			99	GVGIISEGNETVEDIAARLNIPVSQVNPR	iTRAQ4plex@N-term	missed R-L@18	-1.0	3189.71	798.44	3190.71	798.68	4	10.6	10.5	50.3	49.7	1.0
			99	EMQDAFQNAYMELGGLGER	iTRAQ4plex@N-term		0.0	2302.01	768.34	2302.05	768.36	3	6.9	12.8	34.9	65.1	1.9
			99	NMVPQQALVIR	iTRAQ4plex@N-term; Deamidated(N)@1		0.0	1412.74	707.38	1412.79	707.40	2	186.8	195.5	48.9	51.1	1.0
			99	QGAIVAVTGDGVNDSPALK	iTRAQ4plex@N-term; Deamidated(Q)@1; Lys->Gln@19		0.1	1956.12	653.05	1955.99	653.00	3	52.8	57.4	47.9	52.1	1.1
			99	GVGIISEGNETVEDIAAR	iTRAQ4plex@N-term; Dimethyl(R)@18		0.1	2001.14	668.05	2001.05	668.02	3	20.6	36.1	36.4	63.6	1.8
			99	AGQENISVSK	iTRAQ4plex@N-term; iTRAQ4plex(K)@10		0.0	1319.78	660.89	1319.73	660.87	2	1164.6	1879.7	38.3	61.7	1.6
			99	EDSPQSHVLVMK	iTRAQ4plex@N-term; iTRAQ4plex(K)@12		0.1	1656.93	553.32	1656.87	553.30	3	491.1	432.2	53.2	46.8	0.9
			99	DTAGDASESALLK	iTRAQ4plex@N-term; iTRAQ4plex(K)@13		0.0	1564.81	522.61	1564.82	522.61	3	154.5	127.4	54.8	45.2	0.8
			99	DTAGDASESALLK	iTRAQ4plex@N-term; iTRAQ4plex(K)@13		0.0	1564.84	783.43	1564.82	783.42	2	141.2	142.0	49.9	50.1	1.0
			99	MQINAEEVVVGDLVEVK	iTRAQ4plex@N-term; iTRAQ4plex(K)@17		-1.0	2158.20	720.41	2159.18	720.73	3	23.3	13.2	63.8	36.2	0.6
			99	QGAIVAVTGDGVNDSPALK	iTRAQ4plex@N-term; iTRAQ4plex(K)@19		0.0	2099.16	700.73	2099.15	700.72	3	184.2	217.2	45.9	54.1	1.2
			99	AGQENISVSK	iTRAQ4plex@N-term; Lys->Gln@10		0.1	1175.69	588.85	1175.59	588.80	2	284.9	315.3	47.5	52.5	1.1
			99	VAEIPFNSTNK	iTRAQ4plex@N-term; Lys->Gln@11		0.0	1362.72	682.37	1362.69	682.35	2	1689.1	1548.6	52.2	47.8	0.9
			99	VAEIPFNSTNK	iTRAQ4plex@N-term; Lys->Gln@11		0.1	1362.75	682.38	1362.69	682.35	2	904.7	780.5	53.7	46.3	0.9
			99	VAEIPFNSTNK	iTRAQ4plex@N-term; Lys->Gln@11		0.0	1362.70	682.36	1362.69	682.35	2	805.5	691.9	53.8	46.2	0.9
			99	VIMVTGDHPITAK	iTRAQ4plex@N-term; Lys->Gln@13		0.1	1524.88	509.30	1524.81	509.28	3	554.6	545.5	50.4	49.6	1.0
			99	DTAGDASESALLK	iTRAQ4plex@N-term; Lys->Gln@13		0.0	1420.72	711.37	1420.68	711.35	2	284.5	176.7	61.7	38.3	0.6
			99	MQINAEEVVVGDLVEVK	iTRAQ4plex@N-term; Lys->Gln@17		0.1	2015.09	672.70	2015.04	672.69	3	1.7	3.3	34.3	65.7	1.9
			99	NLEAVETLGSTSTICSDK	iTRAQ4plex@N-term; Pyridylethyl(C)@15; iTRAQ4plex(K)@18		0.0	2260.17	754.40	2260.15	754.39	3	523.8	638.6	45.1	54.9	1.2
			99	NLEAVETLGSTSTICSDK	iTRAQ4plex@N-term; Pyridylethyl(C)@15; Lys->Gln@18		0.0	2116.05	706.36	2116.01	706.34	3	595.2	636.0	48.3	51.7	1.1
			97	FDTDELNFPTEK	iTRAQ4plex@N-term; iTRAQ4plex(K)@12		0.0	1742.84	581.95	1742.86	581.96	3	42.9	78.4	35.4	64.6	1.8
			97	FDTDELNFPTEK	iTRAQ4plex@N-term; Lys->Gln@12		0.0	1598.76	533.93	1598.72	533.91	3	8.8	8.4	51.4	48.6	0.9
			96	YQLSIHER	iTRAQ4plex@N-term		0.0	1188.62	397.21	1188.64	397.22	3	539.0	423.7	56.0	44.0	0.8
			90	AQDILAR	iTRAQ4plex@N-term		0.0	929.55	465.78	929.54	465.78	2	6860.3	5504.2	55.5	44.5	0.8
			90	LSLDELGR	iTRAQ4plex@N-term		1.0	1046.58	524.30	1045.59	523.80	2	1325.6	1279.0	50.9	49.1	1.0
			76	TGTLTQNR	iTRAQ4plex@N-term		0.1	1033.63	517.82	1033.56	517.79	2	428.7	536.3	44.4	55.6	1.3
			65	GLTNQR	iTRAQ4plex@N-term		0.0	831.52	416.77	831.47	416.74	2	58.0	64.7	47.3	52.7	1.1
			54	YQVDLSK	iTRAQ4plex@N-term; Lys->Gln@7		0.0	995.53	498.77	995.50	498.76	2	1210.6	773.5	61.0	39.0	0.6
			52	QGAIVAVTGDGVNDSPALKK	iTRAQ4plex@N-term; iTRAQ4plex(K)@19; Lys->Gln@20	missed K-K@19	0.1	2227.26	557.82	2227.21	557.81	4	668.7	527.9	55.9	44.1	0.8
			32	GVGIISEGNETVEDIAARLNIPVSQVNPR	iTRAQ4plex@N-term	missed R-L@18	-1.0	3189.73	798.44	3190.71	798.68	4					
			25	EVAMDDHK	iTRAQ4plex@N-term; Lys->Gln@8		0.1	1087.59	544.80	1087.47	544.74	2					
			23	TGTLTQNR	iTRAQ4plex@N-term		0.0	1033.61	517.81	1033.56	517.79	2					
			13	LIFDNLK	iTRAQ4plex@N-term; iTRAQ4plex(K)@7		0.0	1149.68	575.85	1149.70	575.86	2					
																Average	1.0
																Stdev	0.3
IPI:IPI00311682.5	ATP1A1	ATPase, Na+/K+ transporting, alpha 1	99	NMVPQQALVIR	iTRAQ4plex@N-term		0.0	1411.76	706.89	1411.81	706.91	2	5773.1	5610.0	50.7	49.3	1.0
			99	VDNSSLTGESEPQTR	iTRAQ4plex@N-term		0.0	1762.80	882.41	1762.85	882.43	2	1908.1	2113.1	47.5	52.5	1.1
			99	NMVPQQALVIR	iTRAQ4plex@N-term		0.0	1411.76	706.89	1411.81	706.91	2	1467.5	1502.7	49.4	50.6	1.0
			99	TSATWFALSR	iTRAQ4plex@N-term		0.0	1282.67	642.34	1282.68	642.35	2	1235.2	1800.1	40.7	59.3	1.5
			99	VDNSSLTGESEPQTR	iTRAQ4plex@N-term		0.0	1762.84	588.62	1762.85	588.62	3	360.6	346.2	51.0	49.0	1.0
			99	DMTSEELDDILR	iTRAQ4plex@N-term		0.0	1579.76	790.89	1579.75	790.88	2	278.4	341.7	44.9	55.1	1.2
			99	LSLDELHR	iTRAQ4plex@N-term		0.0	1125.61	376.21	1125.63	376.22	3	237.0	373.9	38.8	61.2	1.6
			99	GVGIISEGNETVEDIAAR	iTRAQ4plex@N-term		1.9	1974.97	659.33	1973.02	658.68	3	74.7	59.9	55.5	44.5	0.8
			99	GVGIISEGNETVEDIAAR	iTRAQ4plex@N-term		0.0	1973.04	658.69	1973.02	658.68	3	54.8	53.1	50.8	49.2	1.0
			99	GVGIISEGNETVEDIAAR	iTRAQ4plex@N-term		4.0	1977.03	660.02	1973.02	658.68	3	48.0	37.0	56.5	43.5	0.8
			99	GVGIISEGNETVEDIAAR	iTRAQ4plex@N-term		0.0	1972.98	658.67	1973.02	658.68	3	38.9	36.3	51.8	48.2	0.9
			99	NMVPQQALVIR	iTRAQ4plex@N-term; Deamidated(N)@1		0.0	1412.74	707.38	1412.79	707.40	2	186.8	195.5	48.9	51.1	1.0
			99	QGAIVAVTGDGVNDSPALK	iTRAQ4plex@N-term; Deamidated(Q)@1; Lys->Gln@19		0.1	1956.12	653.05	1955.99	653.00	3	52.8	57.4	47.9	52.1	1.1
			99	GVGIISEGNETVEDIAAR	iTRAQ4plex@N-term; Dimethyl(R)@18		0.1	2001.14	668.05	2001.05	668.02	3	20.6	36.1	36.4	63.6	1.8
			99	QGAIVAVTGDGVNDSPALK	iTRAQ4plex@N-term; iTRAQ4plex(K)@19		0.0	2099.16	700.73	2099.15	700.72	3	184.2	217.2	45.9	54.1	1.2
			99	AAVPDAVGK	iTRAQ4plex@N-term; iTRAQ4plex(K)@9		0.0	1114.69	558.35	1114.66	558.34	2	1607.5	2177.6	42.5	57.5	1.4
			99	VIMVTGDHPITAK	iTRAQ4plex@N-term; Lys->Gln@13		0.1	1524.88	509.30	1524.81	509.28	3	554.6	545.5	50.4	49.6	1.0
			99	IVEIPFNSTNK	iTRAQ4plex@N-term; No iTRAQ4plex(K)@11		0.0	1404.77	703.39	1404.77	703.39	2	241.0	216.3	52.7	47.3	0.9
			99	NLEAVETLGSTSTICSDK	iTRAQ4plex@N-term; Pyridylethyl(C)@15; iTRAQ4plex(K)@18		0.0	2260.17	754.40	2260.15	754.39	3	523.8	638.6	45.1	54.9	1.2
			99	NLEAVETLGSTSTICSDK	iTRAQ4plex@N-term; Pyridylethyl(C)@15; Lys->Gln@18		0.0	2116.05	706.36	2116.01	706.34	3	595.2	636.0	48.3	51.7	1.1
			99	NIAFFSTNCVEGTAR	iTRAQ4plex@N-term; Pyridylethyl(C)@9		-0.9	1877.00	626.67	1877.92	626.98	3	342.5	341.0	50.1	49.9	1.0
			76	AAEILAR	iTRAQ4plex@N-term		0.0	886.53	444.27	886.54	444.28	2	3740.9	3687.7	50.4	49.6	1.0
			76	TGTLTQNR	iTRAQ4plex@N-term		0.1	1033.63	517.82	1033.56	517.79	2	428.7	536.3	44.4	55.6	1.3
			52	QGAIVAVTGDGVNDSPALKK	iTRAQ4plex@N-term; iTRAQ4plex(K)@19; Lys->Gln@20	missed K-K@19	0.1	2227.26	557.82	2227.21	557.81	4	668.7	527.9	55.9	44.1	0.8
			34	GLTPAR	iTRAQ4plex@N-term		0.0	757.49	379.75	757.46	379.74	2					
			23	TGTLTQNR	iTRAQ4plex@N-term		0.0	1033.61	517.81	1033.56	517.79	2					
			13	LIFDNLK	iTRAQ4plex@N-term; iTRAQ4plex(K)@7		0.0	1149.68	575.85	1149.70	575.86	2					
																Average	1.1
																Stdev	0.2
IPI:IPI00121550.2	ATP1B1	ATPase, Na+/K+ transporting, beta 1	99	AYGENIGYSEK	iTRAQ4plex@N-term; iTRAQ4plex(K)@11		0.0	1517.78	759.90	1517.76	759.89	2	498.0	693.2	41.8	58.2	1.4
			99	SYEAYVLNIIR	iTRAQ4plex@N-term		0.0	1483.82	742.92	1483.82	742.92	2	1357.7	1202.5	53.0	47.0	0.9
			99	SYEAYVLNIIR	iTRAQ4plex@N-term		0.0	1483.81	742.91	1483.82	742.92	2	730.7	634.2	53.5	46.5	0.9
			99	SYEAYVLNIIR	iTRAQ4plex@N-term		4.0	1487.81	744.91	1483.82	742.92	2	555.9	559.6	49.8	50.2	1.0
			99	AYGENIGYSEK	iTRAQ4plex@N-term; Lys->Arg@11		0.0	1401.71	701.86	1401.66	701.84	2	367.9	448.8	45.0	55.0	1.2
			99	AYGENIGYSEK	iTRAQ4plex@N-term; Lys->Gln@11		0.0	1373.64	687.83	1373.62	687.82	2	1248.2	1143.7	52.2	47.8	0.9
			99	VAPPGLTQIPQIQK	iTRAQ4plex@N-term; Lys->Gln@14		0.0	1632.96	545.33	1632.93	545.32	3	150.9	127.4	54.2	45.8	0.8
			94	SYEAYVLNIIR	iTRAQ4plex@N-term		3.0	1486.79	744.40	1483.82	742.92	2	390.4	382.8	50.5	49.5	1.0

			72	DDMIFEDCGNVPSEPK	iTRAQ4plex@N-term; Pyridylethyl(C)@8; Lys->Gln@16		0.1	2043.94	682.32	2043.87	682.30	3	110.2	138.6	44.3	55.7	1.3
			39	TEISFRPNDPK	iTRAQ4plex@N-term; Lys->Gln@11		0.0	1446.75	483.26	1446.72	483.25	3					
			11	FIWNSEK	iTRAQ4plex@N-term; Lys->Gln@7		0.0	1066.54	534.28	1066.52	534.27	2					
																Average	1.0
																Stdev	0.2
IPI:IPI00553338.2	ATP1B2	ATPase, Na+/K+ transporting, beta 2	99	INAANIATDDER	iTRAQ4plex@N-term		0.0	1445.73	723.87	1445.72	723.87	2	838.2	1048.6	44.4	55.6	1.3
			99	INAANIATDDER	iTRAQ4plex@N-term		1.0	1446.72	724.37	1445.72	723.87	2	2318.7	2995.5	43.6	56.4	1.3
			99	YYEQPDNGVLNYPK	iTRAQ4plex@N-term; Deamidated(N)@7; iTRAQ4plex(K)@14		0.0	1987.98	663.67	1987.98	663.67	3	159.1	202.0	44.1	55.9	1.3
			21	YYEQPDNGVLNYPKR	iTRAQ4plex@N-term; Lys->Gln@14	missed K-R@14	0.0	1998.97	667.33	1998.96	667.33	3					
																Average	1.3
																Stdev	0.0
IPI:IPI00648173.1	CLTC	Clathrin, heavy polypeptide (Hc)	99	ALEHFTDLYDIK	iTRAQ4plex@N-term; iTRAQ4plex(K)@12		0.0	1751.93	584.98	1751.93	584.99	3	69.7	245.4	22.1	77.9	3.5
			99	IYIDSNNNPER	iTRAQ4plex@N-term		0.0	1477.70	739.86	1477.73	739.87	2	8454.2	8302.4	50.5	49.5	1.0
			99	ENPYYDSR	iTRAQ4plex@N-term		0.0	1186.55	594.28	1186.54	594.28	2	3722.2	4048.4	47.9	52.1	1.1
			99	IYIDSNNNPER	iTRAQ4plex@N-term		0.0	1477.75	739.88	1477.73	739.87	2	3486.1	2748.4	55.9	44.1	0.8
			99	LLYNNVSNFGR	iTRAQ4plex@N-term		0.0	1439.72	720.87	1439.76	720.89	2	3343.7	3489.6	48.9	51.1	1.0
			99	LLYNNVSNFGR	iTRAQ4plex@N-term		0.0	1439.73	720.87	1439.76	720.89	2	2825.2	2805.2	50.2	49.8	1.0
			99	NNLAGAEELFAR	iTRAQ4plex@N-term		0.0	1447.77	724.89	1447.75	724.88	2	2157.8	1692.6	56.0	44.0	0.8
			99	ENPYYDSR	iTRAQ4plex@N-term		0.0	1186.58	594.30	1186.54	594.28	2	1854.6	2824.8	39.6	60.4	1.5
			99	NNLAGAEELFAR	iTRAQ4plex@N-term		0.0	1447.74	724.88	1447.75	724.88	2	1226.8	979.2	55.6	44.4	0.8
			99	NNLAGAEELFAR	iTRAQ4plex@N-term		0.0	1447.77	724.89	1447.75	724.88	2	1085.0	805.4	57.4	42.6	0.7
			99	IYIDSNNNPER	iTRAQ4plex@N-term		1.0	1478.72	740.37	1477.73	739.87	2	663.6	568.3	53.9	46.1	0.9
			99	NNLAGAEELFAR	iTRAQ4plex@N-term		0.0	1447.75	724.88	1447.75	724.88	2	522.7	368.1	58.7	41.3	0.7
			99	LLLPWLEAR	iTRAQ4plex@N-term		0.0	1253.78	627.90	1253.76	627.89	2	333.9	326.6	50.6	49.4	1.0
			99	LASTLVHLGEYQAAVDGAF	iTRAQ4plex@N-term		0.0	2114.09	705.70	2114.12	705.72	3	261.5	353.1	42.6	57.4	1.3
			99	WLLLTGISAQQNR	iTRAQ4plex@N-term		0.0	1642.93	822.47	1642.93	822.47	2	69.5	100.3	40.9	59.1	1.4
			99	IVLDNSVFSEHR	iTRAQ4plex@N-term		0.0	1558.81	520.61	1558.82	520.61	3	64.9	75.5	46.3	53.7	1.2
			99	LAELEEFINGPNNAHIQQVGDF	iTRAQ4plex@N-term		0.0	2607.28	870.10	2607.32	870.11	3	46.3	84.9	35.3	64.7	1.8
			99	TSIDAYDNFDNISLAQR	iTRAQ4plex@N-term		0.0	2085.99	696.34	2086.01	696.34	3	20.9	28.4	42.4	57.6	1.4
			99	FDVNTSAVQVLIEHIGNLDF	iTRAQ4plex@N-term		-1.0	2382.29	795.11	2383.26	795.43	3	4.9	15.0	24.8	75.2	3.0
			99	FDVNTSAVQVLIEHIGNLDF	iTRAQ4plex@N-term		0.1	2383.31	795.44	2383.26	795.43	3	1.7	12.0	12.4	87.6	7.0
			99	NNLAGAEELFAR	iTRAQ4plex@N-term; Ala->Val@11		0.0	1475.76	738.89	1475.79	738.90	2	302.5	836.0	26.6	73.4	2.8
			99	NNLAGAEELFAR	iTRAQ4plex@N-term; Ala->Val@11		0.0	1475.77	738.89	1475.79	738.90	2	272.2	712.7	27.6	72.4	2.6
			99	IYIDSNNNPER	iTRAQ4plex@N-term; Dimethyl(R)@11		0.0	1505.78	753.90	1505.76	753.89	2	255.8	760.2	25.2	74.8	3.0
			99	EAIDSYIK	iTRAQ4plex@N-term; iTRAQ4plex(K)@8		0.0	1225.66	613.84	1225.68	613.85	2	1639.0	1918.4	46.1	53.9	1.2
			99	ALEHFTDLYDIKR	iTRAQ4plex@N-term; Lys->Gln@12	missed K-R@12	0.0	1763.89	441.98	1763.90	441.98	4	12.4	11.5	51.9	48.1	0.9
			99	QLPLVKPYLR	iTRAQ4plex@N-term; Lys->Gln@6		0.0	1369.83	457.62	1369.82	457.61	3	22.8	26.1	46.7	53.3	1.1
			99	TLQIFNIEMK	iTRAQ4plex@N-term; No iTRAQ4plex(K)@10		0.0	1379.76	690.89	1379.76	690.89	2	150.1	97.2	60.7	39.3	0.6
			99	ESNCYDPER	iTRAQ4plex@N-term; Pyridylethyl(C)@4		0.0	1360.61	454.54	1360.58	454.54	3	439.2	399.1	52.4	47.6	0.9
			98	TLQIFNIEMK	iTRAQ4plex@N-term; Lys->Gln@10		0.0	1379.77	690.89	1379.72	690.87	2	140.5	111.8	55.7	44.3	0.8
			97	EAIDSYIK	iTRAQ4plex@N-term; Lys->Gln@8		0.1	1081.60	541.80	1081.54	541.78	2	2505.5	1490.5	62.7	37.3	0.6
			96	NLQNLLILTAIK	iTRAQ4plex@N-term; Lys->Gln@12		0.0	1496.89	749.45	1496.90	749.46	2	47.6	50.3	48.6	51.4	1.1
			93	AYEFAER	iTRAQ4plex@N-term		0.0	1028.50	515.26	1028.50	515.26	2	4018.8	6392.4	38.6	61.4	1.6
			93	IYIDSNNNPER	iTRAQ4plex@N-term		-1.0	1476.74	739.38	1477.73	739.87	2	1546.2	1753.3	46.9	53.1	1.1
			93	LLLPWLEAR	iTRAQ4plex@N-term		-7.1	1246.65	624.33	1253.76	627.89	2	265.2	252.5	51.2	48.8	1.0
			92	NLQNLLILTAIK	iTRAQ4plex@N-term; iTRAQ4plex(K)@12		0.0	1641.02	548.01	1641.04	548.02	3	6.3	28.0	18.3	81.7	4.4
			92	IAAYLFK	iTRAQ4plex@N-term; iTRAQ4plex(K)@7		0.0	1112.70	557.36	1112.68	557.35	2	1033.8	1352.0	43.3	56.7	1.3
			91	LLLPWLEAR	iTRAQ4plex@N-term		1.0	1254.77	628.39	1253.76	627.89	2	223.9	232.1	49.1	50.9	1.0
			89	VGYTPDWIFLLR	iTRAQ4plex@N-term		-0.9	1621.98	812.00	1622.89	812.45	2	43.3	67.9	38.9	61.1	1.6
			88	VMEYINR	iTRAQ4plex@N-term		0.0	1067.53	534.77	1067.56	534.79	2	4181.4	6851.5	37.9	62.1	1.6
			88	DAMQYASESK	iTRAQ4plex@N-term; iTRAQ4plex(K)@10		0.0	1416.71	709.36	1416.68	709.35	2	64.4	140.5	31.4	68.6	2.2
			86	EHLELFWSR	iTRAQ4plex@N-term		0.0	1359.66	454.23	1359.71	454.24	3	43.1	73.1	37.1	62.9	1.7
			86	LLLPWLEAR	iTRAQ4plex@N-term; Dimethyl(R)@9		0.0	1281.84	641.93	1281.79	641.90	2	102.0	313.6	24.5	75.5	3.1
			84	DAMQYASESK	iTRAQ4plex@N-term; Lys->Gln@10		0.1	1272.62	637.32	1272.54	637.28	2	190.2	203.1	48.4	51.6	1.1
			84	NLQNLLILTAIK	iTRAQ4plex@N-term; Lys->Gln@12		0.1	1496.97	749.49	1496.90	749.46	2	35.1	41.0	46.1	53.9	1.2
			81	ESYVETELIFALAK	iTRAQ4plex@N-term; iTRAQ4plex(K)@14		0.0	1900.06	634.36	1900.04	634.36	3	1.7	4.4	28.1	71.9	2.6
			76	TLQIFNIEMK	iTRAQ4plex@N-term; iTRAQ4plex(K)@10		0.0	1523.87	508.96	1523.86	508.96	3	10.3	19.2	34.9	65.1	1.9
			76	NLQNLLILTAIK	iTRAQ4plex@N-term; Lys->Gln@12		0.0	1496.92	749.47	1496.90	749.46	2	48.3	80.7	37.4	62.6	1.7
			72	LLLPWLEAR	iTRAQ4plex@N-term		0.0	1253.79	627.90	1253.76	627.89	2	61.9	57.2	52.0	48.0	0.9
			71	AVNYFSK	iTRAQ4plex@N-term; iTRAQ4plex(K)@7		0.0	1115.61	558.81	1115.62	558.82	2	5821.6	7085.5	45.1	54.9	1.2
			46	YLQMAR	iTRAQ4plex@N-term		0.0	924.51	463.26	924.50	463.26	2					
			35	TLQIFNIEMK	iTRAQ4plex@N-term; Lys->Arg@10		0.0	1407.77	704.89	1407.77	704.89	2					
			28	AGLLQR	iTRAQ4plex@N-term		0.0	800.50	401.26	800.50	401.26	2					
			21	TLQIFNIEMK	iTRAQ4plex@N-term; Lys->Arg@10		0.0	1407.78	704.90	1407.77	704.89	2					
			17	AMLSANIR	iTRAQ4plex@N-term; Met->Ser@2; Deamidated(N)@6		0.0	975.58	488.80	975.55	488.78	2					
			13	IAAYLFK	iTRAQ4plex@N-term; Lys->Gln@7		0.0	968.57	485.29	968.55	485.28	2					
																Average	1.6
																Stdev	1.2
IPI:IPI00648927.1	CLTA	Clathrin, light polypeptide (Lca)	99	LEALDANSR	iTRAQ4plex@N-term		0.0	1131.57	566.79	1131.60	566.81	2	19597.3	15085.5	56.5	43.5	0.8
			99	LQSEPESIR	iTRAQ4plex@N-term		0.0	1201.66	601.84	1201.64	601.83	2	431.4	611.6	41.4	58.6	1.4
			99	VADEAFYK	iTRAQ4plex@N-term; iTRAQ4plex(K)@8		0.0	1229.64	615.83	1229.65	615.83	2	4496.1	6552.3	40.7	59.3	1.5
			98	LEALDANSR	iTRAQ4plex@N-term		0.0	1131.59	566.80	1131.60	566.81	2	1257.4	1292.8	49.3	50.7	1.0
			98	ELEEWYAR	iTRAQ4plex@N-term		0.0	1238.59	620.30	1238.61	620.31	2	604.2	540.1	52.8	47.2	0.9
			98	VADEAFYK	iTRAQ4plex@N-term; Lys->Gln@8		0.0	1085.54	543.78	1085.52	543.76	2	1219.9	1218.6	50.0	50.0	1.0
			97	LQSEPESIR	iTRAQ4plex@N-term		0.0	1201.64	601.83	1201.64	601.83	2	5696.0	8466.4	40.2	59.8	1.5
			85	QDEQLQK	iTRAQ4plex@N-term; iTRAQ4plex(K)@7		0.0	1175.67	588.84	1175.64	588.83	2	3394.8	2937.3	53.6	46.4	0.9
			68	QDEQLQK	iTRAQ4plex@N-term; Lys->Gln@7		0.1	1031.56	516.79	1031.50	516.76	2	7.0	8.4	45.4	54.6	1.2
			66	SVLISLK	iTRAQ4plex@N-term; iTRAQ4plex(K)@7		0.0	1046.68	524.35	1046.69	524.35	2	1264.7	1293.4	49.4	50.6	1.0
			65	SVLISLK	iTRAQ4plex@N-term; iTRAQ4plex(K)@7		0.0	1046.67	524.34	1046.69	524.35	2	3341.7	3194.1	51.1	48.9	1.0
			38	QAPLVH	iTRAQ4plex@N-term		0.0	807.48	404.75	807.47	404.74	2					
			22	ELEEWYAR	iTRAQ4plex@N-term; Dimethyl(R)@8		0.0	1266.65	634.33	1266.64	634.33	2					
																Average	1.1
																Stdev	0.3
IPI:IPI00228978.2	CLTB	Clathrin, light polypeptide (Lcb)	99	AFYQQPDADTIGYVASEEAFVK	iTRAQ4plex@N-term; iTRAQ4plex(K)@22		0.0	2736.32	913.11	2736.35	913.12	3	65.5	59.3	52.5	47.5	0.9
			99	LTQEPESIR	iTRAQ4plex@N-term		0.0	1215.65	608.83	1215.66	608.84	2	5384.4	7829.6	40.7	59.3	1.5
			99	DLEEWNQR	iTRAQ4plex@N-term		1.0	1233.58	617.80	1232.59	617.30	2	1906.4	1461.3	56.6	43.4	0.8
			99	LTQEPESIR	iTRAQ4plex@N-term		0.0	1216.68	609.35	1215.66	608.84	2	603.1	772.2	43.9	56.1	1.3
			99	LTQEPESIR	iTRAQ4plex@N-term		1.0	1216.69	609.35	1215.66	608.84	2	577.5	720.6	44.5	55.5	1.2
			99	EETPGTEWEK	iTRAQ4plex@N-term; iTRAQ4plex(K)@10		0.0	1492.74	747.38	1492.73	747.37	2	905.5	959.4	48.6	51.4	1.1
			99	AFYQQPDADTIGYVASEEAFVK	iTRAQ4plex@N-term; iTRAQ4plex(K)@22		-0.1	2736.27	913.10	2736.35	913.12	3	70.6	70.9	49.9	50.1	1.0
			99	AFYQQPDADTIGYVASEEAFVK	iTRAQ4plex@N-term; iTRAQ4plex(K)@22		0.0	2736.39	913.14	2736.35	913.12	3	10.7	8.3	56.3	43.7	0.8
			99	QSEQVEK	iTRAQ4plex@N-term; iTRAQ4plex(K)@7		-1.9	1132.69	567.35	1134.61	568.31	2	219.8	193.6	53.2	46.8	0.9
			99	QSEQVEK	iTRAQ4plex@N-term; iTRAQ4plex(K)@7		0.0	1134.66	568.34	1134.61	568.31	2	82.7	91.2	47.5	52.5	1.1
			99	LQELDAASK	iTRAQ4plex@N-term; iTRAQ4plex(K)@9		0.0	1261.73	631.87	1261.71	631.86	2	4654.3	4326.0	51.8	48.2	0.9
			99	EETPGTEWEK	iTRAQ4plex@N-term; Lys->Arg@10		0.0	1376.63	689.32	1376.63	689.32	2	587.5	748.1	44.0	56.0	1.3
			99	LQELDAASK	iTRAQ4plex@N-term; Lys->Arg@9		0.0	1145.63	573.82	1145.62	573.82	2	941.1	1585.6	37.2	62.8	1.7
			99	EETPGTEWEK	iTRAQ4plex@N-term; Lys->Gln@10		0.0	1348.64	675.32	1348.59	675.30	2	1862.3	1212.4	60.6	39.4	0.7
			86	EETPGTEWEK	iTRAQ4plex@N-term; Lys->Arg@10		0.0	1376.64	689.33	1376.63	689.32	2	718.0	982.8	42.2	57.8	1.4
			84	ASEEAFVK	iTRAQ4plex@N-term; Lys->Gln@8	cleaved V-A@N-term	0.1	1023.56	512.79	1023.50	512.76	2	242.0	236.9	50.5	49.5	1.0
			80	SVLMSLK	iTRAQ4plex@N-term; iTRAQ4plex(K)@7		0.0	1064.64	533.33	1064.65	533.33	2	1959.4	1962.9	50.0	50.0	1.0
			57	DLEEWNQR	iTRAQ4plex@N-term; Gln->Arg@7		0.0	1260.63	631.32	1260.63	631.32	2	1353.9	1955.9	40.9	59.1	1.4
			42	VTEQEWR	iTRAQ4plex@N-term; Deamidated(Q)@4		0.0	1091.55	546.78	1091.54	546.78	2					
			39	AFYQQPDADTIGYVASEEAFVK	iTRAQ4plex@N-term; Ala->Val@19; iTRAQ4plex(K)@22		0.0	2764.43	922.48	2764.38	922.47	3					
			39	LTQEPESIRK	iTRAQ4plex@N-term; Lys->Gln@10	missed R-K@9	0.1	1343.79	448.94	1343.72	448.91	3					
			14	DLEEWNQR	iTRAQ4plex@N-term; Oxidation(W)@5		0.0	1248.61	625.31	1248.59	625.30	2					
																Average	1.1
																Stdev	0.3
IPI:IPI00622235.6	VCP	Valosin-containing protein	99	EMVELPLR	iTRAQ4plex@N-term		0.0	1129.62	565.82	1129.63	565.82	2	1028.4	1501.5	40.6	59.4	1.5
			99	SVSDNDIR	iTRAQ4plex@N-term		0.1	1048.58	525.30	1048.53	525.27	2	1642.3	1376.3	54.4	45.6	0.8
			99	EVDIGIPDATGR	iTRAQ4plex@N-term		0.0	1385.74	693.88	1385.73	693.87	2	669.1	617.3	52.0	48.0	0.9
			99	GGNIGDGGGAADR	iTRAQ4plex@N-term		0.1	1259.68	630.85	1259.60	630.81	2	256.5	191.6	57.2	42.8	0.7
			99	EVDIGIPDATGR	iTRAQ4plex@N-term		0.0	1385.73	693.87	1385.73	693.87	2	253.5	223.4	53.2	46.8	0.9
			99	QTNPSAMEVEEDDPVPEIR	iTRAQ4plex@N-term		0.0	2299.04	767.35	2299.08	767.37	3	10.4	15.7	39.9	60.1	1.5
			99	LDQLIYIPLPDEK	iTRAQ4plex@N-term; iTRAQ4plex(K)@13		0.0	1844.05	615.69	1844.05	615.69	3	28.0	34.8	44.6	55.4	1.2
			99	VINQILTEMDGMSTK	iTRAQ4plex@N-term; iTRAQ4plex(K)@15		0.0	1967.05	656.69	1967.03	656.68	3	60.1	58.4	50.7	49.3	1.0
			99	NAPAIIFIDELDAIAPK	iTRAQ4plex@N-term; iTRAQ4plex(K)@17		0.1	2098.25	700.43	2098.19	700.40	3	31.6	37.9	45.4	54.6	1.2
			99	NAPAIIFIDELDAIAPK	iTRAQ4plex@N-term; iTRAQ4plex(K)@17		0.1	2098.26	700.43	2098.19	700.40	3	22.8	27.4	45.4	54.6	1.2
			99	ETVVEVPQVTWEDIGGLEDVKR	iTRAQ4plex@N-term; iTRAQ4plex(K)@21	missed K-R@21	0.0	2785.50	697.38	2785.47	697.38	4	19.0	31.8	37.4	62.6	1.7
			99	NAPAIIFIDELDAIAPK	iTRAQ4plex@N-term; Lys->Gln@17		0.0	1954.08	652.37	1954.05	652.36	3	14.3	9.2	60.8	39.2	0.6
			99	NAPAIIFIDELDAIAPK	iTRAQ4plex@N-term; Lys->Gln@17		0.0	1954.08	652.37	1954.05	652.36	3	10.6	9.4	53.2	46.8	0.9
			99	LIVDEAINEDNSVVSLSQPK	iTRAQ4plex@N-term; Lys->Gln@20		0.0	2313.17	772.06	2313.18	772.07	3	21.4	16.6	56.3	43.7	0.8
			99	GPELLTMWFGESEANVR	iTRAQ4plex@N-term; Oxidation(F)@9		0.0	2095.01	699.34	2095.02	699.35	3	14.3	10.3	58.0	42.0	0.7
			99	VINQILTEMDGMSTK	iTRAQ4plex@N-term; Oxidation(M)@9; Lys->Gln@15		0.1	1838.95	613.99	1838.89	613.97	3	5.4	3.1	63.8	36.2	0.6
			99	MTNGFSGADLTEICQR	iTRAQ4plex@N-term; Pyridylethyl(C)@14		0.0	1990.89	664.64	1990.94	664.65	3	258.9	226.3	53.4	46.6	0.9
			98	ESIESEIR	iTRAQ4plex@N-term		0.0	1105.61	553.81	1105.57	553.79	2	672.4	779.0	46.3	53.7	1.2
			98	MDELQLFR	iTRAQ4plex@N-term		0.0	1194.61	598.31	1194.62	598.32	2	316.0	266.4	54.3	45.7	0.8
			98	LDQLIYIPLPDEK	iTRAQ4plex@N-term; Lys->Gln@13		0.1	1699.98	567.67	1699.92	567.65	3	1.7	3.3	34.3	65.7	1.9
			96	LDQLIYIPLPDEK	iTRAQ4plex@N-term; iTRAQ4plex(K)@13		0.0	1844.04	615.69	1844.05	615.69	3	29.8	34.6	46.3	53.7	1.2
			94	DHFEEAMR	iTRAQ4plex@N-term		0.0	1177.52	393.52	1177.53	393.52	3	364.0	303.5	54.5	45.5	0.8
			74	QAAPCVLFFDELDSIAK	iTRAQ4plex@N-term; Pyridylethyl(C)@5; Glu->Gln@11; Lys->Gln@17		0.1	2114.14	705.72	2114.06	705.69	3	43.1	21.2	67.1	32.9	0.5
			48	DVDLEFLAK	iTRAQ4plex@N-term; Lys->Gln@9		0.0	1192.64	597.33	1192.61	597.31	2					
			46	VINQILTEMDGMSTK	iTRAQ4plex@N-term; Lys->Gln@15		0.0	1822.93	608.65	1822.89	608.64	3					
			28	IVSQLLTLMDGLK	iTRAQ4plex@N-term; iTRAQ4plex(K)@13		0.0	1718.03	573.68	1718.03	573.68	3					
			24	NAPAIIFIDELDAIAPK	iTRAQ4plex@N-term; Lys->Arg@17		0.1	1982.19	661.74	1982.10	661.71	3					
			24	NAPAIIFIDELDAIAPK	iTRAQ4plex@N-term; Lys->Gln@17		0.1	1954.18	652.40	1954.05	652.36	3					
			23	GPELLTMWFGESEANVR	iTRAQ4plex@N-term; Leu->Asn@5; Oxidation(F)@9		0.1	2096.03	699.69	2095.98	699.67	3					
			15	NAPAIIFIDELDAIAPK	iTRAQ4plex@N-term; Lys->Arg@17		0.1	1982.19	661.74	1982.10	661.71	3					
			11	GFGSFR	iTRAQ4plex@N-term		0.0	813.43	407.72	813.43	407.72	2					
																Average	1.0
																Stdev	0.4
IPI:IPI00316682.2	GOLGA5	Golgin A5	99	ANYISSAADNIF	iTRAQ4plex@N-term		0.0	1437.75	719.88	1437.73	719.87	2	577.3	325.9	63.9	36.1	0.6
			99	TLSNSSQSELESR	iTRAQ4plex@N-term		0.0	1580.81	791.41	1580.78	791.40	2	2361.1	1337.1	63.8	36.2	0.6
			99	TLSNSSQSELESR	iTRAQ4plex@N-term		0.0	1580.75	791.38	1580.78	791.40	2	2309.1	1157.0	66.6	33.4	0.5
			99	QMQSEFAAR	iTRAQ4plex@N-term		1.0	1211.60	606.81	1210.59	606.30	2	1430.1	775.3	64.8	35.2	0.5
			99	NSLVFQLER	iTRAQ4plex@N-term		0.0	1248.67	625.34	1248.69	625.35	2	1128.7	661.2	63.1	36.9	0.6
			99	YMEEDLHR	iTRAQ4plex@N-term		1.0	1236.59	413.20	1235.57	412.86	3	376.9	250.9	60.0	40.0	0.7
			99	EGSSLQNQALQTLQER	iTRAQ4plex@N-term		-0.1	1944.94	649.32	1945.00	649.34	3	95.6	88.3	52.0	48.0	0.9
			99	EGSSLQNQALQTLQER	iTRAQ4plex@N-term		0.0	1945.00	649.34	1945.00	649.34	3	79.9	66.2	54.7	45.3	0.8
			99	NEVQSLNQEMASLLQR	iTRAQ4plex@N-term		0.0	2003.03	668.68	2003.02	668.68	3	49.5	46.7	51.5	48.5	0.9
			99	SELQDMEAQQVSEAESAF	iTRAQ4plex@N-term		0.0	2151.02	718.01	2150.99	718.00	3	34.2	16.1	68.0	32.0	0.5
			99	NEVQSLNQEMASLLQR	iTRAQ4plex@N-term		0.0	2002.99	668.67	2003.02	668.68	3	26.3	31.4	45.5	54.5	1.2
			99	SEPDDELLFDFLNSSQK	iTRAQ4plex@N-term; iTRAQ4plex(K)@17		0.0	2271.11	758.04	2271.12	758.05	3	8.1	27.9	22.5	77.5	3.4
			99	SEPDDELLFDFLNSSQK	iTRAQ4plex@N-term; iTRAQ4plex(K)@17		0.1	2271.18	758.07	2271.12	758.05	3	1.7	13.0	11.5	88.5	7.7
			99	NVPVLFNDTETNLAGMYGK	iTRAQ4plex@N-term; iTRAQ4plex(K)@19		0.0	2370.25	791.09	2370.21	791.08	3	89.0	69.2	56.3	43.7	0.8
			99	ATILAGTANVK	iTRAQ4plex@N-term; Lys->Gln@11		0.1	1201.75	601.88	1201.68	601.85	2	380.5	253.7	60.0	40.0	0.7
			99	NVPVLFNDTETNLAGMYGK	iTRAQ4plex@N-term; Lys->Gln@19		0.1	2226.17	743.06	2226.07	743.03	3	12.6	5.0	71.8	28.2	0.4
			99	QTMLESLSTEK	iTRAQ4plex@N-term; No iTRAQ4plex(K)@11		0.0	1409.70	705.86	1409.72	705.87	2	582.3	340.8	63.1	36.9	0.6
			99	NEVQSLNQEMASLLQR	iTRAQ4plex@N-term; Oxidation(M)@10		0.0	2019.00	674.01	2019.02	674.01	3	46.5	28.6	61.9	38.1	0.6
			99	SELQDMEAQQVSEAESAF	iTRAQ4plex@N-term; Ser->Asp@16		0.0	2179.00	727.34	2178.98	727.33	3	26.1	37.0	41.4	58.6	1.4
			97	QELETELER	iTRAQ4plex@N-term		0.0	1289.67	645.84	1289.66	645.84	2	6356.7	3663.2	63.4	36.6	0.6
			97	TEALEALR	iTRAQ4plex@N-term		1.0	1046.60	524.31	1045.59	523.80	2	2366.7	1232.7	65.8	34.2	0.5
			97	NSLVFQLER	iTRAQ4plex@N-term		1.0	1249.68	625.85	1248.69	625.35	2	2071.8	1141.2	64.5	35.5	0.6
			95	EQESYK	iTRAQ4plex@N-term; iTRAQ4plex(K)@6		0.1	1070.62	536.32	1070.55	536.28	2	326.4	180.5	64.4	35.6	0.6
			94	SQELSNLR	iTRAQ4plex@N-term		0.0	1089.62	545.82	1089.59	545.80	2	3956.1	2455.7	61.7	38.3	0.6
			94	ETQEELNK	iTRAQ4plex@N-term; Lys->Gln@8		0.1	1133.62	567.82	1133.53	567.77	2	21.3	17.7	54.7	45.3	0.8
			80	QELETELER	iTRAQ4plex@N-term		0.0	1289.67	645.84	1289.66	645.84	2	6902.6	4098.5	62.7	37.3	0.6
			47	QELVDYK	iTRAQ4plex@N-term; iTRAQ4plex(K)@7		0.0	1181.65	591.83	1181.65	591.83	2					
			33	MEVDR	iTRAQ4plex@N-term		0.0	792.44	397.23	792.39	397.20	2					
																Average	1.1
																Stdev	1.5
IPI:IPI00130013.72	TFG	Trk-fused gene	99	LTLFVNGQPR	iTRAQ4plex@N-term	cleaved R-P@C-term	0.0	1287.72	644.87	1287.74	644.88	2	2643.3	5427.7	32.8	67.2	2.1
			99	LTLFVNGQPR	iTRAQ4plex@N-term	cleaved R-P@C-term	0.0	1287.76	644.89	1287.74	644.88	2	717.7	1138.9	38.7	61.3	1.6
			99	NRPPFGQGYAQPGPGY	iTRAQ4plex@N-term	cleaved Y-R@C-term	-0.1	1848.84	617.29	1848.90	617.31	3	442.5	357.7	55.3	44.7	0.8
			99	LLDSLEPPGEPGPSTSIPENDTVDGR	iTRAQ4plex@N-term		0.9	2836.31	946.45	2835.39	946.14	3	436.1	385.9	53.1	46.9	0.9
			99	GLTDDQVSGPPSAPTEDR	iTRAQ4plex@N-term	cleaved F-G@N-term	0.0	1984.94	662.65	1984.95	662.66	3	190.6	191.6	49.9	50.1	1.0
			99	NVMSAFGLTDDQVSGPPSAPTEDR	iTRAQ4plex@N-term		0.0	2634.22	879.08	2634.24	879.09	3	164.4	158.1	51.0	49.0	1.0
			99	NVMSAFGLTDDQVSGPPSAPTEDR	iTRAQ4plex@N-term		0.0	2634.26	879.09	2634.24	879.09	3	157.7	139.9	53.0	47.0	0.9
			99	GLTDDQVSGPPSAPTEDR	iTRAQ4plex@N-term	cleaved F-G@N-term	0.0	1984.95	662.66	1984.95	662.66	3	152.2	140.2	52.1	47.9	0.9
			99	SAFGLTDDQVSGPPSAPTEDR	iTRAQ4plex@N-term	cleaved M-S@N-term	0.0	2290.07	764.36	2290.08	764.37	3	108.6	182.8	37.3	62.7	1.7
			99	SAFGLTDDQVSGPPSAPTEDR	iTRAQ4plex@N-term	cleaved M-S@N-term	0.0	2290.08	764.37	2290.08	764.37	3	80.3	157.0	33.8	66.2	2.0
			99	IPIHNEDITYDELVLMMQR	iTRAQ4plex@N-term		0.0	2473.28	825.43	2473.25	825.42	3	45.5	55.7	45.0	55.0	1.2
			99	IPIHNEDITYDELVLMMQR	iTRAQ4plex@N-term		0.1	2473.31	825.44	2473.25	825.42	3	40.8	35.3	53.6	46.4	0.9
			99	IPIHNEDITYDELVLMMQR	iTRAQ4plex@N-term		0.0	2473.26	825.43	2473.25	825.42	3	39.1	31.0	55.8	44.2	0.8
			99	NVMSAFGLTDDQVSGPPSAPTEDR	iTRAQ4plex@N-term		0.0	2634.20	879.07	2634.24	879.09	3	34.9	46.2	43.0	57.0	1.3
			99	LLDSLEPPGEPGPSTSIPENDTVDGR	iTRAQ4plex@N-term		0.0	2835.36	946.13	2835.39	946.14	3	19.2	24.2	44.3	55.7	1.3
			99	LLDSLEPPGEPGPSTSIPENDTVDGR	iTRAQ4plex@N-term		0.0	2835.44	946.15	2835.39	946.14	3	15.9	14.6	52.2	47.8	0.9
			99	LLDSLEPPGEPGPSTSIPENDTVDGR	iTRAQ4plex@N-term		0.0	2835.42	946.15	2835.39	946.14	3	10.3	19.2	34.9	65.1	1.9
			99	NVMSAFGLTDDQVSGPPSAPTEDR	iTRAQ4plex@N-term		0.0	2634.27	659.57	2634.24	659.57	4	1.7	13.0	11.5	88.5	7.7
			99	NVMSAFGLTDDQVSGPPSAPTEDR	iTRAQ4plex@N-term; Cation:K(D)@11		-0.1	2672.12	669.04	2672.19	669.06	4	33.5	34.4	49.3	50.7	1.0
			99	NVMSAFGLTDDQVSGPPSAPTEDR	iTRAQ4plex@N-term; Cation:K(D)@11		0.0	2672.19	669.06	2672.19	669.06	4	32.0	25.8	55.4	44.6	0.8
			99	NVMSAFGLTDDQVSGPPSAPTEDR	iTRAQ4plex@N-term; Cation:K(D)@11		0.0	2672.18	669.05	2672.19	669.06	4	23.0	21.9	51.2	48.8	1.0
			99	LLDSLEPPGEPGPSTSIPENDTVDGR	iTRAQ4plex@N-term; Dehydrated(D)@24		0.0	2817.33	940.12	2817.38	940.13	3	15.6	23.3	40.1	59.9	1.5
			99	LLDSLEPPGEPGPSTSIPENDTVDGR	iTRAQ4plex@N-term; Dehydrated(E)@10		0.1	2817.43	940.15	2817.38	940.13	3	14.2	11.4	55.5	44.5	0.8
			99	NVMSAFGLTDDQVSGPPSAPTEDR	iTRAQ4plex@N-term; Dehydrated(S)@14		0.0	2616.26	873.09	2616.22	873.08	3	12.0	21.3	36.1	63.9	1.8
			99	LLDSLEPPGEPGPSTSIPENDTVDGR	iTRAQ4plex@N-term; Dimethyl(R)@26		0.0	2863.40	955.47	2863.42	955.48	3	62.5	142.6	30.5	69.5	2.3
			99	LLDSLEPPGEPGPSTSIPENDTVDGR	iTRAQ4plex@N-term; Dimethyl(R)@26		0.0	2863.39	955.47	2863.42	955.48	3	45.5	57.8	44.0	56.0	1.3
			99	LLDSLEPPGEPGPSTSIPENDTVDGR	iTRAQ4plex@N-term; Dimethyl(R)@26		0.1	2863.49	955.50	2863.42	955.48	3	4.5	25.9	14.9	85.1	5.7
			99	IPIHNEDITYDELVLMMQR	iTRAQ4plex@N-term; Gln->Arg@18		0.0	2501.31	834.78	2501.29	834.77	3	1.7	6.5	20.7	79.3	3.8
			99	LLSNDEVTIK	iTRAQ4plex@N-term; iTRAQ4plex(K)@10		0.0	1418.83	710.42	1418.82	710.42	2	1519.4	1835.3	45.3	54.7	1.2
			99	QSTQVMAASMSAFDPLK	iTRAQ4plex@N-term; iTRAQ4plex(K)@17		0.0	2099.02	700.68	2099.06	700.70	3	154.8	269.2	36.5	63.5	1.7
			99	QSTQVMAASMSAFDPLK	iTRAQ4plex@N-term; iTRAQ4plex(K)@17		0.0	2099.08	700.70	2099.06	700.70	3	79.1	139.9	36.1	63.9	1.8
			99	QSTQVMAASMSAFDPLKNQDEIN	iTRAQ4plex@N-term; iTRAQ4plex(K)@17; Deamidated(N)@18; Dehydrated(E)@21; Deamidated(N)@23	cleaved N-K@C-term; missed K-N@17	0.0	2796.35	700.10	2796.32	700.09	4	54.8	52.0	51.3	48.7	0.9
			99	QSTQVMAASMSAFDPLKNQDEINK	iTRAQ4plex@N-term; iTRAQ4plex(K)@17; iTRAQ4plex(K)@24	missed K-N@17	-1.1	3083.48	617.70	3084.56	617.92	5	427.8	410.8	51.0	49.0	1.0
			99	QSTQVMAASMSAFDPLKNQDEINK	iTRAQ4plex@N-term; iTRAQ4plex(K)@17; iTRAQ4plex(K)@24	missed K-N@17	0.1	3084.61	772.16	3084.56	772.15	4	270.4	260.7	50.9	49.1	1.0
			99	QSTQVMAASMSAFDPLKNQDEINK	iTRAQ4plex@N-term; iTRAQ4plex(K)@17; Lys->Gln@24	missed K-N@17	0.0	2940.45	736.12	2940.42	736.11	4	152.4	136.1	52.8	47.2	0.9
			99	QSTQVMAASMSAFDPLKNQDEINK	iTRAQ4plex@N-term; iTRAQ4plex(K)@17; Lys->Gln@24	missed K-N@17	0.0	2940.38	736.10	2940.42	736.11	4	60.5	47.6	56.0	44.0	0.8
			99	QSTQVMAASMSAFDPLKNQDEINK	iTRAQ4plex@N-term; iTRAQ4plex(K)@17; Lys->Gln@24	missed K-N@17	0.0	2940.40	736.11	2940.42	736.11	4	51.0	55.4	48.0	52.0	1.1
			99	NQDEINK	iTRAQ4plex@N-term; iTRAQ4plex(K)@7		0.1	1147.66	574.84	1147.61	574.81	2	303.6	703.7	30.1	69.9	2.3
			99	IPIHNEDITYDELVLMMQR	iTRAQ4plex@N-term; Leu->Gln@13		0.1	2488.32	830.45	2488.22	830.41	3	18.9	32.9	36.5	63.5	1.7
			99	QSTQVMAASMSAFDPLK	iTRAQ4plex@N-term; Lys->Gln@17		0.0	1954.96	652.66	1954.93	652.65	3	75.8	127.9	37.2	62.8	1.7
			99	QSTQVMAASMSAFDPLK	iTRAQ4plex@N-term; Lys->Gln@17		0.0	1954.91	652.64	1954.93	652.65	3	58.2	110.3	34.5	65.5	1.9
			99	QSTQVMAASMSAFDPLK	iTRAQ4plex@N-term; Lys->Gln@17		0.0	1954.94	652.65	1954.93	652.65	3	20.8	32.9	38.7	61.3	1.6
			99	QSTQVMAASMSAFDPLKNQDEINK	iTRAQ4plex@N-term; Lys->Gln@17; Lys->Gln@24	missed K-N@17	0.1	2796.35	700.10	2796.28	700.08	4	12.3	12.6	49.6	50.4	1.0
			99	NVMSAFGLTDDQVSGPPSAPTEDR	iTRAQ4plex@N-term; Oxidation(D)@11; Deamidated(Q)@12		0.0	2651.22	884.75	2651.21	884.75	3	86.1	96.4	47.2	52.8	1.1
			99	NVMSAFGLTDDQVSGPPSAPTEDR	iTRAQ4plex@N-term; Oxidation(D)@11; Deamidated(Q)@12		0.1	2651.29	884.77	2651.21	884.75	3	76.9	97.7	44.0	56.0	1.3
			99	NVMSAFGLTDDQVSGPPSAPTEDR	iTRAQ4plex@N-term; Oxidation(D)@11; Dimethyl(R)@24		0.0	2678.26	893.76	2678.26	893.76	3	17.6	68.7	20.4	79.6	3.9
			99	NVMSAFGLTDDQVSGPPSAPTEDR	iTRAQ4plex@N-term; Oxidation(M)@3		-0.1	2650.17	884.40	2650.23	884.42	3	81.2	82.4	49.6	50.4	1.0
			99	NVMSAFGLTDDQVSGPPSAPTEDR	iTRAQ4plex@N-term; Oxidation(M)@3		-0.1	2650.16	884.39	2650.23	884.42	3	58.2	60.6	49.0	51.0	1.0
			99	NVMSAFGLTDDQVSGPPSAPTEDR	iTRAQ4plex@N-term; Oxidation(M)@3		0.0	2650.21	884.41	2650.23	884.42	3	17.4	24.3	41.7	58.3	1.4
			99	NVMSAFGLTDDQVSGPPSAPTEDR	iTRAQ4plex@N-term; Oxidation(M)@3; Dimethyl(R)@24		0.0	2678.24	893.75	2678.26	893.76	3	10.0	25.6	28.2	71.8	2.5
			99	QSTQVMAASMSAFDPLK	iTRAQ4plex@N-term; Oxidation(M)@6; iTRAQ4plex(K)@17		0.0	2115.03	706.02	2115.06	706.03	3	45.1	66.5	40.4	59.6	1.5
			99	QSTQVMAASMSAFDPLK	iTRAQ4plex@N-term; Oxidation(M)@6; Lys->Gln@17		0.1	1970.98	658.00	1970.92	657.98	3	31.2	46.6	40.1	59.9	1.5
			99	QSTQVMAASMSAFDPLK	iTRAQ4plex@N-term; Oxidation(M)@6; Lys->Gln@17		0.1	1970.98	658.00	1970.92	657.98	3	24.1	42.5	36.1	63.9	1.8
			99	LLDSLEPPGEPGPSTSIPENDTVDGR	iTRAQ4plex@N-term; Oxidation(P)@13; Phospho(T)@15		0.1	2931.47	978.16	2931.35	978.12	3	13.7	24.5	36.0	64.0	1.8
			99	NVMSAFGLTDDQVSGPPSAPTEDR	iTRAQ4plex@N-term; Ser->Trp@14		0.0	2733.31	912.11	2733.28	912.10	3	24.3	36.9	39.6	60.4	1.5
			99	NVMSAFGLTDDQVSGPPSAPTEDR	iTRAQ4plex@N-term; Val->Trp@13		0.1	2721.35	908.12	2721.25	908.09	3	29.4	45.4	39.3	60.7	1.5
			98	AQLGEDIR	iTRAQ4plex@N-term		0.0	1044.52	523.27	1044.57	523.29	2	30328.5	22671.7	57.2	42.8	0.7
			98	LLDSLEPPGEPGPSTSIPENDTVDGR	iTRAQ4plex@N-term		0.0	2835.35	709.84	2835.39	709.85	4	99.2	88.3	52.9	47.1	0.9
			97	QSTQVMAASMSAFDPLK	iTRAQ4plex@N-term; Oxidation(M)@6; Oxidation(M)@10; iTRAQ4plex(K)@17		0.0	2131.02	711.35	2131.05	711.36	3	37.9	63.5	37.4	62.6	1.7
			96	QSTQVMAASMSAFDPLK	iTRAQ4plex@N-term; Methyl(D)@14; Methyl(L)@16; iTRAQ4plex(K)@17		0.0	2127.10	710.04	2127.09	710.04	3	40.5	93.8	30.1	69.9	2.3
			95	NQDEINK	iTRAQ4plex@N-term; iTRAQ4plex(K)@7		0.1	1147.68	574.84	1147.61	574.81	2	260.1	743.1	25.9	74.1	2.9
			88	NQDEINK	iTRAQ4plex@N-term; iTRAQ4plex(K)@7		0.0	1147.65	574.83	1147.61	574.81	2	1313.7	3855.0	25.4	74.6	2.9
			87	QSTQVMAASMSAFDPLKNQDEINK	iTRAQ4plex@N-term; Lys->Gln@17; Lys->Arg@24	missed K-N@17	0.0	2824.36	942.46	2824.32	942.45	3	34.2	66.9	33.8	66.2	2.0
			73	EEKPAASDSSGK	iTRAQ4plex@N-term; Lys->Glu@3; iTRAQ4plex(K)@12		0.1	1493.78	498.93	1493.71	498.91	3	304.7	323.7	48.5	51.5	1.1
			43	EEKPAASDSSGK	iTRAQ4plex@N-term; Glu->Asn@1; Glu->Pro@2; iTRAQ4plex(K)@3; iTRAQ4plex(K)@12		0.0	1589.86	530.96	1589.87	530.97	3					
			33	NVMSAFGLTDDQVSGPPSAPTEDR	iTRAQ4plex@N-term; Thr->His@9; Oxidation(D)@11		-0.1	2686.11	672.53	2686.24	672.57	4					
			30	ELVELR	iTRAQ4plex@N-term		0.0	901.50	451.76	901.54	451.78	2					
			28	ELVELR	iTRAQ4plex@N-term		0.0	901.53	451.77	901.54	451.78	2					
			20	EEKPAASDSSGK	iTRAQ4plex@N-term; iTRAQ4plex(K)@3; Dehydrated(S)@7; Oxidation(D)@8; Lys->Gln@12		0.1	1490.79	497.94	1490.71	497.91	3					
			10	NQDEINK	iTRAQ4plex@N-term; Deamidated(Q)@2; Lys->Gln@7		0.1	1004.52	503.27	1004.45	503.23	2					
																Average	1.6
																Stdev	1.1
IPI:IPI00831568.1	L1CAM	L1 cell adhesion molecule	99	AFGAPVPSVQWLDEEGTTVLQDER	iTRAQ4plex@N-term		0.1	2787.48	930.17	2787.38	930.14	3	1.7	10.9	13.5	86.5	6.4
			99	EATQITQGPR	iTRAQ4plex@N-term		0.0	1243.67	622.84	1243.66	622.84	2	10206.6	5670.3	64.3	35.7	0.6
			99	EATQITQGPR	iTRAQ4plex@N-term		0.0	1243.67	622.84	1243.66	622.84	2	2316.6	1553.2	59.9	40.1	0.7
			99	WMDWNAPQIQYR	iTRAQ4plex@N-term		0.0	1750.84	876.43	1750.84	876.43	2	497.0	268.4	64.9	35.1	0.5
			99	LSPYVHYTFR	iTRAQ4plex@N-term		0.0	1425.73	476.25	1425.75	476.26	3	164.7	98.8	62.5	37.5	0.6
			99	WMDWNAPQIQYR	iTRAQ4plex@N-term		0.0	1750.79	584.61	1750.84	584.62	3	119.7	127.6	48.4	51.6	1.1
			99	AQLLVVGSPGPVPHLELSDF	iTRAQ4plex@N-term		0.0	2227.22	743.41	2227.24	743.42	3	71.3	50.4	58.6	41.4	0.7
			99	AFGAPVPSVQWLDEEGTTVLQDER	iTRAQ4plex@N-term		0.0	2787.40	930.14	2787.38	930.14	3	43.1	22.3	65.9	34.1	0.5
			99	YRIEQGSLILSNVQPSDTMVTQCEAR	iTRAQ4plex@N-term; Deamidated(Q)@14; Pyridylethyl(C)@23	missed R-I@2	0.0	3187.53	797.89	3187.58	797.90	4	12.4	10.4	54.5	45.5	0.8
			99	AFGAPVPSVQWLDEEGTTVLQDER	iTRAQ4plex@N-term; Glu->Gln@15; Dimethyl(R)@24		0.2	2814.58	939.20	2814.43	939.15	3	1.7	6.5	20.7	79.3	3.8
			99	LVVFPTDDISLK	iTRAQ4plex@N-term; iTRAQ4plex(K)@12		0.0	1633.97	817.99	1633.95	817.98	2	364.0	255.2	58.8	41.2	0.7
			99	LVVFPTDDISLK	iTRAQ4plex@N-term; iTRAQ4plex(K)@12		0.0	1633.93	545.65	1633.95	545.66	3	85.5	64.9	56.8	43.2	0.8
			99	YGPGEPSPVSETVVTPEAAPEKNPVDVR	iTRAQ4plex@N-term; iTRAQ4plex(K)@22	missed K-N@22	0.0	3208.69	803.18	3208.65	803.17	4	176.8	119.6	59.7	40.3	0.7
			99	LVVFPTDDISLK	iTRAQ4plex@N-term; Lys->Gln@12		0.0	1489.83	745.92	1489.81	745.91	2	331.4	194.6	63.0	37.0	0.6
			99	LVVFPTDDISLK	iTRAQ4plex@N-term; Lys->Gln@12		0.1	1489.87	745.94	1489.81	745.91	2	159.5	90.3	63.9	36.1	0.6
			99	LVVFPTDDISLK	iTRAQ4plex@N-term; Lys->Gln@12		0.1	1489.89	745.95	1489.81	745.91	2	141.9	121.7	53.8	46.2	0.9
			99	RLVVFPTDDISLK	iTRAQ4plex@N-term; Lys->Gln@13	missed R-L@1	0.0	1645.96	549.66	1645.92	549.65	3	18.6	41.4	31.0	69.0	2.2
			99	QSQVHLSWSPAEDHNSPIEK	iTRAQ4plex@N-term; Lys->Gln@20		0.0	2432.15	609.04	2432.15	609.04	4	346.4	334.3	50.9	49.1	1.0
			99	QSQVHLSWSPAEDHNSPIEK	iTRAQ4plex@N-term; Lys->Gln@20		0.0	2432.15	609.04	2432.15	609.04	4	250.3	210.0	54.4	45.6	0.8
			99	YDIEFEDKEMAPEK	iTRAQ4plex@N-term; Lys->Gln@8; Lys->Gln@14		0.1	1886.91	629.98	1886.80	629.94	3	98.4	60.0	62.1	37.9	0.6
			99	YDIEFEDKEMAPEK	iTRAQ4plex@N-term; Lys->Gln@8; Lys->Gln@14		0.1	1886.87	629.96	1886.80	629.94	3	84.6	37.8	69.1	30.9	0.4
			99	TLQLLNVGEEDDGEYTCLAENSLGSAR	iTRAQ4plex@N-term; Pyridylethyl(C)@17		0.0	3145.52	787.39	3145.50	787.38	4	12.4	11.5	51.9	48.1	0.9
			99	IEQGSLILSNVQPSDTMVTQCEAR	iTRAQ4plex@N-term; Pyridylethyl(C)@21		0.0	2867.38	717.85	2867.43	717.86	4	65.1	72.3	47.4	52.6	1.1
			99	IEQGSLILSNVQPSDTMVTQCEAR	iTRAQ4plex@N-term; Pyridylethyl(C)@21		-0.1	2867.33	717.84	2867.43	717.86	4	61.9	57.4	51.9	48.1	0.9
			98	YDIEFEDK	iTRAQ4plex@N-term; iTRAQ4plex(K)@8		0.0	1345.66	673.84	1345.66	673.84	2	688.9	1226.7	36.0	64.0	1.8
			97	FQLQATTQQGPGEAIVR	iTRAQ4plex@N-term; Dimethyl(R)@17		0.0	2015.08	672.70	2015.09	672.70	3	30.4	69.2	30.5	69.5	2.3
			96	FQLQATTQQGPGEAIVR	iTRAQ4plex@N-term		-0.1	1987.01	663.34	1987.06	663.36	3	293.4	183.9	61.5	38.5	0.6
			96	FQLQATTQQGPGEAIVR	iTRAQ4plex@N-term		0.0	1987.01	663.35	1987.06	663.36	3	284.4	178.9	61.4	38.6	0.6
			94	LVVFPTDDISLK	iTRAQ4plex@N-term; iTRAQ4plex(K)@12		-5.0	1628.93	543.98	1633.95	545.66	3	17.6	20.0	46.8	53.2	1.1
			94	YGPGEPSPVSETVVTPEAAPEK	iTRAQ4plex@N-term; iTRAQ4plex(K)@22		0.0	2528.31	843.78	2528.29	843.77	3	49.0	60.9	44.6	55.4	1.2
			89	IYWMNSK	iTRAQ4plex@N-term; iTRAQ4plex(K)@7		0.0	1228.66	615.34	1228.65	615.33	2	3145.6	2096.5	60.0	40.0	0.7
			84	YGPGEPSPVSETVVTPEAAPEKNPVDVR	iTRAQ4plex@N-term; Lys->Gln@22	missed K-N@22	-0.1	3064.46	767.12	3064.51	767.14	4	62.0	54.7	53.2	46.8	0.9
			83	FQLQATTQQGPGEAIVR	iTRAQ4plex@N-term; Dimethyl(R)@17		0.0	2015.09	672.71	2015.09	672.70	3	32.2	69.0	31.9	68.1	2.1
			48	WMDWNAPQIQYR	iTRAQ4plex@N-term; Dimethyl(R)@12		0.0	1778.90	593.97	1778.87	593.96	3					
			42	VTAINK	iTRAQ4plex@N-term; iTRAQ4plex(K)@6		0.0	932.62	467.32	932.59	467.30	2					
			39	FQGIYR	iTRAQ4plex@N-term		0.0	926.50	464.26	926.51	464.26	2					
			37	YDIEFEDK	iTRAQ4plex@N-term; Lys->Gln@8		0.0	1201.55	601.78	1201.53	601.77	2					
			24	ALPEGK	iTRAQ4plex@N-term; Lys->Gln@6		0.1	757.50	379.76	757.41	379.71	2					
			18	LVVFPTDDISLK	iTRAQ4plex@N-term; Lys->Arg@12		0.0	1517.84	759.93	1517.86	759.94	2					
			16	VQAVNNQGK	iTRAQ4plex@N-term; Lys->Gln@9		0.1	1100.65	551.33	1100.57	551.29	2					
			12	IYWMNSK	iTRAQ4plex@N-term; Lys->Gln@7		0.0	1084.54	543.28	1084.51	543.26	2					
			11	VQAVNNQGK	iTRAQ4plex@N-term; Lys->Gln@9		0.1	1100.67	551.34	1100.57	551.29	2					
																Average	1.2
																Stdev	1.2
IPI:IPI00122971.1	NCAM1	Neural cell adhesion molecule 1	99	AATTNPSQGEDLK	iTRAQ4plex@N-term; Lys->Gln@13		0.1	1474.76	738.39	1474.70	738.36	2	57.3	184.2	23.7	76.3	3.2
			99	QDDGGSPIR	iTRAQ4plex@N-term		0.0	1087.59	544.80	1087.54	544.78	2	88.2	188.4	31.9	68.1	2.1

			99	IGQESLEFILVQADTPSSPSIDF	iTRAQ4plex@N-term		0.0	2645.40	882.81	2645.37	882.80	3	1.7	5.4	23.8	76.2	3.2
			99	DESKEPIVEVR	iTRAQ4plex@N-term; iTRAQ4plex(K)@4		0.0	1587.92	530.31	1587.87	530.30	3	159.6	339.3	32.0	68.0	2.1
			99	TVPNDATQTK	iTRAQ4plex@N-term; Lys->Gln@10		0.1	1217.68	609.85	1217.60	609.81	2	413.5	852.4	32.7	67.3	2.1
			99	SLGEESWHFK	iTRAQ4plex@N-term; Lys->Gln@10		0.0	1362.63	455.22	1362.63	455.22	3	72.2	127.2	36.2	63.8	1.8
			99	LPSGSDHVMLK	iTRAQ4plex@N-term; Lys->Gln@11		0.0	1326.67	443.23	1326.67	443.23	3	157.0	359.1	30.4	69.6	2.3
			99	GLGEISAATEFK	iTRAQ4plex@N-term; Lys->Gln@12		0.0	1365.74	683.88	1365.69	683.85	2	249.5	333.8	42.8	57.2	1.3
			99	GLGEISAATEFK	iTRAQ4plex@N-term; Lys->Gln@12		0.0	1365.73	683.87	1365.69	683.85	2	219.6	299.5	42.3	57.7	1.4
			99	LEGQMGEDGNSIK	iTRAQ4plex@N-term; Lys->Gln@13		0.0	1520.72	761.37	1520.69	761.35	2	494.0	1003.4	33.0	67.0	2.0
			99	LEGQMGEDGNSIK	iTRAQ4plex@N-term; Lys->Gln@13		0.1	1520.75	761.38	1520.69	761.35	2	425.2	932.3	31.3	68.7	2.2
			99	GVTASSSSPASAPK	iTRAQ4plex@N-term; Lys->Gln@14		0.1	1389.75	695.88	1389.69	695.85	2	40.2	51.6	43.8	56.2	1.3
			99	EANMEGIVTIMGLKPETR	iTRAQ4plex@N-term; No iTRAQ4plex(K)@14		0.0	2132.15	711.72	2132.11	711.71	3	17.0	36.3	31.9	68.1	2.1
			99	EANMEGIVTIMGLKPETR	iTRAQ4plex@N-term; Oxidation(M)@4; Lys->Gln@14		0.0	2148.04	717.02	2148.07	717.03	3	6.0	35.6	14.5	85.5	5.9
			97	FIVLSNNYLQIR	iTRAQ4plex@N-term		0.0	1622.96	541.99	1622.93	541.98	3	15.5	26.6	36.9	63.1	1.7
			96	DESKEPIVEVR	iTRAQ4plex@N-term; iTRAQ4plex(K)@4; Dimethyl(R)@11		0.0	1615.86	539.63	1615.90	539.64	3	91.7	295.4	23.7	76.3	3.2
			96	VSSLTLK	iTRAQ4plex@N-term; iTRAQ4plex(K)@7		0.0	1034.68	518.35	1034.66	518.34	2	1121.3	3261.8	25.6	74.4	2.9
			94	QDDGGSPIR	iTRAQ4plex@N-term		0.0	1087.56	544.79	1087.54	544.78	2	11.7	31.0	27.3	72.7	2.7
			86	TQPVREPSAPK	iTRAQ4plex@N-term; Lys->Gln@11	missed R-E@5	0.1	1352.80	451.94	1352.72	451.91	3	158.1	230.0	40.7	59.3	1.5
			81	DMEEGK	iTRAQ4plex@N-term; iTRAQ4plex(K)@6		0.0	995.53	498.77	995.48	498.75	2	22.1	95.5	18.8	81.2	4.3
			74	LSPNQQR	iTRAQ4plex@N-term		0.0	985.58	493.80	985.54	493.78	2	31.2	97.4	24.3	75.7	3.1
			71	QEAPSTK	iTRAQ4plex@N-term; iTRAQ4plex(K)@7		0.0	1047.61	524.81	1047.58	524.80	2	42.9	76.4	36.0	64.0	1.8
			35	GEINFK	iTRAQ4plex@N-term; iTRAQ4plex(K)@6		0.0	994.56	498.29	994.57	498.29	2					
			15	IGQESLEFILVQADTPSSPSIDF	iTRAQ4plex@N-term; Deamidated(Q)@12; Ser->Leu@20		0.1	2672.47	891.83	2672.40	891.81	3					
																Average	2.5
																Stdev	1.1
IPI:IPI00329927.3	NFASC	Neurofascin	99	AYLTVLADQATPTNR	iTRAQ4plex@N-term		0.0	1776.93	593.32	1776.95	593.32	3	38.2	57.2	40.0	60.0	1.5
			99	EVAGDTIIFR	iTRAQ4plex@N-term		0.0	1263.69	632.85	1263.69	632.85	2	590.3	1071.5	35.5	64.5	1.8
			99	DLELTDLAER	iTRAQ4plex@N-term		0.0	1317.66	659.84	1317.69	659.85	2	380.9	640.8	37.3	62.7	1.7
			99	DLELTDLAER	iTRAQ4plex@N-term		0.0	1317.69	659.85	1317.69	659.85	2	354.1	573.6	38.2	61.8	1.6
			99	VIAVNEVGSSHPSLPSEF	iTRAQ4plex@N-term		0.0	2021.08	674.70	2021.07	674.70	3	253.6	420.3	37.6	62.4	1.7
			99	DLELTDLAER	iTRAQ4plex@N-term		0.0	1317.70	659.86	1317.69	659.85	2	168.7	291.4	36.7	63.3	1.7
			99	DDEPLYIGNR	iTRAQ4plex@N-term		0.0	1334.66	668.34	1334.66	668.34	2	167.8	414.0	28.8	71.2	2.5
			99	DDEPLYIGNR	iTRAQ4plex@N-term		0.0	1334.67	668.34	1334.66	668.34	2	131.8	348.1	27.5	72.5	2.6
			99	YVVGQTPVYVPYEIF	iTRAQ4plex@N-term		0.0	1926.07	643.03	1926.04	643.02	3	32.4	64.8	33.3	66.7	2.0
			99	TPSFMYPQGTSSSQMVLR	iTRAQ4plex@N-term		0.0	2160.01	721.01	2160.05	721.02	3	25.6	150.6	14.5	85.5	5.9
			99	TPSFMYPQGTSSSQMVLR	iTRAQ4plex@N-term		0.0	2160.02	721.01	2160.05	721.02	3	22.8	76.0	23.1	76.9	3.3
			99	YVVGQTPVYVPYEIF	iTRAQ4plex@N-term; Dimethyl(R)@15		0.0	1954.08	652.37	1954.07	652.36	3	1.7	6.5	20.7	79.3	3.8
			96	TSGAPPESNPSDVK	iTRAQ4plex@N-term; No iTRAQ4plex(K)@14		0.0	1528.77	765.39	1528.75	765.38	2	22.6	82.6	21.5	78.5	3.7
																Average	2.6
																Stdev	1.3
IPI:IPI00123058.1	CNTN1	Contactin-1	99	DGEYVVEVR	iTRAQ4plex@N-term		0.0	1208.64	605.33	1208.62	605.32	2	330.8	361.9	47.8	52.2	1.1
			99	TDGAAPNVAPSDVGGGGGTNR	iTRAQ4plex@N-term		0.0	2012.99	672.01	2012.96	672.00	3	14.2	12.5	53.2	46.8	0.9
			98	FIPLIPIPER	iTRAQ4plex@N-term		0.0	1337.82	669.92	1337.82	669.92	2	249.1	245.1	50.4	49.6	1.0
			93	VQVTSQEYSAR	iTRAQ4plex@N-term		0.0	1410.76	706.39	1410.72	706.37	2	142.9	146.3	49.4	50.6	1.0
			84	FIPLIPIPER	iTRAQ4plex@N-term		0.0	1337.81	669.91	1337.82	669.92	2	228.2	263.2	46.4	53.6	1.2
																Average	1.0
																Stdev	0.1
IPI:IPI00403536.1	NRCAM	Neuron-glia-CAM-related cell adhesion molecule	99	DNGELPNDER	iTRAQ4plex@N-term		0.1	1301.66	651.84	1301.60	651.81	2	301.6	358.1	45.7	54.3	1.2
			99	DNGELPNDER	iTRAQ4plex@N-term		0.0	1301.61	651.81	1301.60	651.81	2	182.0	226.0	44.6	55.4	1.2
			98	YIVSGTPTFVPYLIK	iTRAQ4plex@N-term; iTRAQ4plex(K)@15		0.0	1985.14	662.72	1985.15	662.72	3	10.6	11.5	47.8	52.2	1.1
			36	QPEYAVVQR	iTRAQ4plex@N-term		0.0	1232.69	617.35	1232.66	617.34	2					
																Average	1.2
																Stdev	0.1
IPI:IPI00321348.3	IGSF8	Immunoglobulin superfamily member 8	99	AIPEAPVGR	iTRAQ4plex@N-term		0.0	1052.61	527.31	1052.61	527.31	2	1130.7	3055.6	27.0	73.0	2.7
			99	SDMAVEAGAPYAER	iTRAQ4plex@N-term		0.0	1609.78	805.90	1609.75	805.88	2	136.0	383.5	26.2	73.8	2.8
			99	LVAQLDTEGIGSLGPGYEDR	iTRAQ4plex@N-term		0.0	2233.13	745.39	2233.13	745.39	3	10.4	17.0	37.9	62.1	1.6
			99	LVAQLDTEGIGSLGPGYEDR	iTRAQ4plex@N-term; Glu->Gln@8; Dimethyl(R)@20		0.0	2260.21	754.41	2260.18	754.40	3	1.7	10.9	13.5	86.5	6.4
			94	QAATSPSR	iTRAQ4plex@N-term		0.1	960.57	481.29	960.51	481.26	2	57.8	170.0	25.4	74.6	2.9
																Average	3.3
																Stdev	1.8
IPI:IPI00750142.1	SYT1	Synaptotagmin 1	99	MDVGGLSDPYVK	iTRAQ4plex@N-term; Lys->Gln@12		0.0	1423.68	712.85	1423.68	712.85	2	276.6	841.7	24.7	75.3	3.0
			99	VFVGYNSTGAELR	iTRAQ4plex@N-term		0.0	1555.76	778.89	1555.81	778.91	2	365.3	1467.5	19.9	80.1	4.0
			99	VFVGYNSTGAELR	iTRAQ4plex@N-term		0.0	1555.77	778.89	1555.81	778.91	2	239.3	912.1	20.8	79.2	3.8
			99	TLVMAVYDFDR	iTRAQ4plex@N-term		0.0	1472.77	737.39	1472.75	737.38	2	189.6	1019.8	15.7	84.3	5.4
			99	TLVMAVYDFDR	iTRAQ4plex@N-term		0.0	1472.75	737.38	1472.75	737.38	2	110.2	538.5	17.0	83.0	4.9
			99	VPMNTVDFGHVTEEWR	iTRAQ4plex@N-term		1.1	2061.08	688.03	2059.99	687.67	3	65.1	220.8	22.8	77.2	3.4
			99	VFVGYNSTGAELR	iTRAQ4plex@N-term; Dimethyl(R)@13		0.0	1583.86	792.94	1583.84	792.93	2	43.2	320.2	11.9	88.1	7.4
			99	TLNPVFNEQFTFK	iTRAQ4plex@N-term; iTRAQ4plex(K)@13		0.0	1871.99	625.00	1872.00	625.01	3	116.6	315.2	27.0	73.0	2.7
			99	MDVGGLSDPYVK	iTRAQ4plex@N-term; Lys->Arg@12		0.0	1451.76	726.89	1451.72	726.87	2	55.4	485.3	10.3	89.7	8.8
			99	MDVGGLSDPYVK	iTRAQ4plex@N-term; Lys->Gln@12		0.0	1423.70	712.86	1423.68	712.85	2	344.5	1038.2	24.9	75.1	3.0
			98	MDVGGLSDPYVK	iTRAQ4plex@N-term; iTRAQ4plex(K)@12		0.0	1567.80	523.61	1567.82	523.61	3	67.3	162.0	29.3	70.7	2.4
			44	VFLLPDK	iTRAQ4plex@N-term; iTRAQ4plex(K)@7		0.0	1118.66	560.34	1118.69	560.35	2					
			38	DLQSAEK	iTRAQ4plex@N-term; Lys->Gln@7		0.1	933.53	467.77	933.45	467.73	2					
			31	TLNPVFNEQFTFK	iTRAQ4plex@N-term; iTRAQ4plex(T)@11; Lys->Arg@13		0.0	1900.04	634.35	1900.01	634.34	3					
			26	YVPTAGK	iTRAQ4plex@N-term; iTRAQ4plex(K)@7		0.0	1022.58	512.30	1022.60	512.31	2					
			12	VFLLPDK	iTRAQ4plex@N-term; Lys->Gln@7		0.0	974.59	488.30	974.56	488.29	2					
																Average	4.4
																Stdev	2.0
IPI:IPI00129622.3	SYT2	Synaptotagmin 2	99	IFVGSNATGTELR	iTRAQ4plex@N-term		0.0	1507.83	754.92	1507.81	754.91	2	470.2	1001.5	32.0	68.0	2.1
			99	TLVMAIYDFDR	iTRAQ4plex@N-term		0.0	1486.75	744.38	1486.76	744.39	2	111.8	394.4	22.1	77.9	3.5
			99	TLVMAIYDFDR	iTRAQ4plex@N-term		0.0	1486.78	744.40	1486.76	744.39	2	83.4	320.7	20.6	79.4	3.8
			99	VPMNTVDLGQPIEEWR	iTRAQ4plex@N-term		0.0	2027.01	676.68	2027.03	676.68	3	25.0	17.5	58.8	41.2	0.7
			99	MDVGGLSDPYVK	iTRAQ4plex@N-term; Lys->Arg@12		0.0	1451.76	726.89	1451.72	726.87	2	55.4	485.3	10.3	89.7	8.8
			99	MDVGGLSDPYVK	iTRAQ4plex@N-term; Lys->Gln@12		0.0	1423.70	712.86	1423.68	712.85	2	344.5	1038.2	24.9	75.1	3.0
			99	MDVGGLSDPYVK	iTRAQ4plex@N-term; Lys->Gln@12		0.0	1423.68	712.85	1423.68	712.85	2	276.6	841.7	24.7	75.3	3.0
			98	MDVGGLSDPYVK	iTRAQ4plex@N-term; iTRAQ4plex(K)@12		0.0	1567.80	523.61	1567.82	523.61	3	67.3	162.0	29.3	70.7	2.4
			44	VFLLPDK	iTRAQ4plex@N-term; iTRAQ4plex(K)@7		0.0	1118.66	560.34	1118.69	560.35	2					
			26	YVPTAGK	iTRAQ4plex@N-term; iTRAQ4plex(K)@7		0.0	1022.58	512.30	1022.60	512.31	2					
			12	VFLLPDK	iTRAQ4plex@N-term; Lys->Gln@7		0.0	974.59	488.30	974.56	488.29	2					
																Average	3.4
																Stdev	2.4
IPI:IPI00113149.1	STX1B2	Syntaxin 1B	99	AIEQSIEQEEGLNR	iTRAQ4plex@N-term		0.0	1758.88	880.45	1758.89	880.45	2	223.6	690.5	24.5	75.5	3.1
			99	AIEQSIEQEEGLNR	iTRAQ4plex@N-term		0.0	1758.88	587.30	1758.89	587.30	3	166.2	609.2	21.4	78.6	3.7
			99	FVEVMTEYNATQSK	iTRAQ4plex@N-term; iTRAQ4plex(K)@14		0.0	1933.95	645.66	1933.97	645.66	3	93.7	239.0	28.2	71.8	2.6
			99	TTTNEELEDMLESGK	iTRAQ4plex@N-term; iTRAQ4plex(K)@15		0.0	1983.92	662.31	1983.96	662.33	3	140.9	200.6	41.3	58.7	1.4
			99	TTTNEELEDMLESGK	iTRAQ4plex@N-term; iTRAQ4plex(K)@15		0.0	1983.92	662.31	1983.96	662.33	3	59.1	135.5	30.4	69.6	2.3
			99	TTTNEELEDMLESGK	iTRAQ4plex@N-term; iTRAQ4plex(K)@15		0.0	1983.91	662.31	1983.96	662.33	3	54.2	118.3	31.4	68.6	2.2
			99	TTTNEELEDMLESGK	iTRAQ4plex@N-term; iTRAQ4plex(K)@15		-0.9	1983.01	662.01	1983.96	662.33	3	39.4	73.2	35.0	65.0	1.9
			99	TTTNEELEDMLESGK	iTRAQ4plex@N-term; iTRAQ4plex(K)@15		0.0	1983.93	662.32	1983.96	662.33	3	23.2	66.2	25.9	74.1	2.9
			99	FVEVMTEYNATQSK	iTRAQ4plex@N-term; Lys->Gln@14		0.0	1789.86	597.63	1789.83	597.62	3	13.0	43.8	22.9	77.1	3.4
			99	TTTNEELEDMLESGK	iTRAQ4plex@N-term; Lys->Gln@15		0.1	1839.94	614.32	1839.82	614.28	3	13.7	26.6	33.9	66.1	1.9
			98	MDSQMTK	iTRAQ4plex@N-term; iTRAQ4plex(K)@7		0.0	1127.57	564.79	1127.56	564.79	2	915.2	2994.9	23.4	76.6	3.3
			97	TTTNEELEDMLESGK	iTRAQ4plex@N-term; iTRAQ4plex(K)@15		-4.9	1979.03	660.68	1983.96	662.33	3	44.3	90.3	32.9	67.1	2.0
			97	TTTNEELEDMLESGK	iTRAQ4plex@N-term; iTRAQ4plex(S)@13; Lys->Arg@15		0.0	2011.96	671.66	2011.96	671.66	3	21.3	67.5	24.0	76.0	3.2
			93	QLEITGR	iTRAQ4plex@N-term		0.0	959.55	480.78	959.55	480.78	2	3979.9	11505.8	25.7	74.3	2.9
			88	LAIFTDDIK	iTRAQ4plex@N-term; Lys->Arg@9		0.0	1206.71	604.36	1206.67	604.34	2	44.3	238.9	15.7	84.3	5.4
			83	AVSDTK	iTRAQ4plex@N-term; iTRAQ4plex(K)@6		0.0	907.57	454.79	907.52	454.77	2	19.8	58.9	25.2	74.8	3.0
																Average	2.8
																Stdev	0.9
IPI:IPI00131618.1	STX1A	Syntaxin 1A	99	FVEVMSEYNATQSDYR	iTRAQ4plex@N-term		0.0	2081.92	694.98	2081.95	694.99	3	20.9	29.6	41.4	58.6	1.4
			99	SIEQSIEQEEGLNR	iTRAQ4plex@N-term		0.0	1774.88	592.63	1774.88	592.63	3	29.1	54.1	35.0	65.0	1.9
			93	QLEITGR	iTRAQ4plex@N-term		0.0	959.55	480.78	959.55	480.78	2	3979.9	11505.8	25.7	74.3	2.9
			83	AVSDTK	iTRAQ4plex@N-term; iTRAQ4plex(K)@6		0.0	907.57	454.79	907.52	454.77	2	19.8	58.9	25.2	74.8	3.0
																Average	2.3
																Stdev	0.8
IPI:IPI00415403.1	STXBP1	Syntaxin-binding protein 1	99	ISEQTYQLSR	iTRAQ4plex@N-term		0.0	1367.70	684.86	1367.72	684.87	2	559.2	621.1	47.4	52.6	1.1
			99	SQLLILDR	iTRAQ4plex@N-term		0.0	1100.65	551.33	1100.67	551.34	2	496.9	724.3	40.7	59.3	1.5
			99	YETSGIGEAR	iTRAQ4plex@N-term		0.0	1225.62	613.81	1225.61	613.81	2	364.0	655.7	35.7	64.3	1.8
			99	SSASFSTTAVSAR	iTRAQ4plex@N-term		1.0	1415.71	708.86	1414.72	708.37	2	305.8	393.7	43.7	56.3	1.3
			99	YETSGIGEAR	iTRAQ4plex@N-term		0.0	1225.61	613.81	1225.61	613.81	2	301.0	574.6	34.4	65.6	1.9
			96	LAEQIATLCATLK	iTRAQ4plex@N-term; Pyridylethyl(C)@9; Lys->Gln@13		0.0	1622.90	541.97	1622.88	541.97	3	10.3	18.1	36.3	63.7	1.8
			88	AVVGEK	iTRAQ4plex@N-term; iTRAQ4plex(K)@6		0.0	889.56	445.79	889.55	445.78	2	155.1	213.0	42.1	57.9	1.4
																Average	1.5
																Stdev	0.3
IPI:IPI00229703.6	VAMP2	Vesicle-associated membrane protein 2	99	ADALQAGASQFETSAAK	iTRAQ4plex@N-term; iTRAQ4plex(K)@17		-0.1	1952.95	651.99	1953.00	652.01	3	58.2	161.2	26.5	73.5	2.8
			99	LQQTQAQVDEVVDIMR	iTRAQ4plex@N-term		0.0	2016.04	673.02	2016.04	673.02	3	9.6	36.6	20.9	79.1	3.8
			99	LQQTQAQVDEVVDIMR	iTRAQ4plex@N-term		0.0	2016.01	673.01	2016.04	673.02	3	6.5	23.6	21.5	78.5	3.7
			99	LQQTQAQVDEVVDIMR	iTRAQ4plex@N-term; Dimethyl(R)@16		0.0	2044.07	682.36	2044.07	682.37	3	1.7	45.7	3.6	96.4	26.9
			44	LSELDDR	iTRAQ4plex@N-term		0.0	990.53	496.27	990.51	496.26	2					
																Average	9.3
																Stdev	11.7
IPI:IPI00125635.1	SNAP25	Synaptosomal-associated protein 25	99	AWGNNQDGVVASQPAF	iTRAQ4plex@N-term		0.0	1812.89	605.30	1812.90	605.31	3	102.1	160.7	38.8	61.2	1.6
			99	EQMAISGGFIR	iTRAQ4plex@N-term		0.0	1351.68	676.85	1351.70	676.86	2	787.7	1577.1	33.3	66.7	2.0
			99	NELEEMQR	iTRAQ4plex@N-term		0.0	1191.60	596.81	1191.57	596.79	2	700.1	3621.1	16.2	83.8	5.2
			99	TLVMLDEQGEQLER	iTRAQ4plex@N-term		0.0	1803.92	602.32	1803.92	602.31	3	25.7	47.7	35.0	65.0	1.9
			99	EQMAISGGFIR	iTRAQ4plex@N-term; Dimethyl(R)@11		0.0	1379.74	690.88	1379.74	690.87	2	64.5	488.5	11.7	88.3	7.6
			99	AWGNNQDGVVASQPAF	iTRAQ4plex@N-term; Trioxidation(W)@2		0.0	1860.92	621.32	1860.88	621.30	3	70.4	126.0	35.8	64.2	1.8
			96	IDEANQR	iTRAQ4plex@N-term		0.1	988.56	495.29	988.51	495.26	2	61.8	160.0	27.9	72.1	2.6
			65	EQMAISGGFIR	iTRAQ4plex@N-term		0.0	1351.69	676.85	1351.70	676.86	2	100.7	2649.9	3.7	96.3	26.3
			43	EQMAISGGFIR	iTRAQ4plex@N-term		0.0	1351.68	676.85	1351.70	676.86	2					
																Average	6.1
																Stdev	8.4
IPI:IPI00115824.1	NIPSNAP1	4-nitrophenylphosphatase domain and SNAP25-like protein homolog 1	99	AGPNIYELR	iTRAQ4plex@N-term		0.0	1175.62	588.82	1175.64	588.83	2	2680.0	2573.4	51.0	49.0	1.0
			99	GWDENVYYTVPLVR	iTRAQ4plex@N-term		0.0	1853.92	618.98	1853.94	618.99	3	28.4	23.9	54.3	45.7	0.8
			99	GWDENVYYTVPLVR	iTRAQ4plex@N-term		0.0	1853.91	618.98	1853.94	618.99	3	26.9	15.3	63.8	36.2	0.6
			99	GWDENVYYTVPLVR	iTRAQ4plex@N-term; Dimethyl(R)@14		0.0	1881.97	628.33	1881.97	628.33	3	1.7	3.3	34.3	65.7	1.9
			99	FSGGYPALMDCMNK	iTRAQ4plex@N-term; Pyridylethyl(C)@11; Lys->Gln@14		0.1	1781.83	594.95	1781.77	594.93	3	101.4	129.1	44.0	56.0	1.3
			79	GWDENVYYTVPLVR	iTRAQ4plex@N-term		0.0	1853.90	618.98	1853.94	618.99	3	5.3	4.1	56.3	43.7	0.8
			11	ETSNLYK	iTRAQ4plex@N-term; Lys->Gln@7		0.1	997.56	499.78	997.48	499.75	2					
																Average	1.1
																Stdev	0.5
IPI:IPI00762323.1	SV2A	Synaptic vesicle glycoprotein 2A	99	EELAQQYETILR	iTRAQ4plex@N-term		0.0	1635.86	818.94	1635.86	818.94	2	72.7	113.1	39.1	60.9	1.6
			99	MADGAPLAGVR	iTRAQ4plex@N-term		0.0	1200.66	601.34	1200.64	601.33	2	1955.7	2264.5	46.3	53.7	1.2
			99	MADGAPLAGVR	iTRAQ4plex@N-term		0.0	1200.66	601.34	1200.64	601.33	2	611.0	704.2	46.5	53.5	1.2
			81	VQDEYSR	iTRAQ4plex@N-term		1.0	1040.54	521.28	1039.51	520.76	2	1798.7	2354.3	43.3	56.7	1.3
																Average	1.3
																Stdev	0.2
IPI:IPI00221456.1	SV2B	Synaptic vesicle glycoprotein 2B	98	HVLFEDTFFDK	iTRAQ4plex@N-term; Lys->Gln@11		0.0	1540.76	514.59	1540.73	514.58	3	42.4	90.5	31.9	68.1	2.1
			72	FLLEMGK	iTRAQ4plex@N-term; iTRAQ4plex(K)@7		0.0	1124.65	563.33	1124.65	563.33	2	404.7	1491.8	21.3	78.7	3.7
																Average	2.9
																Stdev	1.1
IPI:IPI00109870.1	NAPG	N-ethylmaleimide-sensitive factor attachment protein, gamma	99	AVQLYQQTANVFENEER	iTRAQ4plex@N-term		0.0	2182.05	728.36	2182.08	728.37	3	52.1	76.2	40.6	59.4	1.5
			99	AVQLYQQTANVFENEER	iTRAQ4plex@N-term		0.0	2182.07	728.37	2182.08	728.37	3	16.7	43.7	27.6	72.4	2.6
			98	EAVAHENNR	iTRAQ4plex@N-term		1.0	1183.62	395.55	1182.59	395.20	3	5.1	9.6	34.9	65.1	1.9
																Average	2.0
																Stdev	0.6
IPI:IPI00125992.1	NPTX1	Neuronal pentraxin-1	99	TPAAETLSQLGQTLQSLK	iTRAQ4plex@N-term; iTRAQ4plex(K)@18		0.0	2173.20	725.41	2173.22	725.41	3	8.5	17.1	33.3	66.7	2.0
			99	TPAAETLSQLGQTLQSLK	iTRAQ4plex@N-term; iTRAQ4plex(K)@18		-1.0	2172.22	725.08	2173.22	725.41	3	1.7	10.9	13.5	86.5	6.4
			99	TPAAETLSQLGQTLQSLK	iTRAQ4plex@N-term; Lys->Gln@18		0.0	2029.13	677.38	2029.08	677.37	3	1.7	6.5	20.7	79.3	3.8
			78	TPAAETLSQLGQTLQSLK	iTRAQ4plex@N-term; iTRAQ4plex(K)@18		0.1	2173.34	725.45	2173.22	725.41	3	5.1	9.6	34.9	65.1	1.9
			28	FQLTFPLR	iTRAQ4plex@N-term		0.0	1164.69	583.35	1164.68	583.35	2					
																Average	3.5
																Stdev	2.1
IPI:IPI00263013.4	PLP1	Proteolipid protein 1	99	GLSATVTGGQK	iTRAQ4plex@N-term; Lys->Gln@11		0.0	1161.62	581.82	1161.61	581.81	2	1686.0	9283.1	15.4	84.6	5.5
			99	GLSATVTGGQK	iTRAQ4plex@N-term; iTRAQ4plex(K)@11		0.0	1305.77	436.26	1305.75	436.26	3	4091.0	7820.3	34.3	65.7	1.9
			99	GLSATVTGGQK	iTRAQ4plex@N-term; iTRAQ4plex(K)@11		0.0	1305.76	436.26	1305.75	436.26	3	491.1	1682.3	22.6	77.4	3.4
			99	MYGVLPWNAFPGK	iTRAQ4plex@N-term; iTRAQ4plex(K)@13		-0.1	1766.86	589.96	1766.94	589.99	3	639.1	5387.3	10.6	89.4	8.4
			99	MYGVLPWNAFPGK	iTRAQ4plex@N-term; iTRAQ4plex(K)@13		1.0	1767.96	590.33	1766.94	589.99	3	11.1	47.2	19.0	81.0	4.3
			99	LIETYFSK	iTRAQ4plex@N-term; iTRAQ4plex(K)@8		0.0	1287.71	644.86	1287.73	644.87	2	382.3	4101.7	8.5	91.5	10.7
			99	GLSATVTGGQK	iTRAQ4plex@N-term; Lys->Arg@11		0.0	1189.64	595.83	1189.65	595.83	2	2551.5	3585.6	41.6	58.4	1.4
			99	MYGVLPWNAFPGK	iTRAQ4plex@N-term; Lys->Arg@13		0.1	1650.90	826.46	1650.85	826.43	2	16.2	206.2	7.3	92.7	12.7
			99	MYGVLPWNAFPGK	iTRAQ4plex@N-term; Lys->Arg@13		0.0	1650.88	826.45	1650.85	826.43	2	6.1	232.9	2.6	97.4	38.0
			99	MYGVLPWNAFPGK	iTRAQ4plex@N-term; Lys->Arg@13		0.0	1650.86	551.29	1650.85	551.29	3	1.7	52.2	3.2	96.8	30.7
			99	MYGVLPWNAFPGK	iTRAQ4plex@N-term; Lys->Gln@13		0.0	1622.82	812.42	1622.80	812.41	2	59.4	276.4	17.7	82.3	4.7
			99	MYGVLPWNAFPGK	iTRAQ4plex@N-term; Lys->Gln@13		0.0	1622.85	541.96	1622.80	541.94	3	7.1	57.3	11.0	89.0	8.1
			99	MYGVLPWNAFPGK	iTRAQ4plex@N-term; Oxidation(M)@1; iTRAQ4plex(K)@13		0.0	1782.89	595.31	1782.94	595.32	3	5.2	58.4	8.2	91.8	11.3
			99	MYGVLPWNAFPGK	iTRAQ4plex@N-term; Oxidation(M)@1; Lys->Gln@13		0.1	1638.87	820.44	1638.80	820.41	2	44.8	126.2	26.2	73.8	2.8
			99	MYGVLPWNAFPGK	iTRAQ4plex@N-term; Oxidation(P)@6; iTRAQ4plex(K)@13		0.0	1782.89	595.31	1782.94	595.32	3	33.4	186.2	15.2	84.8	5.6
			99	TSASIGSLCADAR	iTRAQ4plex@N-term; Pyridylethyl(C)@9		0.0	1499.72	500.91	1499.75	500.92	3	473.9	1850.4	20.4	79.6	3.9
			98	QIFGDYK	iTRAQ4plex@N-term; iTRAQ4plex(K)@7		0.0	1157.62	579.82	1157.63	579.82	2	1318.4	11877.7	10.0	90.0	9.0
			96	MYGVLPWNAFPGK	iTRAQ4plex@N-term; Pro->pyro-Glu(P)@11; Gly->Ala@12; iTRAQ4plex(K)@13		0.0	1794.93	599.32	1794.94	599.32	3	1.7	10.9	13.5	86.5	6.4
			84	LIETYFSK	iTRAQ4plex@N-term; Lys->Gln@8		0.0	1143.63	572.82	1143.59	572.80	2	120.6	804.6	13.0	87.0	6.7
																Average	9.2
																Stdev	9.5
IPI:IPI00276515.4	MAG	Myelin-associated glycoprotein	99	GQAQAPPR	iTRAQ4plex@N-term		0.0	967.55	484.78	967.53	484.77	2	19.2	25.3	43.1	56.9	1.3
			99	TQVVHESFQGR	iTRAQ4plex@N-term		0.0	1430.76	477.93	1430.74	477.92	3	531.0	590.5	47.3	52.7	1.1
			99	NLYGTQSLELPFQGAHR	iTRAQ4plex@N-term		0.0	2074.04	692.36	2074.07	692.36	3	334.3	716.1	31.8	68.2	2.1
			99	NVTESSSFSGGDNPHVLYSPEFR	iTRAQ4plex@N-term		0.0	2669.22	890.75	2669.25	890.76	3	286.7	365.9	43.9	56.1	1.3
			99	NLYGTQSLELPFQGAHR	iTRAQ4plex@N-term		0.0	2074.04	692.36	2074.07	692.36	3	268.6	361.1	42.7	57.3	1.3
			99	NVTESSSFSGGDNPHVLYSPEFR	iTRAQ4plex@N-term		-0.1	2669.20	890.74	2669.25	890.76	3	172.8	228.5	43.1	56.9	1.3
			99	SGLLLTSILTIR	iTRAQ4plex@N-term		0.0	1429.88	715.95	1429.90	715.96	2	51.0	55.6	47.9	52.1	1.1
			99	SGLLLTSILTIR	iTRAQ4plex@N-term		0.0	1429.87	715.94	1429.90	715.96	2	50.2	78.4	39.0	61.0	1.6
			99	SGLLLTSILTIR	iTRAQ4plex@N-term		0.0	1429.88	715.95	1429.90	715.96	2	37.0	38.7	48.9	51.1	1.0
			99	GQAQAPPR	iTRAQ4plex@N-term		1.1	968.59	485.30	967.53	484.77	2	24.1	42.3	36.2	63.8	1.8
			95	EFVYSER	iTRAQ4plex@N-term		0.0	1072.57	537.29	1072.53	537.27	2	769.7	1119.5	40.7	59.3	1.5
			41	DGMVLR	iTRAQ4plex@N-term		0.0	833.48	417.75	833.46	417.73	2					
			12	EAVAK	iTRAQ4plex@N-term; iTRAQ4plex(K)@5		0.0	804.52	403.27	804.49	403.25	2					
																Average	1.4
																Stdev	0.3
IPI:IPI00223378.1	MBP	Myelin basic protein	99	DTGILDSIGR	iTRAQ4plex@N-term		0.0	1189.63	595.82	1189.64	595.83	2	1409.9	1727.6	44.9	55.1	1.2
			99	YLATASTMDHAR	iTRAQ4plex@N-term		1.0	1480.70	494.57	1479.73	494.25	3	1255.3	1551.0	44.7	55.3	1.2
			99	DTGILDSIGRFFSGDR	iTRAQ4plex@N-term; Deamidated(R)@10	missed R-F@10	0.0	1899.99	634.34	1899.94	634.32	3	5.2	7.4	41.4	58.6	1.4
			99	TTHYGSLPQK	iTRAQ4plex@N-term; Lys->Gln@10		0.1	1274.70	425.91	1274.64	425.89	3	253.3	330.1	43.4	56.6	1.3
			99	GAYDAQGTLSK	iTRAQ4plex@N-term; Lys->Gln@11		0.0	1253.62	627.82	1253.60	627.81	2	574.5	403.2	58.8	41.2	0.7
			99	TQDENPVVHFFK	iTRAQ4plex@N-term; Lys->Gln@12		0.0	1603.77	535.60	1603.78	535.60	3	78.3	111.9	41.2	58.8	1.4
			57	DTGILDSIGRFFSGDR	iTRAQ4plex@N-term	missed R-F@10	1.2	1900.17	634.40	1898.96	633.99	3	1.7	0.0	100.0	0.0	0.0
			27	NIVTPR	iTRAQ4plex@N-term		0.0	842.52	422.27	842.51	422.26	2					
			17	TPPPSQGK	iTRAQ4plex@N-term; Lys->Gln@8		0.1	954.55	478.28	954.49	478.25	2					
																Average	1.0
																Stdev	0.5
IPI:IPI00130280.1	ATP5A1	ATP synthase, H+ transporting, mitochondrial F1 complex, alpha subunit 1	99	AVDSLVPIGR	iTRAQ4plex@N-term		0.0	1169.66	585.84	1169.69	585.85	2	2620.8	3590.9	42.2	57.8	1.4
			99	VLSIGDGIAR	iTRAQ4plex@N-term		0.0	1143.64	572.83	1143.67	572.84	2	1716.3	1604.8	51.7	48.3	0.9
			99	TGTAEMSSILEER	iTRAQ4plex@N-term		0.0	1566.73	784.37	1566.77	784.39	2	550.1	519.6	51.4	48.6	0.9
			99	VGSAAQTR	iTRAQ4plex@N-term		0.0	932.56	467.29	932.52	467.27	2	474.8	426.0	52.7	47.3	0.9
			99	EAYPGDVFYLHSR	iTRAQ4plex@N-term		0.0	1696.85	566.62	1696.83	566.62	3	183.9	225.0	45.0	55.0	1.2
			99	TGAIVDVPVGEELLGR	iTRAQ4plex@N-term		0.0	1767.96	884.99	1767.99	885.00	2	128.6	135.8	48.6	51.4	1.1
			99	TGAIVDVPVGEELLGR	iTRAQ4plex@N-term		0.0	1767.98	590.33	1767.99	590.34	3	13.9	21.2	39.5	60.5	1.5
			99	TGAIVDVPVGEELLGR	iTRAQ4plex@N-term		0.0	1767.98	590.33	1767.99	590.34	3	8.9	6.2	59.1	40.9	0.7
			99	QGQYSPMAIEEQVAVIYAGVF	iTRAQ4plex@N-term		-0.9	2451.34	818.12	2452.25	818.43	3	7.0	9.5	42.3	57.7	1.4
			99	GMSLNLEPDNVGVVVFGNDK	iTRAQ4plex@N-term; iTRAQ4plex(K)@20		0.0	2391.27	798.10	2391.23	798.09	3	21.1	21.9	49.1	50.9	1.0
			99	ISEQSDAK	iTRAQ4plex@N-term; iTRAQ4plex(K)@8		0.1	1164.69	583.35	1164.62	583.32	2	1144.4	1180.0	49.2	50.8	1.0
			99	VVDALGNAIDGK	iTRAQ4plex@N-term; Lys->Gln@12		0.0	1314.68	658.35	1314.69	658.35	2	983.6	926.2	51.5	48.5	0.9
			99	VVDALGNAIDGK	iTRAQ4plex@N-term; Lys->Gln@12		0.0	1314.71	658.36	1314.69	658.35	2	537.3	467.2	53.5	46.5	0.9
			98	VVDALGNAIDGK	iTRAQ4plex@N-term; iTRAQ4plex(K)@12		0.0	1458.82	487.28	1458.83	487.28	3	103.7	116.3	47.1	52.9	1.1
			98	EPMQTGIK	iTRAQ4plex@N-term; iTRAQ4plex(K)@8		0.0	1190.64	596.33	1190.66	596.34	2	3023.9	3626.2	45.5	54.5	1.2
			93	LELAQYR	iTRAQ4plex@N-term		0.0	1035.56	518.79	1035.58	518.80	2	3619.3	3408.0	51.5	48.5	0.9
			90	TGTAEMSSILEER	iTRAQ4plex@N-term; Dimethyl(R)@13		0.0	1594.79	532.60	1594.80	532.61	3	12.4	11.5	51.9	48.1	0.9
			86	QAVAYR	iTRAQ4plex@N-term		0.0	850.50	426.26	850.48	426.25	2	248.0	273.4	47.6	52.4	1.1
			69	ELIIGDR	iTRAQ4plex@N-term		0.0	958.54	480.28	958.56	480.29	2	4651.7	4899.1	48.7	51.3	1.1
			35	VVDALGNAIDGK	iTRAQ4plex@N-term; Deamidated(N)@7; Lys->Arg@12		0.0	1343.68	672.85	1343.72	672.87	2					
																Average	1.1
																Stdev	0.2
IPI:IPI00468481.2	ATP5B	ATP synthase, H+ transporting mitochondrial F1 complex, beta subunit	99	AIAELGIYPAVDPLDSTSR	iTRAQ4plex@N-term		0.0	2131.13	711.38	2131.13	711.38	3	7.0	8.4	45.4	54.6	1.2
			99	TIAMDGTEGLVR	iTRAQ4plex@N-term		-0.1	1405.69	703.85	1405.74	703.88	2	6722.4	5467.5	55.1	44.9	0.8
			99	TIAMDGTEGLVR	iTRAQ4plex@N-term		0.0	1405.72	703.87	1405.74	703.88	2	2330.0	1887.3	55.2	44.8	0.8
			99	IMDPNIVGNEHYDVAR	iTRAQ4plex@N-term		-0.1	1985.92	662.98	1985.98	663.00	3	909.8	890.4	50.5	49.5	1.0
			99	IMNVIGEPIDER	iTRAQ4plex@N-term		0.0	1528.82	765.42	1528.80	765.41	2	758.1	685.2	52.5	47.5	0.9
			99	FTQAGSEVSALLGR	iTRAQ4plex@N-term		0.0	1578.87	790.44	1578.85	790.43	2	757.8	593.6	56.1	43.9	0.8
			99	FTQAGSEVSALLGR	iTRAQ4plex@N-term		0.0	1578.84	790.43	1578.85	790.43	2	630.2	481.1	56.7	43.3	0.8
			99	IMNVIGEPIDER	iTRAQ4plex@N-term		0.0	1528.81	765.41	1528.80	765.41	2	530.1	413.3	56.2	43.8	0.8
			99	IMNVIGEPIDER	iTRAQ4plex@N-term		0.0	1528.83	765.42	1528.80	765.41	2	348.3	283.7	55.1	44.9	0.8
			99	VALTGLTVAEYFR	iTRAQ4plex@N-term		0.0	1582.90	792.46	1582.88	792.45	2	258.1	245.8	51.2	48.8	1.0
			99	IMNVIGEPIDER	iTRAQ4plex@N-term		0.0	1528.80	765.41	1528.80	765.41	2	251.3	234.0	51.8	48.2	0.9
			99	LVLEVAQHLGESTVR	iTRAQ4plex@N-term		0.0	1794.01	599.01	1794.01	599.01	3	55.1	45.7	54.7	45.3	0.8
			99	IPSAVGYQPTLATDMGTMQER	iTRAQ4plex@N-term		0.0	2409.21	804.08	2409.18	804.07	3	35.0	44.1	44.2	55.8	1.3
			99	IPSAVGYQPTLATDMGTMQER	iTRAQ4plex@N-term		0.0	2409.17	804.06	2409.18	804.07	3	29.7	38.0	43.9	56.1	1.3
			99	IPSAVGYQPTLATDMGTMQER	iTRAQ4plex@N-term		0.0	2409.21	804.08	2409.18	804.07	3	12.3	14.7	45.4	54.6	1.2
			99	VALTGLTVAEYFR	iTRAQ4plex@N-term		0.0	1582.91	528.65	1582.88	528.64	3	5.2	7.4	41.4	58.6	1.4
			99	FLSQPFQVAEVFTGH	iTRAQ4plex@N-term	cleaved H-M@C-term	0.0	1849.97	617.66	1849.95	617.66	3	1.7	13.0	11.5	88.5	7.7
			99	TVLIMELINNVAK	iTRAQ4plex@N-term; iTRAQ4plex(K)@13		0.1	1745.10	873.56	1745.04	873.53	2	10.6	10.5	50.3	49.7	1.0
			99	TVLIMELINNVAK	iTRAQ4plex@N-term; iTRAQ4plex(K)@13		0.0	1745.00	582.67	1745.04	582.69	3	1.7	6.5	20.7	79.3	3.8
			99	VVDLLAPYAK	iTRAQ4plex@N-term; Lys->Gln@10		0.0	1231.71	616.86	1231.69	616.85	2	155.9	139.3	52.8	47.2	0.9
			99	EGNDLYHEMIESGVINLK	iTRAQ4plex@N-term; Lys->Gln@18		0.1	2204.12	735.71	2204.05	735.69	3	14.3	10.3	58.0	42.0	0.7
			99	FLSQPFQVAEVFTGHMGK	iTRAQ4plex@N-term; Lys->Gln@18		0.1	2166.15	723.06	2166.07	723.03	3	6.8	14.9	31.2	68.8	2.2
			99	EGNDLYHEMIESGVINLK	iTRAQ4plex@N-term; Lys->Gln@18		0.0	2204.07	735.70	2204.05	735.69	3	6.6	18.2	26.8	73.2	2.7
			99	VLDSGAPIKIPVGPETLGR	iTRAQ4plex@N-term; Lys->Gln@9	missed K-I@9	0.0	2062.15	688.39	2062.15	688.39	3	105.2	74.0	58.7	41.3	0.7
			99	TVLIMELINNVAK	iTRAQ4plex@N-term; No iTRAQ4plex(K)@13		0.0	1600.94	801.48	1600.93	801.47	2	31.4	41.2	43.3	56.7	1.3
			99	FLSQPFQVAEVFTGHMGK	iTRAQ4plex@N-term; No iTRAQ4plex(K)@18		0.0	2166.14	723.06	2166.11	723.04	3	9.0	4.0	69.3	30.7	0.4
			99	TIAMDGTEGLVR	iTRAQ4plex@N-term; Oxidation(M)@4		0.0	1421.68	711.85	1421.73	711.87	2	568.8	409.7	58.1	41.9	0.7
			99	LVLEVAQHLGESTVR	iTRAQ4plex@N-term; Ser->Asp@12		0.0	1822.01	608.35	1822.01	608.34	3	10.0	27.7	26.4	73.6	2.8
			98	IMNVIGEPIDER	iTRAQ4plex@N-term; Dimethyl(R)@12		0.0	1556.85	779.43	1556.84	779.42	2	84.3	297.0	22.1	77.9	3.5
			98	VVDLLAPYAK	iTRAQ4plex@N-term; Lys->Gln@10		0.0	1231.72	616.87	1231.69	616.85	2	144.7	95.3	60.3	39.7	0.7

			96	IMNVIGEPIDER	iTRAQ4plex@N-term; Oxidation(M)@2		0.0	1544.81	773.41	1544.80	773.41	2	145.8	115.9	55.7	44.3	0.8
			91	FTQAGSEVSALLGR	iTRAQ4plex@N-term; Dimethyl(R)@14		-0.1	1606.82	804.42	1606.88	804.45	2	30.9	106.1	22.6	77.4	3.4
			85	TVLIMELINNVAK	iTRAQ4plex@N-term; Asn->Leu@10; Lys->Gln@13		0.1	1600.04	801.03	1599.94	800.98	2	28.3	26.1	52.1	47.9	0.9
			79	IMNVIGEPIDER	iTRAQ4plex@N-term; Oxidation(M)@2		0.0	1544.79	773.40	1544.80	773.41	2	110.2	90.0	55.0	45.0	0.8
			74	LAEEHGS	iTRAQ4plex@N-term		1.0	886.48	444.24	885.43	443.72	2	128.7	132.7	49.2	50.8	1.0
			61	TVLIMELINNVAK	iTRAQ4plex@N-term; Lys->Arg@13		0.0	1628.98	815.50	1628.94	815.48	2	8.6	16.0	34.9	65.1	1.9
			58	IPVGPETLGR	iTRAQ4plex@N-term		0.0	1181.68	591.85	1181.69	591.85	2	871.7	1533.4	36.2	63.8	1.8
			55	LAEEHGS	iTRAQ4plex@N-term		0.0	885.47	443.74	885.43	443.72	2	19.2	26.4	42.0	58.0	1.4
			32	ILQDYK	iTRAQ4plex@N-term; iTRAQ4plex(K)@6		0.0	1066.64	534.33	1066.63	534.32	2					
			21	EGNDLYHEMIESGVINLK	iTRAQ4plex@N-term; Deamidated(N)@16; iTRAQ4plex(K)@18		0.0	2349.17	588.30	2349.18	588.30	4					
			19	GLFGGAGVGK	iTRAQ4plex@N-term; iTRAQ4plex(K)@10	cleaved I-G@N-term	0.0	1149.68	575.85	1149.67	575.84	2					
																Average	1.5
																Stdev	1.3
IPI:IPI00125460.1	ATP5J	ATP synthase, H+ transporting, mitochondrial F0 complex, subunit F	99	FEVIDKPQS	iTRAQ4plex@N-term; iTRAQ4plex(K)@6		0.0	1349.72	675.87	1349.74	675.88	2	3393.9	5066.3	40.1	59.9	1.5
			99	QASGGPVDIGPEYQQDLDR	iTRAQ4plex@N-term		-0.1	2187.99	730.34	2188.05	730.36	3	164.4	157.2	51.1	48.9	1.0
			99	QASGGPVDIGPEYQQDLDR	iTRAQ4plex@N-term		-0.1	2187.99	730.34	2188.05	730.36	3	153.1	168.3	47.6	52.4	1.1
			99	GEMDTFPTFK	iTRAQ4plex@N-term; Lys->Gln@10		0.0	1315.60	658.81	1315.59	658.80	2	768.2	508.3	60.2	39.8	0.7
			99	GEMDTFPTFK	iTRAQ4plex@N-term; Lys->Gln@10		0.0	1315.63	658.82	1315.59	658.80	2	416.1	330.8	55.7	44.3	0.8
			99	FEVIDKPQS	iTRAQ4plex@N-term; Lys->Gln@6		0.0	1205.63	603.82	1205.61	603.81	2	2149.4	2771.0	43.7	56.3	1.3
			97	GEMDTFPTFK	iTRAQ4plex@N-term; Lys->Gln@10		0.0	1315.60	658.81	1315.59	658.80	2	223.2	149.7	59.9	40.1	0.7
			97	FEVIDKPQS	iTRAQ4plex@N-term; Lys->Gln@6		0.0	1205.63	603.82	1205.61	603.81	2	1251.5	1603.2	43.8	56.2	1.3
			91	ELDPVQK	iTRAQ4plex@N-term; iTRAQ4plex(K)@7		0.0	1115.66	558.84	1115.64	558.83	2	889.5	1495.2	37.3	62.7	1.7
			86	GEMDTFPTFK	iTRAQ4plex@N-term; Lys->Gln@10		0.0	1315.60	658.81	1315.59	658.80	2	448.0	308.2	59.2	40.8	0.7
			54	GEMDTFPTFK	iTRAQ4plex@N-term; Lys->Arg@10		0.1	1343.68	672.85	1343.63	672.82	2	192.6	287.2	40.1	59.9	1.5
			40	GEMDTFPTFK	iTRAQ4plex@N-term; Lys->Arg@10		0.1	1343.68	672.85	1343.63	672.82	2					
																Average	1.1
																Stdev	0.4
IPI:IPI00118986.1	ATP5O	ATP synthase, H+ transporting, mitochondrial F1 complex, O subunit	99	FSPLTANLMNLLAENGR	iTRAQ4plex@N-term		0.0	2004.06	669.03	2004.06	669.03	3	10.5	13.7	43.3	56.7	1.3
			99	TDPSIMGGMIVR	iTRAQ4plex@N-term		0.0	1419.72	710.87	1419.73	710.87	2	1100.5	881.4	55.5	44.5	0.8
			99	TDPSIMGGMIVR	iTRAQ4plex@N-term		0.0	1419.72	710.87	1419.73	710.87	2	781.8	636.7	55.1	44.9	0.8
			99	LGNTQGIISAFSTIMSVHR	iTRAQ4plex@N-term		1.0	2176.16	726.39	2175.16	726.06	3	23.0	21.9	51.2	48.8	1.0
			99	SFLSPNQILK	iTRAQ4plex@N-term; iTRAQ4plex(K)@10		0.0	1433.81	478.94	1433.85	478.96	3	48.9	64.4	43.2	56.8	1.3
			99	YATALYSAASK	iTRAQ4plex@N-term; Lys->Gln@11		0.0	1288.68	645.35	1288.64	645.33	2	821.1	465.2	63.8	36.2	0.6
			99	VSLAVLNPYIK	iTRAQ4plex@N-term; Lys->Gln@11		0.1	1359.86	680.94	1359.79	680.90	2	338.0	212.7	61.4	38.6	0.6
			99	YATALYSAASK	iTRAQ4plex@N-term; Lys->Gln@11		0.0	1288.66	645.34	1288.64	645.33	2	290.1	122.3	70.3	29.7	0.4
			99	VSLAVLNPYIK	iTRAQ4plex@N-term; Lys->Gln@11		0.0	1359.82	680.92	1359.79	680.90	2	251.4	180.2	58.2	41.8	0.7
			99	GEVPCTVTTASPLDDAVLSELK	iTRAQ4plex@N-term; Pyridylethyl(C)@5; Lys->Gln@22		0.1	2493.33	832.12	2493.24	832.09	3	26.0	38.1	40.6	59.4	1.5
			99	GEVPCTVTTASPLDDAVLSELK	iTRAQ4plex@N-term; Pyridylethyl(C)@5; Lys->Gln@22		0.0	2493.20	832.07	2493.24	832.09	3	15.7	21.1	42.6	57.4	1.3
			97	VSLAVLNPYIK	iTRAQ4plex@N-term; iTRAQ4plex(K)@11		0.0	1503.91	502.31	1503.93	502.32	3	87.0	72.6	54.5	45.5	0.8
			57	YVDMSAK	iTRAQ4plex@N-term; Lys->Gln@7		0.0	956.48	479.25	956.44	479.23	2	1389.5	884.9	61.1	38.9	0.6
																Average	0.9
																Stdev	0.3
IPI:IPI00623553.1	ATP5H	ATP synthase, H+ transporting, mitochondrial F0 complex, subunit d	99	LASLSEKPPAIDWAYYR	iTRAQ4plex@N-term; Lys->Gln@7		0.0	2123.12	708.71	2123.08	708.70	3	158.6	117.2	57.5	42.5	0.7
			99	YTALVDQEEKEDVK	iTRAQ4plex@N-term; iTRAQ4plex(K)@10; Lys->Gln@14		0.0	1954.01	652.34	1953.98	652.33	3	155.0	216.0	41.8	58.2	1.4
			99	LASLSEKPPAIDWAYYR	iTRAQ4plex@N-term; iTRAQ4plex(K)@7		-0.1	2267.16	756.73	2267.22	756.75	3	1072.0	910.1	54.1	45.9	0.8
			99	LASLSEKPPAIDWAYYR	iTRAQ4plex@N-term; iTRAQ4plex(K)@7		0.0	2267.24	756.75	2267.22	756.75	3	306.8	267.1	53.5	46.5	0.9
			99	LASLSEKPPAIDWAYYR	iTRAQ4plex@N-term; Lys->Gln@7		0.0	2123.11	708.71	2123.08	708.70	3	200.1	131.9	60.3	39.7	0.7
			99	LASLSEKPPAIDWAYYR	iTRAQ4plex@N-term; Lys->Gln@7		0.0	2123.11	708.71	2123.08	708.70	3	88.7	75.7	54.0	46.0	0.9
			97	TIDWVSFVEVMPQNQK	iTRAQ4plex@N-term; Lys->Arg@16		0.0	2092.08	698.37	2092.05	698.36	3	5.1	9.6	34.9	65.1	1.9
			83	YPYWPHQPIENL	iTRAQ4plex@N-term		0.0	1699.84	850.93	1699.85	850.93	2	118.3	216.3	35.4	64.6	1.8
			34	IQEYEK	iTRAQ4plex@N-term; iTRAQ4plex(K)@6		0.0	1096.61	549.31	1096.60	549.31	2					
			10	TIDWVSFVEVMPQNQK	iTRAQ4plex@N-term; Lys->Arg@16		0.0	2092.07	698.37	2092.05	698.36	3					
																Average	1.1
																Stdev	0.5
IPI:IPI00111770.7	ATP5K	ATP synthase, H+ transporting, mitochondrial F1F0 complex, subunit e	99	ELAEAQDDSILK	iTRAQ4plex@N-term; iTRAQ4plex(K)@12		0.0	1618.87	810.44	1618.87	810.44	2	409.3	267.3	60.5	39.5	0.7
			99	ELAEAQDDSILK	iTRAQ4plex@N-term; iTRAQ4plex(K)@12		0.0	1618.84	540.62	1618.87	540.63	3	227.9	272.9	45.5	54.5	1.2
			99	YSALIIGMAYGAK	iTRAQ4plex@N-term; iTRAQ4plex(K)@13		0.0	1644.91	549.31	1644.92	549.31	3	69.0	63.6	52.0	48.0	0.9
			99	ELAEAQDDSILK	iTRAQ4plex@N-term; Lys->Arg@12		0.0	1502.79	752.40	1502.77	752.39	2	209.1	336.4	38.3	61.7	1.6
			99	ELAEAQDDSILK	iTRAQ4plex@N-term; Lys->Gln@12		0.0	1474.73	738.37	1474.73	738.37	2	647.3	412.3	61.1	38.9	0.6
			26	IAAEEK	iTRAQ4plex@N-term; iTRAQ4plex(K)@6		0.0	947.58	474.80	947.55	474.78	2					
																Average	1.0
																Stdev	0.4
IPI:IPI00230241.6	ATP5E	ATP synthase, H+ transporting, mitochondrial F1 complex, epsilon subunit	99	QAGLSYIR	iTRAQ4plex@N-term		0.0	1050.57	526.29	1050.59	526.30	2	4182.9	3209.6	56.6	43.4	0.8
			99	TSGSSIK	iTRAQ4plex@N-term; iTRAQ4plex(K)@7		0.1	966.61	484.31	966.56	484.29	2	81.5	71.7	53.2	46.8	0.9
			64	TSGSSIK	iTRAQ4plex@N-term; iTRAQ4plex(K)@7		0.0	966.60	484.31	966.56	484.29	2	48.9	63.2	43.6	56.4	1.3
																Average	1.0
																Stdev	0.3
IPI:IPI00856384.1	ATP5J2	ATP synthase, H+ transporting, mitochondrial F0 complex, subunit F2	99	DFTPSGIAGAFR	iTRAQ4plex@N-term		0.0	1381.70	691.86	1381.71	691.86	2	2387.9	2255.3	51.4	48.6	0.9
			99	DFTPSGIAGAFR	iTRAQ4plex@N-term		0.0	1381.69	691.85	1381.71	691.86	2	2639.6	2328.0	53.1	46.9	0.9
			99	LGELPSWIMMR	iTRAQ4plex@N-term		0.0	1475.77	738.89	1475.78	738.89	2	575.2	432.3	57.1	42.9	0.8
			99	LGELPSWIMMR	iTRAQ4plex@N-term		0.0	1475.77	738.89	1475.78	738.89	2	562.7	374.4	60.0	40.0	0.7
			99	LGELPSWIMMR	iTRAQ4plex@N-term		0.1	1475.84	738.93	1475.78	738.89	2	142.9	95.1	60.0	40.0	0.7
			86	LGELPSWIMMR	iTRAQ4plex@N-term		0.0	1475.82	738.92	1475.78	738.89	2	91.9	89.7	50.6	49.4	1.0
																Average	0.8
																Stdev	0.1
IPI:IPI00119115.2	ATP6V1E1	ATPase, H+ transporting, lysosomal V1 subunit E1	99	ARDDLITDLLNEAK	iTRAQ4plex@N-term; Lys->Gln@14	missed R-D@2	0.0	1729.90	577.64	1729.90	577.64	3	5.2	8.5	37.9	62.1	1.6
			99	LDLIAQQMMPEVR	iTRAQ4plex@N-term		0.0	1686.92	563.32	1686.89	563.30	3	10.0	25.7	28.1	71.9	2.6
			99	ARDDLITDLLNEAK	iTRAQ4plex@N-term; Lys->Arg@14	missed R-D@2	0.0	1757.97	587.00	1757.94	586.99	3	1.7	4.4	28.1	71.9	2.6
			90	QIEQQK	iTRAQ4plex@N-term; iTRAQ4plex(K)@6		0.1	1060.67	531.34	1060.61	531.31	2	15.9	66.6	19.2	80.8	4.2
			56	LDLIAQQMMPEVR	iTRAQ4plex@N-term; Dimethyl(R)@13		0.0	1714.93	572.65	1714.92	572.65	3	1.7	15.2	10.0	90.0	9.0
			39	AEEIDAK	iTRAQ4plex@N-term; Lys->Gln@7		0.1	918.54	460.28	918.44	460.23	2					
																Average	4.0
																Stdev	2.9
IPI:IPI00119113.3	ATP6V1B2	ATPase, H+ transporting, lysosomal V1 subunit B2	99	EQALAVSR	iTRAQ4plex@N-term		0.0	1016.59	509.30	1016.57	509.29	2	1493.9	3132.9	32.3	67.7	2.1
			99	QIYPPINVLPSLSR	iTRAQ4plex@N-term		0.0	1740.00	581.01	1740.01	581.01	3	11.9	24.5	32.7	67.3	2.1
			99	QIYPPINVLPSLSR	iTRAQ4plex@N-term		0.0	1740.04	581.02	1740.01	581.01	3	10.3	18.1	36.3	63.7	1.8
			99	GFPGYMYTDLATIYER	iTRAQ4plex@N-term		0.0	2039.96	680.99	2039.98	681.00	3	10.2	22.4	31.2	68.8	2.2
			99	GIVNGAAPELPVPTGGPMAGAR	iTRAQ4plex@N-term; Deamidated(N)@4		-0.1	2176.09	726.37	2176.14	726.39	3	36.3	59.1	38.0	62.0	1.6
			99	SGQVLEVSGSK	iTRAQ4plex@N-term; Lys->Gln@11		0.0	1233.67	617.84	1233.63	617.82	2	85.8	154.9	35.7	64.3	1.8
			99	TVSGVNGPLVILDHVK	iTRAQ4plex@N-term; Lys->Gln@16		0.0	1791.00	598.01	1791.00	598.01	3	50.3	76.3	39.7	60.3	1.5
																Average	1.9
																Stdev	0.3
IPI:IPI00828950.1	ATP6V0A1	ATPase, H+ transporting, lysosomal V0 subunit A1	99	DLNPDVNVFQR	iTRAQ4plex@N-term		0.0	1459.72	730.87	1459.75	730.88	2	116.8	307.5	27.5	72.5	2.6
			97	NFLELTELK	iTRAQ4plex@N-term; iTRAQ4plex(K)@9		0.0	1393.77	465.60	1393.81	465.61	3	1.7	16.3	9.4	90.6	9.6
			97	NFLELTELK	iTRAQ4plex@N-term; Lys->Gln@9		0.0	1249.71	625.86	1249.67	625.84	2	31.5	139.7	18.4	81.6	4.4
			91	EMASGVNTR	iTRAQ4plex@N-term		0.1	1107.62	554.82	1107.55	554.78	2	100.8	445.6	18.5	81.5	4.4
			38	NFLELTELK	iTRAQ4plex@N-term; Lys->Arg@9		0.0	1277.74	639.88	1277.71	639.86	2					
																Average	5.3
																Stdev	3.0
IPI:IPI00123817.1	ATP6V1G2	ATPase, H+ transporting, lysosomal V1 subunit G2	99	QQAAMGSQGNLSAEVEQATR	iTRAQ4plex@N-term		0.0	2219.04	740.69	2219.07	740.70	3	59.3	178.7	24.9	75.1	3.0
			98	QVQGMQSSQQR	iTRAQ4plex@N-term		0.1	1419.80	474.27	1419.70	474.24	3	14.0	17.9	43.8	56.2	1.3
			98	QVQGMQSSQQR	iTRAQ4plex@N-term		0.1	1419.78	474.27	1419.70	474.24	3	1.7	8.7	16.3	83.7	5.1
			95	EEAQMEVEQYR	iTRAQ4plex@N-term		1.0	1555.71	519.58	1554.71	519.24	3	6.2	31.1	16.6	83.4	5.0
																Average	3.6
																Stdev	1.8
IPI:IPI00407692.3	ATP6V1A	ATPase, H+ transporting, lysosomal V1 subunit A	99	ADYAQLLEDMQNAFR	iTRAQ4plex@N-term		0.0	1927.91	643.65	1927.92	643.65	3	1.7	5.4	23.8	76.2	3.2
			99	TVGMLSNMISFYDMAR	iTRAQ4plex@N-term		0.0	1978.93	660.65	1978.94	660.66	3	1.7	6.5	20.7	79.3	3.8
			99	TVGMLSNMISFYDMAR	iTRAQ4plex@N-term		0.0	1978.97	660.66	1978.94	660.66	3	1.7	15.2	10.0	90.0	9.0
			99	EILQEEEDLAEIVQLVGK	iTRAQ4plex@N-term; iTRAQ4plex(K)@18		0.0	2342.33	781.78	2342.28	781.77	3	1.7	19.6	8.0	92.0	11.5
			75	ALDEYYDK	iTRAQ4plex@N-term; iTRAQ4plex(K)@8		-1.0	1302.68	652.35	1303.65	652.83	2	95.0	304.2	23.8	76.2	3.2
			61	ITWSIIR	iTRAQ4plex@N-term		0.0	1031.62	516.82	1031.62	516.82	2	251.3	734.0	25.5	74.5	2.9
																Average	5.6
																Stdev	3.7
IPI:IPI00118787.1	ATP6V1D	ATPase, H+ transporting, lysosomal V1 subunit D	99	TLAYIITELDER	iTRAQ4plex@N-term		0.0	1579.86	790.94	1579.86	790.94	2	44.7	77.6	36.6	63.4	1.7
			87	GGEQLAK	iTRAQ4plex@N-term; iTRAQ4plex(K)@7		0.0	989.62	495.82	989.57	495.79	2	36.8	93.9	28.2	71.8	2.6
																Average	2.1
																Stdev	0.6
IPI:IPI00119618.1	CANX	Calnexin	99	APVPTGEVYFADSFDR	iTRAQ4plex@N-term		0.0	1913.89	638.97	1913.93	638.98	3	24.6	27.3	47.4	52.6	1.1
			99	APVPTGEVYFADSFDR	iTRAQ4plex@N-term		0.0	1913.89	638.97	1913.93	638.98	3	11.8	27.6	29.9	70.1	2.3
			98	APVPTGEVYFADSFDR	iTRAQ4plex@N-term		0.0	1913.92	638.98	1913.93	638.98	3	6.7	17.1	28.1	71.9	2.6
			84	EIEDPEDR	iTRAQ4plex@N-term		0.0	1145.55	573.78	1145.53	573.77	2	179.7	441.4	28.9	71.1	2.5
			70	TGVYEEK	iTRAQ4plex@N-term; Lys->Gln@7		0.1	968.52	485.27	968.46	485.24	2	12.1	20.2	37.4	62.6	1.7
																Average	2.0
																Stdev	0.6
IPI:IPI00475246.1	IGHG1	Immunoglobulin heavy constant gamma 1	99	APQVYTIPPPK	iTRAQ4plex@N-term; Lys->Gln@11		0.0	1353.75	677.88	1353.74	677.88	2	1412.6	1401.9	50.2	49.8	1.0
			99	EEQFNSTFR	iTRAQ4plex@N-term		0.0	1300.63	651.32	1300.62	651.32	2	5779.5	3283.8	63.8	36.2	0.6
			99	EEQFNSTFR	iTRAQ4plex@N-term		0.0	1300.64	651.33	1300.62	651.32	2	1790.6	1125.4	61.4	38.6	0.6
			99	DDPEVQFSWFVDDVEVHTAQTQPR	iTRAQ4plex@N-term		0.0	2988.37	748.10	2988.40	748.11	4	34.7	53.0	39.6	60.4	1.5
			99	DDPEVQFSWFVDDVEVHTAQTQPR	iTRAQ4plex@N-term		1.2	2989.58	748.40	2988.40	748.11	4	17.3	27.6	38.5	61.5	1.6
			99	DDPEVQFSWFVDDVEVHTAQTQPR	iTRAQ4plex@N-term		0.0	2988.42	748.11	2988.40	748.11	4	16.0	11.3	58.6	41.4	0.7
			99	DDPEVQFSWFVDDVEVHTAQTQPR	iTRAQ4plex@N-term		0.0	2988.38	748.10	2988.40	748.11	4	5.1	11.8	30.1	69.9	2.3
			99	DDPEVQFSWFVDDVEVHTAQTQPR	iTRAQ4plex@N-term		0.0	2988.41	748.11	2988.40	748.11	4	1.7	3.3	34.3	65.7	1.9
			99	SVSELPIMHQDWLNGK	iTRAQ4plex@N-term; Asn->Pro@14; iTRAQ4plex(K)@16		0.0	2124.10	709.04	2124.13	709.05	3	335.2	289.7	53.6	46.4	0.9
			99	SVSELPIMHQDWLNGK	iTRAQ4plex@N-term; Asn->Pro@14; Lys->Gln@16		0.0	1979.98	661.00	1979.99	661.00	3	196.0	144.3	57.6	42.4	0.7
			99	SVSELPIMHQDWLNGKEFK	iTRAQ4plex@N-term; Deamidated(N)@14; iTRAQ4plex(K)@16; Dehydrated(E)@17; Lys->Arg@19		0.0	2556.27	640.07	2556.30	640.08	4	13.7	25.5	34.9	65.1	1.9
			99	SVSELPIMHQDWLNGK	iTRAQ4plex@N-term; Deamidated(N)@14; Lys->Gln@16		0.0	1998.00	667.01	1997.96	667.00	3	131.9	145.5	47.5	52.5	1.1
			99	SVSELPIMHQDWLNGK	iTRAQ4plex@N-term; Deamidated(N)@14; Lys->Gln@16		0.0	1997.97	667.00	1997.96	667.00	3	108.1	95.6	53.1	46.9	0.9
			99	TTPPSVYPLAPGSAAQTNSMVTLGCLVK	iTRAQ4plex@N-term; Deamidated(N)@18; Pyridylethyl(C)@25; iTRAQ4plex(K)@28		-0.1	3196.61	800.16	3196.68	800.18	4	10.4	14.7	41.5	58.5	1.4
			99	DDPEVQFSWFVDDVEVHTAQTQPR	iTRAQ4plex@N-term; Gln->Arg@22		0.0	3016.41	755.11	3016.44	755.12	4	1.7	5.4	23.8	76.2	3.2
			99	SVSELPIMHQDWLNGK	iTRAQ4plex@N-term; Gly->Ala@15; Lys->Pro@16		0.0	1980.00	661.01	1979.99	661.00	3	56.9	44.5	56.1	43.9	0.8
			99	DVLTITLTPK	iTRAQ4plex@N-term; iTRAQ4plex(K)@10		1.0	1388.84	695.42	1387.85	694.93	2	1481.0	1282.8	53.6	46.4	0.9
			99	DVLTITLTPK	iTRAQ4plex@N-term; iTRAQ4plex(K)@10		0.0	1387.86	694.94	1387.85	694.93	2	1257.0	1202.2	51.1	48.9	1.0
			99	DVLTITLTPK	iTRAQ4plex@N-term; iTRAQ4plex(K)@10		0.0	1387.81	694.91	1387.85	694.93	2	708.2	599.5	54.2	45.8	0.8
			99	VNSAAFPAPIEK	iTRAQ4plex@N-term; iTRAQ4plex(K)@12		0.0	1530.83	511.29	1530.86	511.30	3	1406.5	1719.0	45.0	55.0	1.2
			99	VNSAAFPAPIEK	iTRAQ4plex@N-term; iTRAQ4plex(K)@12		0.0	1530.82	511.28	1530.86	511.30	3	852.9	747.0	53.3	46.7	0.9
			99	VNSAAFPAPIEK	iTRAQ4plex@N-term; iTRAQ4plex(K)@12		0.0	1530.84	511.29	1530.86	511.30	3	603.9	548.6	52.4	47.6	0.9
			99	VNSAAFPAPIEKTISK	iTRAQ4plex@N-term; iTRAQ4plex(K)@12; Ser->Pro@15; Lys->Glu@16	missed K-T@12	0.0	1971.13	658.05	1971.09	658.04	3	240.4	180.3	57.1	42.9	0.7
			99	SVSELPIMHQDWLNGK	iTRAQ4plex@N-term; iTRAQ4plex(K)@16		0.0	2141.09	714.70	2141.12	714.71	3	6513.7	5172.7	55.7	44.3	0.8
			99	SVSELPIMHQDWLNGK	iTRAQ4plex@N-term; iTRAQ4plex(K)@16		0.0	2141.10	714.71	2141.12	714.71	3	109.8	98.8	52.6	47.4	0.9
			99	SVSELPIMHQDWLNGKEFK	iTRAQ4plex@N-term; iTRAQ4plex(K)@16; Glu->Pro@17; Lys->Arg@19		-0.1	2541.28	636.33	2541.34	636.34	4	16.0	13.5	54.2	45.8	0.8
			99	NTQPIMDTDGSYFIYSK	iTRAQ4plex@N-term; iTRAQ4plex(K)@17		-0.1	2267.05	756.69	2267.10	756.71	3	186.7	196.7	48.7	51.3	1.1
			99	NTQPIMDTDGSYFIYSK	iTRAQ4plex@N-term; iTRAQ4plex(K)@17		0.0	2267.07	756.70	2267.10	756.71	3	165.4	179.1	48.0	52.0	1.1
			99	VNSAAFPAPIEK	iTRAQ4plex@N-term; Lys->Arg@12		0.0	1414.78	708.40	1414.77	708.39	2	2313.6	3934.4	37.0	63.0	1.7
			99	VNSAAFPAPIEK	iTRAQ4plex@N-term; Lys->Arg@12		0.0	1414.73	708.37	1414.77	708.39	2	1272.7	1824.8	41.1	58.9	1.4
			99	SVSELPIMHQDWLNGK	iTRAQ4plex@N-term; Lys->Arg@16		0.0	2025.03	676.02	2025.02	676.01	3	304.7	872.7	25.9	74.1	2.9
			99	DVLTITLTPK	iTRAQ4plex@N-term; Lys->Gln@10		0.0	1243.72	622.87	1243.71	622.86	2	1007.0	736.1	57.8	42.2	0.7
			99	DVLTITLTPK	iTRAQ4plex@N-term; Lys->Gln@10		0.0	1243.74	622.88	1243.71	622.86	2	424.7	244.7	63.4	36.6	0.6
			99	APQVYTIPPPK	iTRAQ4plex@N-term; Lys->Gln@11		0.0	1353.79	677.90	1353.74	677.88	2	935.6	887.4	51.3	48.7	0.9
			99	VNSAAFPAPIEK	iTRAQ4plex@N-term; Lys->Gln@12		0.0	1386.72	694.37	1386.73	694.37	2	15116.7	8610.6	63.7	36.3	0.6
			99	SVSELPIMHQDWLNGK	iTRAQ4plex@N-term; Lys->Gln@16		-0.1	1996.90	666.64	1996.98	666.67	3	6592.4	3952.7	62.5	37.5	0.6
			99	SVSELPIMHQDWLNGK	iTRAQ4plex@N-term; Lys->Gln@16		0.0	1997.02	666.68	1996.98	666.67	3	109.7	103.1	51.5	48.5	0.9
			99	SVSELPIMHQDWLNGK	iTRAQ4plex@N-term; Lys->Gln@16		0.0	1997.00	666.67	1996.98	666.67	3	91.3	55.2	62.3	37.7	0.6
			99	NTQPIMDTDGSYFIYSK	iTRAQ4plex@N-term; Lys->Gln@17		0.0	2122.96	708.66	2122.96	708.66	3	43.0	25.5	62.7	37.3	0.6
			99	SVSELPIMHQDWLNGK	iTRAQ4plex@N-term; Oxidation(D)@11; Oxidation(W)@12; iTRAQ4plex(K)@16		0.0	2173.11	725.38	2173.11	725.38	3	310.5	266.8	53.8	46.2	0.9
			99	SVSELPIMHQDWLNGK	iTRAQ4plex@N-term; Oxidation(H)@9; Deamidated(Q)@10; Dehydrated(D)@11; Oxidation(W)@12; iTRAQ4plex(K)@16		0.0	2156.06	540.02	2156.08	540.03	4	123.9	215.2	36.5	63.5	1.7
			99	SVSELPIMHQDWLNGK	iTRAQ4plex@N-term; Oxidation(M)@8; iTRAQ4plex(K)@16		0.0	2157.14	720.06	2157.11	720.05	3	608.2	480.8	55.9	44.1	0.8
			99	SVSELPIMHQDWLNGK	iTRAQ4plex@N-term; Oxidation(M)@8; Lys->Gln@16		0.0	2012.96	671.99	2012.97	672.00	3	1029.5	719.5	58.9	41.1	0.7
			99	SVSELPIMHQDWLNGK	iTRAQ4plex@N-term; Oxidation(M)@8; Lys->Gln@16		0.0	2012.95	671.99	2012.97	672.00	3	103.5	71.9	59.0	41.0	0.7
			99	TTPPSVYPLAPGSAAQTNSMVTLGCLVK	iTRAQ4plex@N-term; Pyridylethyl(C)@25; iTRAQ4plex(K)@28		0.0	3195.67	799.92	3195.69	799.93	4	29.7	37.8	44.0	56.0	1.3
			99	TTPPSVYPLAPGSAAQTNSMVTLGCLVK	iTRAQ4plex@N-term; Pyridylethyl(C)@25; Lys->Gln@28		0.1	3051.60	763.91	3051.55	763.90	4	216.2	140.8	60.6	39.4	0.7
			99	SMVTLGCLVK	iTRAQ4plex@N-term; Pyridylethyl(C)@7; Lys->Gln@10	cleaved N-S@N-term	0.0	1298.69	433.91	1298.68	433.90	3	72.1	129.5	35.8	64.2	1.8
			98	VTCVVVDISK	iTRAQ4plex@N-term; Pyridylethyl(C)@3; Lys->Gln@10		0.0	1310.74	437.92	1310.70	437.91	3	56.6	102.7	35.5	64.5	1.8
			97	SVSELPIMHQD	iTRAQ4plex@N-term; Dehydrated(D)@11	cleaved D-W@C-term	0.0	1380.71	691.36	1380.68	691.35	2	183.2	144.6	55.9	44.1	0.8
			97	DVLTITLTPK	iTRAQ4plex@N-term; Lys->Arg@10		0.0	1271.75	636.88	1271.76	636.89	2	148.1	304.3	32.7	67.3	2.1
			97	DVLTITLTPK	iTRAQ4plex@N-term; Lys->Arg@10		0.0	1271.77	636.89	1271.76	636.89	2	122.5	302.1	28.9	71.1	2.5
			97	DVLTITLTPK	iTRAQ4plex@N-term; Lys->Arg@10		0.0	1271.77	636.89	1271.76	636.89	2	56.8	197.2	22.4	77.6	3.5
			95	VNSAAFPAPIEK	iTRAQ4plex@N-term; Cation:Na(E)@11; iTRAQ4plex(K)@12		0.0	1552.83	518.62	1552.85	518.62	3	159.9	182.4	46.7	53.3	1.1
			94	APQVYTIPPPK	iTRAQ4plex@N-term; Lys->Gln@11		0.1	1353.79	677.90	1353.74	677.88	2	368.1	393.2	48.4	51.6	1.1
			93	SVSELPIMHQDW	iTRAQ4plex@N-term; Dehydrated(D)@11	cleaved W-L@C-term	0.0	1566.76	784.39	1566.76	784.39	2	296.3	253.2	53.9	46.1	0.9
			90	TPPSVYPLAPGSAAQTNSMVTLGCLVK	iTRAQ4plex@N-term; Pyridylethyl(C)@24; iTRAQ4plex(K)@27	cleaved T-T@N-term	0.1	3094.71	1032.58	3094.64	1032.56	3	10.3	18.1	36.3	63.7	1.8
			86	SVSELPIMHQDWLNGK	iTRAQ4plex@N-term; Oxidation(D)@11; Oxidation(W)@12; Lys->Gln@16		0.0	2029.01	677.34	2028.97	677.33	3	271.3	186.8	59.2	40.8	0.7
			85	APQVYTIPPPKEQMAK	iTRAQ4plex@N-term; iTRAQ4plex(K)@11; Deamidated(Q)@13; Met->Leu@14; Lys->Arg@16		-0.1	2096.12	699.71	2096.19	699.74	3	191.1	128.5	59.8	40.2	0.7
			83	APQVYTIPPPK	iTRAQ4plex@N-term; Lys->Arg@11		0.0	1381.76	461.59	1381.78	461.60	3	25.0	66.1	27.5	72.5	2.6
			80	NTQPIMDTDGSYFIYSK	iTRAQ4plex@N-term; Ile->Trp@14; iTRAQ4plex(K)@17		0.0	2340.14	781.05	2340.10	781.04	3	306.2	232.2	56.9	43.1	0.8
			77	SVSELPIMHQDWLNGKEF	iTRAQ4plex@N-term; iTRAQ4plex(K)@16; Phe->Met@18	cleaved F-K@C-term	0.1	2401.25	601.32	2401.20	601.31	4	1.7	3.2	35.0	65.0	1.9
			75	SVSELPIMHQDWLNGK	iTRAQ4plex@N-term; Deamidated(Q)@10; Methylthio(D)@11; iTRAQ4plex(K)@16		0.1	2188.14	548.04	2188.09	548.03	4	413.8	343.0	54.7	45.3	0.8
			73	VNSAAFPAPIEK	iTRAQ4plex@N-term; Methyl(I)@10; Methyl(E)@11; iTRAQ4plex(K)@12		-0.1	1558.80	520.61	1558.90	520.64	3	884.3	2088.8	29.7	70.3	2.4
			72	SLSHSPG	iTRAQ4plex@N-term	cleaved G-K@C-term	0.0	827.45	414.73	827.43	414.72	2	1592.5	881.5	64.4	35.6	0.6
			71	VNSAAFPAPIEK	iTRAQ4plex@N-term; Deamidated(N)@2; Lys->Gln@12		0.0	1387.70	694.86	1387.71	694.86	2	21790.2	12645.1	63.3	36.7	0.6
			70	DDPEVQFSWFVDDVEVHTAQTQPR	iTRAQ4plex@N-term		0.1	2988.49	748.13	2988.40	748.11	4	1.7	8.7	16.3	83.7	5.1
			66	SLSHSPG	iTRAQ4plex@N-term; Dehydrated(S)@5	cleaved G-K@C-term	0.0	809.45	405.73	809.42	405.71	2	177.8	91.9	65.9	34.1	0.5
			62	DDPEVQFSWFVDDVEVHTAQTQPR	iTRAQ4plex@N-term; Gln->Arg@22		0.1	3016.52	755.14	3016.44	755.12	4	8.6	16.0	34.9	65.1	1.9
			60	EQMAK	iTRAQ4plex@N-term; iTRAQ4plex(K)@5		0.0	893.53	447.77	893.49	447.75	2	29.9	32.5	47.9	52.1	1.1
			53	TPPSVYPLAPGSAAQTNSMVTLGCLVK	iTRAQ4plex@N-term; Pyridylethyl(C)@24; iTRAQ4plex(K)@27	cleaved T-T@N-term	-0.1	3094.54	1032.52	3094.64	1032.56	3	31.8	31.3	50.4	49.6	1.0
			45	SVSELPIMHQDWLNGK	iTRAQ4plex@N-term; Asp->Asn@11; Lys->Gln@16		0.0	1996.01	666.34	1996.00	666.34	3					
			41	SLSHSPG	iTRAQ4plex@N-term; Dehydrated(S)@5	cleaved G-K@C-term	0.1	809.47	405.74	809.42	405.71	2					
			38	EQMAK	iTRAQ4plex@N-term; iTRAQ4plex(K)@5		0.0	893.54	447.78	893.49	447.75	2					
			31	TTPPSVYPLAPGSAAQTNSMVTLGCLVK	iTRAQ4plex@N-term; Deamidated(N)@18; Pyridylethyl(C)@25; Lys->Gln@28		0.0	3052.52	764.14	3052.54	764.14	4					
			27	SVSELPIMHQDWLNGK	iTRAQ4plex@N-term; Dehydrated(D)@11; iTRAQ4plex(K)@16		0.0	2123.08	531.78	2123.11	531.78	4					
			24	SVSELPIMHQDWLNGK	iTRAQ4plex@N-term; Lys->Arg@16		0.0	2025.05	676.03	2025.02	676.01	3					
			21	EQMAK	iTRAQ4plex@N-term; iTRAQ4plex(K)@5		0.0	893.53	447.77	893.49	447.75	2					
			17	NTQPIMDTDGSYFIYSK	iTRAQ4plex@N-term; Dehydrated(D)@9; Ile->Val@14; iTRAQ4plex(K)@17		-0.1	2235.02	746.02	2235.08	746.03	3					
			17	SLSHSPG	iTRAQ4plex@N-term; Dehydrated(S)@5	cleaved G-K@C-term	0.0	809.45	405.73	809.42	405.71	2					
			17	NTQPIMDTDGSYFIYSK	iTRAQ4plex@N-term; Ile->Val@14; iTRAQ4plex(K)@17		0.0	2253.09	752.04	2253.09	752.04	3					
			17	NTQPIMDTDGSYFIYSK	iTRAQ4plex@N-term; Ile->Val@14; iTRAQ4plex(K)@17		0.0	2253.04	752.02	2253.09	752.04	3					
			17	NTQPIMDTDGSYFIYSK	iTRAQ4plex@N-term; Ile->Val@14; iTRAQ4plex(K)@17		0.0	2253.10	752.04	2253.09	752.04	3					
			17	NTQPIMDTDGSYFIYSK	iTRAQ4plex@N-term; Ile->Val@14; iTRAQ4plex(K)@17		0.0	2253.09	752.04	2253.09	752.04	3					
			17	NTQPIMDTDGSYFIYSK	iTRAQ4plex@N-term; Ile->Val@14; iTRAQ4plex(K)@17		0.0	2253.09	752.04	2253.09	752.04	3					
			17	NTQPIMDTDGSYFIYSK	iTRAQ4plex@N-term; Ile->Val@14; iTRAQ4plex(K)@17		0.0	2253.12	752.05	2253.09	752.04	3					
			17	NTQPIMDTDGSYFIYSK	iTRAQ4plex@N-term; Ile->Val@14; Lys->Arg@17		0.0	2137.00	713.34	2136.99	713.34	3					
			17	NTQPIMDTDGSYFIYSK	iTRAQ4plex@N-term; Ile->Val@14; Lys->Arg@17		0.0	2137.02	713.35	2136.99	713.34	3					
			17	NTQPIMDTDGSYFIYSK	iTRAQ4plex@N-term; Ile->Val@14; Lys->Arg@17		0.0	2136.99	713.34	2136.99	713.34	3					
			17	NTQPIMDTDGSYFIYSK	iTRAQ4plex@N-term; Ile->Val@14; Lys->Gln@17		0.0	2108.98	704.00	2108.95	703.99	3					
			17	NTQPIMDTDGSYFIYSK	iTRAQ4plex@N-term; Oxidation(M)@6; Ile->Val@14; iTRAQ4plex(K)@17		-0.1	2269.03	757.35	2269.08	757.37	3					
			17	NTQPIMDTDGSYFIYSK	iTRAQ4plex@N-term; Oxidation(M)@6; Ile->Val@14; iTRAQ4plex(K)@17		-0.1	2269.03	757.35	2269.08	757.37	3					
			17	NTQPIMDTDGSYFIYSK	iTRAQ4plex@N-term; Oxidation(M)@6; Ile->Val@14; iTRAQ4plex(K)@17		-0.1	2269.01	757.34	2269.08	757.37	3					
			17	NTQPIMDTDGSYFIYSK	iTRAQ4plex@N-term; Oxidation(M)@6; Ile->Val@14; iTRAQ4plex(K)@17		0.0	2269.03	757.35	2269.08	757.37	3					
			17	NTQPIMDTDGSYFIYSK	iTRAQ4plex@N-term; Oxidation(M)@6; Ile->Val@14; Lys->Gln@17		0.0	2124.94	709.32	2124.94	709.32	3					
			11	VNSAAFPAPIEK	iTRAQ4plex@N-term; Pro->Asn@7; Lys->Gln@12		0.0	1403.74	702.88	1403.72	702.87	2					
																Average	1.3
																Stdev	0.8
IPI:IPI00315359.1	KCNAB2	Potassium voltage-gated channel, shaker-related subfamily, beta member 2	99	AMTHVINQGMAMYWGTSR	iTRAQ4plex@N-term		0.0	2197.00	733.34	2197.04	733.35	3	36.9	40.8	47.5	52.5	1.1
			99	QTGSPGMIYSTR	iTRAQ4plex@N-term		0.0	1440.68	721.35	1440.72	721.36	2	25936.1	12828.8	66.9	33.1	0.5
			99	MYPESTTGSPAR	iTRAQ4plex@N-term		0.0	1439.69	720.85	1439.68	720.85	2	14307.5	7065.4	66.9	33.1	0.5
			99	QTGSPGMIYSTR	iTRAQ4plex@N-term		0.0	1440.68	721.35	1440.72	721.36	2	12208.2	5929.2	67.3	32.7	0.5
			99	ELQAIAER	iTRAQ4plex@N-term		0.0	1072.57	537.29	1072.60	537.31	2	9427.1	9469.9	49.9	50.1	1.0
			99	YDSGIPPYSR	iTRAQ4plex@N-term		0.0	1297.62	649.82	1297.64	649.83	2	6049.2	4214.3	58.9	41.1	0.7
			99	YDSGIPPYSR	iTRAQ4plex@N-term		0.0	1297.61	649.81	1297.64	649.83	2	5363.7	3795.4	58.6	41.4	0.7
			99	MYPESTTGSPAR	iTRAQ4plex@N-term		0.0	1439.72	720.87	1439.68	720.85	2	4349.6	2004.0	68.5	31.5	0.5
			99	ELQAIAER	iTRAQ4plex@N-term		0.0	1072.57	537.29	1072.60	537.31	2	3799.7	3479.8	52.2	47.8	0.9
			99	MYPESTTGSPAR	iTRAQ4plex@N-term		0.0	1439.71	720.86	1439.68	720.85	2	3123.0	1416.4	68.8	31.2	0.5
			99	YDSGIPPYSR	iTRAQ4plex@N-term		1.0	1298.67	650.34	1297.64	649.83	2	1362.9	962.4	58.6	41.4	0.7
			99	YDSGIPPYSR	iTRAQ4plex@N-term		0.0	1297.61	649.81	1297.64	649.83	2	1203.9	956.8	55.7	44.3	0.8
			99	YDSGIPPYSR	iTRAQ4plex@N-term		0.0	1297.61	649.81	1297.64	649.83	2	975.2	655.4	59.8	40.2	0.7
			99	YDSGIPPYSR	iTRAQ4plex@N-term		0.0	1297.66	649.84	1297.64	649.83	2	861.5	760.5	53.1	46.9	0.9
			99	YDSGIPPYSR	iTRAQ4plex@N-term		0.0	1297.62	649.82	1297.64	649.83	2	427.7	314.4	57.6	42.4	0.7
			99	YDSGIPPYSR	iTRAQ4plex@N-term		0.0	1297.67	649.84	1297.64	649.83	2	321.7	359.2	47.3	52.7	1.1
			99	AMTHVINQGMAMYWGTSR	iTRAQ4plex@N-term		-0.1	2196.99	733.34	2197.04	733.35	3	305.5	251.6	54.8	45.2	0.8
			99	WSSMEIMEAYSVAR	iTRAQ4plex@N-term		0.0	1802.86	601.96	1802.85	601.96	3	59.1	34.5	63.2	36.8	0.6
			99	WSSMEIMEAYSVAR	iTRAQ4plex@N-term		0.0	1802.86	601.96	1802.85	601.96	3	57.4	32.4	63.9	36.1	0.6
			99	LQLEYVDVVFANRPDPNTPMEETVR	iTRAQ4plex@N-term		0.1	3075.60	769.91	3075.55	769.89	4	37.0	38.6	48.9	51.1	1.0
			99	LQLEYVDVVFANRPDPNTPMEETVR	iTRAQ4plex@N-term		0.0	3075.59	769.90	3075.55	769.89	4	15.9	16.8	48.6	51.4	1.1
			99	WSSMEIMEAYSVAR	iTRAQ4plex@N-term		0.0	1802.86	601.96	1802.85	601.96	3	12.2	15.8	43.6	56.4	1.3
			99	MYPESTTGSPARLSLR	iTRAQ4plex@N-term; Deamidated(R)@12	missed R-L@12	0.0	1909.97	637.66	1909.97	637.66	3	144.1	111.5	56.4	43.6	0.8
			99	MYPESTTGSPARLSLR	iTRAQ4plex@N-term; Deamidated(R)@12	missed R-L@12	0.0	1909.97	637.66	1909.97	637.66	3	116.6	114.8	50.4	49.6	1.0
			99	LSLRQTGSPGMIYSTR	iTRAQ4plex@N-term; Deamidated(R)@4	missed R-Q@4	0.0	1910.98	638.00	1911.00	638.01	3	242.0	189.0	56.1	43.9	0.8
			99	LSLRQTGSPGMIYSTR	iTRAQ4plex@N-term; Deamidated(R)@4	missed R-Q@4	-0.1	1910.95	637.99	1911.00	638.01	3	229.1	191.6	54.5	45.5	0.8
			99	LSLRQTGSPGMIYSTR	iTRAQ4plex@N-term; Deamidated(R)@4	missed R-Q@4	0.0	1910.97	638.00	1911.00	638.01	3	64.2	46.2	58.2	41.8	0.7
			99	LSSSIVHEIDSILGNKPYSK	iTRAQ4plex@N-term; iTRAQ4plex(K)@16; No iTRAQ4plex(K)@20		0.0	2474.33	619.59	2474.36	619.60	4	19.6	14.5	57.5	42.5	0.7
			99	LSSSIVHEIDSILGNKPYS	iTRAQ4plex@N-term; iTRAQ4plex(K)@16; Tyr->Phe@18	cleaved S-K@C-term	0.0	2330.23	583.56	2330.27	583.58	4	23.3	14.3	61.9	38.1	0.6
			99	SSLVITTK	iTRAQ4plex@N-term; iTRAQ4plex(K)@8		0.0	1135.70	568.86	1135.71	568.86	2	3457.6	3373.3	50.6	49.4	1.0
			99	LSSSIVHEIDSILGNKPY	iTRAQ4plex@N-term; Lys->Gln@16	cleaved Y-S@C-term	0.0	2115.14	706.05	2115.10	706.04	3	28.3	27.2	51.0	49.0	1.0
			99	QTGSPGMIYSTR	iTRAQ4plex@N-term; Oxidation(M)@7		0.0	1456.73	729.37	1456.71	729.36	2	1312.3	692.9	65.4	34.6	0.5
			99	IGVGAMTWSPLACGIVSGK	iTRAQ4plex@N-term; Pyridylethyl(C)@13; Lys->Gln@19		0.1	2095.12	699.38	2095.07	699.36	3	171.3	118.0	59.2	40.8	0.7
			99	AMTHVINQGMAMYWGTSR	iTRAQ4plex@N-term; Ser->Asp@17		0.1	2225.09	742.70	2225.03	742.68	3	12.2	16.9	41.9	58.1	1.4
			99	YDSGIPPYSR	iTRAQ4plex@N-term; Ser->Asp@9		0.0	1325.68	663.85	1325.64	663.83	2	701.0	1797.2	28.1	71.9	2.6
			98	NEGVSSVLLGASNAEQLMENIGAIQVLPK	iTRAQ4plex@N-term; iTRAQ4plex(K)@29		-1.0	3267.72	817.94	3268.76	818.20	4	5.3	4.1	56.3	43.7	0.8
			97	MYPESTTGSPAR	iTRAQ4plex@N-term		0.0	1439.73	720.87	1439.68	720.85	2	589.2	300.8	66.2	33.8	0.5
			96	QTGSPGMIYSTR	iTRAQ4plex@N-term		3.0	1443.71	722.86	1440.72	721.36	2	2629.8	1395.6	65.3	34.7	0.5
			92	ILSEEGR	iTRAQ4plex@N-term		0.0	946.53	474.27	946.52	474.27	2	21561.5	17817.3	54.8	45.2	0.8
			91	AEVVLGNIIK	iTRAQ4plex@N-term; iTRAQ4plex(K)@10		0.0	1342.83	448.62	1342.84	448.62	3	46.3	85.0	35.3	64.7	1.8
			91	LSSSIVHEIDSILGNKPYSK	iTRAQ4plex@N-term; iTRAQ4plex(K)@16; Lys->Gln@20		0.0	2474.35	495.88	2474.33	495.87	5	5.5	0.0	100.0	0.0	0.0
			90	MYPESTTGSPAR	iTRAQ4plex@N-term		3.1	1442.74	722.38	1439.68	720.85	2	317.9	213.0	59.9	40.1	0.7
			90	NEGVSSVLLGASNAEQLMENIGAIQVLPK	iTRAQ4plex@N-term; iTRAQ4plex(K)@29		0.0	3268.76	818.20	3268.76	818.20	4	10.8	5.0	68.3	31.7	0.5
			84	AEVVLGNIIK	iTRAQ4plex@N-term; Lys->Gln@10		0.1	1198.76	600.39	1198.70	600.36	2	97.0	99.3	49.4	50.6	1.0
			79	WSSMEIMEAYSVAR	iTRAQ4plex@N-term; Ala->Val@13		0.0	1830.87	611.30	1830.88	611.30	3	44.2	41.8	51.4	48.6	0.9
			79	LQLEYVDVVFANRPDPNTPMEETVR	iTRAQ4plex@N-term; Deamidated(N)@17; Dimethyl(R)@25		0.0	3104.56	777.15	3104.56	777.15	4	8.5	16.9	33.5	66.5	2.0
			76	ILSEEGR	iTRAQ4plex@N-term		0.0	946.54	474.28	946.52	474.27	2	4008.5	4075.2	49.6	50.4	1.0
			75	MYPESTTGSPAR	iTRAQ4plex@N-term; Oxidation(M)@1		0.0	1455.72	728.87	1455.68	728.85	2	217.4	110.9	66.2	33.8	0.5
			60	YGSPK	iTRAQ4plex@N-term; iTRAQ4plex(K)@5		0.0	838.51	420.26	838.48	420.25	2	62.8	33.5	65.2	34.8	0.5
			33	NEGVSSVLLGASNAEQLMENIGAIQVLPK	iTRAQ4plex@N-term; iTRAQ4plex(K)@29		-0.9	3267.88	817.98	3268.76	818.20	4					
			31	VEVQLPELFHK	iTRAQ4plex@N-term; Lys->Gln@11		0.0	1481.83	494.95	1481.80	494.94	3					
			30	GYQWLK	iTRAQ4plex@N-term; iTRAQ4plex(K)@6		0.0	1081.62	541.82	1081.62	541.82	2					
			29	ILSEEGR	iTRAQ4plex@N-term; Dimethyl(R)@7		0.0	974.55	488.28	974.55	488.28	2					
			26	IGVGAMTWSPLACGIVSGK	iTRAQ4plex@N-term; Oxidation(M)@6; Trp->Tyr@8; Pyridylethyl(C)@13; Lys->Gln@19		0.0	2088.05	697.02	2088.05	697.02	3					
			18	YDSGIPPYSR	iTRAQ4plex@N-term		-8.0	1289.67	645.84	1297.64	649.83	2					
			16	LSSSIVHEIDSILGNKP	iTRAQ4plex@N-term; iTRAQ4plex(K)@16; Pro->Asp@17	cleaved P-Y@C-term	0.0	2114.15	705.73	2114.15	705.72	3					
																Average	0.8
																Stdev	0.4

IPI:IPI00761937.1	UBAP2L	Ubiquitin-associated protein 2-like	99	AINVLLEGNPDTHSWEMVGK	iTRAQ4plex@N-term; iTRAQ4plex(K)@20		-0.1	2497.22	625.31	2497.29	625.33	4	19.0	29.6	39.2	60.8	1.6
			99	TAQALAQLAAQHSQSGSTTTSSWDMGSTTQSPSLVQYDLK	iTRAQ4plex@N-term; Deamidated(Q)@30; iTRAQ4plex(K)@40		0.0	4471.20	895.25	4471.17	895.24	5	8.5	17.1	33.3	66.7	2.0
			99	DGSLASNPYSGDLTK	iTRAQ4plex@N-term; iTRAQ4plex(K)@15		0.0	1811.87	604.97	1811.91	604.98	3	175.5	104.8	62.6	37.4	0.6
			99	DGSLASNPYSGDLTK	iTRAQ4plex@N-term; iTRAQ4plex(K)@15		0.0	1811.92	604.98	1811.91	604.98	3	105.1	77.3	57.6	42.4	0.7
			99	TATEEWGTEDWNEDLSETK	iTRAQ4plex@N-term; iTRAQ4plex(K)@19		0.0	2528.18	843.73	2528.14	843.72	3	75.1	48.2	60.9	39.1	0.6
			99	TATEEWGTEDWNEDLSETK	iTRAQ4plex@N-term; iTRAQ4plex(K)@19		-0.1	2528.09	843.70	2528.14	843.72	3	63.9	54.0	54.2	45.8	0.8
			99	TPSSMENDSSNLDPSQAPSLAQPLVFSNSK	iTRAQ4plex@N-term; iTRAQ4plex(K)@30		-1.0	3434.67	859.68	3435.67	859.92	4	22.7	28.5	44.4	55.6	1.3
			99	TAQALAQLAAQHSQSGSTTTSSWDMGSTTQSPSLVQYDLK	iTRAQ4plex@N-term; iTRAQ4plex(K)@40		0.1	4470.24	895.06	4470.19	895.04	5	35.6	28.0	56.0	44.0	0.8
			99	GFGDVGEAK	iTRAQ4plex@N-term; iTRAQ4plex(K)@9		0.0	1166.62	584.32	1166.62	584.32	2	2642.7	1591.1	62.4	37.6	0.6
			99	IDLAVLLGK	iTRAQ4plex@N-term; iTRAQ4plex(K)@9		0.0	1228.83	615.42	1228.80	615.41	2	82.7	89.0	48.2	51.8	1.1
			99	GGSTTGSQFLEQFK	iTRAQ4plex@N-term; Lys->Gln@14		0.0	1629.77	815.89	1629.78	815.90	2	342.8	183.1	65.2	34.8	0.5
			99	GGSTTGSQFLEQFK	iTRAQ4plex@N-term; Lys->Gln@14		0.0	1629.78	815.90	1629.78	815.90	2	337.6	175.7	65.8	34.2	0.5
			99	GGSTTGSQFLEQFK	iTRAQ4plex@N-term; Lys->Gln@14		0.0	1629.79	815.90	1629.78	815.90	2	155.7	94.9	62.1	37.9	0.6
			99	QNAISQPASGSTFSHHSMVSMLGK	iTRAQ4plex@N-term; Lys->Gln@24		0.0	2645.23	662.32	2645.24	662.32	4	54.1	22.9	70.2	29.8	0.4
			99	NGFSSVQATQLQTTQSVEGATGSAVK	iTRAQ4plex@N-term; Lys->Gln@26		0.0	2739.31	914.11	2739.34	914.12	3	14.0	17.9	43.8	56.2	1.3
			99	TPSSMENDSSNLDPSQAPSLAQPLVFSNSK	iTRAQ4plex@N-term; Lys->Gln@30		0.0	3291.58	1098.20	3291.53	1098.18	3	24.9	18.5	57.5	42.5	0.7
			99	IDLAVLLGK	iTRAQ4plex@N-term; Lys->Gln@9		0.0	1084.71	543.36	1084.66	543.34	2	32.0	24.9	56.3	43.7	0.8
			99	IDLAVLLGK	iTRAQ4plex@N-term; Lys->Gln@9		0.0	1084.70	543.36	1084.66	543.34	2	19.8	7.9	71.4	28.6	0.4
			99	TATEEWGTEDWNEDLSETK	iTRAQ4plex@N-term; No iTRAQ4plex(K)@19		0.0	2384.07	795.70	2384.04	795.69	3	25.4	5.6	82.1	17.9	0.2
			99	STSAPQMSPGSSDNQSSSPQPAQQK	iTRAQ4plex@N-term; No iTRAQ4plex(K)@25		0.0	2675.22	892.75	2675.22	892.75	3	50.0	32.9	60.4	39.6	0.7
			98	DGGQTESNEEGKENR	iTRAQ4plex@N-term; iTRAQ4plex(K)@12		0.1	1937.02	646.68	1936.90	646.64	3	25.3	8.8	74.1	25.9	0.3
			98	GGSTTGSQFLEQFK	iTRAQ4plex@N-term; iTRAQ4plex(K)@14		1.0	1774.88	592.64	1773.91	592.31	3	157.3	102.2	60.6	39.4	0.6
			94	AINVLLEGNPDTHSWEMVGK	iTRAQ4plex@N-term; iTRAQ4plex(K)@20		-0.1	2497.21	625.31	2497.29	625.33	4	68.2	87.4	43.8	56.2	1.3
			42	DLTQAK	iTRAQ4plex@N-term; iTRAQ4plex(K)@6		0.0	962.59	482.30	962.56	482.29	2					
																Average	0.8
																Stdev	0.4
IPI:IPI00756692.1	ELMO2	Engulfment and cell motility protein 2	99	EPSSYDFVYHYG	iTRAQ4plex@N-term		0.0	1606.70	804.36	1606.71	804.36	2	764.0	472.0	61.8	38.2	0.6
			99	SLSYSEILR	iTRAQ4plex@N-term		0.0	1210.65	606.33	1210.67	606.34	2	4182.5	2170.4	65.8	34.2	0.5
			99	SLSYSEILR	iTRAQ4plex@N-term		0.0	1210.68	606.35	1210.67	606.34	2	1454.1	768.4	65.4	34.6	0.5
			99	NGTILQLAVSPSR	iTRAQ4plex@N-term		0.0	1498.84	750.42	1498.86	750.44	2	475.3	264.7	64.2	35.8	0.6
			99	MSQDDFQSPPIVELR	iTRAQ4plex@N-term		0.0	1904.95	635.99	1904.94	635.99	3	94.9	56.9	62.5	37.5	0.6
			99	MSQDDFQSPPIVELR	iTRAQ4plex@N-term		0.0	1904.91	635.98	1904.94	635.99	3	83.9	57.3	59.4	40.6	0.7
			99	NGTILQLAVSPSR	iTRAQ4plex@N-term		0.0	1498.83	500.62	1498.86	500.63	3	37.3	29.9	55.5	44.5	0.8
			99	VAIEWPGANAQLLEIDQK	iTRAQ4plex@N-term; iTRAQ4plex(K)@18		0.0	2282.27	761.76	2282.25	761.76	3	48.2	31.8	60.2	39.8	0.7
			99	VAIEWPGANAQLLEIDQK	iTRAQ4plex@N-term; iTRAQ4plex(K)@18		0.0	2282.22	761.75	2282.25	761.76	3	31.9	29.2	52.2	47.8	0.9
			99	SLAILESMVLNSQSLYQK	iTRAQ4plex@N-term; iTRAQ4plex(K)@18		-1.0	2310.25	771.09	2311.27	771.43	3	6.9	10.6	39.6	60.4	1.5
			99	LLDLENIQIPEAPPPVPK	iTRAQ4plex@N-term; iTRAQ4plex(K)@18		0.1	2270.39	757.80	2270.31	757.78	3	5.5	0.0	100.0	0.0	0.0
			99	LLDLENIQIPEAPPPVPK	iTRAQ4plex@N-term; Lys->Arg@18		0.1	2154.29	719.10	2154.22	719.08	3	10.7	8.3	56.3	43.7	0.8
			99	IQPEILELIK	iTRAQ4plex@N-term; Lys->Gln@10		0.0	1338.82	670.42	1338.79	670.40	2	494.0	252.5	66.2	33.8	0.5
			99	LLDLENIQIPEAPPPVPK	iTRAQ4plex@N-term; Lys->Gln@18		0.1	2126.24	709.75	2126.17	709.73	3	21.4	14.4	59.8	40.2	0.7
			99	EVCDGWSLPNPEYYTLR	iTRAQ4plex@N-term; Pyridylethyl(C)@3		0.0	2290.05	764.36	2290.09	764.37	3	27.8	40.1	41.0	59.0	1.4
			98	EPSSYDFVYHYG	iTRAQ4plex@N-term		0.0	1606.67	804.34	1606.71	804.36	2	259.2	217.8	54.3	45.7	0.8
			98	MSQDDFQSPPIVELR	iTRAQ4plex@N-term; Dimethyl(R)@15		0.0	1933.00	645.34	1932.97	645.33	3	8.5	18.1	31.9	68.1	2.1
			98	IQPEILELIK	iTRAQ4plex@N-term; iTRAQ4plex(K)@10		1.0	1483.91	495.64	1482.93	495.32	3	69.3	57.1	54.8	45.2	0.8
			98	SDLDTLLSMEMK	iTRAQ4plex@N-term; Lys->Gln@12		0.0	1525.73	763.87	1525.71	763.86	2	53.3	43.6	55.0	45.0	0.8
			98	LLDLENIQIPEAPPPVPK	iTRAQ4plex@N-term; Lys->Gln@18		0.0	2126.21	709.74	2126.17	709.73	3	26.7	20.7	56.3	43.7	0.8
			98	YADGPQLYVTEQAK	iTRAQ4plex@N-term; Lys->Trp@14		0.0	1783.84	892.93	1783.85	892.93	2	522.3	330.4	61.2	38.8	0.6
			98	YADGPQLYVTEQAK	iTRAQ4plex@N-term; Lys->Trp@14		0.0	1783.87	595.63	1783.85	595.63	3	66.9	71.9	48.2	51.8	1.1
			98	YADGPQLYVTEQAK	iTRAQ4plex@N-term; Lys->Trp@14		0.1	1783.91	595.64	1783.85	595.63	3	42.5	38.4	52.5	47.5	0.9
			97	SLSYSEILR	iTRAQ4plex@N-term; Dimethyl(R)@9		0.0	1238.69	620.35	1238.70	620.36	2	313.5	683.0	31.5	68.5	2.2
			97	SDLDTLLSMEMK	iTRAQ4plex@N-term; iTRAQ4plex(K)@12		0.0	1669.83	557.62	1669.85	557.62	3	42.6	36.4	53.9	46.1	0.9
			92	IQPEILELIK	iTRAQ4plex@N-term; Lys->Gln@10		0.1	1338.85	670.43	1338.79	670.40	2	404.0	210.2	65.8	34.2	0.5
			91	IPVADIK	iTRAQ4plex@N-term; iTRAQ4plex(K)@7		0.0	1042.63	522.32	1042.66	522.34	2	4411.8	3354.5	56.8	43.2	0.8
			81	IQPEILELIK	iTRAQ4plex@N-term; iTRAQ4plex(K)@10		2.0	1484.93	495.98	1482.93	495.32	3	38.8	39.8	49.4	50.6	1.0
			71	IQPEILELIK	iTRAQ4plex@N-term; Lys->Arg@10		0.1	1366.89	684.45	1366.83	684.42	2	36.0	66.9	35.0	65.0	1.9
			58	SLSYSEILR	iTRAQ4plex@N-term		0.0	1210.65	606.33	1210.67	606.34	2	332.3	221.0	60.1	39.9	0.7
			29	IQPEILELIK	iTRAQ4plex@N-term; Lys->Gln@10		0.0	1338.82	670.42	1338.79	670.40	2					
																Average	0.9
																Stdev	0.5
IPI:IPI00556847.1	IGK	Immunoglobulin kappa chain complex	99	DSTYSMSSTLTLTK	iTRAQ4plex@N-term; Lys->Gln@14		0.0	1677.79	839.90	1677.79	839.90	2	917.9	968.9	48.6	51.4	1.1
			99	QNGVLNSWTDQDSK	iTRAQ4plex@N-term; Asp->Pro@10; iTRAQ4plex(K)@14		-0.1	1860.90	621.31	1860.96	621.33	3	424.4	454.7	48.3	51.7	1.1
			99	QNGVLNSWTDQDSK	iTRAQ4plex@N-term; Deamidated(N)@2; iTRAQ4plex(K)@14		-0.1	1879.83	627.62	1879.92	627.65	3	1523.3	1579.8	49.1	50.9	1.0
			99	QNGVLNSWTDQDSK	iTRAQ4plex@N-term; Deamidated(N)@2; iTRAQ4plex(K)@14		0.0	1879.93	627.65	1879.92	627.65	3	380.1	464.9	45.0	55.0	1.2
			99	QNGVLNSWTDQDSK	iTRAQ4plex@N-term; Deamidated(N)@2; iTRAQ4plex(K)@14		0.0	1879.91	627.64	1879.92	627.65	3	287.4	398.5	41.9	58.1	1.4
			99	QNGVLNSWTDQDSK	iTRAQ4plex@N-term; Deamidated(N)@2; Lys->Arg@14		0.0	1763.82	882.92	1763.82	882.92	2	511.7	1169.6	30.4	69.6	2.3
			99	QNGVLNSWTDQDSK	iTRAQ4plex@N-term; Deamidated(N)@2; Lys->Arg@14		0.0	1763.82	588.95	1763.82	588.95	3	93.2	203.2	31.4	68.6	2.2
			99	QNGVLNSWTDQDSK	iTRAQ4plex@N-term; Deamidated(N)@2; Lys->Gln@14		0.0	1735.73	868.87	1735.78	868.90	2	4267.8	3740.3	53.3	46.7	0.9
			99	QNGVLNSWTDQDSK	iTRAQ4plex@N-term; Deamidated(N)@2; Lys->Gln@14		0.0	1735.81	868.91	1735.78	868.90	2	1381.3	1309.2	51.3	48.7	0.9
			99	QNGVLNSWTDQDSK	iTRAQ4plex@N-term; Deamidated(N)@2; Ser->Asp@13; iTRAQ4plex(K)@14		0.0	1907.95	636.99	1907.91	636.98	3	144.1	413.6	25.8	74.2	2.9
			99	QNGVLNSWTDQDSK	iTRAQ4plex@N-term; Dehydrated(S)@7; Lys->Gln@14		0.0	1716.79	573.27	1716.78	573.27	3	138.7	109.7	55.8	44.2	0.8
			99	DSTYSMSSTLTLTKDEYER	iTRAQ4plex@N-term; Dehydrated(S)@8; Thr->Asn@13; iTRAQ4plex(K)@14; Oxidation(D)@15		0.0	2525.16	842.73	2525.18	842.74	3	84.9	80.0	51.5	48.5	0.9
			99	DSTYSMSSTLTLTKDEYER	iTRAQ4plex@N-term; Dehydrated(T)@11; Thr->Asn@13; iTRAQ4plex(K)@14; Oxidation(D)@15		0.0	2525.14	842.72	2525.18	842.74	3	105.8	107.6	49.6	50.4	1.0
			99	QNGVLNSWTDQDSK	iTRAQ4plex@N-term; Dehydrated(T)@9; Lys->Gln@14		0.1	1716.83	573.29	1716.78	573.27	3	100.5	103.5	49.3	50.7	1.0
			99	DSTYSMSSTLTLTKDEYER	iTRAQ4plex@N-term; Dehydrated(T)@9; Thr->Asn@13; iTRAQ4plex(K)@14; Oxidation(D)@15		0.0	2525.16	842.73	2525.18	842.74	3	241.8	193.6	55.5	44.5	0.8
			99	DSTYSMSSTLTLTKDEYER	iTRAQ4plex@N-term; iTRAQ4plex(K)@14		0.0	2514.22	839.08	2514.20	839.08	3	2539.6	610.5	80.6	19.4	0.2
			99	QNGVLNSWTDQDSK	iTRAQ4plex@N-term; iTRAQ4plex(K)@14		-0.1	1878.87	627.30	1878.93	627.32	3	1865.9	2466.6	43.1	56.9	1.3
			99	DSTYSMSSTLTLTKDEYER	iTRAQ4plex@N-term; iTRAQ4plex(K)@14		-0.1	2514.12	839.05	2514.20	839.08	3	1682.4	431.3	79.6	20.4	0.3
			99	DSTYSMSSTLTLTKDEYER	iTRAQ4plex@N-term; iTRAQ4plex(K)@14		0.0	2514.20	839.07	2514.20	839.08	3	683.2	182.2	78.9	21.1	0.3
			99	DSTYSMSSTLTLTK	iTRAQ4plex@N-term; iTRAQ4plex(K)@14		-0.1	1821.88	608.30	1821.93	608.32	3	423.1	488.4	46.4	53.6	1.2
			99	DSTYSMSSTLTLTK	iTRAQ4plex@N-term; iTRAQ4plex(K)@14		0.0	1821.92	608.31	1821.93	608.32	3	355.6	384.8	48.0	52.0	1.1
			99	QNGVLNSWTDQDSK	iTRAQ4plex@N-term; iTRAQ4plex(K)@14		0.0	1878.94	627.32	1878.93	627.32	3	273.4	280.6	49.4	50.6	1.0
			99	DSTYSMSSTLTLTK	iTRAQ4plex@N-term; iTRAQ4plex(K)@14		0.0	1821.88	608.30	1821.93	608.32	3	196.9	320.6	38.0	62.0	1.6
			99	DSTYSMSSTLTLTK	iTRAQ4plex@N-term; iTRAQ4plex(K)@14		0.0	1821.89	608.30	1821.93	608.32	3	194.2	242.8	44.4	55.6	1.2
			99	DSTYSMSSTLTLTK	iTRAQ4plex@N-term; iTRAQ4plex(K)@14		0.0	1821.89	608.31	1821.93	608.32	3	150.1	200.1	42.9	57.1	1.3
			99	DSTYSMSSTLTLTK	iTRAQ4plex@N-term; iTRAQ4plex(K)@14		0.0	1821.88	608.30	1821.93	608.32	3	109.3	163.8	40.0	60.0	1.5
			99	DSTYSMSSTLTLTK	iTRAQ4plex@N-term; iTRAQ4plex(K)@14		0.0	1821.88	608.30	1821.93	608.32	3	97.1	96.0	50.3	49.7	1.0
			99	DSTYSMSSTLTLTKDEYER	iTRAQ4plex@N-term; iTRAQ4plex(K)@14; Dimethyl(R)@19		0.0	2542.20	848.41	2542.24	848.42	3	418.2	371.1	53.0	47.0	0.9
			99	DSTYSMSSTLTLTKDEYER	iTRAQ4plex@N-term; iTRAQ4plex(K)@14; Dimethyl(R)@19		0.0	2542.19	848.40	2542.24	848.42	3	198.1	187.6	51.4	48.6	0.9
			99	QNGVLNSWTDQDSKDSTYSMSSTLTLTK	iTRAQ4plex@N-term; iTRAQ4plex(K)@14; iTRAQ4plex(K)@28		0.0	3538.72	885.69	3538.75	885.69	4	102.9	38.4	72.8	27.2	0.4
			99	QNGVLNSWTDQDSKDSTYSMSSTLTLTK	iTRAQ4plex@N-term; iTRAQ4plex(K)@14; Lys->Gln@28		0.0	3394.61	849.66	3394.61	849.66	4	99.7	26.7	78.9	21.1	0.3
			99	QNGVLNSWTDQDSK	iTRAQ4plex@N-term; Lys->Arg@14		0.0	1762.83	588.62	1762.84	588.62	3	299.2	575.9	34.2	65.8	1.9
			99	QNGVLNSWTDQDSK	iTRAQ4plex@N-term; Lys->Gln@14		0.0	1734.76	868.39	1734.79	868.40	2	7731.8	5736.1	57.4	42.6	0.7
			99	QNGVLNSWTDQDSK	iTRAQ4plex@N-term; Lys->Gln@14		0.0	1734.78	868.40	1734.79	868.40	2	928.9	720.3	56.3	43.7	0.8
			99	QNGVLNSWTDQDSK	iTRAQ4plex@N-term; Lys->Gln@14		0.0	1734.84	579.29	1734.79	579.27	3	632.7	813.5	43.7	56.3	1.3
			99	DSTYSMSSTLTLTKDEYER	iTRAQ4plex@N-term; Lys->Gln@14		0.0	2370.06	791.03	2370.07	791.03	3	464.1	117.8	79.8	20.2	0.3
			99	DSTYSMSSTLTLTKDEYER	iTRAQ4plex@N-term; Lys->Gln@14		0.0	2370.06	791.03	2370.07	791.03	3	312.1	72.7	81.1	18.9	0.2
			99	DSTYSMSSTLTLTK	iTRAQ4plex@N-term; Lys->Gln@14		0.1	1677.85	560.29	1677.79	560.27	3	6.6	20.4	24.4	75.6	3.1
			99	DSTYSMSSTLTLTKDEYER	iTRAQ4plex@N-term; Lys->Gln@14; Dimethyl(R)@19		0.0	2398.09	800.37	2398.10	800.37	3	186.5	152.4	55.0	45.0	0.8
			99	DSTYSMSSTLTLTKDEYER	iTRAQ4plex@N-term; Lys->Gln@14; Glu->Arg@18; Deamidated(R)@19		0.0	2398.07	800.37	2398.11	800.38	3	125.1	81.0	60.7	39.3	0.6
			99	DSTYSMSSTLTLTK	iTRAQ4plex@N-term; Oxidation(M)@6; Lys->Gln@14		0.0	1693.80	847.91	1693.78	847.90	2	87.3	113.7	43.4	56.6	1.3
			99	QNGVLNSWTDQDSK	iTRAQ4plex@N-term; Oxidation(W)@8; Lys->Gln@14		0.0	1750.83	876.42	1750.79	876.40	2	235.7	161.3	59.4	40.6	0.7
			99	FSGSGSGTDFTLKISR	iTRAQ4plex@N-term; Phe->Met@10; Thr->Gln@11; iTRAQ4plex(K)@13	missed K-I@13	0.0	1958.03	653.69	1958.01	653.68	3	409.7	305.2	57.3	42.7	0.7
			99	QNGVLNSWTDQDSK	iTRAQ4plex@N-term; Phospho(S)@7; Oxidation(W)@8; Asp->Gly@10; iTRAQ4plex(K)@14		0.0	1916.85	480.22	1916.89	480.23	4	97.8	78.7	55.4	44.6	0.8
			99	VEAEDLGVYYCLQATHFPRTFGGGTK	iTRAQ4plex@N-term; Pyridylethyl(C)@11; Ala->Thr@14; Lys->Glu@26	missed R-T@19	0.0	3138.50	785.63	3138.49	785.63	4	220.5	173.9	55.9	44.1	0.8
			99	VEAEDLGVYYCLQATHFPRTFGGGTK	iTRAQ4plex@N-term; Pyridylethyl(C)@11; Deamidated(Q)@13; Ala->Thr@14; Lys->Gln@26	missed R-T@19	0.0	3138.50	785.63	3138.49	785.63	4	47.3	57.9	44.9	55.1	1.2
			99	DSTYSMSSTLTLTKDEYER	iTRAQ4plex@N-term; Ser->Gln@8; Thr->Ala@13; iTRAQ4plex(K)@14		0.0	2525.17	842.73	2525.22	842.75	3	68.8	68.9	50.0	50.0	1.0
			99	DSTYSMSSTLTLTKDEYER	iTRAQ4plex@N-term; Thr->Asn@13; iTRAQ4plex(K)@14; Dehydrated(E)@18		-0.1	2509.13	628.29	2509.19	628.30	4	1118.0	1202.6	48.2	51.8	1.1
			99	FSGSGSGTDFTLKISR	iTRAQ4plex@N-term; Thr->Pro@11; iTRAQ4plex(K)@13	missed K-I@13	0.0	1943.01	648.68	1943.04	648.69	3	472.5	340.5	58.1	41.9	0.7
			99	DSTYSMSSTLTLTKDEYER	iTRAQ4plex@N-term; Thr->Pro@13; iTRAQ4plex(K)@14		0.0	2510.19	837.74	2510.21	837.74	3	270.7	203.2	57.1	42.9	0.8
			99	DSTYSMSSTLTLTKDEYER	iTRAQ4plex@N-term; Thr->Pro@13; iTRAQ4plex(K)@14		0.0	2510.19	837.74	2510.21	837.74	3	182.3	168.7	51.9	48.1	0.9
			98	FSGSGSGTDFTLK	iTRAQ4plex@N-term; iTRAQ4plex(K)@13		0.0	1590.80	531.27	1590.81	531.28	3	396.1	428.1	48.1	51.9	1.1
			98	QNGVLNSWTDQDSK	iTRAQ4plex@N-term; Ser->Oxoalanine(S)@7; Oxidation(W)@8; iTRAQ4plex(K)@14		0.0	1892.90	631.97	1892.91	631.98	3	192.6	238.5	44.7	55.3	1.2
			98	DSTYSMSSTLTLTKDEYER	iTRAQ4plex@N-term; Thr->Asn@13; iTRAQ4plex(K)@14; Dehydrated(E)@18		0.0	2509.19	628.31	2509.19	628.30	4	107.4	115.5	48.2	51.8	1.1
			98	TYLNWLLQR	iTRAQ4plex@N-term; Tyr->Phe@2	cleaved R-P@C-term	0.0	1333.77	667.89	1333.76	667.89	2	31.2	47.7	39.5	60.5	1.5
			98	TSTSPIVKSFNR	iTRAQ4plex@N-term; Val->Leu@7; iTRAQ4plex(K)@8; Dehydrated(S)@9	missed K-S@8	0.0	1619.88	540.97	1619.92	540.98	3	2180.6	1417.5	60.6	39.4	0.7
			97	QNGVLNSWTDQDSK	iTRAQ4plex@N-term; Dioxidation(W)@8; Lys->Gln@14		0.0	1766.79	884.40	1766.78	884.40	2	807.3	640.6	55.8	44.2	0.8
			97	DSTYSMSSTLTLTK	iTRAQ4plex@N-term; iTRAQ4plex(K)@14		0.0	1821.89	608.30	1821.93	608.32	3	29.8	86.6	25.6	74.4	2.9
			97	TSTSPIVK	iTRAQ4plex@N-term; Lys->Arg@8		0.0	1003.58	502.80	1003.58	502.80	2	6406.1	14164.1	31.1	68.9	2.2
			97	FSGSGSGTDFTLK	iTRAQ4plex@N-term; Lys->Gln@13		0.0	1446.67	724.34	1446.67	724.34	2	2854.7	1548.6	64.8	35.2	0.5
			97	FSGSGSGTDFTLK	iTRAQ4plex@N-term; Lys->Gln@13		0.0	1446.71	724.36	1446.67	724.34	2	1436.4	952.7	60.1	39.9	0.7
			97	FSGSGSGTDFTLK	iTRAQ4plex@N-term; Lys->Gln@13		0.0	1446.67	724.34	1446.67	724.34	2	1337.4	808.0	62.3	37.7	0.6
			97	FSGSGSGTDFTLK	iTRAQ4plex@N-term; Lys->Gln@13		0.0	1446.71	724.36	1446.67	724.34	2	590.9	403.7	59.4	40.6	0.7
			97	TYLNWLLQR	iTRAQ4plex@N-term; Tyr->Phe@2	cleaved R-P@C-term	0.0	1333.76	667.88	1333.76	667.89	2	503.3	998.3	33.5	66.5	2.0
			96	TSTSPIVK	iTRAQ4plex@N-term; iTRAQ4plex(K)@8		0.0	1119.63	560.82	1119.67	560.84	2	47126.8	27132.6	63.5	36.5	0.6
			95	LIYLVSK	iTRAQ4plex@N-term; iTRAQ4plex(K)@7	cleaved L-L@N-term	0.0	1122.69	562.35	1122.73	562.37	2	17362.1	14726.3	54.1	45.9	0.8
			95	TSTSPIVK	iTRAQ4plex@N-term; iTRAQ4plex(K)@8		0.0	1119.66	560.84	1119.67	560.84	2	19345.5	15414.0	55.7	44.3	0.8
			94	QNGVLNSWTDQDSKDSTYSMSSTLTLTK	iTRAQ4plex@N-term; iTRAQ4plex(K)@14; iTRAQ4plex(K)@28		-3.0	3535.76	884.95	3538.75	885.69	4	88.9	69.2	56.2	43.8	0.8
			93	QNGVLNSWTDQDSKDSTYSMSSTLTLTK	iTRAQ4plex@N-term; Trp->Tyr@8; Deamidated(Q)@11; iTRAQ4plex(K)@14; Oxidation(M)@20; iTRAQ4plex(K)@28		0.1	3532.85	884.22	3532.71	884.18	4	73.1	102.3	41.7	58.3	1.4
			85	QNGVLNSWTDQDSK	iTRAQ4plex@N-term; Dioxidation(W)@8; Lys->Gln@14		0.0	1766.78	884.40	1766.78	884.40	2	767.5	578.9	57.0	43.0	0.8
			83	WKIDGSER	iTRAQ4plex@N-term; FormaldehydeAdduct(W)@1; iTRAQ4plex(K)@2	missed K-I@2	-0.1	1289.64	645.83	1289.70	645.86	2	1311.9	904.5	59.2	40.8	0.7
			83	DSTYSMSSTLTLTK	iTRAQ4plex@N-term; iTRAQ4plex(K)@14		0.0	1821.89	608.31	1821.93	608.32	3	16.9	38.3	30.6	69.4	2.3
			83	DSTYSMSSTLTLTKDEYER	iTRAQ4plex@N-term; Methyl(T)@13; iTRAQ4plex(K)@14; Oxidation(D)@15; Tyr->Gln@17		0.0	2509.21	628.31	2509.21	628.31	4	22.9	24.1	48.7	51.3	1.1
			76	DSTYSMSSTLTLTK	iTRAQ4plex@N-term; Oxidation(M)@6; iTRAQ4plex(K)@14		0.0	1837.91	613.64	1837.92	613.65	3	50.6	67.3	42.9	57.1	1.3
			70	QNGVLNSWTDQDSKDSTYSMSSTLTLTK	iTRAQ4plex@N-term; iTRAQ4plex(K)@14; Ser->Pro@16; iTRAQ4plex(K)@28		0.0	3548.80	888.21	3548.77	888.20	4	39.0	35.4	52.4	47.6	0.9
			60	SSQSLLHSNGK	iTRAQ4plex@N-term; His->Asp@7; iTRAQ4plex(K)@11		0.0	1422.72	475.25	1422.76	475.26	3	1862.2	1567.6	54.3	45.7	0.8
			56	LIYLVSK	iTRAQ4plex@N-term; Lys->Arg@7	cleaved L-L@N-term	0.0	1006.66	504.34	1006.63	504.32	2	1525.7	3313.9	31.5	68.5	2.2
			51	TSTSPIVK	iTRAQ4plex@N-term; Lys->Gln@8		0.0	975.59	488.80	975.54	488.78	2	521.4	304.3	63.1	36.9	0.6
			49	LIYLVSK	iTRAQ4plex@N-term; Lys->Gln@7	cleaved L-L@N-term	0.0	978.58	490.30	978.59	490.30	2					
			49	LIYLVSK	iTRAQ4plex@N-term; Lys->Gln@7	cleaved L-L@N-term	0.0	978.62	490.32	978.59	490.30	2					
			45	TSTSPIVK	iTRAQ4plex@N-term; Lys->Gln@8		0.1	975.61	488.81	975.54	488.78	2					
			41	IDGSER	iTRAQ4plex@N-term		0.0	819.44	410.73	819.42	410.72	2					
			41	VEAEDLGVYYCLQATHFPRTFGGGTK	iTRAQ4plex@N-term; Pyridylethyl(C)@11; Ala->Thr@14; Deamidated(R)@19; Lys->Arg@26	missed R-T@19	-0.1	3166.46	792.62	3166.53	792.64	4					
			38	DSTYSMSSTLTLTKDEYER	iTRAQ4plex@N-term; Thr->Pro@11; iTRAQ4plex(K)@14; Glu->Gln@18		0.0	2509.21	628.31	2509.23	628.31	4					
			37	DSTYSMSSTLTLTK	iTRAQ4plex@N-term; iTRAQ4plex(K)@14		0.0	1821.91	608.31	1821.93	608.32	3					
			36	TFGGGTK	iTRAQ4plex@N-term; Lys->Gln@7		0.1	810.48	406.25	810.40	406.21	2					
			32	IDGSER	iTRAQ4plex@N-term		0.0	819.43	410.72	819.42	410.72	2					
			30	IDGSER	iTRAQ4plex@N-term		0.0	819.43	410.72	819.42	410.72	2					
			28	IDGSER	iTRAQ4plex@N-term		0.0	819.44	410.73	819.42	410.72	2					
			28	QNGVLNSWTDQDSKDSTYSMSSTLTL	iTRAQ4plex@N-term; iTRAQ4plex(K)@14; Phospho(S)@16; iTRAQ4plex(Y)@18	cleaved L-T@C-term	0.1	3389.66	848.42	3389.57	848.40	4					
			25	DSTYSMSSTLTLTK	iTRAQ4plex@N-term; Dehydrated(T)@11; iTRAQ4plex(K)@14		0.0	1803.93	602.32	1803.92	602.31	3					
			24	IDGSER	iTRAQ4plex@N-term		0.0	819.43	410.72	819.42	410.72	2					
			24	QNGVLNSWTDQDSKDSTYSMSSTLTLTK	iTRAQ4plex@N-term; Deamidated(Q)@11; Lys->Arg@14; Dehydrated(T)@23; iTRAQ4plex(K)@28		0.1	3405.70	852.43	3405.62	852.41	4					
			23	QNGVLNSWTDQDSK	iTRAQ4plex@N-term; Deamidated(N)@2; Dioxidation(W)@8; Lys->Gln@14		0.1	1767.82	884.92	1767.77	884.89	2					
			18	QNGVLNSWTDQDSKDSTYSMSSTLTLTK	iTRAQ4plex@N-term; Deamidated(N)@2; Trp->Tyr@8; iTRAQ4plex(K)@14; Leu->Met@24; iTRAQ4plex(K)@28		-0.1	3534.62	707.93	3534.67	707.94	5					
			18	QNGVLNSWTDQDSK	iTRAQ4plex@N-term; Deamidated(N)@6; Oxidation(W)@8; Lys->Gln@14		0.0	1751.79	876.90	1751.77	876.89	2					
			15	LIYLVSK	iTRAQ4plex@N-term; Ser->Asp@6; iTRAQ4plex(K)@7	cleaved L-L@N-term	0.0	1150.73	576.37	1150.72	576.37	2					
			12	DSTYSMSSTLTLTK	iTRAQ4plex@N-term; iTRAQ4plex(K)@14		0.0	1821.90	608.31	1821.93	608.32	3					
			12	LLIYLVSKLESGVPDR	iTRAQ4plex@N-term; Lys->Arg@8; Glu->Asp@10	missed K-L@8	0.0	1959.14	654.06	1959.13	654.05	3					
			11	LIYLVSK	iTRAQ4plex@N-term; Ser->Asp@6; iTRAQ4plex(K)@7	cleaved L-L@N-term	0.0	1150.71	576.36	1150.72	576.37	2					
			10	ESGVPDR	iTRAQ4plex@N-term	cleaved L-E@N-term	0.0	902.49	452.25	902.46	452.24	2					
			10	FSGSGSGTDFTLK	iTRAQ4plex@N-term; Methyl(S)@2; Dehydrated(D)@9; iTRAQ4plex(K)@13		0.0	1586.79	529.94	1586.82	529.95	3					
			10	FSGSGSGTDFTLK	iTRAQ4plex@N-term; Methyl(S)@2; Lys->Gln@13		0.0	1460.68	731.35	1460.69	731.35	2					
			10	FSGSGSGTDFTLK	iTRAQ4plex@N-term; Methyl(S)@2; Lys->Gln@13		0.0	1460.69	731.35	1460.69	731.35	2					
			10	FSGSGSGTDFTLK	iTRAQ4plex@N-term; Methyl(S)@2; Lys->Gln@13		0.0	1460.68	731.35	1460.69	731.35	2					
			10	FSGSGSGTDFTLK	iTRAQ4plex@N-term; Methyl(S)@2; Lys->Gln@13		0.0	1460.68	731.35	1460.69	731.35	2					
			10	FSGSGSGTDFTLKISR	iTRAQ4plex@N-term; Methyl(S)@2; Thr->Pro@11; iTRAQ4plex(K)@13	missed K-I@13	0.0	1957.07	653.36	1957.05	653.36	3					
			10	DSTYSMSSTLTLTK	iTRAQ4plex@N-term; Oxidation(M)@6; iTRAQ4plex(K)@14		0.0	1837.94	613.65	1837.92	613.65	3					
																Average	1.1
																Stdev	0.6
IPI:IPI00828887.1	IGH-V7183	Immunoglobulin heavy chain (V7183 family)	99	DNAKNTLYLQMSR	iTRAQ4plex@N-term; iTRAQ4plex(K)@4; Thr->Pro@6	missed K-N@4	0.0	1836.95	613.32	1836.98	613.33	3	2375.1	1405.7	62.8	37.2	0.6
			99	NTLYLQMSR	iTRAQ4plex@N-term		0.0	1268.66	635.34	1268.67	635.34	2	16297.9	9386.4	63.5	36.5	0.6
			99	NTLYLQMSR	iTRAQ4plex@N-term		0.0	1268.67	635.34	1268.67	635.34	2	5131.7	2885.8	64.0	36.0	0.6
			99	NTLYLQMSR	iTRAQ4plex@N-term		0.0	1268.68	635.35	1268.67	635.34	2	4376.2	2427.8	64.3	35.7	0.6
			99	LVESGGGLVQPGGSLK	iTRAQ4plex@N-term; iTRAQ4plex(K)@16	cleaved Q-L@N-term	0.0	1784.98	596.00	1785.02	596.02	3	373.7	438.2	46.0	54.0	1.2
			99	DNAKNTLYLQMSR	iTRAQ4plex@N-term; iTRAQ4plex(K)@4; Asn->Glu@5; Thr->Pro@6	missed K-N@4	0.0	1851.96	618.33	1851.98	618.33	3	1451.8	981.0	59.7	40.3	0.7
			99	DNAKNTLYLQMSR	iTRAQ4plex@N-term; iTRAQ4plex(K)@4; Thr->Pro@6; Ser->Asp@12	missed K-N@4	0.0	1865.01	622.68	1864.97	622.66	3	184.3	364.1	33.6	66.4	2.0
			99	FSGSGSGTDFTLKISR	iTRAQ4plex@N-term; Phe->Met@10; Thr->Gln@11; iTRAQ4plex(K)@13	missed K-I@13	0.0	1958.03	653.69	1958.01	653.68	3	409.7	305.2	57.3	42.7	0.7
			99	VEAEDLGVYYCFQASHVPWTFGGGTK	iTRAQ4plex@N-term; Pyridylethyl(C)@11; Deamidated(Q)@13; Ala->Val@14; Lys->Gln@26		0.0	3138.50	785.63	3138.46	785.62	4	47.3	57.9	44.9	55.1	1.2
			99	SLPVSLGDQASISCR	iTRAQ4plex@N-term; Pyridylethyl(C)@14	cleaved L-S@N-term	0.0	1780.93	594.65	1780.93	594.65	3	84.9	128.8	39.7	60.3	1.5
			99	SEDTAMYYCAAREGL	iTRAQ4plex@N-term; Pyridylethyl(C)@9; Ala->Tyr@11; Deamidated(R)@12	cleaved L-F@C-term; missed R-E@12	-0.1	2020.81	674.61	2020.87	674.63	3	549.6	382.2	59.0	41.0	0.7
			99	FSGSGSGTDFTLKISR	iTRAQ4plex@N-term; Thr->Pro@11; iTRAQ4plex(K)@13	missed K-I@13	0.0	1943.01	648.68	1943.04	648.69	3	472.5	340.5	58.1	41.9	0.7
			98	NTLYLQMSR	iTRAQ4plex@N-term		0.0	1268.67	635.34	1268.67	635.34	2	770.8	439.8	63.7	36.3	0.6
			98	FSGSGSGTDFTLK	iTRAQ4plex@N-term; iTRAQ4plex(K)@13		0.0	1590.80	531.27	1590.81	531.28	3	396.1	428.1	48.1	51.9	1.1
			97	FSGSGSGTDFTLK	iTRAQ4plex@N-term; Lys->Gln@13		0.0	1446.67	724.34	1446.67	724.34	2	2854.7	1548.6	64.8	35.2	0.5
			97	FSGSGSGTDFTLK	iTRAQ4plex@N-term; Lys->Gln@13		0.0	1446.71	724.36	1446.67	724.34	2	1436.4	952.7	60.1	39.9	0.7
			97	FSGSGSGTDFTLK	iTRAQ4plex@N-term; Lys->Gln@13		0.0	1446.67	724.34	1446.67	724.34	2	1337.4	808.0	62.3	37.7	0.6
			97	FSGSGSGTDFTLK	iTRAQ4plex@N-term; Lys->Gln@13		0.0	1446.71	724.36	1446.67	724.34	2	590.9	403.7	59.4	40.6	0.7
			96	FSGVPDR	iTRAQ4plex@N-term		0.0	920.48	461.25	920.48	461.25	2	19766.8	15804.2	55.6	44.4	0.8
			94	NTLYLQMSR	iTRAQ4plex@N-term		0.0	1268.67	635.34	1268.67	635.34	2	6233.5	3628.7	63.2	36.8	0.6
			93	NTLYLQMSR	iTRAQ4plex@N-term		3.0	1271.67	636.84	1268.67	635.34	2	3980.2	2446.9	61.9	38.1	0.6
			93	NTLYLQMSR	iTRAQ4plex@N-term		0.0	1268.68	635.34	1268.67	635.34	2	604.8	375.4	61.7	38.3	0.6
			80	NTLYLQMSR	iTRAQ4plex@N-term; Met->Val@7		-0.1	1236.64	619.33	1236.69	619.35	2	450.2	248.1	64.5	35.5	0.6
			56	TYYPDTVK	iTRAQ4plex@N-term; Oxidation(Y)@3; iTRAQ4plex(K)@8	cleaved S-T@N-term	0.0	1289.71	645.86	1289.67	645.84	2	994.8	918.0	52.0	48.0	0.9
			49	NTLYLQMSR	iTRAQ4plex@N-term; Oxidation(M)@7		0.0	1284.64	643.33	1284.66	643.34	2					
			42	FSGVPDR	iTRAQ4plex@N-term; Oxidation(F)@1		0.0	936.50	469.26	936.48	469.25	2					
			31	LEWVAYISIGGGSTYY	iTRAQ4plex@N-term; Oxidation(Y)@15; Tyr->Arg@16	cleaved Y-P@C-term	0.0	1930.95	644.66	1930.99	644.67	3					
			25	TFGGGTK	iTRAQ4plex@N-term; Lys->Gln@7	cleaved W-T@N-term	0.1	810.48	406.25	810.40	406.21	2					
			24	SEDTAMYYCAAREGL	iTRAQ4plex@N-term; Pyridylethyl(C)@9; Ala->Tyr@11; Deamidated(R)@12	cleaved L-F@C-term; missed R-E@12	0.0	2020.84	674.62	2020.87	674.63	3					
			17	TYYPDTVK	iTRAQ4plex@N-term; iTRAQ4plex(K)@8	cleaved S-T@N-term	-2.0	1271.65	636.83	1273.68	637.85	2					
			16	VEAEDLGVYYCFQASHVPWTFGGGTK	iTRAQ4plex@N-term; Pyridylethyl(C)@11; Deamidated(Q)@13; Lys->Arg@26		0.0	3138.50	785.63	3138.47	785.62	4					
			11	DNAKNTLYLQMSR	iTRAQ4plex@N-term; Dehydrated(D)@1; Deamidated(N)@2; Lys->Arg@4	missed K-N@4	0.0	1707.83	570.28	1707.85	570.29	3					
			10	FSGSGSGTDFTLK	iTRAQ4plex@N-term; Methyl(S)@2; Dehydrated(D)@9; iTRAQ4plex(K)@13		0.0	1586.79	529.94	1586.82	529.95	3					
			10	FSGSGSGTDFTLK	iTRAQ4plex@N-term; Methyl(S)@2; Lys->Gln@13		0.0	1460.68	731.35	1460.69	731.35	2					
			10	FSGSGSGTDFTLK	iTRAQ4plex@N-term; Methyl(S)@2; Lys->Gln@13		0.0	1460.69	731.35	1460.69	731.35	2					
			10	FSGSGSGTDFTLK	iTRAQ4plex@N-term; Methyl(S)@2; Lys->Gln@13		0.0	1460.68	731.35	1460.69	731.35	2					
			10	FSGSGSGTDFTLK	iTRAQ4plex@N-term; Methyl(S)@2; Lys->Gln@13		0.0	1460.68	731.35	1460.69	731.35	2					
			10	FSGSGSGTDFTLKISR	iTRAQ4plex@N-term; Methyl(S)@2; Thr->Pro@11; iTRAQ4plex(K)@13	missed K-I@13	0.0	1957.07	653.36	1957.05	653.36	3					
																Average	0.8
																Stdev	0.4
IPI:IPI00853676.1	IGKC	Immunoglobulin kappa constant	99	LIYIVSNRFSGVPDR	iTRAQ4plex@N-term; Asn->Cys@7; Pyridylethyl(C)@7	cleaved L-L@N-term; missed R-F@8	0.1	1973.12	658.72	1973.07	658.70	3	479.4	450.1	51.6	48.4	0.9
			99	QNGVLNSWTDQDSK	iTRAQ4plex@N-term; Asp->Pro@10; iTRAQ4plex(K)@14		-0.1	1860.90	621.31	1860.96	621.33	3	424.4	454.7	48.3	51.7	1.1
			99	QNGVLNSWTDQDSK	iTRAQ4plex@N-term; Deamidated(N)@2; iTRAQ4plex(K)@14		-0.1	1879.83	627.62	1879.92	627.65	3	1523.3	1579.8	49.1	50.9	1.0
			99	QNGVLNSWTDQDSK	iTRAQ4plex@N-term; Deamidated(N)@2; iTRAQ4plex(K)@14		0.0	1879.93	627.65	1879.92	627.65	3	380.1	464.9	45.0	55.0	1.2
			99	QNGVLNSWTDQDSK	iTRAQ4plex@N-term; Deamidated(N)@2; iTRAQ4plex(K)@14		0.0	1879.91	627.64	1879.92	627.65	3	287.4	398.5	41.9	58.1	1.4
			99	QNGVLNSWTDQDSK	iTRAQ4plex@N-term; Deamidated(N)@2; Lys->Arg@14		0.0	1763.82	882.92	1763.82	882.92	2	511.7	1169.6	30.4	69.6	2.3
			99	QNGVLNSWTDQDSK	iTRAQ4plex@N-term; Deamidated(N)@2; Lys->Arg@14		0.0	1763.82	588.95	1763.82	588.95	3	93.2	203.2	31.4	68.6	2.2
			99	QNGVLNSWTDQDSK	iTRAQ4plex@N-term; Deamidated(N)@2; Lys->Gln@14		0.0	1735.73	868.87	1735.78	868.90	2	4267.8	3740.3	53.3	46.7	0.9
			99	QNGVLNSWTDQDSK	iTRAQ4plex@N-term; Deamidated(N)@2; Lys->Gln@14		0.0	1735.81	868.91	1735.78	868.90	2	1381.3	1309.2	51.3	48.7	0.9
			99	QNGVLNSWTDQDSK	iTRAQ4plex@N-term; Deamidated(N)@2; Ser->Asp@13; iTRAQ4plex(K)@14		0.0	1907.95	636.99	1907.91	636.98	3	144.1	413.6	25.8	74.2	2.9
			99	QNGVLNSWTDQDSK	iTRAQ4plex@N-term; Dehydrated(S)@7; Lys->Gln@14		0.0	1716.79	573.27	1716.78	573.27	3	138.7	109.7	55.8	44.2	0.8
			99	DSTYSMSSTLTLTKDEYER	iTRAQ4plex@N-term; Dehydrated(S)@8; Thr->Asn@13; iTRAQ4plex(K)@14; Oxidation(D)@15		0.0	2525.16	842.73	2525.18	842.74	3	84.9	80.0	51.5	48.5	0.9
			99	DSTYSMSSTLTLTKDEYER	iTRAQ4plex@N-term; Dehydrated(T)@11; Thr->Asn@13; iTRAQ4plex(K)@14; Oxidation(D)@15		0.0	2525.14	842.72	2525.18	842.74	3	105.8	107.6	49.6	50.4	1.0
			99	QNGVLNSWTDQDSK	iTRAQ4plex@N-term; Dehydrated(T)@9; Lys->Gln@14		0.1	1716.83	573.29	1716.78	573.27	3	100.5	103.5	49.3	50.7	1.0
			99	DSTYSMSSTLTLTKDEYER	iTRAQ4plex@N-term; Dehydrated(T)@9; Thr->Asn@13; iTRAQ4plex(K)@14; Oxidation(D)@15		0.0	2525.16	842.73	2525.18	842.74	3	241.8	193.6	55.5	44.5	0.8
			99	DSTYSMSSTLTLTKDEYER	iTRAQ4plex@N-term; iTRAQ4plex(K)@14		0.0	2514.22	839.08	2514.20	839.08	3	2539.6	610.5	80.6	19.4	0.2
			99	QNGVLNSWTDQDSK	iTRAQ4plex@N-term; iTRAQ4plex(K)@14		-0.1	1878.87	627.30	1878.93	627.32	3	1865.9	2466.6	43.1	56.9	1.3
			99	DSTYSMSSTLTLTKDEYER	iTRAQ4plex@N-term; iTRAQ4plex(K)@14		-0.1	2514.12	839.05	2514.20	839.08	3	1682.4	431.3	79.6	20.4	0.3
			99	DSTYSMSSTLTLTKDEYER	iTRAQ4plex@N-term; iTRAQ4plex(K)@14		0.0	2514.20	839.07	2514.20	839.08	3	683.2	182.2	78.9	21.1	0.3
			99	DSTYSMSSTLTLTK	iTRAQ4plex@N-term; iTRAQ4plex(K)@14		-0.1	1821.88	608.30	1821.93	608.32	3	423.1	488.4	46.4	53.6	1.2
			99	DSTYSMSSTLTLTK	iTRAQ4plex@N-term; iTRAQ4plex(K)@14		0.0	1821.92	608.31	1821.93	608.32	3	355.6	384.8	48.0	52.0	1.1
			99	QNGVLNSWTDQDSK	iTRAQ4plex@N-term; iTRAQ4plex(K)@14		0.0	1878.94	627.32	1878.93	627.32	3	273.4	280.6	49.4	50.6	1.0
			99	DSTYSMSSTLTLTK	iTRAQ4plex@N-term; iTRAQ4plex(K)@14		0.0	1821.88	608.30	1821.93	608.32	3	196.9	320.6	38.0	62.0	1.6
			99	DSTYSMSSTLTLTK	iTRAQ4plex@N-term; iTRAQ4plex(K)@14		0.0	1821.89	608.30	1821.93	608.32	3	194.2	242.8	44.4	55.6	1.2
			99	DSTYSMSSTLTLTK	iTRAQ4plex@N-term; iTRAQ4plex(K)@14		0.0	1821.89	608.31	1821.93	608.32	3	150.1	200.1	42.9	57.1	1.3
			99	DSTYSMSSTLTLTK	iTRAQ4plex@N-term; iTRAQ4plex(K)@14		0.0	1821.88	608.30	1821.93	608.32	3	109.3	163.8	40.0	60.0	1.5
			99	DSTYSMSSTLTLTK	iTRAQ4plex@N-term; iTRAQ4plex(K)@14		0.0	1821.88	608.30	1821.93	608.32	3	97.1	96.0	50.3	49.7	1.0
			99	DSTYSMSSTLTLTKDEYER	iTRAQ4plex@N-term; iTRAQ4plex(K)@14; Dimethyl(R)@19		0.0	2542.20	848.41	2542.24	848.42	3	418.2	371.1	53.0	47.0	0.9
			99	DSTYSMSSTLTLTKDEYER	iTRAQ4plex@N-term; iTRAQ4plex(K)@14; Dimethyl(R)@19		0.0	2542.19	848.40	2542.24	848.42	3	198.1	187.6	51.4	48.6	0.9
			99	QNGVLNSWTDQDSKDSTYSMSSTLTLTK	iTRAQ4plex@N-term; iTRAQ4plex(K)@14; iTRAQ4plex(K)@28		0.0	3538.72	885.69	3538.75	885.69	4	102.9	38.4	72.8	27.2	0.4
			99	QNGVLNSWTDQDSKDSTYSMSSTLTLTK	iTRAQ4plex@N-term; iTRAQ4plex(K)@14; Lys->Gln@28		0.0	3394.61	849.66	3394.61	849.66	4	99.7	26.7	78.9	21.1	0.3
			99	QNGVLNSWTDQDSK	iTRAQ4plex@N-term; Lys->Arg@14		0.0	1762.85	882.43	1762.84	882.43	2	507.5	1182.6	30.0	70.0	2.3
			99	QNGVLNSWTDQDSK	iTRAQ4plex@N-term; Lys->Arg@14		0.0	1762.83	588.62	1762.84	588.62	3	299.2	575.9	34.2	65.8	1.9
			99	QNGVLNSWTDQDSK	iTRAQ4plex@N-term; Lys->Gln@14		0.0	1734.76	868.39	1734.79	868.40	2	7731.8	5736.1	57.4	42.6	0.7
			99	QNGVLNSWTDQDSK	iTRAQ4plex@N-term; Lys->Gln@14		0.0	1734.78	868.40	1734.79	868.40	2	928.9	720.3	56.3	43.7	0.8
			99	DSTYSMSSTLTLTK	iTRAQ4plex@N-term; Lys->Gln@14		0.0	1677.79	839.90	1677.79	839.90	2	917.9	968.9	48.6	51.4	1.1
			99	QNGVLNSWTDQDSK	iTRAQ4plex@N-term; Lys->Gln@14		0.0	1734.84	579.29	1734.79	579.27	3	632.7	813.5	43.7	56.3	1.3
			99	DSTYSMSSTLTLTKDEYER	iTRAQ4plex@N-term; Lys->Gln@14		0.0	2370.06	791.03	2370.07	791.03	3	464.1	117.8	79.8	20.2	0.3
			99	DSTYSMSSTLTLTKDEYER	iTRAQ4plex@N-term; Lys->Gln@14		0.0	2370.06	791.03	2370.07	791.03	3	312.1	72.7	81.1	18.9	0.2
			99	DSTYSMSSTLTLTK	iTRAQ4plex@N-term; Lys->Gln@14		0.1	1677.85	560.29	1677.79	560.27	3	6.6	20.4	24.4	75.6	3.1
			99	DSTYSMSSTLTLTKDEYER	iTRAQ4plex@N-term; Lys->Gln@14; Dimethyl(R)@19		0.0	2398.09	800.37	2398.10	800.37	3	186.5	152.4	55.0	45.0	0.8
			99	DSTYSMSSTLTLTKDEYER	iTRAQ4plex@N-term; Lys->Gln@14; Glu->Arg@18; Deamidated(R)@19		0.0	2398.07	800.37	2398.11	800.38	3	125.1	81.0	60.7	39.3	0.6
			99	DSTYSMSSTLTLTK	iTRAQ4plex@N-term; Oxidation(M)@6; Lys->Gln@14		0.0	1693.80	847.91	1693.78	847.90	2	87.3	113.7	43.4	56.6	1.3
			99	QNGVLNSWTDQDSK	iTRAQ4plex@N-term; Oxidation(W)@8; Lys->Gln@14		0.0	1750.83	876.42	1750.79	876.40	2	235.7	161.3	59.4	40.6	0.7
			99	FSGSGSGTDFTLKISR	iTRAQ4plex@N-term; Phe->Met@10; Thr->Gln@11; iTRAQ4plex(K)@13	missed K-I@13	0.0	1958.03	653.69	1958.01	653.68	3	409.7	305.2	57.3	42.7	0.7
			99	QNGVLNSWTDQDSK	iTRAQ4plex@N-term; Phospho(S)@7; Oxidation(W)@8; Asp->Gly@10; iTRAQ4plex(K)@14		0.0	1916.85	480.22	1916.89	480.23	4	97.8	78.7	55.4	44.6	0.8
			99	QNGVLNSWTDQDSK	iTRAQ4plex@N-term; Ser->Asp@13; iTRAQ4plex(K)@14		0.0	1906.93	636.65	1906.93	636.65	3	86.8	177.6	32.8	67.2	2.0
			99	DSTYSMSSTLTLTKDEYER	iTRAQ4plex@N-term; Ser->Gln@8; Thr->Ala@13; iTRAQ4plex(K)@14		0.0	2525.17	842.73	2525.22	842.75	3	68.8	68.9	50.0	50.0	1.0
			99	DSTYSMSSTLTLTKDEYER	iTRAQ4plex@N-term; Thr->Asn@13; iTRAQ4plex(K)@14; Dehydrated(E)@18		-0.1	2509.13	628.29	2509.19	628.30	4	1118.0	1202.6	48.2	51.8	1.1
			99	FSGSGSGTDFTLKISR	iTRAQ4plex@N-term; Thr->Pro@11; iTRAQ4plex(K)@13	missed K-I@13	0.0	1943.01	648.68	1943.04	648.69	3	472.5	340.5	58.1	41.9	0.7
			99	DSTYSMSSTLTLTKDEYER	iTRAQ4plex@N-term; Thr->Pro@13; iTRAQ4plex(K)@14		0.0	2510.19	837.74	2510.21	837.74	3	270.7	203.2	57.1	42.9	0.8
			99	DSTYSMSSTLTLTKDEYER	iTRAQ4plex@N-term; Thr->Pro@13; iTRAQ4plex(K)@14		0.0	2510.19	837.74	2510.21	837.74	3	182.3	168.7	51.9	48.1	0.9
			98	FSGSGSGTDFTLK	iTRAQ4plex@N-term; iTRAQ4plex(K)@13		0.0	1590.80	531.27	1590.81	531.28	3	396.1	428.1	48.1	51.9	1.1
			98	QNGVLNSWTDQDSK	iTRAQ4plex@N-term; Ser->Oxoalanine(S)@7; Oxidation(W)@8; iTRAQ4plex(K)@14		0.0	1892.90	631.97	1892.91	631.98	3	192.6	238.5	44.7	55.3	1.2
			98	DSTYSMSSTLTLTKDEYER	iTRAQ4plex@N-term; Thr->Asn@13; iTRAQ4plex(K)@14; Dehydrated(E)@18		0.0	2509.19	628.31	2509.19	628.30	4	107.4	115.5	48.2	51.8	1.1
			98	TSTSPIVKSFNR	iTRAQ4plex@N-term; Val->Leu@7; iTRAQ4plex(K)@8; Dehydrated(S)@9	missed K-S@8	0.0	1619.88	540.97	1619.92	540.98	3	2180.6	1417.5	60.6	39.4	0.7
			97	QNGVLNSWTDQDSK	iTRAQ4plex@N-term; Dioxidation(W)@8; Lys->Gln@14		0.0	1766.79	884.40	1766.78	884.40	2	807.3	640.6	55.8	44.2	0.8
			97	DSTYSMSSTLTLTK	iTRAQ4plex@N-term; iTRAQ4plex(K)@14		0.0	1821.89	608.30	1821.93	608.32	3	29.8	86.6	25.6	74.4	2.9
			97	TSTSPIVK	iTRAQ4plex@N-term; Lys->Arg@8		0.0	1003.58	502.80	1003.58	502.80	2	6406.1	14164.1	31.1	68.9	2.2
			97	FSGSGSGTDFTLK	iTRAQ4plex@N-term; Lys->Gln@13		0.0	1446.67	724.34	1446.67	724.34	2	2854.7	1548.6	64.8	35.2	0.5
			97	FSGSGSGTDFTLK	iTRAQ4plex@N-term; Lys->Gln@13		0.0	1446.71	724.36	1446.67	724.34	2	1436.4	952.7	60.1	39.9	0.7
			97	FSGSGSGTDFTLK	iTRAQ4plex@N-term; Lys->Gln@13		0.0	1446.67	724.34	1446.67	724.34	2	1337.4	808.0	62.3	37.7	0.6
			97	FSGSGSGTDFTLK	iTRAQ4plex@N-term; Lys->Gln@13		0.0	1446.71	724.36	1446.67	724.34	2	590.9	403.7	59.4	40.6	0.7
			96	FSGVPDR	iTRAQ4plex@N-term		0.0	920.48	461.25	920.48	461.25	2	19766.8	15804.2	55.6	44.4	0.8
			96	TSTSPIVK	iTRAQ4plex@N-term; iTRAQ4plex(K)@8		0.0	1119.63	560.82	1119.67	560.84	2	47126.8	27132.6	63.5	36.5	0.6
			95	TSTSPIVK	iTRAQ4plex@N-term; iTRAQ4plex(K)@8		0.0	1119.66	560.84	1119.67	560.84	2	19345.5	15414.0	55.7	44.3	0.8
			95	LLIYIVSNRFSGVPDR	iTRAQ4plex@N-term; Phe->Leu@10	missed R-F@9	0.0	1958.16	653.73	1958.14	653.72	3	485.2	393.4	55.2	44.8	0.8
			94	LLIYIVSNRFSGVPDR	iTRAQ4plex@N-term; Deamidated(R)@9; Phe->Leu@10	missed R-F@9	0.0	1959.14	654.06	1959.13	654.05	3	26.0	40.3	39.2	60.8	1.6
			94	QNGVLNSWTDQDSKDSTYSMSSTLTLTK	iTRAQ4plex@N-term; iTRAQ4plex(K)@14; iTRAQ4plex(K)@28		-3.0	3535.76	884.95	3538.75	885.69	4	88.9	69.2	56.2	43.8	0.8
			93	QNGVLNSWTDQDSKDSTYSMSSTLTLTK	iTRAQ4plex@N-term; Trp->Tyr@8; Deamidated(Q)@11; iTRAQ4plex(K)@14; Oxidation(M)@20; iTRAQ4plex(K)@28		0.1	3532.85	884.22	3532.71	884.18	4	73.1	102.3	41.7	58.3	1.4
			85	QNGVLNSWTDQDSK	iTRAQ4plex@N-term; Dioxidation(W)@8; Lys->Gln@14		0.0	1766.78	884.40	1766.78	884.40	2	767.5	578.9	57.0	43.0	0.8
			83	WKIDGSER	iTRAQ4plex@N-term; FormaldehydeAdduct(W)@1; iTRAQ4plex(K)@2	missed K-I@2	-0.1	1289.64	645.83	1289.70	645.86	2	1311.9	904.5	59.2	40.8	0.7
			83	DSTYSMSSTLTLTK	iTRAQ4plex@N-term; iTRAQ4plex(K)@14		0.0	1821.89	608.31	1821.93	608.32	3	16.9	38.3	30.6	69.4	2.3
			83	DSTYSMSSTLTLTKDEYER	iTRAQ4plex@N-term; Methyl(T)@13; iTRAQ4plex(K)@14; Oxidation(D)@15; Tyr->Gln@17		0.0	2509.21	628.31	2509.21	628.31	4	22.9	24.1	48.7	51.3	1.1
			76	DSTYSMSSTLTLTK	iTRAQ4plex@N-term; Oxidation(M)@6; iTRAQ4plex(K)@14		0.0	1837.91	613.64	1837.92	613.65	3	50.6	67.3	42.9	57.1	1.3
			70	QNGVLNSWTDQDSKDSTYSMSSTLTLTK	iTRAQ4plex@N-term; iTRAQ4plex(K)@14; Ser->Pro@16; iTRAQ4plex(K)@28		0.0	3548.80	888.21	3548.77	888.20	4	39.0	35.4	52.4	47.6	0.9
			51	TSTSPIVK	iTRAQ4plex@N-term; Lys->Gln@8		0.0	975.59	488.80	975.54	488.78	2	521.4	304.3	63.1	36.9	0.6
			45	TSTSPIVK	iTRAQ4plex@N-term; Lys->Gln@8		0.1	975.61	488.81	975.54	488.78	2					
			42	FSGVPDR	iTRAQ4plex@N-term; Oxidation(F)@1		0.0	936.50	469.26	936.48	469.25	2					
			41	IDGSER	iTRAQ4plex@N-term		0.0	819.44	410.73	819.42	410.72	2					
			40	VEAEDLGVYFCSQSTHVPGTFGGGTK	iTRAQ4plex@N-term; Pyridylethyl(C)@11; Ser->Asp@14; Oxidation(H)@16; Oxidation(P)@18; iTRAQ4plex(K)@26		0.0	3138.50	785.63	3138.48	785.63	4					
			38	DSTYSMSSTLTLTKDEYER	iTRAQ4plex@N-term; Thr->Pro@11; iTRAQ4plex(K)@14; Glu->Gln@18		0.0	2509.21	628.31	2509.23	628.31	4					
			37	DSTYSMSSTLTLTK	iTRAQ4plex@N-term; iTRAQ4plex(K)@14		0.0	1821.91	608.31	1821.93	608.32	3					
			32	IDGSER	iTRAQ4plex@N-term		0.0	819.43	410.72	819.42	410.72	2					
			30	IDGSER	iTRAQ4plex@N-term		0.0	819.43	410.72	819.42	410.72	2					

			28	IDGSER	iTRAQ4plex@N-term		0.0	819.44	410.73	819.42	410.72	2				
			28	QNGVLNSWTDQDSKDSTYSMSSTLTL	iTRAQ4plex@N-term; iTRAQ4plex(K)@14; Phospho(S)@16; iTRAQ4plex(Y)@18	cleaved L-T@C-term	0.1	3389.66	848.42	3389.57	848.40	4				
			25	TFGGGTK	iTRAQ4plex@N-term; Lys->Gln@7	cleaved G-T@N-term	0.1	810.48	406.25	810.40	406.21	2				
			25	DSTYSMSSTLTLTK	iTRAQ4plex@N-term; Dehydrated(T)@11; iTRAQ4plex(K)@14		0.0	1803.93	602.32	1803.92	602.31	3				
			24	IDGSER	iTRAQ4plex@N-term		0.0	819.43	410.72	819.42	410.72	2				
			24	QNGVLNSWTDQDSKDSTYSMSSTLTLTK	iTRAQ4plex@N-term; Deamidated(Q)@11; Lys->Arg@14; Dehydrated(T)@23; iTRAQ4plex(K)@28		0.1	3405.70	852.43	3405.62	852.41	4				
			23	QNGVLNSWTDQDSK	iTRAQ4plex@N-term; Deamidated(N)@2; Dioxidation(W)@8; Lys->Gln@14		0.1	1767.82	884.92	1767.77	884.89	2				
			18	QNGVLNSWTDQDSKDSTYSMSSTLTLTK	iTRAQ4plex@N-term; Deamidated(N)@2; Trp->Tyr@8; iTRAQ4plex(K)@14; Leu->Met@24; iTRAQ4plex(K)@28		-0.1	3534.62	707.93	3534.67	707.94	5				
			18	QNGVLNSWTDQDSK	iTRAQ4plex@N-term; Deamidated(N)@6; Oxidation(W)@8; Lys->Gln@14		0.0	1751.79	876.90	1751.77	876.89	2				
			12	DSTYSMSSTLTLTK	iTRAQ4plex@N-term; iTRAQ4plex(K)@14		0.0	1821.90	608.31	1821.93	608.32	3				
			10	FSGSGSGTDFTLK	iTRAQ4plex@N-term; Methyl(S)@2; Dehydrated(D)@9; iTRAQ4plex(K)@13		0.0	1586.79	529.94	1586.82	529.95	3				
			10	FSGSGSGTDFTLK	iTRAQ4plex@N-term; Methyl(S)@2; Lys->Gln@13		0.0	1460.68	731.35	1460.69	731.35	2				
			10	FSGSGSGTDFTLK	iTRAQ4plex@N-term; Methyl(S)@2; Lys->Gln@13		0.0	1460.69	731.35	1460.69	731.35	2				
			10	FSGSGSGTDFTLK	iTRAQ4plex@N-term; Methyl(S)@2; Lys->Gln@13		0.0	1460.69	731.35	1460.69	731.35	2				
			10	FSGSGSGTDFTLK	iTRAQ4plex@N-term; Methyl(S)@2; Lys->Gln@13		0.0	1460.68	731.35	1460.69	731.35	2				
			10	FSGSGSGTDFTLKISR	iTRAQ4plex@N-term; Methyl(S)@2; Thr->Pro@11; iTRAQ4plex(K)@13	missed K-I@13	0.0	1957.07	653.36	1957.05	653.36	3				
			10	DSTYSMSSTLTLTK	iTRAQ4plex@N-term; Oxidation(M)@6; iTRAQ4plex(K)@14		0.0	1837.94	613.65	1837.92	613.65	3				
															Average	1.1
															Stdev	0.6
IPI:IPI00115564.5	SLC25A4	Solute carrier family 25, member 4	99	EFNGLGDCLTK	iTRAQ4plex@N-term; Pyridylethyl(C)@8; Lys->Gln@11		0.0	1444.72	482.58	1444.68	482.57	3	215.4	263.7	45.0	55.0	1.2
			99	GAWSNVLR	iTRAQ4plex@N-term		0.0	1045.57	523.79	1045.58	523.80	2	1918.4	1932.2	49.8	50.2	1.0
			99	QIFLGGVDR	iTRAQ4plex@N-term		0.0	1147.62	574.82	1147.65	574.83	2	1834.3	2630.1	41.1	58.9	1.4
			99	EQGFLSFWR	iTRAQ4plex@N-term		0.0	1312.66	657.34	1312.67	657.34	2	556.4	647.5	46.2	53.8	1.2
			99	EQGFLSFWR	iTRAQ4plex@N-term		0.0	1312.68	657.35	1312.67	657.34	2	423.2	536.4	44.1	55.9	1.3
			99	EQGFLSFWR	iTRAQ4plex@N-term		0.0	1312.64	657.33	1312.67	657.34	2	304.6	326.4	48.3	51.7	1.1
			99	YFPTQALNFAFK	iTRAQ4plex@N-term; iTRAQ4plex(K)@12		0.0	1733.94	578.99	1733.94	578.99	3	87.0	123.5	41.3	58.7	1.4
			99	LLLQVQHASK	iTRAQ4plex@N-term; Lys->Gln@10		0.0	1279.75	427.59	1279.74	427.59	3	390.0	343.8	53.2	46.8	0.9
			99	YFPTQALNFAFK	iTRAQ4plex@N-term; Lys->Gln@12		0.1	1589.86	795.94	1589.80	795.91	2	334.4	361.4	48.1	51.9	1.1
			99	GADIMYTGTLDCWR	iTRAQ4plex@N-term; Pyridylethyl(C)@12		0.0	1849.84	617.62	1849.86	617.63	3	248.7	253.7	49.5	50.5	1.0
			97	MMMQSGR	iTRAQ4plex@N-term		0.0	983.45	492.73	983.45	492.73	2	4196.9	4578.0	47.8	52.2	1.1
			97	GNLANVIR	iTRAQ4plex@N-term		0.0	999.59	500.80	999.59	500.80	2	3043.6	3788.6	44.5	55.5	1.2
			94	YFPTQALNFAFK	iTRAQ4plex@N-term; Lys->Gln@12		0.1	1589.90	795.96	1589.80	795.91	2	54.3	66.4	45.0	55.0	1.2
			93	YFPTQALNFAFK	iTRAQ4plex@N-term; Lys->Gln@12		0.1	1589.88	795.95	1589.80	795.91	2	116.6	114.8	50.4	49.6	1.0
			92	TAVAPIER	iTRAQ4plex@N-term		0.0	999.59	500.80	999.58	500.80	2	14230.6	13072.5	52.1	47.9	0.9
			46	QISAEK	iTRAQ4plex@N-term; iTRAQ4plex(K)@6		0.0	962.60	482.31	962.56	482.29	2					
			11	YFPTQALNFAFK	iTRAQ4plex@N-term; Lys->Arg@12		0.0	1617.88	540.30	1617.84	540.29	3					
															Average	1.1
															Stdev	0.2
IPI:IPI00653675.1	C1S	Complement component 1, s subcomponen	99	TTLLENAQR	iTRAQ4plex@N-term		1.0	1189.68	595.85	1188.66	595.34	2	1566.0	1061.8	59.6	40.4	0.7
			99	WVNDQLGIELPR	iTRAQ4plex@N-term		0.0	1582.86	792.44	1582.86	792.44	2	467.5	325.8	58.9	41.1	0.7
			99	WVNDQLGIELPR	iTRAQ4plex@N-term		0.0	1582.85	792.43	1582.86	792.44	2	169.0	132.3	56.1	43.9	0.8
			99	ISDPLMYVGTMSVR	iTRAQ4plex@N-term		0.0	1711.92	856.97	1711.88	856.95	2	106.6	87.0	55.1	44.9	0.8
			99	TMQENSGPR	iTRAQ4plex@N-term		0.0	1162.59	582.30	1162.55	582.28	2	77.9	72.0	52.0	48.0	0.9
			99	WVNDQLGIELPR	iTRAQ4plex@N-term		0.0	1582.83	528.62	1582.86	528.63	3	51.7	37.1	58.2	41.8	0.7
			99	SDFSNEER	iTRAQ4plex@N-term	cleaved T-S@N-term	0.1	1126.58	564.30	1126.50	564.26	2	29.9	32.5	47.9	52.1	1.1
			99	ISDPLMYVGTMSVR	iTRAQ4plex@N-term		0.0	1711.91	571.64	1711.88	571.63	3	19.3	23.0	45.6	54.4	1.2
			99	TQSNTLGIVFQTDLMGQK	iTRAQ4plex@N-term; iTRAQ4plex(K)@18		0.0	2268.18	757.07	2268.20	757.07	3	12.5	8.2	60.4	39.6	0.7
			99	ASGALINEYWVLTAAHVLEK	iTRAQ4plex@N-term; iTRAQ4plex(K)@20		0.0	2472.41	825.14	2472.36	825.13	3	19.6	15.6	55.7	44.3	0.8
			99	EENPTVRPEDYVFTDNMICAGEK	iTRAQ4plex@N-term; Pyridylethyl(C)@19; iTRAQ4plex(K)@23		2.0	3051.39	763.86	3049.44	763.37	4	93.3	149.2	38.5	61.5	1.6
															Average	0.9
															Stdev	0.3
IPI:IPI00169625.5	LIGHT CHAIN	Anti-acid phosphatase variable light chain 18	99	FTGSGSGTDFTLKISR	iTRAQ4plex@N-term; Phe->Met@10; Thr->Gln@11; iTRAQ4plex(K)@13	missed K-I@13	0.0	1972.02	658.35	1972.03	658.35	3	2479.0	2464.6	50.1	49.9	1.0
			99	FTGSGSGTDFTLK	iTRAQ4plex@N-term; Cation:K(D)@9; iTRAQ4plex(K)@13		0.0	1642.77	411.70	1642.78	411.70	4	64.2	45.2	58.7	41.3	0.7
			99	FTGSGSGTDFTLK	iTRAQ4plex@N-term; Dehydrated(D)@9; iTRAQ4plex(K)@13		0.0	1586.79	529.94	1586.82	529.95	3	337.3	382.6	46.9	53.1	1.1
			99	FTGSGSGTDFTLK	iTRAQ4plex@N-term; iTRAQ4plex(K)@13		0.0	1604.78	535.93	1604.83	535.95	3	513.8	561.4	47.8	52.2	1.1
			99	FTGSGSGTDFTLK	iTRAQ4plex@N-term; iTRAQ4plex(K)@13		0.0	1604.82	535.95	1604.83	535.95	3	159.9	180.3	47.0	53.0	1.1
			99	LDSGVPDRFTGSGSGTDFTLK	iTRAQ4plex@N-term; iTRAQ4plex(K)@21	missed R-F@8	0.0	2444.21	815.75	2444.24	815.75	3	1181.2	171.4	87.3	12.7	0.1
			99	FTGSGSGTDFTLK	iTRAQ4plex@N-term; Lys->Gln@13		0.0	1460.68	731.35	1460.69	731.35	2	5813.4	4309.5	57.4	42.6	0.7
			99	FTGSGSGTDFTLK	iTRAQ4plex@N-term; Lys->Gln@13		0.0	1460.68	731.35	1460.69	731.35	2	4827.0	3760.0	56.2	43.8	0.8
			99	FTGSGSGTDFTLK	iTRAQ4plex@N-term; Lys->Gln@13		0.0	1460.68	731.35	1460.69	731.35	2	2882.5	2189.3	56.8	43.2	0.8
			99	FTGSGSGTDFTLK	iTRAQ4plex@N-term; Lys->Gln@13		0.0	1460.69	731.35	1460.69	731.35	2	2853.4	2185.6	56.6	43.4	0.8
			99	LDSGVPDRFTGSGSGTDFTLK	iTRAQ4plex@N-term; Lys->Gln@21	missed R-F@8	0.1	2300.16	767.73	2300.10	767.71	3	862.5	232.9	78.7	21.3	0.3
			99	LDSGVPDRFTGSGSGTDFTLK	iTRAQ4plex@N-term; Lys->Gln@21	missed R-F@8	0.0	2300.15	767.72	2300.10	767.71	3	846.9	160.9	84.0	16.0	0.2
			99	LDSGVPDRFTGSGSGTDFTLK	iTRAQ4plex@N-term; Lys->Gln@21	missed R-F@8	0.0	2300.12	767.71	2300.10	767.71	3	674.0	132.7	83.6	16.4	0.2
			99	LIYLVSKLDSGVPDR	iTRAQ4plex@N-term; Methyl(L)@4; Dehydrated(S)@6; iTRAQ4plex(K)@7	missed K-L@7	0.0	1958.14	653.72	1958.14	653.72	3	661.5	675.4	49.5	50.5	1.0
			99	FTGSGSGTDFTLKISR	iTRAQ4plex@N-term; Thr->Pro@11; iTRAQ4plex(K)@13	missed K-I@13	0.0	1957.07	653.36	1957.05	653.36	3	1813.2	1808.5	50.1	49.9	1.0
			98	SSQSLLDSDGK	iTRAQ4plex@N-term; Asp->Asn@9; iTRAQ4plex(K)@11		0.0	1422.72	475.25	1422.76	475.26	3	1862.2	1567.6	54.3	45.7	0.8
			98	TYLNWLLQR	iTRAQ4plex@N-term; Tyr->Phe@2	cleaved R-P@C-term	0.0	1333.77	667.89	1333.76	667.89	2	31.2	47.7	39.5	60.5	1.5
			97	TYLNWLLQR	iTRAQ4plex@N-term; Tyr->Phe@2	cleaved R-P@C-term	0.0	1333.76	667.88	1333.76	667.89	2	503.3	998.3	33.5	66.5	2.0
			95	LIYLVSK	iTRAQ4plex@N-term; iTRAQ4plex(K)@7		0.0	1122.69	562.35	1122.73	562.37	2	17362.1	14726.3	54.1	45.9	0.8
			74	LDSGVPDR	iTRAQ4plex@N-term		1.0	1002.50	502.26	1001.53	501.77	2	26210.8	22980.9	53.3	46.7	0.9
			68	FTGSGSGTDFTLK	iTRAQ4plex@N-term; Lys->Arg@13		0.0	1488.71	745.36	1488.73	745.37	2	238.2	490.4	32.7	67.3	2.1
			49	LIYLVSK	iTRAQ4plex@N-term; Lys->Gln@7		0.0	978.58	490.30	978.59	490.30	2					
			49	LIYLVSK	iTRAQ4plex@N-term; Lys->Gln@7		0.0	978.62	490.32	978.59	490.30	2					
			15	LIYLVSK	iTRAQ4plex@N-term; Ser->Asp@6; iTRAQ4plex(K)@7		0.0	1150.73	576.37	1150.72	576.37	2					
			15	FTGSGSGTDFTLK	iTRAQ4plex@N-term; Thr->Glu@11; iTRAQ4plex(K)@13		0.0	1632.83	545.28	1632.82	545.28	3					
			13	VEAEDLGVYYCWQGTHFP	iTRAQ4plex@N-term; Pyridylethyl(C)@11		-8.2	2353.88	785.63	2362.09	788.37	3					
			11	LIYLVSK	iTRAQ4plex@N-term; Ser->Asp@6; iTRAQ4plex(K)@7		0.0	1150.71	576.36	1150.72	576.37	2					
															Average	0.9
															Stdev	0.5
IPI:IPI00464378.3	CR2	Complement receptor 2	99	DVVMTQTPLSLPVSLGDQASISCR	iTRAQ4plex@N-term; Pyridylethyl(C)@23		0.0	2765.38	692.35	2765.42	692.36	4	12.4	11.5	51.9	48.1	0.9
			99	FSGSGSGTDFTLKISR	iTRAQ4plex@N-term; Phe->Met@10; Thr->Gln@11; iTRAQ4plex(K)@13	missed K-I@13	0.0	1958.03	653.69	1958.01	653.68	3	409.7	305.2	57.3	42.7	0.7
			99	SLPVSLGDQASISCR	iTRAQ4plex@N-term; Pyridylethyl(C)@14	cleaved L-S@N-term	0.0	1780.93	594.65	1780.93	594.65	3	84.9	128.8	39.7	60.3	1.5
			99	FSGSGSGTDFTLKISR	iTRAQ4plex@N-term; Thr->Pro@11; iTRAQ4plex(K)@13	missed K-I@13	0.0	1943.01	648.68	1943.04	648.69	3	472.5	340.5	58.1	41.9	0.7
			98	FSGSGSGTDFTLK	iTRAQ4plex@N-term; iTRAQ4plex(K)@13		0.0	1590.80	531.27	1590.81	531.28	3	396.1	428.1	48.1	51.9	1.1
			97	FSGSGSGTDFTLK	iTRAQ4plex@N-term; Lys->Gln@13		0.0	1446.67	724.34	1446.67	724.34	2	2854.7	1548.6	64.8	35.2	0.5
			97	FSGSGSGTDFTLK	iTRAQ4plex@N-term; Lys->Gln@13		0.0	1446.71	724.36	1446.67	724.34	2	1436.4	952.7	60.1	39.9	0.7
			97	FSGSGSGTDFTLK	iTRAQ4plex@N-term; Lys->Gln@13		0.0	1446.67	724.34	1446.67	724.34	2	1337.4	808.0	62.3	37.7	0.6
			97	FSGSGSGTDFTLK	iTRAQ4plex@N-term; Lys->Gln@13		0.0	1446.71	724.36	1446.67	724.34	2	590.9	403.7	59.4	40.6	0.7
			96	FSGVPDR	iTRAQ4plex@N-term		0.0	920.48	461.25	920.48	461.25	2	19766.8	15804.2	55.6	44.4	0.8
			42	FSGVPDR	iTRAQ4plex@N-term; Oxidation(F)@1		0.0	936.50	469.26	936.48	469.25	2					
			25	TFGGGTK	iTRAQ4plex@N-term; Lys->Gln@7	cleaved P-T@N-term	0.1	810.48	406.25	810.40	406.21	2					
			10	FSGSGSGTDFTLK	iTRAQ4plex@N-term; Methyl(S)@2; Dehydrated(D)@9; iTRAQ4plex(K)@13		0.0	1586.79	529.94	1586.82	529.95	3					
			10	FSGSGSGTDFTLK	iTRAQ4plex@N-term; Methyl(S)@2; Lys->Gln@13		0.0	1460.68	731.35	1460.69	731.35	2					
			10	FSGSGSGTDFTLK	iTRAQ4plex@N-term; Methyl(S)@2; Lys->Gln@13		0.0	1460.69	731.35	1460.69	731.35	2					
			10	FSGSGSGTDFTLK	iTRAQ4plex@N-term; Methyl(S)@2; Lys->Gln@13		0.0	1460.69	731.35	1460.69	731.35	2					
			10	FSGSGSGTDFTLK	iTRAQ4plex@N-term; Methyl(S)@2; Lys->Gln@13		0.0	1460.68	731.35	1460.69	731.35	2					
			10	FSGSGSGTDFTLKISR	iTRAQ4plex@N-term; Methyl(S)@2; Thr->Pro@11; iTRAQ4plex(K)@13	missed K-I@13	0.0	1957.07	653.36	1957.05	653.36	3					
															Average	0.8
															Stdev	0.3
IPI:IPI00844736.1	12KDA PROTEI	12 kDa protein; LOC674678 similar to histone H4	99	DAVTYTEH	iTRAQ4plex@N-term	cleaved H-A@C-term	0.0	1078.49	540.25	1078.51	540.26	2	869.0	1405.3	38.2	61.8	1.6
			99	VFLENVIR	iTRAQ4plex@N-term		0.0	1132.65	567.33	1132.67	567.34	2	3341.5	2448.4	57.7	42.3	0.7
			99	ISGLIYEETR	iTRAQ4plex@N-term		0.0	1323.67	662.84	1323.72	662.87	2	1648.1	1218.5	57.5	42.5	0.7
			99	ISGLIYEETR	iTRAQ4plex@N-term		0.0	1323.67	662.84	1323.72	662.87	2	1624.9	1200.9	57.5	42.5	0.7
			99	ISGLIYEETR	iTRAQ4plex@N-term		0.0	1323.70	662.86	1323.72	662.87	2	1140.1	897.6	56.0	44.0	0.8
			99	DAVTYTEH	iTRAQ4plex@N-term	cleaved H-A@C-term	0.0	1078.50	540.26	1078.51	540.26	2	776.6	1431.7	35.2	64.8	1.8
			99	ISGLIYEETR	iTRAQ4plex@N-term		0.0	1323.72	662.87	1323.72	662.87	2	547.2	448.2	55.0	45.0	0.8
			99	DAVTYTEHAK	iTRAQ4plex@N-term; iTRAQ4plex(K)@10		0.0	1421.76	474.93	1421.74	474.92	3	697.2	599.9	53.8	46.2	0.9
			99	TVTAMDVVYALK	iTRAQ4plex@N-term; iTRAQ4plex(K)@12		0.0	1597.91	799.96	1597.90	799.96	2	200.0	286.1	41.1	58.9	1.4
			99	TVTAMDVVYALKR	iTRAQ4plex@N-term; iTRAQ4plex(K)@12	missed K-R@12	0.0	1754.00	585.67	1754.00	585.67	3	75.7	30.8	71.1	28.9	0.4
			99	TVTAMDVVYALK	iTRAQ4plex@N-term; iTRAQ4plex(K)@12		0.0	1597.89	533.64	1597.90	533.64	3	65.6	58.2	53.0	47.0	0.9
			99	TVTAMDVVYALK	iTRAQ4plex@N-term; iTRAQ4plex(K)@12		2.0	1599.88	534.30	1597.90	533.64	3	52.5	65.1	44.6	55.4	1.2
			99	TVTAMDVVYALK	iTRAQ4plex@N-term; iTRAQ4plex(K)@12		-8.1	1589.82	530.95	1597.90	533.64	3	50.8	60.9	45.5	54.5	1.2
			99	TVTAMDVVYALK	iTRAQ4plex@N-term; Lys->Arg@12		0.0	1481.84	741.93	1481.80	741.91	2	38.7	141.5	21.5	78.5	3.7
			99	TVTAMDVVYALK	iTRAQ4plex@N-term; Lys->Arg@12		0.0	1481.84	741.93	1481.80	741.91	2	15.3	81.7	15.8	84.2	5.3
			99	TVTAMDVVYALK	iTRAQ4plex@N-term; Lys->Gln@12		0.0	1453.77	727.89	1453.76	727.89	2	369.4	304.7	54.8	45.2	0.8
			99	DNIQGITKPAIR	iTRAQ4plex@N-term; Lys->Gln@8		0.1	1468.86	490.63	1468.81	490.61	3	1216.1	670.8	64.5	35.5	0.6
			99	DNIQGITKPAIR	iTRAQ4plex@N-term; Lys->Gln@8; Dimethyl(R)@12		0.0	1496.86	499.96	1496.84	499.96	3	178.2	330.2	35.1	64.9	1.9
			99	ISGLIYEETR	iTRAQ4plex@N-term; Thr->Glu@9		0.0	1351.71	676.86	1351.71	676.86	2	297.3	775.5	27.7	72.3	2.6
			99	ISGLIYEETR	iTRAQ4plex@N-term; Thr->Glu@9		0.0	1351.74	676.88	1351.71	676.86	2	295.5	725.7	28.9	71.1	2.5
			99	TVTAMDVVYALKR	iTRAQ4plex@N-term; Tyr->Met@9; iTRAQ4plex(K)@12; Carbamyl(R)@13	missed K-R@12	0.0	1764.97	589.33	1764.98	589.34	3	146.1	107.2	57.7	42.3	0.7
			98	DAVTYTEHAK	iTRAQ4plex@N-term; No iTRAQ4plex(K)@10		0.0	1277.68	426.90	1277.64	426.89	3	346.6	178.7	66.0	34.0	0.5
			93	VFLENVIR	iTRAQ4plex@N-term		0.0	1132.69	567.35	1132.67	567.34	2	282.3	185.4	60.4	39.6	0.7
			89	VFLENVIR	iTRAQ4plex@N-term; Dimethyl(R)@8		0.0	1160.69	581.35	1160.70	581.36	2	108.2	343.8	23.9	76.1	3.2
															Average	1.5
															Stdev	1.2
IPI:IPI00461514.4	HIST1H2BG	Histone cluster 1, H2bg	99	AMGIMNSFVNDIFER	iTRAQ4plex@N-term		-1.9	1884.99	943.50	1886.91	944.46	2	1924.5	1016.6	65.4	34.6	0.5
			99	AMGIMNSFVNDIFER	iTRAQ4plex@N-term		3.0	1889.95	630.99	1886.91	629.98	3	166.8	93.1	64.2	35.8	0.6
			99	AMGIMNSFVNDIFER	iTRAQ4plex@N-term		0.0	1886.93	629.98	1886.91	629.98	3	46.2	36.3	56.1	43.9	0.8
			99	AMGIMNSFVNDIFER	iTRAQ4plex@N-term		0.1	1887.04	630.02	1886.91	629.98	3	18.7	39.5	32.1	67.9	2.1
			99	AMGIMNSFVNDIFER	iTRAQ4plex@N-term; Deamidated(N)@6; Deamidated(N)@10		0.0	1888.89	630.64	1888.88	630.63	3	36.6	49.6	42.4	57.6	1.4
			99	LLLPGELAK	iTRAQ4plex@N-term; iTRAQ4plex(K)@9		0.0	1240.77	621.39	1240.80	621.41	2	2016.8	1340.7	60.1	39.9	0.7
			99	ESYSVYVYK	iTRAQ4plex@N-term; iTRAQ4plex(K)@9		0.0	1424.73	713.37	1424.74	713.38	2	1194.6	809.0	59.6	40.4	0.7
			99	ESYSVYVYK	iTRAQ4plex@N-term; iTRAQ4plex(K)@9		0.0	1424.72	713.37	1424.74	713.38	2	985.9	711.1	58.1	41.9	0.7
			99	QVHPDTGISSK	iTRAQ4plex@N-term; Lys->Gln@11		0.1	1311.72	438.25	1311.65	438.23	3	374.1	279.6	57.2	42.8	0.7
			99	LLLPGELAK	iTRAQ4plex@N-term; Lys->Gln@9		0.0	1096.68	549.35	1096.66	549.34	2	1880.2	975.9	65.8	34.2	0.5
			99	AMGIMNSFVNDIFER	iTRAQ4plex@N-term; Oxidation(M)@5		0.0	1902.94	635.32	1902.91	635.31	3	12.3	14.7	45.4	54.6	1.2
			98	ESYSVYVYK	iTRAQ4plex@N-term; Lys->Gln@9		0.0	1280.62	641.32	1280.60	641.31	2	535.8	259.5	67.4	32.6	0.5
			81	IASEASR	iTRAQ4plex@N-term		0.0	876.52	439.27	876.48	439.25	2	220.5	173.6	56.0	44.0	0.8
			71	IASEASR	iTRAQ4plex@N-term		3.0	879.53	440.77	876.48	439.25	2	381.9	265.5	59.0	41.0	0.7
			69	IASEASR	iTRAQ4plex@N-term		0.0	876.51	439.26	876.48	439.25	2	56.3	60.8	48.1	51.9	1.1
			47	AMGIMNSFVNDIFER	iTRAQ4plex@N-term; Glu->Arg@14; Deamidated(R)@15		0.1	1915.02	639.35	1914.96	639.33	3					
			26	EIQTAVR	iTRAQ4plex@N-term		0.0	959.56	480.79	959.55	480.78	2					
			25	EIQTAVR	iTRAQ4plex@N-term		0.0	959.57	480.79	959.55	480.78	2					
			22	STITSR	iTRAQ4plex@N-term		0.0	807.45	404.73	807.46	404.74	2					
			15	AMGIMNSFVNDIFER	iTRAQ4plex@N-term; Oxidation(M)@5; Deamidated(N)@6; Methyl(S)@7		0.1	1918.01	640.34	1917.91	640.31	3					
															Average	0.9
															Stdev	0.4
IPI:IPI00785410.1	COX5B	Cytochrome c oxidase, subunit Vb	99	ASGGGVPTDEEQATGLER	iTRAQ4plex@N-term	cleaved M-A@N-term	0.0	1916.92	639.98	1916.92	639.98	3	66.2	90.3	42.3	57.7	1.4
			99	ASGGGVPTDEEQATGLER	iTRAQ4plex@N-term	cleaved M-A@N-term	0.0	1916.91	639.98	1916.92	639.98	3	109.2	115.9	48.5	51.5	1.1
			99	GLDPYNMLPPK	iTRAQ4plex@N-term; iTRAQ4plex(K)@11		0.0	1531.82	511.61	1531.83	511.62	3	69.9	90.4	43.6	56.4	1.3
			87	EIMIAAQK	iTRAQ4plex@N-term; Lys->Gln@8		0.0	1046.60	524.31	1046.56	524.28	2	1021.3	893.4	53.3	46.7	0.9
															Average	1.1
															Stdev	0.2
IPI:IPI00311147.1	C1QB	Complement component 1, q subcomponent, beta polypeptide	99	FEKVITNANENYEPR	iTRAQ4plex@N-term; Dehydrated(E)@2; iTRAQ4plex(K)@3; Val->Leu@4	missed K-V@3	0.0	2107.09	703.37	2107.09	703.37	3	416.7	314.6	57.0	43.0	0.8
			99	VITNANENYEPR	iTRAQ4plex@N-term		-6.0	1556.75	779.38	1562.78	782.40	2	6333.7	3491.3	64.5	35.5	0.6
			99	VITNANENYEPR	iTRAQ4plex@N-term		0.0	1562.76	782.39	1562.78	782.40	2	5548.2	3804.5	59.3	40.7	0.7
			99	VITNANENYEPR	iTRAQ4plex@N-term		0.0	1562.80	782.41	1562.78	782.40	2	3781.5	2276.8	62.4	37.6	0.6
			99	VITNANENYEPR	iTRAQ4plex@N-term		0.0	1562.79	782.40	1562.78	782.40	2	2919.5	1878.6	60.8	39.2	0.6
			99	VITNANENYEPR	iTRAQ4plex@N-term		0.0	1562.79	782.40	1562.78	782.40	2	1795.0	1203.6	59.9	40.1	0.7
			99	VPGLYYFTYHASSR	iTRAQ4plex@N-term		0.0	1803.91	602.31	1803.91	602.31	3	287.3	248.9	53.6	46.4	0.9
			99	VPGLYYFTYHASSR	iTRAQ4plex@N-term		0.0	1803.87	602.30	1803.91	602.31	3	240.0	191.3	55.7	44.3	0.8
			99	VITNANENYEPR	iTRAQ4plex@N-term; Asn->Pro@8		0.0	1545.77	516.26	1545.79	516.27	3	107.3	118.0	47.6	52.4	1.1
			80	VAFSALR	iTRAQ4plex@N-term		-6.0	900.53	451.27	906.54	454.28	2	11313.1	7188.1	61.1	38.9	0.6
			75	VAFSALR	iTRAQ4plex@N-term		0.0	906.53	454.27	906.54	454.28	2	1266.4	1297.3	49.4	50.6	1.0
			66	VAFSALR	iTRAQ4plex@N-term		0.0	906.53	454.27	906.54	454.28	2	1191.9	834.4	58.8	41.2	0.7
			41	VAFSALR	iTRAQ4plex@N-term; Dimethyl(R)@7		0.0	934.55	468.28	934.57	468.29	2					
			34	DSMQK	iTRAQ4plex@N-term; iTRAQ4plex(K)@5		0.0	895.51	448.76	895.47	448.74	2					
			33	GLPGLAGDLGEFGEKGDPGIPGTPGKVGPK	iTRAQ4plex@N-term; Oxidation(P)@3; Oxidation(F)@12; iTRAQ4plex(K)@15; Lys->Ser@26; Oxidation(P)@29; iTRAQ4plex(K)@30	missed K-G@15; missed K-V@26	0.0	3255.67	814.93	3255.70	814.93	4					
			27	VAFSALR	iTRAQ4plex@N-term; Dimethyl(R)@7		0.0	934.56	468.29	934.57	468.29	2					
			11	GLPGLAGDLGEFGEKGDPGIPGTPGKVGP	iTRAQ4plex@N-term; Oxidation(P)@3; iTRAQ4plex(K)@15; Oxidation(P)@24; Lys->Ser@26	cleaved P-K@C-term; missed K-G@15; missed	-0.2	2967.35	990.13	2967.51	990.18	3					
															Average	0.8
															Stdev	0.2
IPI:IPI00224210.5	UQCRQ	Ubiquinol-cytochrome c reductase, complex III subunit VI	99	AFPSYFSK	iTRAQ4plex@N-term; iTRAQ4plex(K)@8		0.0	1233.65	617.83	1233.66	617.84	2	2145.7	1772.4	54.8	45.2	0.8
			99	HVISYSLSPFEQR	iTRAQ4plex@N-term		0.0	1705.84	569.62	1705.89	569.64	3	331.7	237.4	58.3	41.7	0.7
			94	EFGNLAR	iTRAQ4plex@N-term		0.0	949.52	475.77	949.51	475.76	2	3030.5	2895.3	51.1	48.9	1.0
			69	GIPNVLR	iTRAQ4plex@N-term		0.0	911.56	456.79	911.57	456.79	2	3504.4	2797.5	55.6	44.4	0.8
															Average	0.8
															Stdev	0.1
IPI:IPI00138144.1	IGKV3-10	Immunoglobulin kappa variable 3-10	99	ASNLESGVPAR	iTRAQ4plex@N-term	cleaved L-A@N-term	1.0	1244.69	623.35	1243.66	622.84	2	3557.9	2687.2	57.0	43.0	0.8
			99	NIVLTQSPASLAVSLGQR	iTRAQ4plex@N-term		0.0	1997.10	666.71	1997.14	666.72	3	31.9	29.2	52.2	47.8	0.9
			99	NIVLTQSPASLAVSLGQR	iTRAQ4plex@N-term		0.0	1997.10	666.71	1997.14	666.72	3	24.8	21.9	53.2	46.8	0.9
			99	LLIYLASNLESGVPAR	iTRAQ4plex@N-term		0.0	1859.09	620.70	1859.06	620.70	3	1.7	4.4	28.1	71.9	2.6
			84	IVLTQSPASLAVSLGQR	iTRAQ4plex@N-term; Val->Cys@2; Pyridylethyl(C)@2; Deamidated(Q)@5	cleaved N-I@N-term	0.1	1993.15	665.39	1993.08	665.37	3	61.7	64.8	48.8	51.2	1.1
			81	FSGSGSR	iTRAQ4plex@N-term		0.0	840.46	421.24	840.42	421.22	2	178.0	135.1	56.9	43.1	0.8
			70	NIVLTQSPASLAVSLGQR	iTRAQ4plex@N-term; Deamidated(N)@1		0.0	1998.14	667.05	1998.12	667.05	3	42.1	50.5	45.4	54.6	1.2
			70	NIVLTQSPASLAVSLGQR	iTRAQ4plex@N-term; Deamidated(N)@1		0.0	1998.10	667.04	1998.12	667.05	3	25.0	16.4	60.3	39.7	0.7
			70	NIVLTQSPASLAVSLGQR	iTRAQ4plex@N-term; Deamidated(N)@1; Gln->Arg@17		0.0	2026.13	676.39	2026.17	676.40	3	8.6	16.0	34.9	65.1	1.9
			27	TFGGGTK	iTRAQ4plex@N-term; Lys->Gln@7	cleaved Y-T@N-term	0.1	810.48	406.25	810.40	406.21	2					
															Average	1.2
															Stdev	0.6
IPI:IPI00153381.1	UQCR10	Cytochrome b-c1 complex subunit 9	99	AFDQGADAIYEHINEGK	iTRAQ4plex@N-term; iTRAQ4plex(K)@17		0.0	2165.07	542.27	2165.06	542.27	4	50.8	60.8	45.5	54.5	1.2
			99	AFDQGADAIYEHINEGK	iTRAQ4plex@N-term; Lys->Gln@17		0.0	2020.92	674.65	2020.92	674.65	3	68.4	82.0	45.5	54.5	1.2
			98	LYSLLFR	iTRAQ4plex@N-term		0.0	1054.65	528.33	1054.63	528.32	2	509.1	390.0	56.6	43.4	0.8
															Average	1.1
															Stdev	0.2
IPI:IPI00127560.1	TTR	Transthyretin	99	GSPAVDVAVK	iTRAQ4plex@N-term; Lys->Gln@10		0.0	1085.63	543.82	1085.58	543.80	2	1392.5	951.6	59.4	40.6	0.7
			99	HYTIAALLSPYSYSTTAVVSNPQN	iTRAQ4plex@N-term		0.0	2740.35	914.46	2740.38	914.47	3	36.4	56.2	39.3	60.7	1.5
			99	TLGISPFHEFADVVFTANDSGHR	iTRAQ4plex@N-term		0.0	2660.32	666.09	2660.31	666.08	4	10.5	13.7	43.3	56.7	1.3
			99	GSPAVDVAVK	iTRAQ4plex@N-term; iTRAQ4plex(K)@10		0.0	1229.72	615.87	1229.72	615.87	2	1128.8	807.6	58.3	41.7	0.7
			99	GSPAVDVAVK	iTRAQ4plex@N-term; Lys->Gln@10		0.1	1085.64	543.83	1085.58	543.80	2	442.5	258.7	63.1	36.9	0.6
			98	HYTIAALLSPYSYSTTAVVSNPQN	iTRAQ4plex@N-term		0.1	2740.44	914.49	2740.38	914.47	3	31.6	37.9	45.4	54.6	1.2
			95	FVEGVYR	iTRAQ4plex@N-term		0.0	1012.57	507.29	1012.55	507.28	2	1705.0	1414.6	54.7	45.3	0.8
															Average	1.0
															Stdev	0.4
IPI:IPI00119224.1	SNRPD3	Small nuclear ribonucleoprotein D3	99	FLILPDMLK	iTRAQ4plex@N-term; iTRAQ4plex(K)@9		0.0	1376.84	459.95	1376.83	459.95	3	8.8	9.4	48.3	51.7	1.1
			99	VAQLEQVYIR	iTRAQ4plex@N-term		0.0	1361.76	681.89	1361.78	681.90	2	496.5	381.2	56.6	43.4	0.8
			99	VAQLEQVYIR	iTRAQ4plex@N-term		0.0	1361.78	681.90	1361.78	681.90	2	419.8	328.7	56.1	43.9	0.8
			99	FLILPDMLK	iTRAQ4plex@N-term; Lys->Gln@9		0.0	1232.73	617.37	1232.70	617.36	2	54.7	54.4	50.2	49.8	1.0
			88	FLILPDMLK	iTRAQ4plex@N-term; Lys->Gln@9		0.1	1232.77	617.39	1232.70	617.36	2	109.4	59.8	64.7	35.3	0.5
			78	FLILPDMLK	iTRAQ4plex@N-term; Lys->Gln@9		0.1	1232.75	617.38	1232.70	617.36	2	73.4	43.9	62.6	37.4	0.6
			38	FLILPDMLK	iTRAQ4plex@N-term; Lys->Gln@9		0.0	1232.74	617.38	1232.70	617.36	2					
															Average	0.8
															Stdev	0.2
IPI:IPI00464383.6	CE9	Immunoglobulin light chain variable region	99	DIQMTQTTSSLSASLGDR	iTRAQ4plex@N-term		0.0	2054.01	685.68	2054.01	685.68	3	21.3	18.6	53.4	46.6	0.9
			70	LLIYYTSR	iTRAQ4plex@N-term		0.0	1171.67	586.84	1171.67	586.84	2	1111.0	793.5	58.3	41.7	0.7
			59	LLIYYTSR	iTRAQ4plex@N-term		0.0	1171.68	586.85	1171.67	586.84	2	934.2	624.5	59.9	40.1	0.7
			44	DIQMTQTTSSLSASLGDR	iTRAQ4plex@N-term; Dehydrated(T)@8; Ser->Glu@14		0.0	2077.99	693.67	2078.01	693.68	3					
			36	TFGGGTK	iTRAQ4plex@N-term; Lys->Gln@7		0.1	810.48	406.25	810.40	406.21	2					
															Average	0.8
															Stdev	0.1
IPI:IPI00110487.1	2310003C23RIK	RIKEN cDNA 2310003C23 gene	99	LIMNYLVTEGFK	iTRAQ4plex@N-term; Lys->Gln@12		0.1	1570.89	786.45	1570.82	786.42	2	119.2	93.1	56.2	43.8	0.8
			99	VWSEVNQAVLDYENR	iTRAQ4plex@N-term		0.0	1964.95	655.99	1964.97	656.00	3	39.2	28.9	57.5	42.5	0.7
			99	MESGIEPSVDLETLDER	iTRAQ4plex@N-term		0.0	2062.97	688.66	2062.99	688.67	3	13.6	27.7	32.9	67.1	2.0
			99	VWSEVNQAVLDYENR	iTRAQ4plex@N-term		0.0	1964.98	656.00	1964.97	656.00	3	8.6	16.0	34.9	65.1	1.9
			99	LIMNYLVTEGFK	iTRAQ4plex@N-term; Lys->Gln@12		0.0	1570.84	786.43	1570.82	786.42	2	95.1	50.7	65.2	34.8	0.5
			53	LLLWAQNELDQK	iTRAQ4plex@N-term; iTRAQ4plex(K)@12		0.0	1757.98	587.00	1757.99	587.00	3	26.2	32.5	44.7	55.3	1.2
															Average	1.2
															Stdev	0.6
IPI:IPI00125929.2	NDUFA4	NADH dehydrogenase (ubiquinone) 1 alpha subcomplex, 4	99	FYSVNVDYSK	iTRAQ4plex@N-term; Lys->Gln@10		0.0	1364.66	683.34	1364.64	683.33	2	493.4	266.2	65.0	35.0	0.5
			99	LALFNPDVSWDR	iTRAQ4plex@N-term		0.0	1575.83	788.92	1575.82	788.92	2	747.8	768.3	49.3	50.7	1.0
			99	LALFNPDVSWDR	iTRAQ4plex@N-term		0.0	1575.85	788.93	1575.82	788.92	2	642.1	506.5	55.9	44.1	0.8
			99	LALFNPDVSWDR	iTRAQ4plex@N-term		0.0	1575.80	788.91	1575.82	788.92	2	101.0	88.5	53.3	46.7	0.9
			99	FYSVNVDYSK	iTRAQ4plex@N-term; Lys->Gln@10		0.0	1364.66	683.34	1364.64	683.33	2	1195.5	735.3	61.9	38.1	0.6
			99	FYSVNVDYSK	iTRAQ4plex@N-term; Lys->Gln@10		0.0	1364.66	683.34	1364.64	683.33	2	854.4	504.1	62.9	37.1	0.6
			97	FYSVNVDYSK	iTRAQ4plex@N-term; Lys->Arg@10		0.0	1392.69	697.35	1392.68	697.35	2	307.7	392.6	43.9	56.1	1.3
			97	FYSVNVDYSK	iTRAQ4plex@N-term; Lys->Arg@10		0.0	1392.69	697.35	1392.68	697.35	2	212.8	284.7	42.8	57.2	1.3
															Average	0.9
															Stdev	0.3
IPI:IPI00127841.3	SLC25A5	Solute carrier family 25 (mitochondrial carrier, adenine nucleotide translocator), member 5	99	DFLAGGVAAAISK	iTRAQ4plex@N-term; Lys->Gln@13		0.0	1362.76	682.39	1362.73	682.37	2	175.4	155.9	53.0	47.0	0.9
			99	GAWSNVLR	iTRAQ4plex@N-term		0.0	1045.57	523.79	1045.58	523.80	2	1918.4	1932.2	49.8	50.2	1.0
			99	EQGVLSFWR	iTRAQ4plex@N-term		0.0	1264.64	633.33	1264.67	633.34	2	402.0	515.4	43.8	56.2	1.3
			99	EQGVLSFWR	iTRAQ4plex@N-term		0.0	1264.66	633.34	1264.67	633.34	2	395.4	497.3	44.3	55.7	1.3
			99	YFPTQALNFAFK	iTRAQ4plex@N-term; iTRAQ4plex(K)@12		0.0	1733.94	578.99	1733.94	578.99	3	87.0	123.5	41.3	58.7	1.4
			99	LLLQVQHASK	iTRAQ4plex@N-term; Lys->Gln@10		0.0	1279.75	427.59	1279.74	427.59	3	390.0	343.8	53.2	46.8	0.9
			99	YFPTQALNFAFK	iTRAQ4plex@N-term; Lys->Gln@12		0.1	1589.86	795.94	1589.80	795.91	2	334.4	361.4	48.1	51.9	1.1
			99	DFLAGGVAAAISK	iTRAQ4plex@N-term; Lys->Gln@13		0.1	1362.79	682.40	1362.73	682.37	2	227.8	174.4	56.6	43.4	0.8
			97	MMMQSGR	iTRAQ4plex@N-term		0.0	983.45	492.73	983.45	492.73	2	4196.9	4578.0	47.8	52.2	1.1
			97	GNLANVIR	iTRAQ4plex@N-term		0.0	999.59	500.80	999.59	500.80	2	3043.6	3788.6	44.5	55.5	1.2
			94	YFPTQALNFAFK	iTRAQ4plex@N-term; Lys->Gln@12		0.1	1589.90	795.96	1589.80	795.91	2	54.3	66.4	45.0	55.0	1.2
			93	YFPTQALNFAFK	iTRAQ4plex@N-term; Lys->Gln@12		0.1	1589.88	795.95	1589.80	795.91	2	116.6	114.8	50.4	49.6	1.0
			92	TAVAPIER	iTRAQ4plex@N-term		0.0	999.59	500.80	999.58	500.80	2	14230.6	13072.5	52.1	47.9	0.9
			11	YFPTQALNFAFK	iTRAQ4plex@N-term; Lys->Arg@12		0.0	1617.88	540.30	1617.84	540.29	3					
															Average	1.1
															Stdev	0.2
IPI:IPI00831162.2	LOC100046973	Similar to Human Ig rearranged gamma chain	99	YASQSISGIPSR	iTRAQ4plex@N-term		0.0	1408.71	705.36	1408.74	705.38	2	3002.2	2068.8	59.2	40.8	0.7
			99	YASQSISGIPSR	iTRAQ4plex@N-term		0.0	1408.73	705.37	1408.74	705.38	2	1645.5	1189.2	58.0	42.0	0.7
			99	YASQSISGIPSR	iTRAQ4plex@N-term		0.0	1408.71	705.36	1408.74	705.38	2	1596.9	1166.9	57.8	42.2	0.7
			99	YASQSISGIPSR	iTRAQ4plex@N-term		0.0	1408.72	705.37	1408.74	705.38	2	707.3	573.2	55.2	44.8	0.8
			99	SPQLLVYAATNLADGVPSR	iTRAQ4plex@N-term		0.0	2115.18	706.07	2115.14	706.06	3	32.0	25.9	55.2	44.8	0.8
			99	SPQLLVYAATNLADGVPSR	iTRAQ4plex@N-term		0.0	2115.19	706.07	2115.14	706.06	3	24.8	23.0	51.9	48.1	0.9
			99	ASQSISNYLHWYQQK	iTRAQ4plex@N-term; Deamidated(N)@7; iTRAQ4plex(K)@15		0.0	2141.07	536.27	2141.08	536.28	4	108.2	94.5	53.4	46.6	0.9
			99	ASQSISNYLHWYQQK	iTRAQ4plex@N-term; Deamidated(N)@7; Lys->Gln@15		0.0	1996.98	666.67	1996.94	666.65	3	89.7	49.6	64.4	35.6	0.6
			99	ASENIYSNLAWYQQK	iTRAQ4plex@N-term; iTRAQ4plex(K)@15		0.0	2102.08	701.70	2102.07	701.70	3	49.2	54.4	47.5	52.5	1.1
			99	ASENIYSNLAWYQQK	iTRAQ4plex@N-term; iTRAQ4plex(K)@15		0.0	2102.08	701.70	2102.07	701.70	3	44.1	45.0	49.5	50.5	1.0
			99	LLIKYASQSISGIPSR	iTRAQ4plex@N-term; iTRAQ4plex(K)@4; Tyr->Phe@5; Ser->Asn@7	missed K-Y@4	-0.1	2031.15	678.06	2031.21	678.08	3	62.4	45.4	57.9	42.1	0.7
			99	FSGSGSGTQYSLK	iTRAQ4plex@N-term; Lys->Gln@13		0.0	1461.74	731.87	1461.69	731.85	2	442.1	270.4	62.0	38.0	0.6
			67	YASQSISGIPSR	iTRAQ4plex@N-term; Deamidated(Q)@4		0.0	1409.71	705.86	1409.73	705.87	2	918.3	659.8	58.2	41.8	0.7
															Average	0.8
															Stdev	0.2
IPI:IPI00553333.2	HBB-B1	Hemoglobin, beta adult major chain	99	LLVVYPWTQR	iTRAQ4plex@N-term		0.0	1417.85	709.93	1417.82	709.92	2	320.2	200.3	61.5	38.5	0.6
			99	LLVVYPWTQR	iTRAQ4plex@N-term		0.0	1417.83	709.92	1417.82	709.92	2	678.2	420.1	61.7	38.3	0.6
			99	VNSDEVGGEALGR	iTRAQ4plex@N-term		0.0	1445.71	723.86	1445.72	723.87	2	384.7	238.0	61.8	38.2	0.6

			99 VITAFNDGLNHLDSLK	iTRAQ4plex@N-term; iTRAQ4plex(K)@16		0.0	2044.08	682.37	2044.12	682.38	3	38.5	47.4	44.8	55.2	1.2
			99 YFDSFGDLSSASAIMGNAK	iTRAQ4plex@N-term; iTRAQ4plex(K)@19		0.0	2268.10	757.04	2268.10	757.04	3	46.4	30.8	60.1	39.9	0.7
															Average	0.8
															Stdev	0.3
IPI:IPI00473844.3	HIST2H2AA1	Histone cluster 2, H2aa1	99 AGLQFPVGR	iTRAQ4plex@N-term		0.0	1087.62	544.82	1087.63	544.82	2	11382.7	7236.7	61.1	38.9	0.6
			99 AGLQFPVGR	iTRAQ4plex@N-term		0.0	1087.63	544.82	1087.63	544.82	2	7859.4	5554.6	58.6	41.4	0.7
			99 VTIAQGGVLPNIQAVLLPK	iTRAQ4plex@N-term; iTRAQ4plex(K)@19		0.0	2218.35	740.46	2218.37	740.46	3	17.6	18.9	48.3	51.7	1.1
			99 VTIAQGGVLPNIQAVLLPK	iTRAQ4plex@N-term; iTRAQ4plex(K)@19		0.0	2218.37	740.47	2218.37	740.46	3	15.9	15.7	50.3	49.7	1.0
			99 VTIAQGGVLPNIQAVLLPK	iTRAQ4plex@N-term; Lys->Gln@19		0.1	2074.28	692.43	2074.23	692.42	3	10.8	6.1	63.8	36.2	0.6
			99 VTIAQGGVLPNIQAVLLPK	iTRAQ4plex@N-term; Lys->Gln@19		0.1	2074.29	692.44	2074.23	692.42	3	1.7	3.3	34.3	65.7	1.9
			75 VTIAQGGVLPNIQAVLLPK	iTRAQ4plex@N-term; iTRAQ4plex(K)@19		-1.0	2217.37	555.35	2218.37	555.60	4	14.0	16.8	45.4	54.6	1.2
			41 NDEELNK	iTRAQ4plex@N-term; Deamidated(N)@6; iTRAQ4plex(K)@7		0.1	1149.65	575.83	1149.58	575.80	2					
			16 NDEELNK	iTRAQ4plex@N-term; Deamidated(N)@6; Lys->Gln@7		0.1	1005.52	503.77	1005.44	503.73	2					
			14 NDEELNK	iTRAQ4plex@N-term; Lys->Gln@7		0.1	1004.52	503.27	1004.45	503.23	2					
															Average	1.0
															Stdev	0.5
IPI:IPI00118963.1	MRPL12	Mitochondrial ribosomal protein L12	99 IQDVGLMPMGGMVPGPVSAAAPASEAAEEEDVP	iTRAQ4plex@N-term; iTRAQ4plex(K)@34		0.0	3637.84	910.47	3637.79	910.46	4	21.1	23.1	47.8	52.2	1.1
			99 NYVQGINLVQAK	iTRAQ4plex@N-term; Lys->Gln@12		0.0	1489.85	745.93	1489.80	745.91	2	317.7	169.9	65.2	34.8	0.5
															Average	0.8
															Stdev	0.4
IPI:IPI00555000.2	UQCRB	Ubiquinol-cytochrome c reductase binding protein	99 SAVSASSK	iTRAQ4plex@N-term; iTRAQ4plex(K)@8		0.0	1023.60	512.81	1023.58	512.80	2	105.2	76.2	58.0	42.0	0.7
			96 ALDLTMR	iTRAQ4plex@N-term		0.0	962.53	482.27	962.53	482.27	2	1303.6	882.6	59.6	40.4	0.7
			19 WLDGFR	iTRAQ4plex@N-term		0.0	936.49	469.25	936.49	469.25	2					
															Average	0.7
															Stdev	0.0
IPI:IPI00742310.3	EWSR1	Ewing sarcoma breakpoint region 1	99 AAVEWFDGK	iTRAQ4plex@N-term; Lys->Gln@9		0.0	1165.58	583.80	1165.55	583.78	2	243.4	248.5	49.5	50.5	1.0
			99 GDATVSYEDPPTAK	iTRAQ4plex@N-term; iTRAQ4plex(K)@14		0.0	1737.86	580.29	1737.87	580.30	3	294.5	254.0	53.7	46.3	0.9
			99 AAVEWFDGKDFQGSK	iTRAQ4plex@N-term; iTRAQ4plex(K)@9; Lys->Gln@15		0.1	1972.01	658.35	1971.96	658.33	3	820.2	489.2	62.6	37.4	0.6
			99 GDATVSYEDPPTAK	iTRAQ4plex@N-term; Lys->Arg@14		0.0	1621.79	541.60	1621.77	541.60	3	59.9	63.9	48.4	51.6	1.1
			99 GDATVSYEDPPTAK	iTRAQ4plex@N-term; Lys->Gln@14		0.1	1593.82	797.92	1593.73	797.87	2	91.6	47.5	65.9	34.1	0.5
			99 AGDWQCPNPGCGNQNFAWR	iTRAQ4plex@N-term; No Pyridylethyl(C)@6; Cys->Thr@11		-0.1	2261.94	754.99	2262.01	755.01	3	202.0	80.9	71.4	28.6	0.4
			61 AAVEWFDGK	iTRAQ4plex@N-term; iTRAQ4plex(K)@9		0.0	1309.65	437.56	1309.69	437.57	3	46.3	84.9	35.3	64.7	1.8
			60 DFQGSK	iTRAQ4plex@N-term; iTRAQ4plex(K)@6		0.0	968.53	485.27	968.52	485.27	2	100.0	116.5	46.2	53.8	1.2
															Average	0.9
															Stdev	0.5
IPI:IPI00457419.3	LOC630279	Ig heavy chain V region MOPC 21	99 DVQLVESGGGLVQPGGSR	iTRAQ4plex@N-term	cleaved C-D@N-term	0.0	1897.96	633.66	1898.00	633.67	3	120.2	164.4	42.2	57.8	1.4
			99 NTLFLQMTSLR	iTRAQ4plex@N-term		0.0	1466.82	734.42	1466.80	734.41	2	252.0	212.7	54.2	45.8	0.8
			99 DVQLVESGGGLVQPGGSR	iTRAQ4plex@N-term	cleaved C-D@N-term	0.0	1897.95	633.66	1898.00	633.67	3	129.8	103.5	55.6	44.4	0.8
			98 NTLFLQMTSLR	iTRAQ4plex@N-term		0.0	1466.81	734.41	1466.80	734.41	2	205.7	178.5	53.5	46.5	0.9
			75 SEDTAMYYCARWGNY	iTRAQ4plex@N-term; Pyridylethyl(C)@9; Asn->Gly@14	cleaved Y-P@C-term; missed R-W@11	0.0	2020.84	674.62	2020.86	674.63	3	579.0	480.6	54.6	45.4	0.8
			33 SEDTAMYYCARWGNY	iTRAQ4plex@N-term; Pyridylethyl(C)@9; Asn->Gly@14	cleaved Y-P@C-term; missed R-W@11	0.0	2020.81	674.61	2020.86	674.63	3					
			32 SEDTAMYYCARWGNY	iTRAQ4plex@N-term; Oxidation(M)@6; Pyridylethyl(C)@9; Asn->Gly@14	cleaved Y-P@C-term; missed R-W@11	0.0	2036.88	679.97	2036.85	679.96	3					
															Average	0.9
															Stdev	0.2
IPI:IPI00420972.3	IGH-1A	Immunoglobulin heavy chain 1a (serum IgG2a)	99 DLPAPIER	iTRAQ4plex@N-term		0.0	1053.57	527.79	1053.59	527.80	2	23294.3	21169.9	52.4	47.6	0.9
			99 NTEPVLDSDGSYFMYSK	iTRAQ4plex@N-term; Asp->Asn@9; Oxidation(M)@14; iTRAQ4plex(K)@17		0.0	2255.08	752.70	2255.07	752.70	3	153.1	116.7	56.8	43.2	0.8
			99 TELNYKNTEPVLDSDGSYFMYSK	iTRAQ4plex@N-term; Glu->Thr@2; iTRAQ4plex(K)@6; Asn->His@7; iTRAQ4plex(K)@23	missed K-N@6	0.0	3127.59	782.90	3127.55	782.90	4	322.7	332.3	49.3	50.7	1.0
			99 APQVYVLPPPEEEMTK	iTRAQ4plex@N-term; iTRAQ4plex(K)@16		0.0	2115.07	706.03	2115.12	706.05	3	181.1	201.2	47.4	52.6	1.1
			99 APQVYVLPPPEEEMTK	iTRAQ4plex@N-term; iTRAQ4plex(K)@16		0.0	2115.12	706.05	2115.12	706.05	3	94.1	77.7	54.8	45.2	0.8
			99 NTEPVLDSDGSYFMYSK	iTRAQ4plex@N-term; iTRAQ4plex(K)@17		-0.1	2240.00	747.67	2240.06	747.69	3	687.1	624.3	52.4	47.6	0.9
			99 NTEPVLDSDGSYFMYSK	iTRAQ4plex@N-term; iTRAQ4plex(K)@17		0.0	2240.01	747.68	2240.06	747.69	3	305.9	289.6	51.4	48.6	0.9
			99 NTEPVLDSDGSYFMYSK	iTRAQ4plex@N-term; iTRAQ4plex(K)@17		0.0	2240.03	747.68	2240.06	747.69	3	285.7	243.8	54.0	46.0	0.9
			99 NTEPVLDSDGSYFMYSK	iTRAQ4plex@N-term; iTRAQ4plex(K)@17		1.0	2241.01	748.01	2240.06	747.69	3	155.0	165.2	48.4	51.6	1.1
			99 APQVYVLPPPEEEMTK	iTRAQ4plex@N-term; Lys->Arg@16		0.0	1999.04	667.35	1999.02	667.35	3	36.5	51.7	41.4	58.6	1.4
			99 VVSALPIQHQDWMSGK	iTRAQ4plex@N-term; Lys->Gln@16		0.0	1939.00	647.34	1938.97	647.33	3	584.5	426.2	57.8	42.2	0.7
			99 NTEPVLDSDGSYFMYSK	iTRAQ4plex@N-term; Lys->Gln@17		0.0	2095.91	699.65	2095.92	699.65	3	96.6	61.4	61.1	38.9	0.6
			99 TTAPSVYPLAPVCGDTTGSSVTLGCLVK	iTRAQ4plex@N-term; Pyridylethyl(C)@13; Thr->Ser@17; Pyridylethyl(C)@25; Lys->Gln@28		0.0	3076.51	770.13	3076.54	770.14	4	56.5	56.3	50.1	49.9	1.0
			99 NTEPVLDSDGSYFMYSK	iTRAQ4plex@N-term; Ser->Asp@16; iTRAQ4plex(K)@17		0.0	2268.05	757.02	2268.05	757.02	3	31.2	46.5	40.2	59.8	1.5
			99 APQVYVLPPPEEEMTK	iTRAQ4plex@N-term; Thr->Glu@15; iTRAQ4plex(K)@16		0.0	2143.13	715.38	2143.11	715.38	3	50.6	117.2	30.2	69.8	2.3
			98 APQVYVLPPPEEEMTK	iTRAQ4plex@N-term; Lys->Arg@16		0.0	1999.06	667.36	1999.02	667.35	3	26.4	28.3	48.2	51.8	1.1
			92 APQVYVLPPPEEEMTK	iTRAQ4plex@N-term; Lys->Gln@16		0.0	1970.99	658.00	1970.98	658.00	3	12.4	11.5	51.9	48.1	0.9
			91 NTEPVLDSDGSYFMYSK	iTRAQ4plex@N-term; Amino(Y)@12; Oxidation(M)@14; iTRAQ4plex(K)@17		0.0	2271.09	758.04	2271.06	758.03	3	119.3	90.9	56.7	43.3	0.8
			78 DLPAPIER	iTRAQ4plex@N-term; Pro->Val@5		-0.1	1055.56	528.79	1055.61	528.81	2	6770.8	7898.9	46.2	53.8	1.2
			76 NTEPVLDSDGSYFMYSK	iTRAQ4plex@N-term; iTRAQ4plex(S)@16; Lys->Arg@17		0.0	2268.07	757.03	2268.06	757.03	3	38.1	58.1	39.6	60.4	1.5
			59 APQVYVLPPPEEEMTKK	iTRAQ4plex@N-term; iTRAQ4plex(K)@16; Lys->Gln@17	missed K-K@16	0.0	2243.22	561.81	2243.18	561.80	4	95.0	53.7	63.9	36.1	0.6
			43 APQVYVLPPPEEEMTK	iTRAQ4plex@N-term; Oxidation(M)@14; Lys->Gln@16		0.0	1987.01	663.34	1986.97	663.33	3					
			39 TELNYK	iTRAQ4plex@N-term; iTRAQ4plex(K)@6		0.0	1054.61	528.31	1054.59	528.30	2					
															Average	1.0
															Stdev	0.4
IPI:IPI00856927.1	RPS19	Ribosomal protein S19	99 DVNQQEFVR	iTRAQ4plex@N-term		0.0	1277.66	639.84	1277.65	639.83	2	508.3	411.7	55.3	44.7	0.8
			99 ELAPYDENWFYTR	iTRAQ4plex@N-term		0.0	1846.91	616.64	1846.86	616.63	3	26.0	39.0	40.0	60.0	1.5
			97 ELAPYDENWFYTR	iTRAQ4plex@N-term		0.0	1846.87	616.63	1846.86	616.63	3	19.0	31.8	37.4	62.6	1.7
			25 LTPQGQR	iTRAQ4plex@N-term		0.0	942.56	472.29	942.54	472.28	2					
															Average	1.3
															Stdev	0.5
IPI:IPI00855114.1	IGL-C2	Immunoglobulin lambda chain, constant region 2	99 FMASSFLHLTSDQWR	iTRAQ4plex@N-term		0.0	1968.93	657.32	1968.96	657.33	3	108.7	80.4	57.5	42.5	0.7
			93 ANGTPITQGVDTSNPTK	iTRAQ4plex@N-term; No iTRAQ4plex(K)@17		0.0	1843.98	615.67	1843.94	615.65	3	42.5	38.4	52.5	47.5	0.9
															Average	0.8
															Stdev	0.1
IPI:IPI00850337.1	LOC100048117	Glyceraldehyde-3-phosphate dehydrogenase-like	99 TVDGPSGK	iTRAQ4plex@N-term; iTRAQ4plex(K)@8		0.1	1047.64	524.83	1047.58	524.80	2	44.0	47.1	48.3	51.7	1.1
			99 VIISAPSADAPMFVMGVNHEK	iTRAQ4plex@N-term; iTRAQ4plex(K)@21		0.0	2500.32	626.09	2500.31	626.08	4	31.0	54.1	36.4	63.6	1.7
			92 VVDLMAYMASK	iTRAQ4plex@N-term; No iTRAQ4plex(K)@11		0.0	1370.72	686.37	1370.71	686.36	2	42.3	43.9	49.1	50.9	1.0
			91 VVDLMAYMASK	iTRAQ4plex@N-term; Lys->Gln@11		0.1	1370.74	686.38	1370.67	686.34	2	43.5	61.2	41.6	58.4	1.4
			87 VVDLMAYMASK	iTRAQ4plex@N-term; iTRAQ4plex(K)@11		0.0	1514.77	505.93	1514.81	505.94	3	28.1	31.5	47.2	52.8	1.1
			77 LTGMAFR	iTRAQ4plex@N-term		0.0	938.51	470.26	938.51	470.26	2	768.4	1203.8	39.0	61.0	1.6
			25 GAAQNIIPASTGAAK	iTRAQ4plex@N-term; Deamidated(N)@5; Lys->Gln@15		0.1	1513.87	757.94	1513.79	757.90	2					
			21 QASEGPLK	iTRAQ4plex@N-term; Lys->Gln@8		0.1	972.58	487.30	972.50	487.26	2					
															Average	1.3
															Stdev	0.3
IPI:IPI00850107.3	IGK-V	Immunoglobulin kappa chain complex variable regio	99 FSGSGSGTDFTLR	iTRAQ4plex@N-term		0.0	1474.71	738.36	1474.72	738.37	2	1518.1	1119.7	57.6	42.4	0.7
			99 FSGSGSGTDFTLR	iTRAQ4plex@N-term		0.0	1474.71	738.36	1474.72	738.37	2	1113.6	773.4	59.0	41.0	0.7
			99 MSNLASGVPDR	iTRAQ4plex@N-term	cleaved Q-M@N-term	0.0	1289.66	645.84	1289.65	645.83	2	636.5	708.4	47.3	52.7	1.1
			99 FSGSGSGTDFTLR	iTRAQ4plex@N-term; Arg->Gln@13		0.0	1446.67	724.34	1446.67	724.34	2	2854.7	1548.6	64.8	35.2	0.5
			99 FSGSGSGTDFTLR	iTRAQ4plex@N-term; Arg->Gln@13		0.0	1446.71	724.36	1446.67	724.34	2	1436.4	952.7	60.1	39.9	0.7
			99 FSGSGSGTDFTLR	iTRAQ4plex@N-term; Arg->Gln@13		0.0	1446.67	724.34	1446.67	724.34	2	1337.4	808.0	62.3	37.7	0.6
			99 FSGSGSGTDFTLR	iTRAQ4plex@N-term; Arg->Gln@13		0.0	1446.71	724.36	1446.67	724.34	2	590.9	403.7	59.4	40.6	0.7
			93 FSGSGSGTDFTLRISR	iTRAQ4plex@N-term; iTRAQ4plex(T)@11; Deamidated(R)@13; Dehydrated(S)@15	missed R-I@13	0.0	1958.03	653.69	1958.01	653.68	3	409.7	305.2	57.3	42.7	0.7
			60 LIYQMSNLASGVPDR	iTRAQ4plex@N-term; Gln->Tyr@4	cleaved L-L@N-term	0.0	1841.94	614.99	1841.95	614.99	3	36.9	40.8	47.5	52.5	1.1
			27 TFGGGTK	iTRAQ4plex@N-term; Lys->Gln@7	cleaved Y-T@N-term	0.1	810.48	406.25	810.40	406.21	2					
			14 GSGTDFTLR	iTRAQ4plex@N-term	cleaved S-G@N-term	0.0	1096.56	549.29	1096.56	549.29	2					
			13 FSGSGSGTDFTLR	iTRAQ4plex@N-term; Methyl(S)@2; iTRAQ4plex(T)@11		0.0	1632.83	545.28	1632.84	545.29	3					
			10 FSGSGSGTDFTLR	iTRAQ4plex@N-term; Methyl(S)@2; Arg->Gln@13		0.0	1460.68	731.35	1460.69	731.35	2					
			10 FSGSGSGTDFTLR	iTRAQ4plex@N-term; Methyl(S)@2; Arg->Gln@13		0.0	1460.69	731.35	1460.69	731.35	2					
			10 FSGSGSGTDFTLR	iTRAQ4plex@N-term; Methyl(S)@2; Arg->Gln@13		0.0	1460.68	731.35	1460.69	731.35	2					
			10 FSGSGSGTDFTLR	iTRAQ4plex@N-term; Methyl(S)@2; Arg->Gln@13		0.0	1460.68	731.35	1460.69	731.35	2					
			10 FSGSGSGTDFTLR	iTRAQ4plex@N-term; Methyl(S)@2; iTRAQ4plex(T)@11		0.0	1632.81	545.28	1632.84	545.29	3					
															Average	0.8
															Stdev	0.2
IPI:IPI00674805.3	13KDA PROTEIN	13 kDa protein	99 FMSTSVGDR	iTRAQ4plex@N-term		0.0	1142.52	572.27	1142.55	572.28	2	9237.1	7060.7	56.7	43.3	0.8
			99 LLIYWASTR	iTRAQ4plex@N-term		0.0	1265.71	633.86	1265.73	633.87	2	1320.5	1070.6	55.2	44.8	0.8
			82 LLIYWASTR	iTRAQ4plex@N-term		0.0	1265.69	633.85	1265.73	633.87	2	334.2	269.7	55.3	44.7	0.8
			63 LLIYWASTR	iTRAQ4plex@N-term; Oxidation(Y)@4		0.0	1281.70	641.86	1281.72	641.87	2	176.5	126.7	58.2	41.8	0.7
															Average	0.8
															Stdev	0.0
IPI:IPI00230192.5	GNAO1	Guanine nucleotide binding protein, alpha O	99 AMDTLGVEYGDKER	iTRAQ4plex@N-term; iTRAQ4plex(K)@12		0.0	1870.91	624.64	1870.93	624.65	3	324.0	296.2	52.2	47.8	0.9
			99 YYLDSLDR	iTRAQ4plex@N-term		0.0	1187.60	594.81	1187.59	594.80	2	893.7	1132.2	44.1	55.9	1.3
			99 LLLLGAGESGK	iTRAQ4plex@N-term; iTRAQ4plex(K)@11		0.0	1344.80	449.27	1344.82	449.28	3	86.1	97.4	46.9	53.1	1.1
			99 QYKPVVYSNTIQSLAAIVR	iTRAQ4plex@N-term; iTRAQ4plex(K)@3		0.0	2437.35	813.46	2437.39	813.47	3	15.6	24.4	39.0	61.0	1.6
			99 EYQLNDSAK	iTRAQ4plex@N-term; iTRAQ4plex(K)@9		0.0	1354.73	678.37	1354.70	678.36	2	123.9	165.4	42.8	57.2	1.3
			99 LLLLGAGESGK	iTRAQ4plex@N-term; Lys->Gln@11		0.0	1200.72	601.37	1200.68	601.35	2	480.8	462.6	51.0	49.0	1.0
			99 LLLLGAGESGK	iTRAQ4plex@N-term; Lys->Gln@11		0.0	1200.71	601.36	1200.68	601.35	2	393.2	505.2	43.8	56.2	1.3
			99 EYQLNDSAK	iTRAQ4plex@N-term; Lys->Gln@9		0.0	1210.59	606.30	1210.56	606.29	2	272.0	319.2	46.0	54.0	1.2
			14 STIVK	iTRAQ4plex@N-term; iTRAQ4plex(K)@5		0.0	834.54	418.28	834.54	418.28	2					
															Average	1.2
															Stdev	0.2
IPI:IPI00109910.4	IGHG	Immunoglobulin heavy chain (gamma polypeptide)	99 APQVYILPPPAEQLSR	iTRAQ4plex@N-term; Ser->Asp@15		0.0	1950.07	651.03	1950.07	651.03	3	25.4	56.3	31.1	68.9	2.2
			99 DLPSPIER	iTRAQ4plex@N-term		0.0	1069.58	535.80	1069.59	535.80	2	15617.9	13666.5	53.3	46.7	0.9
			99 DLPSPIER	iTRAQ4plex@N-term		0.0	1069.59	535.80	1069.59	535.80	2	906.3	788.1	53.5	46.5	0.9
			99 ILPPPAEQLSR	iTRAQ4plex@N-term	cleaved Y-I@N-term	0.0	1363.79	682.90	1363.79	682.90	2	498.7	823.0	37.7	62.3	1.7
			99 NTLFLQMTSLR	iTRAQ4plex@N-term		0.0	1466.82	734.42	1466.80	734.41	2	252.0	212.7	54.2	45.8	0.8
			99 APQVYILPPPAEQLSR	iTRAQ4plex@N-term		-0.1	1922.02	641.68	1922.07	641.70	3	162.7	154.0	51.4	48.6	0.9
			99 APQVYILPPPAEQLSR	iTRAQ4plex@N-term		0.0	1922.03	641.68	1922.07	641.70	3	136.2	129.2	51.3	48.7	0.9
			99 APQVYILPPPAEQLSR	iTRAQ4plex@N-term		0.0	1922.03	641.68	1922.07	641.70	3	47.8	43.5	52.4	47.6	0.9
			99 APQVYILPPPAEQLSR	iTRAQ4plex@N-term		0.0	1922.03	641.69	1922.07	641.70	3	33.6	32.3	51.0	49.0	1.0
			99 APQVYILPPPAEQLSR	iTRAQ4plex@N-term		0.0	1922.06	641.69	1922.07	641.70	3	17.5	21.1	45.4	54.6	1.2
			99 DVLMISLTPK	iTRAQ4plex@N-term; iTRAQ4plex(K)@10		0.0	1403.85	702.93	1403.83	702.92	2	320.3	496.3	39.2	60.8	1.5
			99 DTAPVLDSDGSYFIYSK	iTRAQ4plex@N-term; iTRAQ4plex(K)@17		0.0	2165.04	722.69	2165.08	722.70	3	66.8	125.4	34.7	65.3	1.9
			99 VVSALPIQHQDWMSGK	iTRAQ4plex@N-term; Lys->Gln@16		0.0	1939.00	647.34	1938.97	647.33	3	584.5	426.2	57.8	42.2	0.7
			99 APQVYILPPPAEQLSR	iTRAQ4plex@N-term; Oxidation(Y)@5		0.0	1938.04	647.02	1938.07	647.03	3	153.6	153.6	50.0	50.0	1.0
			98 NTLFLQMTSLR	iTRAQ4plex@N-term		0.0	1466.81	734.41	1466.80	734.41	2	205.7	178.5	53.5	46.5	0.9
			97 DVLMISLTPK	iTRAQ4plex@N-term; Lys->Gln@10		0.0	1259.70	630.86	1259.69	630.85	2	117.2	147.2	44.3	55.7	1.3
			96 DVLMISLTPK	iTRAQ4plex@N-term; Lys->Gln@10		0.0	1259.74	630.88	1259.69	630.85	2	200.8	263.1	43.3	56.7	1.3
			90 DVLMISLTPK	iTRAQ4plex@N-term; Lys->Gln@10		0.0	1259.73	630.87	1259.69	630.85	2	60.2	55.2	52.2	47.8	0.9
			79 APQVYILPPPAEQLSR	iTRAQ4plex@N-term; Cation:K(E)@12		0.0	1960.01	491.01	1960.03	491.01	4	6.9	10.6	39.6	60.4	1.5
			78 DVLMISLTPK	iTRAQ4plex@N-term; Lys->Gln@10		0.0	1259.73	630.87	1259.69	630.85	2	91.7	95.1	49.1	50.9	1.0
			45 APQVYILPPPAEQLSR	iTRAQ4plex@N-term; Ala->Phe@11		-0.1	1998.00	667.01	1998.11	667.04	3					
			30 SEDTAMYYCARELW	iTRAQ4plex@N-term; Pyridylethyl(C)@9; Deamidated(R)@11; Leu->Phe@13	cleaved W-L@C-term; missed R-E@11	0.0	2020.84	674.62	2020.85	674.62	3					
			20 APQVYILPPPAEQLSR	iTRAQ4plex@N-term; Pro->Gln@8		0.0	1953.11	652.04	1953.08	652.03	3					
			13 SEDTAMYYCARELW	iTRAQ4plex@N-term; Oxidation(M)@6; Pyridylethyl(C)@9; Deamidated(R)@11; Leu->Phe@13	cleaved W-L@C-term; missed R-E@11	0.0	2036.88	679.97	2036.84	679.95	3					
			13 SEDTAMYYCARELW	iTRAQ4plex@N-term; Pyridylethyl(C)@9; Deamidated(R)@11; Leu->Phe@13	cleaved W-L@C-term; missed R-E@11	0.0	2020.81	674.61	2020.85	674.62	3					
															Average	1.2
															Stdev	0.4
IPI:IPI00421014.8	ANTI-CARCINOI	Anti-carcinoma embryonic antigen light chain variable region	99 ASENIYSYLAWYQQK	iTRAQ4plex@N-term; iTRAQ4plex(K)@15		0.0	2151.13	718.05	2151.09	718.04	3	30.2	23.8	55.9	44.1	0.8
			99 ASENIYSYLAWYQQK	iTRAQ4plex@N-term; iTRAQ4plex(K)@15		0.0	2151.12	718.05	2151.09	718.04	3	15.8	19.0	45.4	54.6	1.2
			90 TLAEGVPSR	iTRAQ4plex@N-term		0.0	1072.60	537.31	1072.60	537.31	2	4926.8	3482.1	58.6	41.4	0.7
			49 FSGSGSGTQFSLK	iTRAQ4plex@N-term; Oxidation(F)@10; Lys->Gln@13		0.0	1461.74	731.87	1461.69	731.85	2					
															Average	0.9
															Stdev	0.3
IPI:IPI00117022.2	IGHG3	Immunoglobulin heavy constant gamma 3	99 AQTPQVYTIPPPR	iTRAQ4plex@N-term		0.0	1610.85	806.43	1610.89	806.45	2	1722.2	1345.1	56.1	43.9	0.8
			99 ALPAPIER	iTRAQ4plex@N-term		0.0	1009.60	505.81	1009.60	505.81	2	8905.6	5868.6	60.3	39.7	0.7
			99 VVSALPIQHQDWMR	iTRAQ4plex@N-term		0.0	1822.93	608.65	1822.96	608.66	3	2216.8	1478.6	60.0	40.0	0.7
			99 WNYGALSSGVR	iTRAQ4plex@N-term		0.0	1352.68	677.35	1352.70	677.36	2	1394.3	1603.8	46.5	53.5	1.2
			99 EVHTAWTQPR	iTRAQ4plex@N-term		0.0	1367.69	456.90	1367.71	456.91	3	1304.5	1056.7	55.2	44.8	0.8
			99 EVHTAWTQPR	iTRAQ4plex@N-term		1.0	1368.68	457.23	1367.71	456.91	3	1234.0	1032.1	54.5	45.5	0.8
			99 AQTPQVYTIPPPR	iTRAQ4plex@N-term		0.0	1610.84	537.95	1610.89	537.97	3	297.6	670.2	30.7	69.3	2.3
			99 WNYGALSSGVR	iTRAQ4plex@N-term; Deamidated(N)@2		0.0	1353.68	677.85	1353.68	677.85	2	3828.4	3543.5	51.9	48.1	0.9
			99 VVSALPIQHQDWMR	iTRAQ4plex@N-term; Deamidated(Q)@8; Deamidated(Q)@10		-0.1	1824.86	609.29	1824.93	609.32	3	3633.3	2526.9	59.0	41.0	0.7
			99 AQTPQVYTIPPPR	iTRAQ4plex@N-term; Dimethyl(R)@13		0.0	1638.93	547.32	1638.92	547.31	3	112.8	216.7	34.2	65.8	1.9
			99 GYFPEPVTVK	iTRAQ4plex@N-term; iTRAQ4plex(K)@10		0.0	1423.77	712.89	1423.80	712.91	2	1943.4	1947.0	50.0	50.0	1.0
			99 GYFPEPVTVK	iTRAQ4plex@N-term; Lys->Gln@10		0.0	1279.67	640.84	1279.66	640.84	2	901.6	763.4	54.2	45.8	0.8
			99 GYFPEPVTVK	iTRAQ4plex@N-term; Lys->Gln@10		0.0	1279.68	640.85	1279.66	640.84	2	655.3	596.0	52.4	47.6	0.9
			99 VVSALPIQHQDWMR	iTRAQ4plex@N-term; Oxidation(M)@13		0.0	1838.93	613.98	1838.96	613.99	3	2636.2	1621.4	61.9	38.1	0.6
			99 VVSALPIQHQDWMR	iTRAQ4plex@N-term; Oxidation(W)@12		0.0	1838.94	613.99	1838.96	613.99	3	227.1	145.1	61.0	39.0	0.6
			92 ATLTVDK	iTRAQ4plex@N-term; iTRAQ4plex(K)@7		0.0	1034.62	518.32	1034.62	518.32	2	10975.9	9891.4	52.6	47.4	0.9
			85 DALMISLTPK	iTRAQ4plex@N-term; Lys->Gln@10		0.0	1231.67	616.84	1231.66	616.84	2	250.3	160.6	60.9	39.1	0.6
			46 ALPAPIER	iTRAQ4plex@N-term		0.0	1009.58	505.80	1009.60	505.81	2					
			10 VVSALPIQHQDWMR	iTRAQ4plex@N-term		-10.1	1812.88	454.23	1822.96	456.75	4					
			10 ATLTVDK	iTRAQ4plex@N-term; Lys->Gln@7		0.1	890.54	446.28	890.48	446.25	2					
															Average	1.0
															Stdev	0.5
IPI:IPI00464406.2	IG KAPPA	Ig kappa chain V-III region PC 6308	99 ASNLESGIPAR	iTRAQ4plex@N-term	cleaved T-A@N-term	0.0	1257.68	629.85	1257.68	629.85	2	5349.0	3397.2	61.2	38.8	0.6
			99 DIVLTQSPASLAVSLGQR	iTRAQ4plex@N-term		0.0	1998.14	667.05	1998.12	667.05	3	42.1	50.5	45.4	54.6	1.2
			99 DIVLTQSPASLAVSLGQR	iTRAQ4plex@N-term		0.0	1998.10	667.04	1998.12	667.05	3	25.0	16.4	60.3	39.7	0.7
			99 DIVLTQSPASLAVSLGQR	iTRAQ4plex@N-term; Gln->Arg@17		0.0	2026.13	676.39	2026.17	676.40	3	8.6	16.0	34.9	65.1	1.9
			84 IVLTQSPASLAVSLGQR	iTRAQ4plex@N-term; Val->Cys@2; Pyridylethyl(C)@2; Deamidated(Q)@5	cleaved D-I@N-term	0.1	1993.15	665.39	1993.08	665.37	3	61.7	64.8	48.8	51.2	1.1
			62 ASNLESGIPAR	iTRAQ4plex@N-term	cleaved T-A@N-term	0.0	1257.70	629.85	1257.68	629.85	2	2131.6	1358.1	61.1	38.9	0.6
			12 DIVLTQSPASLAVSLGQR	iTRAQ4plex@N-term; Methyl(S)@10; Deamidated(Q)@17		-0.1	2013.06	672.03	2013.12	672.05	3					
															Average	1.0
															Stdev	0.5
IPI:IPI00471437.1	IGH-1A	Immunoglobulin heavy chain 1a (serum IgG2a	99 ALPAPIER	iTRAQ4plex@N-term		0.0	1009.60	505.81	1009.60	505.81	2	8905.6	5868.6	60.3	39.7	0.7
			99 NTEPVLDSDGSYFMYSK	iTRAQ4plex@N-term; Asp->Asn@9; Oxidation(M)@14; iTRAQ4plex(K)@17		0.0	2255.08	752.70	2255.07	752.70	3	153.1	116.7	56.8	43.2	0.8
			99 TELNYKNTEPVLDSDGSYFMYSK	iTRAQ4plex@N-term; Glu->Thr@2; iTRAQ4plex(K)@6; Asn->His@7; iTRAQ4plex(K)@23	missed K-N@6	0.0	3127.59	782.90	3127.55	782.90	4	322.7	332.3	49.3	50.7	1.0
			99 APQVYVLPPPEEEMTK	iTRAQ4plex@N-term; iTRAQ4plex(K)@16		0.0	2115.07	706.03	2115.12	706.05	3	181.1	201.2	47.4	52.6	1.1
			99 APQVYVLPPPEEEMTK	iTRAQ4plex@N-term; iTRAQ4plex(K)@16		0.0	2115.12	706.05	2115.12	706.05	3	94.1	77.7	54.8	45.2	0.8
			99 NTEPVLDSDGSYFMYSK	iTRAQ4plex@N-term; iTRAQ4plex(K)@17		-0.1	2240.00	747.67	2240.06	747.69	3	687.1	624.3	52.4	47.6	0.9
			99 NTEPVLDSDGSYFMYSK	iTRAQ4plex@N-term; iTRAQ4plex(K)@17		0.0	2240.01	747.68	2240.06	747.69	3	305.9	289.6	51.4	48.6	0.9
			99 NTEPVLDSDGSYFMYSK	iTRAQ4plex@N-term; iTRAQ4plex(K)@17		0.0	2240.03	747.68	2240.06	747.69	3	285.7	243.8	54.0	46.0	0.9
			99 NTEPVLDSDGSYFMYSK	iTRAQ4plex@N-term; iTRAQ4plex(K)@17		1.0	2241.01	748.01	2240.06	747.69	3	155.0	165.2	48.4	51.6	1.1
			99 APQVYVLPPPEEEMTK	iTRAQ4plex@N-term; Lys->Arg@16		0.0	1999.04	667.35	1999.02	667.35	3	36.5	51.7	41.4	58.6	1.4
			99 VVSALPIQHQDWMSGK	iTRAQ4plex@N-term; Lys->Gln@16		0.0	1939.00	647.34	1938.97	647.33	3	584.5	426.2	57.8	42.2	0.7
			99 NTEPVLDSDGSYFMYSK	iTRAQ4plex@N-term; Lys->Gln@17		0.0	2095.91	699.65	2095.92	699.65	3	96.6	61.4	61.1	38.9	0.6
			99 TTAPSVYPLAPVCGDTTGSSVTLGCLVK	iTRAQ4plex@N-term; Pyridylethyl(C)@13; Thr->Ser@17; Pyridylethyl(C)@25; Lys->Gln@28		0.0	3076.51	770.13	3076.54	770.14	4	56.5	56.3	50.1	49.9	1.0
			99 NTEPVLDSDGSYFMYSK	iTRAQ4plex@N-term; Ser->Asp@16; iTRAQ4plex(K)@17		0.0	2268.05	757.02	2268.05	757.02	3	31.2	46.5	40.2	59.8	1.5
			98 APQVYVLPPPEEEMTK	iTRAQ4plex@N-term; Lys->Arg@16		0.0	1999.06	667.36	1999.02	667.35	3	26.4	28.3	48.2	51.8	1.1
			92 APQVYVLPPPEEEMTK	iTRAQ4plex@N-term; Lys->Gln@16		0.0	1970.99	658.00	1970.98	658.00	3	12.4	11.5	51.9	48.1	0.9
			91 NTEPVLDSDGSYFMYSK	iTRAQ4plex@N-term; Amino(Y)@12; Oxidation(M)@14; iTRAQ4plex(K)@17		0.0	2271.09	758.04	2271.06	758.03	3	119.3	90.9	56.7	43.3	0.8
			76 NTEPVLDSDGSYFMYSK	iTRAQ4plex@N-term; iTRAQ4plex(S)@16; Lys->Arg@17		0.0	2268.07	757.03	2268.06	757.03	3	38.1	58.1	39.6	60.4	1.5
			59 APQVYVLPPPEEEMTKK	iTRAQ4plex@N-term; iTRAQ4plex(K)@16; Lys->Gln@17	missed K-K@16	0.0	2243.22	561.81	2243.18	561.80	4	95.0	53.7	63.9	36.1	0.6
			46 ALPAPIER	iTRAQ4plex@N-term		0.0	1009.58	505.80	1009.60	505.81	2					
			43 APQVYVLPPPEEEMTK	iTRAQ4plex@N-term; Oxidation(M)@14; Lys->Gln@16		0.0	1987.01	663.34	1986.97	663.33	3					
			39 TELNYK	iTRAQ4plex@N-term; iTRAQ4plex(K)@6		0.0	1054.61	528.31	1054.59	528.30	2					
															Average	1.0
															Stdev	0.3
IPI:IPI00124470.1	C1QC	Complement component 1, q subcomponent, C chain	99 FNSVVTNPQGH	iTRAQ4plex@N-term	cleaved H-Y@C-term	0.0	1342.70	672.36	1342.68	672.34	2	217.6	253.7	46.2	53.8	1.2
			99 QVSSGGALLR	iTRAQ4plex@N-term		0.0	1130.68	566.35	1130.65	566.33	2	7712.9	5407.8	58.8	41.2	0.7
			99 QTTQYPEANALVR	iTRAQ4plex@N-term		0.0	1633.82	817.92	1633.85	817.93	2	1307.1	736.4	64.0	36.0	0.6
			99 QTTQYPEANALVR	iTRAQ4plex@N-term		4.0	1637.85	546.96	1633.85	545.63	3	368.8	222.8	62.3	37.7	0.6
			99 QTTQYPEANALVR	iTRAQ4plex@N-term		0.0	1633.88	545.63	1633.85	545.63	3	333.2	196.1	63.0	37.0	0.6
			99 FNSVVTNPQGHYNPSTGK	iTRAQ4plex@N-term; No iTRAQ4plex(K)@18		0.0	2090.06	697.69	2090.03	697.68	3	545.9	684.6	44.4	55.6	1.3
			99 GPPGEPGVEGR	iTRAQ4plex@N-term; Oxidation(P)@3; Oxidation(P)@6; Glu->Asn@9; Carbamyl(R)@11		0.0	1254.64	628.33	1254.61	628.31	2	2132.2	4489.3	32.2	67.8	2.1
			99 GTSGLPGDPGPR	iTRAQ4plex@N-term; Oxidation(P)@6		0.0	1269.62	635.82	1269.64	635.83	2	11281.9	6638.7	63.0	37.0	0.6
			99 GPPGEPGVEGR	iTRAQ4plex@N-term; Oxidation(P)@6		0.1	1210.66	606.34	1210.61	606.31	2	3999.7	2364.6	62.8	37.2	0.6
			99 GTSGLPGDPGPR	iTRAQ4plex@N-term; Oxidation(P)@6		0.0	1269.67	635.84	1269.64	635.83	2	1794.9	1108.0	61.8	38.2	0.6
			99 GTSGLPGDPGPR	iTRAQ4plex@N-term; Oxidation(P)@6; Dimethyl(R)@12		0.0	1297.71	649.86	1297.67	649.84	2	1002.3	1865.2	35.0	65.0	1.9
			99 GTSGLPGDPGPR	iTRAQ4plex@N-term; Oxidation(P)@6; Oxidation(P)@9		0.0	1285.64	643.83	1285.64	643.83	2	4667.9	3006.5	60.8	39.2	0.6
			99 GTSGLPGDPGPR	iTRAQ4plex@N-term; Oxidation(P)@6; Oxidation(P)@9		0.0	1285.65	643.83	1285.64	643.83	2	3846.1	2561.3	60.0	40.0	0.7
			99 GTSGLPGDPGPR	iTRAQ4plex@N-term; Oxidation(P)@6; Oxidation(P)@9		0.0	1285.63	643.82	1285.64	643.83	2	2789.8	1974.3	58.6	41.4	0.7
			96 GPPGEPGVEGR	iTRAQ4plex@N-term; Oxidation(P)@2; Oxidation(P)@3; Oxidation(P)@6		0.1	1242.65	622.33	1242.60	622.31	2	82.5	46.8	63.8	36.2	0.6
			93 GTSGLPGDPGPR	iTRAQ4plex@N-term; Thr->Val@2; Oxidation(P)@9		0.0	1267.68	634.85	1267.66	634.84	2	8336.3	5224.0	61.5	38.5	0.6
			86 QTTQYPEANALVR	iTRAQ4plex@N-term; Deamidated(Q)@4; Oxidation(Y)@5		0.0	1650.86	826.44	1650.83	826.42	2	498.2	287.6	63.4	36.6	0.6
															Average	0.8
															Stdev	0.5
IPI:IPI00314109.9	KCNAB1	Potassium voltage-gated channel, shaker-related subfamily, beta member 1	99 WSAMEIMEAYSVAR	iTRAQ4plex@N-term		0.0	1786.88	596.63	1786.85	596.62	3	5.5	0.0	100.0	0.0	0.0
			99 AMTHVINQGMAMYWGTSR	iTRAQ4plex@N-term		-0.1	2196.99	733.34	2197.04	733.35	3	305.5	251.6	54.8	45.2	0.8
			99 AMTHVINQGMAMYWGTSR	iTRAQ4plex@N-term		0.0	2197.00	733.34	2197.04	733.35	3	36.9	40.8	47.5	52.5	1.1
			99 SSLVITTK	iTRAQ4plex@N-term; iTRAQ4plex(K)@8		0.0	1135.70	568.86	1135.71	568.86	2	3457.6	3373.3	50.6	49.4	1.0
			99 AMTHVINQGMAMYWGTSR	iTRAQ4plex@N-term; Ser->Asp@17		0.1	2225.09	742.70	2225.03	742.68	3	12.2	16.9	41.9	58.1	1.4
															Average	0.9
															Stdev	0.5
IPI:IPI00136310.1	MRPL23	Mitochondrial ribosomal protein L23	99 NVLYPLYQLGGPQLR	iTRAQ4plex@N-term		0.0	1874.01	625.68	1874.05	625.69	3	16.0	12.4	56.3	43.7	0.8
			99 SPEPLEEELPQQR	iTRAQ4plex@N-term		0.0	1694.83	565.95	1694.86	565.96	3	27.3	53.1	34.0	66.0	1.9
			99 NYLEQIYNVPVAAVR	iTRAQ4plex@N-term		0.0	1892.03	631.68	1892.03	631.68	3	12.7	3.9	76.6	23.4	0.3
			94 NYLEQIYNVPVAAVR	iTRAQ4plex@N-term		0.0	1892.05	631.69	1892.03	631.68	3	19.4	18.8	50.8	49.2	1.0
			68 NYLEQIYNVPVAAVR	iTRAQ4plex@N-term		0.0	1892.01	631.68	1892.03	631.68	3	31.9	28.1	53.2	46.8	0.9
															Average	1.0
															Stdev	0.6
IPI:IPI00132799.4	C1QBP	Complement component 1, q subcomponent binding protein	99 AEEQEPELTSTPNFVVEVTK	iTRAQ4plex@N-term; Lys->Gln@20		0.0	2390.14	797.72	2390.16	797.73	3	24.6	27.2	47.5	52.5	1.1
			99 EVSFQATGDSEWR	iTRAQ4plex@N-term		0.0	1654.75	828.38	1654.77	828.39	2	288.5	367.2	44.0	56.0	1.3
			99 AFVEFLTDEIK	iTRAQ4plex@N-term; iTRAQ4plex(K)@11		0.0	1598.89	800.45	1598.88	800.45	2	106.3	194.5	35.4	64.6	1.8
			99 AFVEFLTDEIK	iTRAQ4plex@N-term; iTRAQ4plex(K)@11		0.0	1598.88	533.97	1598.88	533.97	3	6.9	12.8	34.9	65.1	1.9
			99 AFVEFLTDEIKEEK	iTRAQ4plex@N-term; iTRAQ4plex(K)@11; Lys->Gln@14		0.1	1985.08	662.70	1985.02	662.68	3	131.8	149.9	46.8	53.2	1.1
			99 MSGDWELEVNGTEAK	iTRAQ4plex@N-term; iTRAQ4plex(K)@15		0.0	1952.90	651.98	1952.94	651.99	3	210.6	143.7	59.4	40.6	0.7
			99 MSGDWELEVNGTEAK	iTRAQ4plex@N-term; iTRAQ4plex(K)@15		0.0	1952.91	651.98	1952.94	651.99	3	177.9	140.5	55.9	44.1	0.8
			99 AEEQEPELTSTPNFVVEVTK	iTRAQ4plex@N-term; iTRAQ4plex(K)@20		0.0	2534.32	845.78	2534.30	845.77	3	95.2	99.2	49.0	51.0	1.0
			99 AEEQEPELTSTPNFVVEVTK	iTRAQ4plex@N-term; iTRAQ4plex(K)@20		-0.1	2534.24	845.75	2534.30	845.77	3	75.6	83.7	47.5	52.5	1.1
			99 AFVEFLTDEIK	iTRAQ4plex@N-term; Lys->Gln@11		0.1	1454.79	728.40	1454.74	728.38	2	171.4	266.6	39.1	60.9	1.6
			99 AFVEFLTDEIK	iTRAQ4plex@N-term; Lys->Gln@11		0.1	1454.80	728.41	1454.74	728.38	2	48.6	73.0	40.0	60.0	1.5
			99 MSGDWELEVNGTEAK	iTRAQ4plex@N-term; Lys->Gln@15		0.0	1808.81	905.41	1808.80	905.41	2	263.0	163.5	61.7	38.3	0.6
			99 MSGDWELEVNGTEAK	iTRAQ4plex@N-term; Lys->Gln@15		0.0	1808.85	603.96	1808.80	603.94	3	75.7	80.3	48.5	51.5	1.1
			98 MSGDWELEVNGTEAK	iTRAQ4plex@N-term; Lys->Gln@15		0.0	1808.85	603.96	1808.80	603.94	3	14.2	11.4	55.5	44.5	0.8
			94 AFVEFLTDEIKEEK	iTRAQ4plex@N-term; Lys->Gln@11; Lys->Gln@14		0.0	1840.94	614.65	1840.89	614.64	3	45.6	54.7	45.4	54.6	1.2
			91 AFVEFLTDEIKEEK	iTRAQ4plex@N-term; Lys->Gln@11; Lys->Arg@14		0.1	1868.98	624.00	1868.93	623.98	3	26.2	34.8	42.9	57.1	1.3
															Average	1.2
															Stdev	0.4
IPI:IPI00474225.1	IGH-1A	Immunoglobulin heavy chain 1a (serum IgG2a	99 ALPAPIER	iTRAQ4plex@N-term		0.0	1009.60	505.81	1009.60	505.81	2	8905.6	5868.6	60.3	39.7	0.7
			99 SSSTAYMELR	iTRAQ4plex@N-term		0.0	1287.63	644.82	1287.63	644.82	2	2219.5	1556.4	58.8	41.2	0.7
			99 SSSTAYMELR	iTRAQ4plex@N-term		0.0	1287.63	644.82	1287.63	644.82	2	837.4	568.5	59.6	40.4	0.7
			99 NTEPVLDSDGSYFMYSK	iTRAQ4plex@N-term; Asp->Asn@9; Oxidation(M)@14; iTRAQ4plex(K)@17		0.0	2255.08	752.70	2255.07	752.70	3	153.1	116.7	56.8	43.2	0.8
			99 TELNYKNTEPVLDSDGSYFMYSK	iTRAQ4plex@N-term; Glu->Thr@2; iTRAQ4plex(K)@6; Asn->His@7; iTRAQ4plex(K)@23	missed K-N@6	0.0	3127.59	782.90	3127.55	782.90	4	322.7	332.3	49.3	50.7	1.0

			99	APQVYVLPPPEEEMTK	iTRAQ4plex@N-term; iTRAQ4plex(K)@16		0.0	2115.07	706.03	2115.12	706.05	3	181.1	201.2	47.4	52.6	1.1
			99	APQVYVLPPPEEEMTK	iTRAQ4plex@N-term; iTRAQ4plex(K)@16		0.0	2115.12	706.05	2115.12	706.05	3	94.1	77.7	54.8	45.2	0.8
			99	NTEPVLDSDGSYFMYSK	iTRAQ4plex@N-term; iTRAQ4plex(K)@17		-0.1	2240.00	747.67	2240.06	747.69	3	687.1	624.3	52.4	47.6	0.9
			99	NTEPVLDSDGSYFMYSK	iTRAQ4plex@N-term; iTRAQ4plex(K)@17		0.0	2240.01	747.68	2240.06	747.69	3	305.9	289.6	51.4	48.6	0.9
			99	NTEPVLDSDGSYFMYSK	iTRAQ4plex@N-term; iTRAQ4plex(K)@17		0.0	2240.03	747.68	2240.06	747.69	3	285.7	243.8	54.0	46.0	0.9
			99	NTEPVLDSDGSYFMYSK	iTRAQ4plex@N-term; iTRAQ4plex(K)@17		1.0	2241.01	748.01	2240.06	747.69	3	155.0	165.2	48.4	51.6	1.1
			99	APQVYVLPPPEEEMTK	iTRAQ4plex@N-term; Lys->Arg@16		0.0	1999.04	667.35	1999.02	667.35	3	36.5	51.7	41.4	58.6	1.4
			99	VVSALPIQHQDWMSGK	iTRAQ4plex@N-term; Lys->Gln@16		0.0	1939.00	647.34	1938.97	647.33	3	584.5	426.2	57.8	42.2	0.7
			99	NTEPVLDSDGSYFMYSK	iTRAQ4plex@N-term; Lys->Gln@17		0.0	2095.91	699.65	2095.92	699.65	3	96.6	61.4	61.1	38.9	0.6
			99	TTAPSVYPLAPVCGDTTGSSVTLGCLVK	iTRAQ4plex@N-term; Pyridylethyl(C)@13; Thr->Ser@17; Pyridylethyl(C)@25; Lys->Gln@28		0.0	3076.51	770.13	3076.54	770.14	4	56.5	56.3	50.1	49.9	1.0
			99	NTEPVLDSDGSYFMYSK	iTRAQ4plex@N-term; Ser->Asp@16; iTRAQ4plex(K)@17		0.0	2268.05	757.02	2268.05	757.02	3	31.2	46.5	40.2	59.8	1.5
			98	APQVYVLPPPEEEMTK	iTRAQ4plex@N-term; Lys->Arg@16		0.0	1999.06	667.36	1999.02	667.35	3	26.4	28.3	48.2	51.8	1.1
			92	ATLTVDK	iTRAQ4plex@N-term; iTRAQ4plex(K)@7		0.0	1034.62	518.32	1034.62	518.32	2	10975.9	9891.4	52.6	47.4	0.9
			92	APQVYVLPPPEEEMTK	iTRAQ4plex@N-term; Lys->Gln@16		0.0	1970.99	658.00	1970.98	658.00	3	12.4	11.5	51.9	48.1	0.9
			91	NTEPVLDSDGSYFMYSK	iTRAQ4plex@N-term; Amino(Y)@12; Oxidation(M)@14; iTRAQ4plex(K)@17		0.0	2271.09	758.04	2271.06	758.03	3	119.3	90.9	56.7	43.3	0.8
			76	NTEPVLDSDGSYFMYSK	iTRAQ4plex@N-term; iTRAQ4plex(S)@16; Lys->Arg@17		0.0	2268.07	757.03	2268.06	757.03	3	38.1	58.1	39.6	60.4	1.5
			59	APQVYVLPPPEEEMTKK	iTRAQ4plex@N-term; iTRAQ4plex(K)@16; Lys->Gln@17	missed K-K@16	0.0	2243.22	561.81	2243.18	561.80	4	95.0	53.7	63.9	36.1	0.6
			46	ALPAPIER	iTRAQ4plex@N-term		0.0	1009.58	505.80	1009.60	505.81	2					
			43	APQVYVLPPPEEEMTK	iTRAQ4plex@N-term; Oxidation(M)@14; Lys->Gln@16		0.0	1987.01	663.34	1986.97	663.33	3					
			39	TELNYK	iTRAQ4plex@N-term; iTRAQ4plex(K)@6		0.0	1054.61	528.31	1054.59	528.30	2					
			10	ATLTVDK	iTRAQ4plex@N-term; Lys->Gln@7		0.1	890.54	446.28	890.48	446.25	2					
																**Average**	**0.9**
																**Stdev**	**0.3**
IPI:IPI00830623.1	FUS	Fusion, derived from t(12;16) malignant liposarcoma (human)	99	AAIDWFDGKEFSGNPIK	iTRAQ4plex@N-term; iTRAQ4plex(K)@9; Lys->Gln@17		0.0	2182.14	728.39	2182.09	728.37	3	41.4	18.0	69.7	30.3	0.4
			99	AAIDWFDGK	iTRAQ4plex@N-term; iTRAQ4plex(K)@9		0.0	1309.70	655.86	1309.69	655.85	2	875.9	765.9	53.3	46.7	0.9
			58	AAIDWFDGK	iTRAQ4plex@N-term; Lys->Gln@9		0.0	1165.59	583.80	1165.55	583.78	2	132.0	93.7	58.5	41.5	0.7
			19	GGSDRGGFNK	iTRAQ4plex@N-term; Carbamyl(R)@5; Oxidation(F)@8; iTRAQ4plex(K)@10	cleaved M-G@N-term; missed R-G@5	0.0	1340.70	671.36	1340.67	671.34	2					
																**Average**	**0.7**
																**Stdev**	**0.2**
IPI:IPI00828449.1	C1QA	Complement component 1, q subcomponent, alpha polypeptide	99	GLFQVLAGGTVLQLR	iTRAQ4plex@N-term		0.0	1715.01	858.51	1715.02	858.52	2	346.4	233.9	59.7	40.3	0.7
			99	GLFQVLAGGTVLQLR	iTRAQ4plex@N-term		0.0	1715.04	572.69	1715.02	572.68	3	9.0	5.1	63.8	36.2	0.6
			99	QNPMTLGNVVIFDK	iTRAQ4plex@N-term; iTRAQ4plex(K)@14		-9.3	1853.71	618.91	1863.02	622.01	3	289.7	232.9	55.4	44.6	0.8
			99	QNPMTLGNVVIFDK	iTRAQ4plex@N-term; iTRAQ4plex(K)@14		-9.3	1853.68	618.90	1863.02	622.01	3	250.5	155.6	61.7	38.3	0.6
			99	QNPMTLGNVVIFDK	iTRAQ4plex@N-term; iTRAQ4plex(K)@14		-0.1	1862.96	622.00	1863.02	622.01	3	34.7	52.9	39.6	60.4	1.5
			99	QNPMTLGNVVIFDK	iTRAQ4plex@N-term; iTRAQ4plex(K)@14		0.0	1862.98	622.00	1863.02	622.01	3	26.0	38.1	40.6	59.4	1.5
			99	QNPMTLGNVVIFDK	iTRAQ4plex@N-term; Lys->Arg@14		0.0	1746.92	874.47	1746.92	874.47	2	115.3	200.4	36.5	63.5	1.7
			99	DSLSFSNTNNK	iTRAQ4plex@N-term; Lys->Gln@11		0.0	1369.66	685.84	1369.62	685.82	2	3394.4	2154.8	61.2	38.8	0.6
			99	DSLSFSNTNNK	iTRAQ4plex@N-term; Lys->Gln@11		0.0	1369.66	685.84	1369.62	685.82	2	123.5	125.5	49.6	50.4	1.0
			99	QNPMTLGNVVIFDK	iTRAQ4plex@N-term; Lys->Gln@14		0.0	1718.93	860.47	1718.88	860.45	2	340.9	184.3	64.9	35.1	0.5
			99	QNPMTLGNVVIFDK	iTRAQ4plex@N-term; Lys->Gln@14		0.0	1718.88	860.45	1718.88	860.45	2	319.5	169.9	65.3	34.7	0.5
			99	QNPMTLGNVVIFDK	iTRAQ4plex@N-term; Lys->Gln@14		0.0	1718.92	573.98	1718.88	573.97	3	60.4	50.8	54.3	45.7	0.8
			99	QNPMTLGNVVIFDK	iTRAQ4plex@N-term; Lys->Gln@14		0.0	1718.89	573.97	1718.88	573.97	3	49.5	47.0	51.3	48.7	0.9
			99	GEPGAAGIR	iTRAQ4plex@N-term; Oxidation(P)@3		0.0	986.55	494.28	986.53	494.27	2	9047.8	5484.6	62.3	37.7	0.6
			99	GEPGAAGIR	iTRAQ4plex@N-term; Oxidation(P)@3		0.0	986.57	494.29	986.53	494.27	2	4842.6	2984.8	61.9	38.1	0.6
			99	GEPGAAGIR	iTRAQ4plex@N-term; Oxidation(P)@3		0.0	986.54	494.28	986.53	494.27	2	3103.0	2015.8	60.6	39.4	0.6
			99	GEPGAAGIR	iTRAQ4plex@N-term; Oxidation(P)@3		0.0	986.57	494.29	986.53	494.27	2	379.5	230.0	62.3	37.7	0.6
			99	GEPGAAGIR	iTRAQ4plex@N-term; Oxidation(P)@3		0.0	986.58	494.30	986.53	494.27	2	222.9	210.4	51.4	48.6	0.9
			98	QNPMTLGNVVIFDK	iTRAQ4plex@N-term; Deamidated(N)@2; Deamidated(N)@8; Lys->Gln@14		0.0	1720.89	574.64	1720.85	574.62	3	239.8	147.0	62.0	38.0	0.6
			92	QNPMTLGNVVIFDK	iTRAQ4plex@N-term; Deamidated(N)@2; Deamidated(N)@8; iTRAQ4plex(K)@14		0.0	1864.98	622.67	1864.98	622.67	3	1149.4	1093.6	51.2	48.8	1.0
			90	GNPGNIR	iTRAQ4plex@N-term		0.0	870.52	436.27	870.48	436.25	2	309.2	350.0	46.9	53.1	1.1
			71	QNPMTLGNVVIFDK	iTRAQ4plex@N-term; iTRAQ4plex(K)@14		-1.1	1861.96	621.66	1863.02	622.01	3	12.3	13.7	47.4	52.6	1.1
			63	GNPGNIR	iTRAQ4plex@N-term		2.0	872.52	437.27	870.48	436.25	2	273.0	341.6	44.4	55.6	1.3
			52	GLFQVLAGGTVLQLR	iTRAQ4plex@N-term; Leu->His@6; Dehydrated(T)@10		0.0	1720.96	574.66	1720.99	574.67	3	7.1	6.3	53.1	46.9	0.9
			34	QNPMTLGNVVIFDK	iTRAQ4plex@N-term; Deamidated(N)@8; Oxidation(F)@12; Asp->Gln@13; iTRAQ4plex(K)@14		0.0	1892.99	632.01	1893.03	632.02	3					
			24	QNPMTLGNVVIFDK	iTRAQ4plex@N-term; Methyl(I)@11; Methyl(D)@13; iTRAQ4plex(K)@14		0.0	1891.01	631.34	1891.05	631.36	3					
																**Average**	**0.9**
																**Stdev**	**0.3**
IPI:IPI00169755.4	IGKV4-57	Immunoglobulin kappa variable 4-57	99	FSGSGSGTSYSLTISR	iTRAQ4plex@N-term		0.0	1749.84	875.93	1749.87	875.94	2	1294.6	977.3	57.0	43.0	0.8
			99	FSGSGSGTSYSLTISR	iTRAQ4plex@N-term		0.0	1749.87	875.94	1749.87	875.94	2	830.1	618.8	57.3	42.7	0.7
			99	LWIYSTSNLASGVPAR	iTRAQ4plex@N-term		0.0	1878.00	627.01	1878.01	627.01	3	89.6	51.7	63.4	36.6	0.6
			99	LWIYSTSNLASGVPAR	iTRAQ4plex@N-term		0.0	1877.96	626.99	1878.01	627.01	3	44.8	25.4	63.9	36.1	0.6
			99	LWIYSTSNLASGVPAR	iTRAQ4plex@N-term		0.0	1877.98	627.00	1878.01	627.01	3	22.7	28.5	44.4	55.6	1.3
			98	LASGVPAR	iTRAQ4plex@N-term	cleaved N-L@N-term	0.0	913.57	457.79	913.55	457.78	2	3049.0	2192.2	58.2	41.8	0.7
			17	TSNLASGVPAR	iTRAQ4plex@N-term	cleaved S-T@N-term	0.0	1215.69	608.85	1215.67	608.84	2					
																**Average**	**0.8**
																**Stdev**	**0.3**
IPI:IPI00654010.2	MRPL9	Mitochondrial ribosomal protein L9	99	AGEATVK	iTRAQ4plex@N-term; iTRAQ4plex(K)@7		0.0	962.60	482.31	962.56	482.29	2	61.4	73.6	45.5	54.5	1.2
			99	WELNPEIVAR	iTRAQ4plex@N-term		0.0	1369.72	685.87	1369.75	685.88	2	569.6	436.7	56.6	43.4	0.8
			99	WELNPEIVAR	iTRAQ4plex@N-term		0.0	1369.72	685.87	1369.75	685.88	2	464.2	366.0	55.9	44.1	0.8
																**Average**	**0.9**
																**Stdev**	**0.2**
IPI:IPI00230707.6	YWHAG	tyrosine 3-monooxygenase/tryptophan 5-monooxygenase activation protein, gamma polypeptide	99	DSTLIMQLLR	iTRAQ4plex@N-term		0.0	1332.78	667.40	1332.76	667.39	2	214.3	344.1	38.4	61.6	1.6
			99	DSTLIMQLLR	iTRAQ4plex@N-term		2.0	1334.75	668.38	1332.76	667.39	2	84.6	139.5	37.7	62.3	1.6
			99	NVTELNEPLSNEER	iTRAQ4plex@N-term		0.0	1786.86	596.63	1786.88	596.63	3	17.6	17.6	50.1	49.9	1.0
			96	LAEQAER	iTRAQ4plex@N-term		0.0	959.55	480.78	959.52	480.77	2	619.4	474.9	56.6	43.4	0.8
			93	LAEQAER	iTRAQ4plex@N-term		0.0	959.56	480.79	959.52	480.77	2	434.4	281.3	60.7	39.3	0.6
			33	DSTLIMQLLR	iTRAQ4plex@N-term; Gln->Arg@7		0.0	1360.76	681.39	1360.80	681.41	2					
																**Average**	**1.1**
																**Stdev**	**0.5**
IPI:IPI00130535.1	DBT	Dihydrolipoamide branched chain transacylase E2	99	AQIMNVSWSADHR	iTRAQ4plex@N-term		0.0	1657.78	553.60	1657.81	553.61	3	204.2	170.6	54.5	45.5	0.8
			99	TLATPAVR	iTRAQ4plex@N-term		0.0	971.62	486.82	971.59	486.80	2	5427.7	3746.7	59.2	40.8	0.7
			99	SYLENPAFMLLDLK	iTRAQ4plex@N-term; iTRAQ4plex(K)@14		0.0	1941.10	648.04	1941.05	648.02	3	14.3	8.1	63.8	36.2	0.6
			99	SYLENPAFMLLDLK	iTRAQ4plex@N-term; Oxidation(M)@9; iTRAQ4plex(K)@14		0.1	1957.11	653.38	1957.05	653.36	3	5.3	6.3	45.4	54.6	1.2
			98	LSFMPFFLK	iTRAQ4plex@N-term; iTRAQ4plex(K)@9		3.0	1419.82	474.28	1416.81	473.28	3	23.1	19.8	53.9	46.1	0.9
			95	TMSAALK	iTRAQ4plex@N-term; iTRAQ4plex(K)@7		0.0	1008.58	505.30	1008.59	505.30	2	2924.2	2851.9	50.6	49.4	1.0
			94	LSFMPFFLK	iTRAQ4plex@N-term; iTRAQ4plex(K)@9		0.0	1416.80	473.28	1416.81	473.28	3	14.2	12.5	53.2	46.8	0.9
			85	LSFMPFFLK	iTRAQ4plex@N-term; Lys->Gln@9		0.0	1272.70	637.36	1272.67	637.34	2	71.9	34.1	67.8	32.2	0.5
			62	LSFMPFFLK	iTRAQ4plex@N-term; Lys->Gln@9		0.1	1272.74	637.38	1272.67	637.34	2	62.5	41.1	60.4	39.6	0.7
																**Average**	**0.8**
																**Stdev**	**0.2**
IPI:IPI00121736.1	PDCD6	Programmed cell death protein 6	99	LSDQFHDILIR	iTRAQ4plex@N-term		0.0	1499.78	500.93	1499.82	500.95	3	104.4	97.7	51.6	48.4	0.9
			99	YITDWQNVFR	iTRAQ4plex@N-term		0.0	1484.76	743.39	1484.75	743.38	2	488.8	442.2	52.5	47.5	0.9
			99	YITDWQNVFR	iTRAQ4plex@N-term		0.0	1484.79	743.40	1484.75	743.38	2	488.7	447.3	52.2	47.8	0.9
			99	SIISMFDR	iTRAQ4plex@N-term		0.0	1111.58	556.80	1111.58	556.80	2	374.5	465.9	44.6	55.4	1.2
			98	YITDWQNVFR	iTRAQ4plex@N-term		0.0	1484.77	743.39	1484.75	743.38	2	317.2	282.6	52.9	47.1	0.9
			95	AGVNFSEFTGVWK	iTRAQ4plex@N-term; iTRAQ4plex(K)@13		0.9	1729.85	577.63	1728.91	577.31	3	23.9	45.8	34.3	65.7	1.9
																**Average**	**1.1**
																**Stdev**	**0.4**
IPI:IPI00170093.3	NDUFS8	NADH dehydrogenase (ubiquinone) Fe-S protein 8	99	GLGMTLSYLFR	iTRAQ4plex@N-term		0.0	1400.76	701.39	1400.76	701.39	2	70.9	62.3	53.2	46.8	0.9
			99	ILMWTELIR	iTRAQ4plex@N-term		0.0	1317.79	659.90	1317.76	659.89	2	140.1	120.5	53.8	46.2	0.9
			99	ILMWTELIR	iTRAQ4plex@N-term		0.0	1317.76	659.89	1317.76	659.89	2	121.7	125.5	49.2	50.8	1.0
			14	GLGMTLSYLFR	iTRAQ4plex@N-term		0.0	1400.77	701.39	1400.76	701.39	2					
																**Average**	**0.9**
																**Stdev**	**0.1**
IPI:IPI00221791.5	SIRPA	Signal-regulatory protein alpha	99	LLIYSFTGEHFPR	iTRAQ4plex@N-term		0.0	1722.92	575.31	1722.92	575.31	3	50.1	81.5	38.1	61.9	1.6
			99	NNMDFSIR	iTRAQ4plex@N-term		0.0	1139.54	570.78	1139.55	570.78	2	2753.9	4203.0	39.6	60.4	1.5
			99	VPRPEDTLTYADLDMVHLSR	iTRAQ4plex@N-term		0.0	2471.22	618.81	2471.26	618.82	4	26.0	40.3	39.2	60.8	1.6
																**Average**	**1.6**
																**Stdev**	**0.1**
IPI:IPI00341282.2	ATP5F1	ATP synthase, H+ transporting, mitochondrial F0 complex, subunit B1	99	QIQDAIDMEK	iTRAQ4plex@N-term; Lys->Gln@10		0.1	1333.68	667.85	1333.63	667.82	2	1103.1	860.9	56.2	43.8	0.8
			99	SISVQQEK	iTRAQ4plex@N-term; iTRAQ4plex(K)@8		0.0	1205.72	603.87	1205.69	603.85	2	1418.4	1394.0	50.4	49.6	1.0
			99	QIQDAIDMEK	iTRAQ4plex@N-term; Lys->Gln@10		0.0	1333.64	667.83	1333.63	667.82	2	594.4	508.1	53.9	46.1	0.9
			36	SISVQQEK	iTRAQ4plex@N-term; Lys->Gln@8		0.1	1061.60	531.81	1061.55	531.78	2					
																**Average**	**0.9**
																**Stdev**	**0.1**
IPI:IPI00309214.1	APCS	Serum amyloid P-component	99	APPSIVLGQEQDNYGGGFQR	iTRAQ4plex@N-term		0.0	2276.09	759.70	2276.13	759.72	3	182.2	120.5	60.2	39.8	0.7
			99	APPSIVLGQEQDNYGGGFQR	iTRAQ4plex@N-term		-0.1	2276.06	759.69	2276.13	759.72	3	89.2	61.5	59.2	40.8	0.7
			98	DNELLIYK	iTRAQ4plex@N-term; iTRAQ4plex(K)@8		0.0	1294.71	648.36	1294.74	648.38	2	1147.7	941.7	54.9	45.1	0.8
			95	TYSDLSR	iTRAQ4plex@N-term		0.0	984.52	493.27	984.50	493.26	2	4384.1	2810.6	60.9	39.1	0.6
																**Average**	**0.7**
																**Stdev**	**0.1**
IPI:IPI00749872.1	SLC1A2	Solute carrier family 1 (glial high affinity glutamate transporter), member 2	99	KNDEVSSLDAFLDLIR	iTRAQ4plex@N-term; Lys->Gln@1	missed K-N@1	0.1	1978.08	660.37	1978.01	660.34	3	10.6	11.5	47.8	52.2	1.1
			99	SELDTIDSQHR	iTRAQ4plex@N-term		0.0	1443.72	482.25	1443.71	482.24	3	400.5	657.0	37.9	62.1	1.6
			99	MQEDIEMTK	iTRAQ4plex@N-term; iTRAQ4plex(K)@9		0.0	1411.67	706.84	1411.69	706.85	2	3230.8	5521.8	36.9	63.1	1.7
			99	MQEDIEMTK	iTRAQ4plex@N-term; iTRAQ4plex(K)@9		0.0	1411.74	706.88	1411.69	706.85	2	176.0	242.6	42.0	58.0	1.4
			99	KNDEVSSLDAFLDLIR	iTRAQ4plex@N-term; Lys->Gln@1	missed K-N@1	0.1	1978.07	660.36	1978.01	660.34	3	1.7	3.3	34.3	65.7	1.9
			99	MQEDIEMTK	iTRAQ4plex@N-term; Lys->Gln@9		0.0	1267.60	634.81	1267.55	634.78	2	1315.9	1395.9	48.5	51.5	1.1
			97	TQSIYDDK	iTRAQ4plex@N-term; Lys->Gln@8		0.0	1112.56	557.29	1112.51	557.26	2	298.1	603.0	33.1	66.9	2.0
			67	NLFPENLVQACFQQIQTVTK	iTRAQ4plex@N-term; Pyridylethyl(C)@11; Lys->Gln@20		0.1	2569.42	857.48	2569.31	857.44	3	15.5	27.6	35.9	64.1	1.8
			11	MQEDIEMTK	iTRAQ4plex@N-term; Lys->Arg@9		0.0	1295.60	648.81	1295.60	648.81	2					
																**Average**	**1.6**
																**Stdev**	**0.4**
IPI:IPI00608050.1	ICT1	Immature colon carcinoma transcript 1	99	FHLASADWIEEPVR	iTRAQ4plex@N-term		0.0	1812.92	605.31	1812.93	605.32	3	149.9	103.9	59.1	40.9	0.7
			99	QASSYIPLDR	iTRAQ4plex@N-term		0.0	1292.67	647.34	1292.68	647.35	2	973.3	805.9	54.7	45.3	0.8
			99	QASSYIPLDR	iTRAQ4plex@N-term		0.0	1292.70	647.36	1292.68	647.35	2	956.6	712.5	57.3	42.7	0.7
																**Average**	**0.8**
																**Stdev**	**0.1**
IPI:IPI00120793.1	PRNP	Prion protein	99	HVAGAAAAG	iTRAQ4plex@N-term	cleaved A-V@C-term	0.1	938.59	470.30	938.51	470.26	2	5.3	6.3	45.4	54.6	1.2
			99	YPGQGSPGGNR	iTRAQ4plex@N-term		0.1	1232.68	617.35	1232.60	617.31	2	915.8	876.6	51.1	48.9	1.0
			99	HVAGAAAAGAVVGGLGGYMLGSAMS	iTRAQ4plex@N-term	cleaved R-P@C-term	0.0	2474.29	825.77	2474.26	825.76	3	20.8	32.9	38.7	61.3	1.6
			11	ENMYR	iTRAQ4plex@N-term		0.0	855.44	428.73	855.40	428.71	2					
																**Average**	**1.2**
																**Stdev**	**0.3**
IPI:IPI00134809.2	DLST	Dihydrolipoamide S-succinyltransferase (E2 component of 2-oxo-glutarate complex)	99	ASAFALQEQPVVNAVIDDATK	iTRAQ4plex@N-term; Lys->Gln@21		0.0	2330.21	777.74	2330.19	777.74	3					
			99	NVETMNYADIER	iTRAQ4plex@N-term		0.0	1597.72	799.87	1597.75	799.88	2	1861.5	1186.5	61.1	38.9	0.6
			99	VEGGTPLFTLR	iTRAQ4plex@N-term		0.0	1332.76	667.39	1332.75	667.38	2	1032.4	1842.1	35.9	64.1	1.8
			99	NVETMNYADIER	iTRAQ4plex@N-term		0.0	1597.72	799.87	1597.75	799.88	2	477.0	314.5	60.3	39.7	0.7
			99	VEGGTPLFTLR	iTRAQ4plex@N-term		0.0	1332.72	667.37	1332.75	667.38	2	430.8	728.9	37.1	62.9	1.7
			99	EAQNTCAMLTTFNEVDMSNIQEMR	iTRAQ4plex@N-term; Deamidated(N)@4; Pyridylethyl(C)@6; Dehydrated(T)@10; Oxidation(F)@12; Deamidated(N)@13		0.0	3024.34	757.09	3024.31	757.08	4	8.7	11.6	42.9	57.1	1.3
			99	LGFMSAFVK	iTRAQ4plex@N-term; iTRAQ4plex(K)@9		0.0	1286.73	429.92	1286.73	429.92	3	26.7	20.7	56.3	43.7	0.8
			97	LGFMSAFVK	iTRAQ4plex@N-term; Lys->Gln@9		0.0	1142.61	572.31	1142.59	572.30	2	87.3	65.1	57.3	42.7	0.7
			96	TINELGEK	iTRAQ4plex@N-term; Lys->Gln@8		0.1	1046.61	524.31	1046.54	524.28	2	663.1	582.0	53.3	46.7	0.9
			92	GLVVPVIR	iTRAQ4plex@N-term		0.0	995.67	498.84	995.66	498.84	2	499.9	442.2	53.1	46.9	0.9
			67	AAVEDPR	iTRAQ4plex@N-term		0.0	900.52	451.27	900.48	451.25	2	14.3	10.3	58.0	42.0	0.7
			27	AAVEDPR	iTRAQ4plex@N-term		0.0	900.52	451.27	900.48	451.25	2					
			27	AAVEDPR	iTRAQ4plex@N-term		0.0	900.52	451.27	900.48	451.25	2					
			27	AAVEDPR	iTRAQ4plex@N-term		0.0	900.52	451.27	900.48	451.25	2					
			13	AAVEDPR	iTRAQ4plex@N-term; Deamidated(R)@7		0.1	901.54	451.78	901.46	451.74	2					
																**Average**	**1.0**
																**Stdev**	**0.4**
IPI:IPI00119220.1	SNRPD2	Small nuclear ribonucleoprotein D2	99	EEEEFNTGPLSVLTQSVK	iTRAQ4plex@N-term; Lys->Gln@18		0.1	2150.12	717.72	2150.05	717.69	3	7.2	3.0	70.7	29.3	0.4
			99	GDSVIVVLR	iTRAQ4plex@N-term		0.0	1100.66	551.34	1100.67	551.34	2	334.4	212.6	61.1	38.9	0.6
			99	EEEEFNTGPLSVLTQSVK	iTRAQ4plex@N-term; iTRAQ4plex(K)@18		0.1	2294.24	765.75	2294.19	765.74	3	19.7	12.3	61.5	38.5	0.6
			94	EEEEFNTGPLSVLTQSVK	iTRAQ4plex@N-term; iTRAQ4plex(K)@18		0.1	2294.27	765.76	2294.19	765.74	3	14.4	7.1	67.1	32.9	0.5
																**Average**	**0.5**
																**Stdev**	**0.1**
IPI:IPI00111856.1	FCGR1	Fc receptor, IgG, high affinity I	99	ELFTTPVLR	iTRAQ4plex@N-term		0.0	1218.72	610.37	1218.71	610.36	2	3352.3	1802.3	65.0	35.0	0.5
			99	LVYNVVFYR	iTRAQ4plex@N-term		0.0	1315.74	658.88	1315.74	658.88	2	1278.2	825.5	60.8	39.2	0.6
			99	LVYNVVFYR	iTRAQ4plex@N-term		0.0	1315.74	658.88	1315.74	658.88	2	1129.4	691.9	62.0	38.0	0.6
			99	VLTEGEPLALR	iTRAQ4plex@N-term		0.0	1340.76	671.39	1340.78	671.40	2	857.4	573.0	59.9	40.1	0.7
			99	VLTEGEPLALR	iTRAQ4plex@N-term		0.0	1340.76	671.39	1340.78	671.40	2	751.7	511.0	59.5	40.5	0.7
			99	LVYNVVFYR	iTRAQ4plex@N-term		0.0	1315.74	658.88	1315.74	658.88	2	691.8	446.2	60.8	39.2	0.6
			99	VLTEGEPLALR	iTRAQ4plex@N-term		1.0	1341.78	671.90	1340.78	671.40	2	501.8	389.1	56.3	43.7	0.8
			99	VLTEGEPLALR	iTRAQ4plex@N-term		-1.0	1339.73	670.87	1340.78	671.40	2	441.6	383.1	53.5	46.5	0.9
			99	SDGVYEEVTATASQTTPK	iTRAQ4plex@N-term; iTRAQ4plex(K)@18		0.0	2171.08	724.70	2171.08	724.70	3	83.5	69.5	54.6	45.4	0.8
			99	SDGVYEEVTATASQTTPK	iTRAQ4plex@N-term; iTRAQ4plex(K)@18		-0.1	2171.03	724.68	2171.08	724.70	3	81.7	67.4	54.8	45.2	0.8
			99	SDGVYEEVTATASQTTPK	iTRAQ4plex@N-term; Lys->Arg@18		0.0	2055.01	686.01	2054.99	686.00	3	5.1	10.7	32.4	67.6	2.1
			99	SDGVYEEVTATASQTTPK	iTRAQ4plex@N-term; Lys->Gln@18		0.0	2026.96	676.66	2026.95	676.66	3	28.3	26.1	52.1	47.9	0.9
			98	ELFTTPVLR	iTRAQ4plex@N-term		0.0	1218.72	610.37	1218.71	610.36	2	2128.0	1206.0	63.8	36.2	0.6
			98	ELFTTPVLR	iTRAQ4plex@N-term		0.0	1218.68	610.35	1218.71	610.36	2	1543.4	880.1	63.7	36.3	0.6
			98	ELFTTPVLR	iTRAQ4plex@N-term		0.0	1218.70	610.36	1218.71	610.36	2	1010.4	592.1	63.1	36.9	0.6
			95	ANSFQQVR	iTRAQ4plex@N-term		0.0	1092.62	547.32	1092.58	547.30	2	2013.9	2673.5	43.0	57.0	1.3
			76	EAPDGPR	iTRAQ4plex@N-term		0.1	884.51	443.26	884.45	443.23	2	39.0	33.2	54.1	45.9	0.8
			69	LVYNVVFYR	iTRAQ4plex@N-term; Dimethyl(R)@9		0.0	1343.73	672.87	1343.77	672.89	2	208.4	454.0	31.5	68.5	2.2
			67	EAPDGPR	iTRAQ4plex@N-term		0.1	884.53	443.27	884.45	443.23	2	31.9	27.0	54.2	45.8	0.8
			17	EAPDGPR	iTRAQ4plex@N-term		0.0	884.49	443.25	884.45	443.23	2					
																**Average**	**0.9**
																**Stdev**	**0.5**
IPI:IPI00338334.4	KCNAB3	Potassium voltage-gated channel, shaker-related subfamily, beta member 3	99	IGVGSVTWSPLACGLITSK	iTRAQ4plex@N-term; Pyridylethyl(C)@13; Lys->Gln@19		0.0	2137.18	713.40	2137.14	713.39	3	28.8	13.0	68.8	31.2	0.5
			99	WSAAEIMEAYSMAR	iTRAQ4plex@N-term		0.0	1758.86	587.30	1758.82	587.28	3	5.2	8.5	37.9	62.1	1.6
			96	VMDLLPTAR	iTRAQ4plex@N-term		1.0	1159.65	580.83	1158.66	580.33	2	614.2	418.9	59.5	40.5	0.7
			30	GYQWLK	iTRAQ4plex@N-term; iTRAQ4plex(K)@6		0.0	1081.62	541.82	1081.62	541.82	2					
																**Average**	**0.9**
																**Stdev**	**0.6**
IPI:IPI00378520.4	MRPL41	Mitochondrial ribosomal protein L41	99	EGTFDANNLEK	iTRAQ4plex@N-term; Lys->Gln@11		0.1	1380.69	691.35	1380.63	691.32	2	160.7	108.8	59.6	40.4	0.7
			99	EMVPEFVVPDLTGFK	iTRAQ4plex@N-term; iTRAQ4plex(K)@15		0.0	1995.09	666.04	1995.06	666.03	3	14.1	14.7	49.0	51.0	1.0
			46	EMVPEFVVPDLTGFK	iTRAQ4plex@N-term; iTRAQ4plex(K)@15		0.0	1995.07	666.03	1995.06	666.03	3					
																**Average**	**0.9**
																**Stdev**	**0.3**
IPI:IPI00653598.2	UQCRC1	Ubiquinol-cytochrome c reductase core protein 1	99	EVESIGAHLNAYSTR	iTRAQ4plex@N-term		0.0	1789.88	597.63	1789.91	597.64	3	97.3	90.4	51.8	48.2	0.9
			99	VVELLADIVQNSSLEDSQIEK	iTRAQ4plex@N-term; iTRAQ4plex(K)@21		0.0	2616.38	873.13	2616.41	873.14	3	14.3	10.3	58.0	42.0	0.7
			99	MVLAAAGGVEHQQLLDLAQK	iTRAQ4plex@N-term; Lys->Gln@20		0.0	2235.22	746.08	2235.18	746.07	3	74.8	56.8	56.8	43.2	0.8
			93	SGMFWLR	iTRAQ4plex@N-term		0.0	1039.53	520.77	1039.54	520.78	2	682.5	552.6	55.3	44.7	0.8
			11	SGMFWLR	iTRAQ4plex@N-term; Dimethyl(R)@7		0.0	1067.55	534.78	1067.57	534.79	2					
																**Average**	**0.8**
																**Stdev**	**0.1**
IPI:IPI00845802.1	HBA-A1	Hemoglobin alpha, adult chain 1	99	IGGHGAEYGAEALER	iTRAQ4plex@N-term; Gly->Val@9		0.0	1714.85	572.62	1714.88	572.63	3	54.9	50.9	51.9	48.1	0.9
			99	MFASFPTTK	iTRAQ4plex@N-term; iTRAQ4plex(K)@9		0.0	1316.70	659.36	1316.70	659.36	2	1691.0	997.0	62.9	37.1	0.6
																**Average**	**0.8**
																**Stdev**	**0.2**
IPI:IPI00320420.3	CLU	Clusterin	99	ASGIIDTLFQDR	iTRAQ4plex@N-term		0.0	1478.77	740.39	1478.79	740.40	2	438.2	375.7	53.8	46.2	0.9
			99	ASGIIDTLFQDR	iTRAQ4plex@N-term		0.0	1478.79	740.40	1478.79	740.40	2	440.2	372.4	54.2	45.8	0.8
			99	QQSQVLDAMQDSFAR	iTRAQ4plex@N-term		0.0	1866.89	623.30	1866.90	623.31	3	95.4	43.0	69.0	31.0	0.5
			99	EQEVSDNELQELSTQGSR	iTRAQ4plex@N-term	cleaved G-E@N-term	0.0	2192.03	731.68	2192.03	731.68	3	46.2	36.3	56.1	43.9	0.8
			99	QQSQVLDAMQDSFAR	iTRAQ4plex@N-term		0.0	1866.90	623.31	1866.90	623.31	3	44.5	33.1	57.4	42.6	0.7
			37	DSEMK	iTRAQ4plex@N-term; iTRAQ4plex(K)@5		0.1	896.51	449.26	896.45	449.23	2					
																**Average**	**0.7**
																**Stdev**	**0.2**
IPI:IPI00828516.1	RPL5	Ribosomal protein L5	99	EFNAEVHR	iTRAQ4plex@N-term		0.0	1144.57	382.53	1144.57	382.53	3	256.5	292.5	46.7	53.3	1.1
			99	VGLTNYAAAYCTGLLLAR	iTRAQ4plex@N-term; Pyridylethyl(C)@11		0.0	2118.15	707.06	2118.14	707.05	3	35.4	33.4	51.4	48.6	0.9
			96	QFSQYIK	iTRAQ4plex@N-term; iTRAQ4plex(K)@7		0.0	1200.71	601.36	1200.67	601.34	2	2824.5	1525.9	64.9	35.1	0.5
			48	HIMGQNVADYMR	iTRAQ4plex@N-term		0.0	1577.79	526.94	1577.76	526.93	3					
																**Average**	**0.9**
																**Stdev**	**0.3**
IPI:IPI00132728.2	CYC1	Cytochrome c1	99	AANNGALPPDLSYIVR	iTRAQ4plex@N-term		0.0	1813.96	605.66	1813.98	605.67	3	46.2	37.2	55.4	44.6	0.8
			99	GLLSSLDHTSIR	iTRAQ4plex@N-term		0.0	1441.78	481.60	1441.80	481.61	3	359.3	332.5	51.9	48.1	0.9
			99	LSDYFPKPYPNPEAAR	iTRAQ4plex@N-term; iTRAQ4plex(K)@7		0.0	2152.14	718.39	2152.12	718.38	3	336.0	217.0	60.8	39.2	0.6
			93	DVATFLR	iTRAQ4plex@N-term		0.0	964.53	483.27	964.55	483.28	2	1939.4	2010.1	49.1	50.9	1.0
			77	AANNGALPPDLSYIVR	iTRAQ4plex@N-term		0.0	1813.97	605.66	1813.98	605.67	3	32.2	19.4	62.4	37.6	0.6
																**Average**	**0.8**
																**Stdev**	**0.2**
IPI:IPI00331555.2	BCKDHA	Branched chain ketoacid dehydrogenase E1, alpha polypeptide	99	AVAENQPFLIEAMTYR	iTRAQ4plex@N-term		0.0	1995.99	666.34	1996.02	666.35	3	10.7	8.3	56.3	43.7	0.8
			99	AVAENQPFLIEAMTYR	iTRAQ4plex@N-term		0.0	1995.99	666.34	1996.02	666.35	3	12.5	9.3	57.3	42.7	0.7
			41	LEFIQPNVISGIPIYR	iTRAQ4plex@N-term		-1.1	2001.06	668.03	2002.14	668.39	3					
																**Average**	**0.8**
																**Stdev**	**0.0**
IPI:IPI00123349.2	SEC23A	SEC23A (S. cerevisiae)	99	AETEEGPDVLR	iTRAQ4plex@N-term		0.0	1358.70	680.36	1358.68	680.35	2	141.6	130.0	52.1	47.9	0.9
			99	SPFLQVFNNSPDESSYYR	iTRAQ4plex@N-term		0.0	2293.12	765.38	2293.08	765.37	3	97.2	143.7	40.4	59.6	1.5
			99	SPFLQVFNNSPDESSYYR	iTRAQ4plex@N-term		0.0	2293.11	765.38	2293.08	765.37	3	81.5	173.8	31.9	68.1	2.1
			99	IDMNLTDLLGELQR	iTRAQ4plex@N-term		-1.0	1772.93	887.47	1773.94	887.98	2	42.2	46.2	47.8	52.2	1.1
			99	MVVPVAALFTPLK	iTRAQ4plex@N-term; Lys->Gln@13		0.1	1528.94	765.48	1528.88	765.45	2	183.8	128.6	58.8	41.2	0.7
			99	MVVPVAALFTPLK	iTRAQ4plex@N-term; Lys->Gln@13		0.1	1528.93	765.47	1528.88	765.45	2	93.1	56.0	62.4	37.6	0.6
			33	DLSAK	iTRAQ4plex@N-term; iTRAQ4plex(K)@5		0.0	820.51	411.26	820.49	411.25	2					
			15	IMMFIGGPATQGPGMVVGDELK	iTRAQ4plex@N-term; Lys->Gln@22		0.2	2391.33	798.12	2391.18	798.07	3					
																**Average**	**1.2**
																**Stdev**	**0.6**
IPI:IPI00108194.1	MRPS26	Mitochondrial ribosomal protein S26	99	VQTPPAVDPAEFFVLTER	iTRAQ4plex@N-term		0.0	2159.11	720.71	2159.14	720.72	3	16.1	10.3	61.1	38.9	0.6
			99	VQTPPAVDPAEFFVLTER	iTRAQ4plex@N-term		0.1	2159.20	720.74	2159.14	720.72	3	17.7	16.7	51.4	48.6	0.9
			41	AQQAITEHR	iTRAQ4plex@N-term		0.0	1196.68	399.90	1196.64	399.89	3					
																**Average**	**0.8**
																**Stdev**	**0.2**
IPI:IPI00119138.1	UQCRC2	Ubiquinol cytochrome c reductase core protein 2	99	ITSEELHYFVQNHFTSAR	iTRAQ4plex@N-term		0.0	2322.13	581.54	2322.15	581.55	4	58.7	45.3	56.4	43.6	0.8
			99	LPNGLVIASLENYAPLSR	iTRAQ4plex@N-term		0.1	2070.23	691.08	2070.16	691.06	3	5.4	3.1	63.8	36.2	0.6
			99	LPNGLVIASLENYAPLSR	iTRAQ4plex@N-term		0.0	2070.15	691.06	2070.16	691.06	3	1.7	3.3	34.3	65.7	1.9
			98	YEDSNNLGTSHLLR	iTRAQ4plex@N-term		0.0	1761.86	588.29	1761.88	588.30	3	288.8	360.1	44.5	55.5	1.2
			11	SMAASGNLGHTPFLDEL	iTRAQ4plex@N-term		-10.9	1892.01	631.68	1902.93	635.32	3					
																**Average**	**1.1**
																**Stdev**	**0.6**

IPI:IPI00622837.1	COX6C	Cytochrome c oxidase polypeptide VIc	96	AYAEFYR	iTRAQ4plex@N-term		0.0	1062.54	532.28	1062.53	532.27	2	1940.6	2577.9	42.9	57.1	1.3
			78	FGVAEPR	iTRAQ4plex@N-term		0.0	918.50	460.26	918.50	460.26	2	3468.9	3768.0	47.9	52.1	1.1
																Average	1.2
																Stdev	0.2
IPI:IPI00116112.3	DCTN2	Dynactin 2	99	ASVEDADTQNK	iTRAQ4plex@N-term; No iTRAQ4plex(K)@11		0.0	1320.67	661.34	1320.63	661.32	2	101.6	73.0	58.2	41.8	0.7
			99	WSPVASTLPELVQR	iTRAQ4plex@N-term		0.0	1725.97	576.33	1725.95	576.33	3	28.5	21.7	56.7	43.3	0.8
			99	ASVEDADTQNK	iTRAQ4plex@N-term; Lys->Gln@11		0.1	1320.68	661.35	1320.59	661.30	2	49.7	42.6	53.8	46.2	0.9
			97	TGYESGDYEMLGEGLGVK	iTRAQ4plex@N-term; Lys->Gln@18		0.0	2047.92	683.65	2047.92	683.65	3	10.7	7.2	59.8	40.2	0.7
			96	GLDFSDR	iTRAQ4plex@N-term		0.0	952.48	477.25	952.47	477.24	2	1049.3	829.7	55.8	44.2	0.8
			78	WSPVASTLPELVQR	iTRAQ4plex@N-term		0.0	1725.95	576.33	1725.95	576.33	3	34.1	19.3	63.8	36.2	0.6
																Average	0.7
																Stdev	0.1
IPI:IPI00114279.1	SLC1A3	Solute carrier family 1 (glial high affinity glutamate transporter), member 3	99	DVEMGNSVIEENEMK	iTRAQ4plex@N-term; Lys->Gln@15		0.0	1866.83	623.29	1866.81	623.28	3	40.8	34.3	54.4	45.6	0.8
			99	IVQVTAADAFLDLIR	iTRAQ4plex@N-term		0.0	1788.03	895.02	1788.03	895.02	2	297.3	375.5	44.2	55.8	1.3
			99	TTTNVLGDSLGAGIVEHLSR	iTRAQ4plex@N-term		0.0	2183.16	728.73	2183.17	728.73	3	51.3	97.8	34.4	65.6	1.9
			99	TTTNVLGDSLGAGIVEHLSR	iTRAQ4plex@N-term		0.0	2183.14	728.72	2183.17	728.73	3	35.1	39.9	46.8	53.2	1.1
			99	DVEMGNSVIEENEMK	iTRAQ4plex@N-term; Deamidated(N)@6; iTRAQ4plex(K)@15		0.0	2011.95	671.66	2011.93	671.65	3	110.2	137.7	44.5	55.5	1.2
			99	DVEMGNSVIEENEMK	iTRAQ4plex@N-term; Lys->Arg@15		0.0	1894.85	632.62	1894.85	632.62	3	10.2	21.3	32.4	67.6	2.1
			32	LQQGVR	iTRAQ4plex@N-term		0.0	843.54	422.78	843.50	422.76	2					
			30	TSYEK	iTRAQ4plex@N-term; iTRAQ4plex(K)@5		0.0	914.53	458.27	914.50	458.25	2					
																Average	1.4
																Stdev	0.5
IPI:IPI00223714.5	HIST1H1E	Histone cluster 1, H1e	99	ALAAAGYDVEK	iTRAQ4plex@N-term; Lys->Gln@11		0.0	1250.66	626.34	1250.63	626.32	2	1046.5	556.4	65.3	34.7	0.5
			99	TSGPPVSELITK	iTRAQ4plex@N-term; Lys->Gln@12		0.0	1371.75	686.88	1371.74	686.88	2	259.3	216.6	54.5	45.5	0.8
			79	GTLVQTK	iTRAQ4plex@N-term; iTRAQ4plex(K)@7		0.0	1033.68	517.85	1033.64	517.83	2	1504.0	1006.1	59.9	40.1	0.7
			70	GTGASGSFK	iTRAQ4plex@N-term; Lys->Gln@9		0.1	954.53	478.27	954.45	478.23	2	84.7	34.7	71.0	29.0	0.4
																Average	0.6
																Stdev	0.2
IPI:IPI00133122.3	MRPL50	Mitochondrial 39S ribosomal protein L50	99	LLAGLADGLGHAVPNSR	iTRAQ4plex@N-term		0.0	1804.03	602.35	1804.01	602.34	3	251.8	169.0	59.8	40.2	0.7
			90	AYTPPSDLQSR	iTRAQ4plex@N-term		0.0	1377.70	689.86	1377.70	689.86	2	423.7	371.8	53.3	46.7	0.9
																Average	0.8
																Stdev	0.1
IPI:IPI00742433.1	HNRPUL1	Heterogeneous nuclear ribonucleoprotein U-like protein 1	99	WDVLIQQATQCLNR	iTRAQ4plex@N-term; Pyridylethyl(C)@11		0.0	1935.99	646.34	1936.01	646.34	3	12.3	13.7	47.4	52.6	1.1
			96	NYILDQTNVYGSAQR	iTRAQ4plex@N-term		0.0	1884.91	629.31	1884.95	629.32	3	95.7	135.0	41.5	58.5	1.4
																Average	1.3
																Stdev	0.2
IPI:IPI00118930.1	NAPA	Alpha-soluble NSF attachment protein	99	LDQWLTTMLLR	iTRAQ4plex@N-term		0.0	1532.84	767.43	1532.85	767.43	2	242.4	277.0	46.7	53.3	1.1
			99	LDQWLTTMLLR	iTRAQ4plex@N-term		0.1	1532.90	767.46	1532.85	767.43	2	155.4	202.2	43.5	56.5	1.3
			99	QAEAMALLAEAER	iTRAQ4plex@N-term		0.0	1545.81	773.91	1545.79	773.90	2	96.9	101.4	48.9	51.1	1.0
			99	QAEAMALLAEAER	iTRAQ4plex@N-term		0.0	1545.83	516.28	1545.79	516.27	3	19.4	19.8	49.5	50.5	1.0
			76	EYDSISR	iTRAQ4plex@N-term		0.0	1012.53	507.27	1012.49	507.25	2	304.9	568.0	34.9	65.1	1.9
																Average	1.3
																Stdev	0.3
IPI:IPI00132504.1	MRPS30	Mitochondrial ribosomal protein S30	99	DLEVVATPVAR	iTRAQ4plex@N-term		0.0	1312.72	657.37	1312.75	657.38	2	153.7	100.5	60.5	39.5	0.7
			98	SVDFYWLR	iTRAQ4plex@N-term		0.0	1228.64	615.33	1228.64	615.33	2	273.0	241.1	53.1	46.9	0.9
			62	SVDFYWLR	iTRAQ4plex@N-term		0.0	1228.63	615.32	1228.64	615.33	2	165.8	170.4	49.3	50.7	1.0
																Average	0.9
																Stdev	0.2
IPI:IPI00850430.1	SLC25A3	Solute carrier family 25 (mitochondrial carrier, phosphate carrier), member 3	99	GVAPLWMR	iTRAQ4plex@N-term		0.0	1072.57	537.29	1072.60	537.31	2	908.5	927.2	49.5	50.5	1.0
			99	IQTQPGYANTLR	iTRAQ4plex@N-term		1.0	1505.83	753.92	1504.81	753.41	2	830.7	702.1	54.2	45.8	0.8
																Average	0.9
																Stdev	0.1
IPI:IPI00131176.1	COX2	Cytochrome c oxidase subunit II	99	MLISSEDVLHSWAVPSLGLK	iTRAQ4plex@N-term; Lys->Gln@20		0.0	2325.23	776.09	2325.22	776.08	3	15.2	33.1	31.6	68.4	2.2
			99	VVLPMELPIR	iTRAQ4plex@N-term		0.0	1309.78	655.90	1309.79	655.90	2	221.4	550.7	28.7	71.3	2.5
			93	VVLPMELPIR	iTRAQ4plex@N-term		0.0	1309.79	655.90	1309.79	655.90	2	139.7	282.4	33.1	66.9	2.0
																Average	2.2
																Stdev	0.2
IPI:IPI00336292.2	MRPS9	Mitochondrial ribosomal protein S9	99	AIAYLFPSGLFEK	iTRAQ4plex@N-term; iTRAQ4plex(K)@13		-1.0	1742.00	581.67	1742.99	582.00	3	7.0	8.4	45.4	54.6	1.2
			99	ALCSFVTEDEVEWMR	iTRAQ4plex@N-term; Pyridylethyl(C)@3		0.0	2062.98	688.67	2062.96	688.66	3	15.6	24.4	39.0	61.0	1.6
			98	AIAYLFPSGLFEK	iTRAQ4plex@N-term; Lys->Gln@13		0.0	1598.88	800.45	1598.85	800.43	2	84.2	48.8	63.3	36.7	0.6
			94	AIAYLFPSGLFEK	iTRAQ4plex@N-term; Lys->Gln@13		0.1	1598.92	800.47	1598.85	800.43	2	74.6	60.1	55.4	44.6	0.8
			45	AIAYLFPSGLFEK	iTRAQ4plex@N-term; iTRAQ4plex(K)@13		-1.0	1741.99	581.67	1742.99	582.00	3					
																Average	1.0
																Stdev	0.4
IPI:IPI00408176.2	CSNK2A1	Casein kinase 2, alpha 1 polypeptide	99	FVHSENQHLVSPEALDFLDK	iTRAQ4plex@N-term; Lys->Gln@20		0.0	2468.22	618.06	2468.21	618.06	4	73.3	96.9	43.1	56.9	1.3
			99	GGPNIITLADIVKDPVSR	iTRAQ4plex@N-term; Lys->Gln@13		0.0	2008.15	670.39	2008.11	670.38	3	19.6	13.4	59.5	40.5	0.7
			99	GGPNIITLADIVKDPVSR	iTRAQ4plex@N-term; Lys->Gln@13		0.0	2008.12	670.38	2008.11	670.38	3	19.2	24.2	44.3	55.7	1.3
			91	FNDILGR	iTRAQ4plex@N-term		0.0	977.52	489.77	977.54	489.78	2	1720.4	1191.9	59.1	40.9	0.7
																Average	1.0
																Stdev	0.3
IPI:IPI00331182.4	GPD2	Glycerol phosphate dehydrogenase 2, mitochondrial	99	DDFSSGTSSR	iTRAQ4plex@N-term		0.0	1201.53	601.77	1201.53	601.77	2	182.6	210.8	46.4	53.6	1.2
			99	NYLSSDVEVR	iTRAQ4plex@N-term		0.0	1324.69	663.35	1324.67	663.34	2	396.9	505.8	44.0	56.0	1.3
			99	SADTQSISR	iTRAQ4plex@N-term		0.1	1107.63	554.82	1107.56	554.79	2	100.8	144.6	41.1	58.9	1.4
			99	VIFFLPWEK	iTRAQ4plex@N-term; Lys->Gln@9		0.1	1321.78	661.90	1321.72	661.87	2	61.3	74.6	45.1	54.9	1.2
			98	VIFFLPWEK	iTRAQ4plex@N-term; iTRAQ4plex(K)@9		2.0	1467.85	490.29	1465.86	489.63	3	10.4	17.0	37.9	62.1	1.6
			90	AGYLTEPVNR	iTRAQ4plex@N-term	cleaved A-A@N-term	0.0	1262.67	632.34	1262.67	632.34	2	208.8	393.9	34.6	65.4	1.9
			65	VIFFLPWEK	iTRAQ4plex@N-term; Lys->Gln@9		0.1	1321.79	661.90	1321.72	661.87	2	31.6	36.7	46.3	53.7	1.2
			28	VIFFLPWEK	iTRAQ4plex@N-term; Lys->Arg@9		0.1	1349.83	675.92	1349.76	675.89	2					
			15	MASVTGK	iTRAQ4plex@N-term; Lys->Gln@7		0.1	836.51	419.26	836.42	419.22	2					
																Average	1.4
																Stdev	0.3
IPI:IPI00381649.3	GFOD1	Glucose-fructose oxidoreductase domain containing 1	99	TQEEAEELAK	iTRAQ4plex@N-term; Lys->Gln@10		0.1	1290.66	646.34	1290.61	646.31	2	166.6	147.6	53.0	47.0	0.9
			98	AFSDIPSPYLR	iTRAQ4plex@N-term		0.0	1408.71	705.36	1408.75	705.38	2	242.4	177.2	57.8	42.2	0.7
			26	MMQAVR	iTRAQ4plex@N-term		0.0	878.49	440.25	878.46	440.24	2					
																Average	0.8
																Stdev	0.1
IPI:IPI00129485.3	FCGR2B	Fc receptor, IgG, low affinity IIb	99	EMGETLPEEVGEYR	iTRAQ4plex@N-term		0.0	1781.79	594.94	1781.83	594.95	3	8.8	8.4	51.4	48.6	0.9
			99	HPEALDEETEHDYQNHI	iTRAQ4plex@N-term		0.0	2220.02	556.01	2219.98	556.00	4	339.5	274.3	55.3	44.7	0.8
			99	SQVQASYTFK	iTRAQ4plex@N-term; Lys->Gln@10		0.0	1301.66	651.84	1301.64	651.83	2	697.4	345.3	66.9	33.1	0.5
			65	LEPPWIQVLK	iTRAQ4plex@N-term; iTRAQ4plex(K)@10		0.0	1509.92	504.31	1509.92	504.31	3	1.7	4.4	28.1	71.9	2.6
																Average	1.2
																Stdev	0.9
IPI:IPI00153660.4	DLAT	Dihydrolipoamide S-acetyltransferase (E2 component of pyruvate dehydrogenase complex)	99	GLETIASDVVSLASK	iTRAQ4plex@N-term; iTRAQ4plex(K)@15		0.0	1777.02	889.52	1777.01	889.51	2	68.0	40.9	62.5	37.5	0.6
			99	VAPAPAGVFTDIPISNIR	iTRAQ4plex@N-term		-0.1	1981.06	661.36	1981.11	661.38	3	62.2	49.6	55.7	44.3	0.8
			99	VPLPSLSPTMQAGTIAR	iTRAQ4plex@N-term		-0.1	1881.99	628.34	1882.05	628.36	3	55.6	30.2	64.8	35.2	0.5
			99	VAPAPAGVFTDIPISNIR	iTRAQ4plex@N-term		0.0	1981.08	661.37	1981.11	661.38	3	31.5	39.0	44.7	55.3	1.2
			99	VPLPSLSPTMQAGTIAR	iTRAQ4plex@N-term		0.0	1882.08	628.37	1882.05	628.36	3	21.1	24.2	46.6	53.4	1.1
			99	VAPAPAGVFTDIPISNIR	iTRAQ4plex@N-term; Dimethyl(R)@18		0.0	2009.19	670.74	2009.14	670.72	3	15.6	23.3	40.1	59.9	1.5
			99	GLETIASDVVSLASK	iTRAQ4plex@N-term; iTRAQ4plex(K)@15		0.0	1777.04	593.35	1777.01	593.34	3	12.4	10.4	54.5	45.5	0.8
			99	GLETIASDVVSLASK	iTRAQ4plex@N-term; Lys->Gln@15		0.1	1632.96	817.49	1632.87	817.44	2	22.9	25.2	47.6	52.4	1.1
			99	GLETIASDVVSLASK	iTRAQ4plex@N-term; Lys->Gln@15		0.1	1632.95	817.48	1632.87	817.44	2	21.4	15.5	58.0	42.0	0.7
			55	GIDLTQVK	iTRAQ4plex@N-term; Lys->Gln@8		0.0	1016.59	509.30	1016.56	509.29	2	337.1	238.6	58.6	41.4	0.7
			25	GLETIASDVVSLASK	iTRAQ4plex@N-term; Lys->Arg@15		0.1	1661.01	554.68	1660.91	554.64	3					
																Average	0.9
																Stdev	0.3
IPI:IPI00848780.1	MASP1	Mannan-binding lectin serine protease 1	99	DNEVMDTFQIECLK	iTRAQ4plex@N-term; Pyridylethyl(C)@12; iTRAQ4plex(K)@14		0.0	2076.98	693.34	2077.01	693.34	3	65.3	66.8	49.4	50.6	1.0
			99	SDFSNEER	iTRAQ4plex@N-term		0.1	1126.58	564.30	1126.50	564.26	2	29.9	32.5	47.9	52.1	1.1
			99	TGTITSPDYPNPYPK	iTRAQ4plex@N-term; iTRAQ4plex(K)@15		0.0	1938.01	647.01	1938.00	647.01	3	184.7	104.6	63.8	36.2	0.6
			99	IEPSQAVYSFK	iTRAQ4plex@N-term; Lys->Gln@11		0.0	1411.75	706.88	1411.71	706.86	2	619.5	473.9	56.7	43.3	0.8
			97	IEPSQAVYSFK	iTRAQ4plex@N-term; Lys->Gln@11		0.0	1411.75	706.88	1411.71	706.86	2	678.6	557.5	54.9	45.1	0.8
			89	IEPSQAVYSFK	iTRAQ4plex@N-term; iTRAQ4plex(K)@11		0.0	1555.83	519.62	1555.85	519.62	3	88.9	120.0	42.6	57.4	1.3
			23	TGTITSPDYPNPYPK	iTRAQ4plex@N-term; Lys->Gln@15		0.0	1793.87	598.97	1793.86	598.96	3					
																Average	0.9
																Stdev	0.3
IPI:IPI00339468.3	DHX9	DEAH (Asp-Glu-Ala-His) box polypeptide 9	99	AIEPPPLDAVIEAEHTLR	iTRAQ4plex@N-term		0.0	2114.13	705.72	2114.15	705.72	3	31.1	50.8	37.9	62.1	1.6
			99	YPSPFFVFGEK	iTRAQ4plex@N-term; iTRAQ4plex(K)@11		0.0	1604.85	535.96	1604.85	535.96	3	5.2	7.4	41.4	58.6	1.4
			98	DFVNYLVR	iTRAQ4plex@N-term		0.0	1168.65	585.33	1168.64	585.33	2	497.6	352.8	58.5	41.5	0.7
																Average	1.3
																Stdev	0.5
IPI:IPI00754918.2	C1R	Complement component 1, r subcomponent	99	ALDEFTIIQDPQPQYQFR	iTRAQ4plex@N-term		0.0	2352.23	785.08	2352.19	785.07	3	6.9	12.8	34.9	65.1	1.9
			99	QRPPDLDTSSNAVDLLFFTDESGDSR	iTRAQ4plex@N-term		0.0	3025.44	1009.49	3025.44	1009.49	3	21.3	18.7	53.2	46.8	0.9
			99	ALDEFTIIQDPQPQYQFR	iTRAQ4plex@N-term		0.0	2352.21	785.08	2352.19	785.07	3	19.7	60.8	24.5	75.5	3.1
			99	QRPPDLDTSSNAVDLLFFTDESGDSR	iTRAQ4plex@N-term		0.1	3025.49	1009.51	3025.44	1009.49	3	10.8	5.0	68.3	31.7	0.5
			99	WILTAAHTIYPK	iTRAQ4plex@N-term; Lys->Gln@12		0.0	1556.87	519.96	1556.85	519.96	3	58.6	48.6	54.7	45.3	0.8
			98	WILTAAHTIYPK	iTRAQ4plex@N-term; Lys->Arg@12		0.0	1584.91	529.31	1584.89	529.30	3	55.5	82.3	40.3	59.7	1.5
																Average	1.4
																Stdev	0.9
IPI:IPI00621806.2	CAMK2A	Calcium/calmodulin-dependent protein kinase II alpha	99	AGAYDFPSPEWDTVTPEAK	iTRAQ4plex@N-term; iTRAQ4plex(K)@19		0.0	2368.18	790.40	2368.15	790.39	3	43.9	51.3	46.1	53.9	1.2
			99	FYFENLWSR	iTRAQ4plex@N-term		0.0	1404.73	703.37	1404.69	703.35	2	186.4	156.6	54.3	45.7	0.8
			99	FYFENLWSR	iTRAQ4plex@N-term		0.0	1404.73	703.37	1404.69	703.35	2	185.9	168.6	52.5	47.5	0.9
			99	VTEQLIEAISNGDFESYTK	iTRAQ4plex@N-term; iTRAQ4plex(K)@19		0.1	2431.29	811.44	2431.24	811.42	3	10.7	8.3	56.3	43.7	0.8
			99	VTEQLIEAISNGDFESYTK	iTRAQ4plex@N-term; iTRAQ4plex(K)@19		0.0	2431.21	811.41	2431.24	811.42	3	5.3	5.2	50.4	49.6	1.0
			99	AGAYDFPSPEWDTVTPEAK	iTRAQ4plex@N-term; Lys->Gln@19		0.0	2224.04	742.35	2224.01	742.34	3	24.2	38.2	38.8	61.2	1.6
			93	FTEEYQLFEELGK	iTRAQ4plex@N-term; iTRAQ4plex(K)@13		-1.0	1919.01	640.68	1919.98	641.00	3	12.1	20.2	37.4	62.6	1.7
																Average	1.1
																Stdev	0.4
IPI:IPI00469307.2	LRPAP1	Low density lipoprotein receptor-related protein associated protein 1	99	AEEGYENLLSPSDMAH	iTRAQ4plex@N-term	cleaved H-I@C-term	0.0	1905.90	636.31	1905.85	636.29	3	5.1	10.7	32.4	67.6	2.1
			99	IQEYNVLLDTLSR	iTRAQ4plex@N-term		0.0	1706.97	854.49	1706.93	854.47	2	227.5	184.2	55.3	44.7	0.8
			99	IQEYNVLLDTLSR	iTRAQ4plex@N-term		0.0	1706.96	854.49	1706.93	854.47	2	197.7	148.7	57.1	42.9	0.8
																Average	1.2
																Stdev	0.8
IPI:IPI00753793.2	SPNA2	Spectrin alpha 2	99	AQLADSFHLQQFFR	iTRAQ4plex@N-term		0.0	1850.92	617.98	1850.95	617.99	3	22.8	27.4	45.4	54.6	1.2
			99	DLMSWINGIR	iTRAQ4plex@N-term		0.0	1347.71	674.86	1347.71	674.86	2	59.6	70.5	45.8	54.2	1.2
			99	DLMSWINGIR	iTRAQ4plex@N-term		0.0	1347.71	674.86	1347.71	674.86	2	57.6	76.9	42.8	57.2	1.3
			84	EANQQQQFNR	iTRAQ4plex@N-term		0.1	1405.74	469.59	1405.68	469.57	3	7.0	8.4	45.4	54.6	1.2
			59	TLAAER	iTRAQ4plex@N-term		0.0	803.50	402.76	803.46	402.74	2	56.5	55.2	50.6	49.4	1.0
			19	NIQSK	iTRAQ4plex@N-term; Deamidated(Q)@3; iTRAQ4plex(K)@5	cleaved T-N@N-term	0.0	877.54	439.78	877.51	439.76	2					
																Average	1.2
																Stdev	0.1
IPI:IPI00113539.2	FN1	Fibronectin 1	99	GVTYNIIVEALQNQR	iTRAQ4plex@N-term		0.0	1861.04	621.35	1861.02	621.35	3	14.3	9.2	60.8	39.2	0.6
			99	WLPSTSPVTGYR	iTRAQ4plex@N-term		0.0	1506.82	754.42	1506.80	754.40	2	1352.1	804.6	62.7	37.3	0.6
			99	WLPSTSPVTGYR	iTRAQ4plex@N-term		0.0	1506.80	754.41	1506.80	754.40	2	1068.8	597.8	64.1	35.9	0.6
			99	TETITGFQVDAIPANGQTPVQR	iTRAQ4plex@N-term		0.0	2486.24	829.76	2486.29	829.77	3	5.3	5.2	50.4	49.6	1.0
			92	STTPDITGYR	iTRAQ4plex@N-term		0.0	1253.66	627.84	1253.64	627.83	2	373.5	244.7	60.4	39.6	0.7
			84	DAPIVNR	iTRAQ4plex@N-term		0.1	927.58	464.80	927.53	464.77	2	894.3	1013.7	46.9	53.1	1.1
			28	STTPDITGYR	iTRAQ4plex@N-term		0.0	1253.65	627.83	1253.64	627.83	2					
																Average	0.8
																Stdev	0.2
IPI:IPI00460042.3	RALGAPA1	Ral GTPase activating protein, alpha subunit 1	99	SLIPLYQNFYEER	iTRAQ4plex@N-term		0.0	1814.96	605.99	1814.93	605.98	3	41.8	58.0	41.9	58.1	1.4
			99	SLIPLYQNFYEER	iTRAQ4plex@N-term		0.0	1814.95	605.99	1814.93	605.98	3	28.6	19.6	59.4	40.6	0.7
			99	SSSTSDILEPFTVER	iTRAQ4plex@N-term		0.0	1810.94	604.65	1810.91	604.64	3	18.1	5.8	75.6	24.4	0.3
			98	NLYSLDLSDLPLDK	iTRAQ4plex@N-term; Lys->Gln@14		0.0	1748.93	875.47	1748.90	875.45	2	14.1	14.7	49.0	51.0	1.0
			88	DLPDILNK	iTRAQ4plex@N-term; Lys->Gln@8		0.0	1070.59	536.30	1070.57	536.29	2	173.0	121.3	58.8	41.2	0.7
			78	STAINASR	iTRAQ4plex@N-term		0.0	962.57	482.29	962.53	482.27	2	46.4	30.8	60.1	39.9	0.7
																Average	0.8
																Stdev	0.4
IPI:IPI00117011.3	HCLS1	Hematopoietic cell specific Lyn substrate 1	99	EVQQPSMPVEEPAAPAQLPK	iTRAQ4plex@N-term; iTRAQ4plex(K)@20		0.0	2433.24	812.09	2433.28	812.10	3	90.8	70.1	56.4	43.6	0.8
			99	TTPIEAASSGAR	iTRAQ4plex@N-term		2.1	1305.76	653.89	1303.69	652.85	2	674.4	522.5	56.3	43.7	0.8
			99	TTPIEAASSGAR	iTRAQ4plex@N-term		0.0	1303.71	652.86	1303.69	652.85	2	402.6	349.0	53.6	46.4	0.9
			99	EVQQPSMPVEEPAAPAQLPK	iTRAQ4plex@N-term; iTRAQ4plex(K)@20		0.0	2433.26	812.10	2433.28	812.10	3	111.0	118.0	48.5	51.5	1.1
			99	EVQQPSMPVEEPAAPAQLPK	iTRAQ4plex@N-term; Lys->Gln@20		0.0	2289.19	764.07	2289.14	764.06	3	30.4	18.2	62.6	37.4	0.6
			59	TTPIEAASSGAR	iTRAQ4plex@N-term		0.0	1303.72	652.87	1303.69	652.85	2	116.1	126.7	47.8	52.2	1.1
																Average	0.9
																Stdev	0.2
IPI:IPI00120302.1	LGI1	Leucine-rich repeat LGI family, member 1	99	GLDSLTNVDLR	iTRAQ4plex@N-term		0.0	1345.71	673.86	1345.73	673.87	2	437.3	402.7	52.1	47.9	0.9
			75	DFDCIITEFAK	iTRAQ4plex@N-term; Pyridylethyl(C)@4; Lys->Gln@11		0.1	1549.78	517.60	1549.72	517.58	3	30.0	29.3	50.6	49.4	1.0
			58	DFDCIITEFAK	iTRAQ4plex@N-term; Pyridylethyl(C)@4; Lys->Arg@11		0.0	1577.79	526.94	1577.77	526.93	3	8.8	8.4	51.4	48.6	0.9
																Average	0.9
																Stdev	0.0
IPI:IPI00785299.1	ATP2B3	ATPase, Ca++ transporting, plasma membrane 3	99	GIIDSTTGEQR	iTRAQ4plex@N-term		0.0	1319.72	660.87	1319.68	660.85	2	211.0	185.8	53.2	46.8	0.9
			99	VYTFNSVR	iTRAQ4plex@N-term		0.0	1128.58	565.30	1128.60	565.31	2	1398.5	1287.9	52.1	47.9	0.9
			99	MVTGDNINTAR	iTRAQ4plex@N-term		0.0	1334.67	668.34	1334.67	668.34	2	742.2	720.5	50.7	49.3	1.0
			98	IQEAYGDVSGLCR	iTRAQ4plex@N-term; Pyridylethyl(C)@12		0.0	1658.79	553.94	1658.82	553.95	3	78.0	117.1	40.0	60.0	1.5
			73	SAEGGEMEER	iTRAQ4plex@N-term		0.0	1237.53	619.77	1237.54	619.78	2	67.7	51.5	56.8	43.2	0.8
			35	IEQEQK	iTRAQ4plex@N-term; iTRAQ4plex(K)@6		0.1	1061.66	531.84	1061.60	531.81	2					
			34	TGTLTTNR	iTRAQ4plex@N-term		0.0	1006.59	504.30	1006.55	504.28	2					
			22	GEIEQER	iTRAQ4plex@N-term		0.0	1003.54	502.78	1003.51	502.76	2					
																Average	1.0
																Stdev	0.3
IPI:IPI00475157.1	SERPINA1B	Serine (or cysteine) preptidase inhibitor, clade A, member 1B	99	IFNNGADLSGITEENAPLK	iTRAQ4plex@N-term; No iTRAQ4plex(K)@19		0.0	2146.08	716.37	2146.10	716.37	3	30.0	31.4	48.8	51.2	1.0
			99	TLMSPLGITR	iTRAQ4plex@N-term		1.0	1232.69	617.35	1231.71	616.86	2	2253.5	1626.4	58.1	41.9	0.7
			99	TLMSPLGITR	iTRAQ4plex@N-term		0.0	1231.68	616.85	1231.71	616.86	2	1079.7	799.9	57.4	42.6	0.7
																Average	0.8
																Stdev	0.2
IPI:IPI00271726.5	MRPL28	Mitochondrial ribosomal protein L28	99	VYVEELVQR	iTRAQ4plex@N-term		0.0	1277.69	639.85	1277.71	639.86	2	762.6	512.9	59.8	40.2	0.7
			98	VYVEELVQR	iTRAQ4plex@N-term		0.0	1277.71	639.86	1277.71	639.86	2	570.9	400.8	58.8	41.2	0.7
			30	SLEEER	iTRAQ4plex@N-term		0.0	905.49	453.75	905.46	453.74	2					
																Average	0.7
																Stdev	0.0
IPI:IPI00407898.1	IGH-6	Immunoglobulin heavy constant mu	99	DLHVPIPAVAEMNPNVNVFVPPR	iTRAQ4plex@N-term		0.0	2668.43	668.12	2668.43	668.11	4	105.8	158.6	40.0	60.0	1.5
			90	ATLTADK	iTRAQ4plex@N-term; iTRAQ4plex(K)@7		0.0	1006.61	504.31	1006.59	504.30	2	6329.6	6755.2	48.4	51.6	1.1
			69	GLEWIGR	iTRAQ4plex@N-term		0.0	973.54	487.78	973.55	487.78	2	3753.5	2841.6	56.9	43.1	0.8
			32	ATLTADK	iTRAQ4plex@N-term; Lys->Arg@7		0.0	890.52	446.27	890.49	446.25	2					
																Average	1.1
																Stdev	0.4
IPI:IPI00556827.1	ATP2B1	ATPase, Ca++ transporting, plasma membrane 1	99	GIIDSTVSEQR	iTRAQ4plex@N-term		0.0	1347.71	674.86	1347.71	674.86	2	159.1	153.0	51.0	49.0	1.0
			99	VYTFNSVR	iTRAQ4plex@N-term		0.0	1128.58	565.30	1128.60	565.31	2	1398.5	1287.9	52.1	47.9	0.9
			99	MVTGDNINTAR	iTRAQ4plex@N-term		0.0	1334.67	668.34	1334.67	668.34	2	742.2	720.5	50.7	49.3	1.0
			38	STDALR	iTRAQ4plex@N-term		0.0	805.47	403.74	805.44	403.73	2					
			35	IEQEQK	iTRAQ4plex@N-term; iTRAQ4plex(K)@6		0.1	1061.66	531.84	1061.60	531.81	2					
			22	GEIEQER	iTRAQ4plex@N-term		0.0	1003.54	502.78	1003.51	502.76	2					
																Average	1.0
																Stdev	0.0
IPI:IPI00109904.3	GALC	Galactosylceramidase	99	EFDGIGAVSGGGATSR	iTRAQ4plex@N-term		0.0	1623.78	542.27	1623.80	542.27	3	146.4	148.3	49.7	50.3	1.0
			99	YVLDDSDGLGR	iTRAQ4plex@N-term	cleaved A-Y@N-term	0.0	1352.62	677.32	1352.67	677.34	2	1766.9	1172.6	60.1	39.9	0.7
			99	YVLDDSDGLGR	iTRAQ4plex@N-term	cleaved A-Y@N-term	0.0	1352.67	677.34	1352.67	677.34	2	525.1	351.6	59.9	40.1	0.7
																Average	0.8
																Stdev	0.2
IPI:IPI00120914.1	EIF3F	Eukaryotic translation initiation factor 3, subunit F	99	LHPVILASIVDSYER	iTRAQ4plex@N-term		0.0	1855.00	619.34	1855.03	619.35	3	17.7	16.7	51.4	48.6	0.9
			99	GMNTLVGYDLVPEPK	iTRAQ4plex@N-term; No iTRAQ4plex(K)@15		0.0	1775.96	888.99	1775.92	888.97	2	44.1	44.9	49.5	50.5	1.0
			99	LNDFASAVR	iTRAQ4plex@N-term		0.0	1135.61	568.81	1135.61	568.81	2	1713.0	2796.5	38.0	62.0	1.6
			78	VSADNTVGR	iTRAQ4plex@N-term		0.0	1061.60	531.81	1061.56	531.79	2	1057.8	946.3	52.8	47.2	0.9
			70	IIDAALR	iTRAQ4plex@N-term		0.0	914.56	458.29	914.57	458.29	2	2348.9	2770.8	45.9	54.1	1.2
			55	WVTYFNKPDIDAWELRK	iTRAQ4plex@N-term; Lys->Gln@7; Lys->Gln@17	missed R-K@16	0.1	2324.19	582.06	2324.13	582.04	4	23.3	12.0	66.0	34.0	0.5
																Average	1.0
																Stdev	0.4
IPI:IPI00111211.1	MRPL46	Mitochondrial ribosomal protein L46	99	ALDEAQR	iTRAQ4plex@N-term		-6.0	939.55	470.78	945.50	473.76	2	75.2	44.9	62.6	37.4	0.6
			99	SLEAASSSSR	iTRAQ4plex@N-term	cleaved L-S@N-term	0.1	1137.65	569.83	1137.57	569.79	2	223.1	152.9	59.3	40.7	0.7
			22	ALDEAQR	iTRAQ4plex@N-term		0.1	945.56	473.79	945.50	473.76	2					
																Average	0.6
																Stdev	0.1
IPI:IPI00119033.1	ICAM5	Intercellular adhesion molecule 5, telencephalin	99	LEPFWADLQPR	iTRAQ4plex@N-term	cleaved A-L@N-term	0.0	1514.79	505.94	1514.80	505.94	3	51.7	38.1	57.6	42.4	0.7
			97	LEPFWADLQPR	iTRAQ4plex@N-term	cleaved A-L@N-term	0.0	1514.82	758.42	1514.80	758.41	2	535.5	469.5	53.3	46.7	0.9
			25	EDAGTYR	iTRAQ4plex@N-term		0.0	954.49	478.25	954.45	478.23	2					
																Average	0.8
																Stdev	0.1
IPI:IPI00323079.1	APP	Amyloid beta A4 protein precursor	99	VESLEQEAANER	iTRAQ4plex@N-term		0.0	1517.76	759.89	1517.74	759.88	2	184.3	163.1	53.1	46.9	0.9
			13	MLNDR	iTRAQ4plex@N-term; Deamidated(N)@3	cleaved A-M@N-term	0.0	792.44	397.23	792.39	397.20	2					
																Average	0.9
																Stdev	0.0
IPI:IPI00223070.3	DOCK4	Dedicator of cytokinesis protein 4	99	LDWGNEQLGLDLVPR	iTRAQ4plex@N-term		0.0	1867.97	623.66	1867.99	623.67	3	5.0	12.8	28.1	71.9	2.6
			99	LDWGNEQLGLDLVPR	iTRAQ4plex@N-term		0.0	1868.01	623.68	1867.99	623.67	3	10.7	8.3	56.3	43.7	0.8
			79	MMEASLYDK	iTRAQ4plex@N-term; Lys->Gln@9		0.0	1230.57	616.29	1230.54	616.28	2	234.9	180.5	56.5	43.5	0.8
			66	LEPEFFR	iTRAQ4plex@N-term		0.0	1080.56	541.29	1080.57	541.29	2	958.0	674.3	58.7	41.3	0.7
			65	FPFFLR	iTRAQ4plex@N-term		0.0	969.55	485.78	969.56	485.79	2	97.5	86.2	53.1	46.9	0.9
																Average	1.1
																Stdev	0.8
